# The effects of exercise on secondary prevention and health-related quality of life in people with existing vascular disease: systematic review and meta-analysis of randomised controlled trials

**DOI:** 10.1016/j.eclinm.2025.103201

**Published:** 2025-05-09

**Authors:** Cathryn Broderick, Marlene Stewart, Katie Thomson, Ceri Sellers, Candida Fenton, Julie Cowie, Wei Xu, Christa St Jean, Keira Charteris, Madhurima Nundy, Vaishali Vardhan, Prerna Krishan, Jolie Pistol, Leonor Rodríguez, Alex Todhunter-Brown, Frederike van Wijck, Sheila Cameron, Catriona Keerie, Rod S. Taylor, Gerry Stansby, Gillian Mead, NESSIE, NIHR Evidence Synthesis Scotland Initiative

**Affiliations:** a NESSIE, Usher Institute, University of Edinburgh, Scotland, UK; b NESSIE, Glasgow Caledonian University, Scotland, UK; c UNCOVER: Applied Evidence Synthesis, Usher Institute, University of Edinburgh, Scotland, UK; d School of Health in Social Science, University of Edinburgh, Scotland, UK; e Glasgow Caledonian University, Scotland, UK; f NESSIE, Scotland, UK; g ECTU, Usher Institute, University of Edinburgh, Scotland, UK; h MRC/CSO Social and Public Health Sciences Unit & Robertson Centre for Biostatistics, School of Health and Well Being, College of Medical, Veterinary and Life Sciences, University of Glasgow, Scotland, UK; i University of Newcastle, England, UK; j Ageing and Health, Usher Institute, University of Edinburgh, Scotland, UK

**Keywords:** Exercise, Secondary prevention, Vascular disease, Polyvascular, Systematic review, Meta-analysis

## Abstract

**Background:**

Polyvascular disease (atherosclerosis across two or more vascular beds) is becoming increasingly common, yet systematic reviews of interventions such as exercise are traditionally targeted at people with a single disease. We aimed to determine the effect of exercise in the secondary prevention of major adverse cardiovascular events and health-related quality of life (HRQoL) in people with an existing vascular disease and to assess the impact of polyvascular disease.

**Methods:**

For this systematic review and meta-analysis, we searched databases (Cochrane Register of Studies Online, MEDLINE, Embase Ovid, CINAHL EBSCO, WHO-ICTRP and ClinicalTrials.gov) in January 2025 for randomised controlled trials (RCTs) of exercise in people with coronary artery disease, heart failure, stroke (including transient ischaemic attack (TIA)) and peripheral arterial disease (PAD). We excluded studies where exercise was delivered for <6 weeks. Two reviewers independently assessed articles for eligibility and extracted data. Disagreements were resolved through discussion. Critical outcomes were mortality (all-cause and cardiovascular-specific), vascular events (myocardial infarction, stroke, amputation, acute limb ischaemia (ALI)), vascular hospitalisations, and HRQoL (EQ-5D and SF-36). We extracted data at end of intervention, medium term (6–30 months follow-up), and long term (>30 months follow-up). We performed random-effects meta-analyses. Risk of bias was assessed using Cochrane's Risk of Bias 1 tool. The certainty of the evidence was assessed using GRADE. PROSPERO registration: CRD42024517019.

**Findings:**

We included 280 RCTs involving 23,419 participants. 114 (40·71%) studies did not report whether their populations had more than one vascular disease. Exercise may result in little to no difference in all-cause mortality compared to no exercise at end of intervention (risk ratio (RR) 0·92, 95% confidence interval (CI) 0·80–1·07; P = 0·30; 143 studies, 12,811 participants; low-certainty evidence). Similar effects were found at medium and long term. Exercise may result in little to no difference in cardiovascular mortality compared to no exercise at end of intervention (RR 0·92, 95% CI 0·75–1·12; P = 0·41; 77 studies, 7319 participants; low-certainty evidence). A similar effect was found at medium term. At long term there may be a difference favouring exercise on cardiovascular mortality (RR 0·81, 95% CI 0·64–1·01; P = 0·06; 10 studies, 3935 participants). Exercise probably reduces vascular hospitalisations compared to no exercise at end of intervention (RR 0·73, 95% CI 0·56–0·95; P = 0·02; 64 studies, 7101 participants; moderate-certainty evidence) and medium term (RR 0·83, 95% CI 0·70–0·99; P = 0·04; 49 studies, 7514 participants; low-certainty evidence), with little or no difference at long term. Exercise probably increases HRQoL as assessed by EQ-5D compared to no exercise at end of intervention (mean difference (MD), 6·20, 95% CI 2·21–10·20; P = 0·002; 8 studies, 805 participants; moderate-certainty evidence), with little or no difference at medium term (MD 2·23, 95% CI –3·19 to 7·66; P = 0·42; 7 studies, 707 participants; moderate-certainty evidence) and long term (MD 6·00, 95% CI –2·05 to 14·05; P = 0·14; 1 study, 73 participants). Exercise probably increases HRQoL as assessed by SF-36 compared to no exercise at end of intervention (MD 6·83, 95% CI 5·22–8·44; P < 0·0001; 50 studies, 3231 participants; moderate-certainty evidence) and medium term (MD 6·44, 95% CI 3·71–9·18; P < 0·0001; 15 studies, 1522 participants; moderate-certainty evidence). No studies reported SF-36 at long term. Data on vascular events were mixed and of low certainty. Evidence was limited, and therefore uncertain, for amputation and ALI. Limiting issues were poor descriptions of exercise, and poor, inconsistently reported study inclusion and exclusion criteria, therefore limiting our ability to categorise included populations as polyvascular/single.

**Interpretation:**

We believe this systematic review and meta-analysis to be the first to combine RCTs with vascular diseases and examine the effects of exercise in people with single conditions and polyvascular disease. We found consistent evidence that exercise improves HRQoL and reduces hospitalisations across vascular disease but does not appear to impact mortality. However, the vast majority of trials were designed to target people with a single vascular condition and did not report the presence of additional vascular diseases. Therefore, it was not possible to formally assess the impact of the addition of polyvascular disease on exercise outcomes or determine the applicability of our findings to a population with polyvascular disease. More trials are needed that include participants with polyvascular conditions to strengthen the evidence on safety of this intervention, in order to inform clinical guidelines.

**Funding:**

This study was funded by the 10.13039/100006662NIHR Evidence Synthesis Programme (NIHR162044).

**Research in context**

**Evidence before this study**

In a preliminary search of Medline and Embase, we scoped the existing evidence on exercise for the secondary prevention of vascular disease between 2016 and 2024. Our search terms were: polyvascular, polyVD, panvascular, comorbidity, multimorbidity, range of vascular disorders, multiple long-term conditions, and exercise. No reviews focused on people with polyvascular diseases, despite this becoming more common. One large overview of systematic reviews in people with long-term conditions found evidence gaps for mortality, hospitalisations and consideration of the impact of multi-morbidity to exercise-based interventions. One systematic review estimated the prevalence of chronic comorbid conditions reported in heart failure clinical trials and concluded there is a need to improve recruitment of multi-morbid patients in trials. This did not further investigate secondary prevention or effect of exercise. An earlier meta-epidemiological review included pre-diabetes, CHD, stroke and heart failure and found limited quality evidence that exercise and drugs had similar effects on mortality outcomes, but did not report PAD or extract information about vascular comorbidities from the included reviews. Another meta-analysis included participants with hypertension or diabetes or cardiovascular diseases and demonstrated a benefit in mortality from exercise. This did not investigate effects on people with polyvascular conditions.

**Added value of this study**

This systematic review and meta-analysis is the first to combine data from 280 RCTs in people with one or more vascular conditions. The meta-analysis provides evidence that exercise in people with heart failure, coronary artery disease, stroke, and/or PAD improved health-related quality of life and reduced hospitalisations with no difference in all-cause or cardiovascular-specific mortality. Fewer than half of the 280 included trials reported vascular comorbidities in people with a single vascular condition, thus restricting the applicability of the evidence to people with polyvascular disease. This highlights the evidence gap regarding guidance on exercise for people with polyvascular conditions.

**Implications of all the available evidence**

This systematic review highlights the safety and benefits of exercise for people with vascular disease. Exercise should be recommended by health care professionals and pathways into exercise should be developed for people with more than one vascular condition. It is imperative that trialists systematically report the presence of vascular comorbidities so that systematic reviewers can extract this information and include in subgroup analyses, so the generalisability of the evidence to people with polyvascular disease can be determined. These findings can be used to inform future research and guideline development.

## Introduction

Globally, cardiovascular diseases (CVDs) including coronary heart disease, cerebrovascular disease, and peripheral arterial disease, collectively remain the leading causes of death and substantially contribute to loss of health and excess health system costs.^1^ In 2017, approximately 17·8 million deaths were attributed to CVD globally, making it the leading cause of death.^2^^,^^3^

Atherosclerosis is the common underlying aetiological factor in CVD and people with coronary artery disease, stroke or peripheral arterial disease often have atheroma in more than one arterial bed; if symptomatic, this is termed ‘polyvascular disease’.^3^ Large scale registry studies have shown that people with polyvascular disease have a higher risk of major adverse cardiovascular events (cardiovascular death, myocardial infarction or stroke) and major adverse limb events (severe limb ischaemia leading to an intervention or major vascular amputation) compared to those with disease in a single arterial territory.^4^^,^^5^

Despite this, systematic reviews and meta-analyses of randomised controlled trials in heart, stroke and circulatory diseases focus on single organ conditions, and review authors tend not to consider the effect of an intervention in people with disease in more than one vascular territory. Clinical guidelines also generally focus on people with a single vascular disease and may not be generalisable to people with polyvascular disease.^6^

An example of an intervention recommended for people with the index conditions of coronary heart disease, cerebrovascular disease or peripheral arterial disease is exercise, defined as ‘‘ … a type of physical activity consisting of planned, structured, and repetitive bodily movement done to improve and/or maintain one or more components of physical fitness”.^7^, ^8^, ^9^, ^10^, ^11^, ^12^, ^13^, ^14^, ^15^, ^16^ In these conditions, exercise improves physical function and reduces the risk of further vascular events.^17^, ^18^, ^19^ Exercise training can target any of the health- and/or skill-related fitness parameters, defined by the American College of Sports Medicine,^19^ and commonly includes aerobic, strength or mixed training. Some exercise programmes may include exercise training alone or in combination with psychological or educational interventions.^19^, ^20^, ^21^

Exercise reduces the risk of recurrent vascular events through multiple effects including on lipid metabolism, vascular biology, and inflammatory and stress response.^22^, ^23^, ^24^

However, systematic reviews and meta-analyses, including Cochrane reviews, focus on exercise for a single vascular disease,^20^^,^^25^, ^26^, ^27^, ^28^ and do not systematically comment on the implications of their results for people with polyvascular disease.^29^, ^30^, ^31^, ^32^, ^33^, ^34^, ^35^ There are no systematic reviews investigating the effectiveness of exercise for secondary prevention in people with polyvascular disease.^36^^,^^37^ Similarly, guidelines recommending exercise for people with a single index vascular condition do not consider the impact of exercise on people with polyvascular disease.^13^^,^^38^^,^^39^ Yet a high prevalence of polyvascular disease is indicated by registries and studies investigating single vascular conditions (e.g., 10·8% chronic HF with reduced ejection fraction to 71·0% in PAD population).^5^^,^^40^, ^41^, ^42^, ^43^ To address the specific exercise requirements in people with polyvascular disease to improve their health, clinicians need to better understand generalisability of the current evidence about the effectiveness of exercise (and any risks) in people with more than one vascular disease. Therefore, all the evidence about the effects of exercise in people with vascular disease needs to be collated into a single review, the characteristics of patients described by their index/additional vascular diseases, and analyses need to explore the impact of polyvascular disease on outcomes. Furthermore, exercise needs to be defined in a standardised manner, as previous reviews, including Cochrane reviews, have used different definitions of exercise. It is important to determine whether there are differences in effects and applicability between types of exercise programmes, and whether the addition of education/psychological interventions influences the effect of exercise. Current services are often focused on single diseases, with anecdotal evidence of exclusion of people with polyvascular disease; fully understanding the effects of exercise on people with polyvascular disease will provide opportunities for efficient service delivery and ensure patients receive optimal care. Thus, a high-quality review and meta-analysis could provide the necessary impetus for clinicians and commissioners to work more closely together to ensure that people with vascular diseases (as defined above) will receive optimum care.

We aimed to determine the effect of exercise on the secondary prevention of the critical outcomes major adverse cardiovascular and limb events and health-related quality of life (HRQoL) in people with existing vascular disease as the index condition (at least one or more of coronary artery disease (including angina), heart failure, stroke (including transient ischaemic attack (TIA)), and peripheral arterial disease (PAD)). The critical outcomes were selected as they are of major importance to people with vascular disease and their families and clinicians.^44^, ^45^, ^46^ We also aimed to investigate the effect of exercise in people with a single vascular disease and polyvascular disease by subgroup analyses. We aimed to use subgroup analyses to investigate the effect of exercise in different vascular conditions (coronary artery disease (including angina), heart failure, stroke (including TIA), and PAD), different aetiologies (ischaemic cause or mixed cause (i.e., study included some participants with non-ischaemic causes of stroke or heart failure). We also aimed to use subgroup analyses to investigate exercise parameters by type of exercise (cardiorespiratory/aerobic or resistance/strength alone or mixed), exercise intensity (high or low/light/moderate or not reported or unclear), and frequency and duration of exercise session (total dose of exercise).

## Methods

### Search strategy and selection criteria

A systematic review with meta-analysis was undertaken. Our protocol (Supplementary information) was registered with the international prospective register of systematic reviews (PROSPERO) on 04.03.24 CRD42024517019.

Patient and public involvement (PPI) was integral to our review and informed by UK Standards.^47^ See Supplementary information for more details.

We undertook a two-way approach for this systematic review. First, we identified relevant randomised controlled trials (RCTs) from five key Cochrane reviews to reduce duplication of effort and expedite the review process.^20^^,^^25^, ^26^, ^27^, ^28^ Secondly, we undertook new searches to identify RCTs published after the Cochrane review search dates as well as for conditions not covered by original searches (e.g., TIA). Searches were conducted of the following databases for RCTs and controlled clinical trials without language, or publication status restrictions (Cochrane Central Register of Controlled Trials via the Cochrane Register of Studies Online (CRSO); MEDLINE (Ovid MEDLINE Epub Ahead of Print, In-Process & Other Non-Indexed Citations, Ovid MEDLINE Daily and Ovid MEDLINE; Embase Ovid; CINAHL EBSCO; World Health Organization International Clinical Trials Registry Platform (who.int/trialsearch); and ClinicalTrials.gov (clinicaltrials.gov). Searches were restricted by date starting from 1 January 2016. The most recent searches were carried out on 20 January 2025. See Supplementary information for search strategy.

Two independent NESSIE reviewers undertook title, abstract and full-text screening in Covidence systematic review software.^48^ Where necessary consensus was reached by team discussion. Consultation with PPI members informed decisions on exercise types that met our inclusion criteria. We only included RCTs, including individual randomised, cluster-randomised and cross-over trials (first phase only) with any length of follow up. We excluded studies which used a quasi-randomisation process (e.g., alternate allocation) or if no English full text was available.

Relevant RCTs included adults (18+) diagnosed with one or more vascular conditions including stroke, TIA, coronary artery disease, angina, heart failure, and PAD. No restrictions were placed on geographical location, gender or ethnic background. Where populations were mixed (vascular and non-vascular conditions) we included when >80% had vascular conditions of interest and reported this.

We included RCTs investigating exercise which incorporated planned, structured, and repetitive bodily movement to improve or maintain strength and/or cardiorespiratory fitness.^17^, ^18^, ^19^ Exercise interventions were of a minimum duration of six weeks, delivered at any timepoint, setting or format by any individual. We excluded exercise solely aimed at improving skills such as balance and coordination (rather than health-related fitness) or provided advice only with no training component. Comparators included usual care, attention control or co-interventions (interventions delivered to all treatment groups in addition to exercise) such as dietary advice–provided the effect of exercise could be determined.

### Data extraction and quality assessment

Two independent NESSIE reviewers extracted data using a piloted form and conducted risk of bias (RoB) assessment adapted from the RoB 1 tool.^49^ Five domains for risk of bias were assessed: the randomisation process, allocation concealment, blinding of outcome assessment, missing outcome data and selection of results reported. Given the challenge of blinding participants or personnel when using exercise interventions, the RoB 1 performance bias domain was not assessed. For studies featured in the five Cochrane reviews, existing risk of bias assessments were used. Consensus checking was implemented by a third reviewer and disagreement resolved by discussion.

Data extracted included information on methods, population, interventions and outcomes. We extracted data at three time points (informed by Cochrane reviews) for critical outcomes: end of intervention, medium-term (6–30 months), and long term (>30 months).^20^^,^^25^, ^26^, ^27^, ^28^ For additional outcomes we extracted data at end of intervention only. When necessary, control groups were split between multiple intervention arms to prevent double counting.^50^^,^^51^ Where studies had been included in Cochrane reviews, data was extracted from both the review and original publication as needed.

Using details provided in study inclusion/exclusion criteria and demographic tables, we categorised included studies by vascular population: a) polyvascular (all participants had at least two of our index vascular conditions), b) mixed (proportion of participants had at least two of our index vascular conditions), c) single (participants had one index condition) or d) unclear (insufficient details to categorise). Aetiology was categorised as ischaemic or mixed. Studies which involved cases of heart failure and stroke from non-vascular causes were categorised as aetiology: mixed. Exercise intensity data was extracted as reported in the study or based on the metric used i.e., percentage of maximum heart rate and categorised as high (77–95%), low/light/moderate (57–76%) or unclear.^19^ To account for differences in exercise programmes, exercise dose was calculated by multiplying the number of weeks, by sessions per week, by duration in hours, informed by French et al.^52^ Exercise type was categorised as cardiorespiratory/aerobic, strength/resistance or mixed (both cardiorespiratory/aerobic and strength/resistance) based on information reported in the study or the type of exercise described.

### Outcomes

Critical outcomes of interest were (i) mortality (all cause and cardiovascular specific), (ii) the occurrence of vascular disease events (such as MI, stroke, amputation or acute limb ischaemia), (iii) hospitalisations for cardiovascular events, and (iv) generic health-related quality of life measures including: EuroQoL Group Quality of Life questionnaire (EQ-5D) and 36-item Short Form Survey (SF-36).^53^^,^^54^

Additional outcomes of interest included those measuring exercise tolerance: walking endurance (6-min walking test (6 MWT)),^55^ oxygen uptake capacity (VO2 max, VO2 peak), repetition maximum test, grip strength, maximum walking time (MWT) and maximum walking distance (MWD). We reported on whether studies detailed major adverse cardiovascular (MACE) or limb (MALE) events. In addition, we reported whether studies detailed healthcare resource use, costs or cost effectiveness.

### Data analysis and synthesis

All data analyses were conducted as prespecified in our protocol. We combined trials across all vascular conditions using random-effects meta-analyses (DerSimonian and Laird inverse-variance method) to reflect the clinical heterogeneity across trials in both their populations and exercise interventions. We categorised and pooled trials into two broad groupings: (1) trials of exercise only interventions vs no excise control and (2) trials of an exercise intervention plus an active co-intervention (e.g., education or psychological support) vs no exercise and the active co-intervention. We investigated the effect of exercise on our critical outcomes (all-cause mortality, cardiovascular-specific mortality, non-fatal MI, non-fatal stroke, hospitalisations for cardiovascular events, acute limb ischaemia, amputation, MALE, MACE, HRQoL: EQ-5D, HRQoL: SF-36) at three timepoints i.e., ‘end of intervention’ (immediately following completion of the exercise intervention), ‘medium term’ (6 months–30 months follow up), and ‘long term’ (>30 months follow up). For our additional outcomes we carried out meta-analysis at the end of intervention timepoint only. Continuous outcomes were expressed and pooled using either mean difference (where outcome measures were the same across trials) and binary outcomes using risk ratios. We reported follow-up scores when available. We pooled intention-to-treat estimates. We quantified statistical heterogeneity by visual inspection of forest plots and using the I^2^ statistic (50–90% may be substantial heterogeneity)^56^ When studies investigated more than one relevant exercise intervention arm, we reported both arms separately, splitting the control group participants between the arms if necessary.^50^ We explored subgroup analysis using stratified meta-analysis to investigate potential differences in exercise intervention effect due to the pre-defined subgroups of: (1) polyvascular/single populations, (2) index vascular condition, (3) ischaemic aetiology (ischaemic/mixed), (4) type of exercise (cardiorespiratory/aerobic or strength/resistance or mixed); (5) exercise intensity (high or low/light/moderate or unclear); and (6) total exercise dose (duration x frequency in hours: ≤20 h or > 20 h). Evidence of significant subgroup effects was assessed by using Test for subgroups differences from the stratified meta-analyses. Consistent with Cochrane methods guidance, we limited subgroup analyses to those outcomes with ≥10 trials. We focused on our interpretation of results based on 95% confidence intervals. We explored the impact of including studies at high risk of detection bias and attrition bias with sensitivity analysis. Review Manager (RevMan Web) was used for all meta-analyses.^57^ We used Grading for Recommendations Assessment, Development and Evaluation (GRADE) to assess the certainty of the evidence for our primary outcomes.^58^ The GRADE approach addresses the following factors that can reduce the quality of the evidence: study design (risk of bias), inconsistency of results, indirectness of evidence, imprecision and publication bias.

### Role of the funding source

The funder of the study had no role in study design, data collection, data analysis, data interpretation, or writing of the report. All authors had full access to the data in the study and had final responsibility for the decision to submit for publication.

## Results

**Fig. 1:**
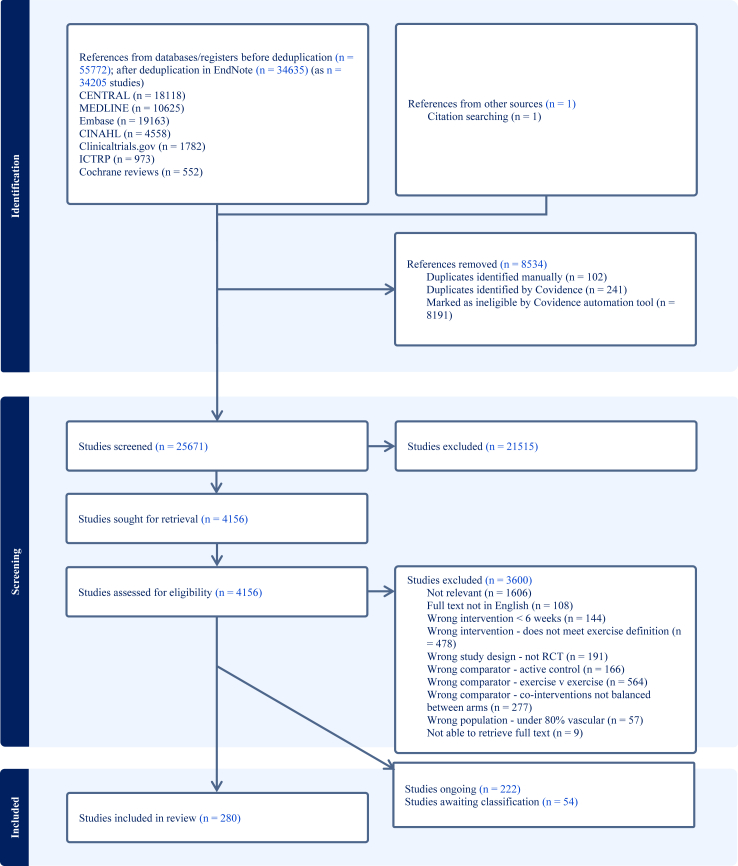
PRSIMA flow diagram.

The results of the search are shown in the PRISMA diagram (Fig. 1). The searches from February 2024 identified a total of 55,772 articles. After deduplication and removing references marked as ineligible by Covidence’ automation tool,^48^ we screened 25,671 articles by title and abstract and assessed 4156 full-text articles for eligibility. We excluded 3600 studies following the inclusion and exclusion criteria (Supplementary Table 1). We assessed 54 studies as awaiting classification (e.g., conference abstract only) and 222 studies as ongoing. We included a total of 280 studies.

Updated searches from January 2025 identified a further 7187 articles. After deduplication and removing references marked as ineligible by Covidence’ automation tool,^48^ we screened 3566 articles by title and abstract, with 569 articles assessed as potentially relevant. Following rapid review, 7/47 studies which met the inclusion criteria reported relevant outcomes. These have not been incorporated into the current analysis as our process of judgement indicates these will not impact the conclusions of our review.^59^ See Supplementary Table 2 for further details.

Risk of bias assessment for our included RCTs showed 23 (8·2%) studies classified as low risk in all five domains (Supplementary Figure 1). 257 (91·8%) studies were considered as being unclear or high-risk in one or more domains. We assessed 12 (4·3%) studies as being unclear or high-risk in all five domains. 61 (21·8%) studies were classified as being at high risk of either detection or attrition bias. As planned in our protocol, we investigated the impact of including these in our analyses by sensitivity analyses. No publication bias was detected by visual inspection of funnel plots.^56^

The total number of participants involved was 23,419, with study sizes ranging from 12 to 2331. See table of study characteristics (Supplementary Table 3). A total of 64·1% of participants were male. The mean age across studies varied from 31·87 (11·49) to 81·60 (3·70) years. Studies were carried out in 51 different countries, but ethnicity was very poorly reported (48/280 (17·1%)). We included participants diagnosed with existing vascular disease. We identified 96 studies which primarily included participants with heart failure, 88 which primarily included participants with coronary artery disease (including five where the index condition was angina), 48 included participants with stroke (including TIA), and 48 included participants with PAD.

Most studies did not report sufficient information to allow us to categorise as involving either polyvascular/mixed/or single condition populations and were assessed as being unclear (114/280; 40·7%). 94/280 (33·6%) studies were categorised as mixed (proportion of the participants had at least two of our index vascular conditions), and 65/280 (23·2%) as single. Only 7/280 (2·5%) studies included participants, all of whom had at least two of our index conditions and were assessed as polyvascular.

Exercise programmes varied by type, dose, duration and intensity of exercise as well as the exercise setting. See table of exercise characteristics (Supplementary Tables 4 and 5). Most studies (164/280; 58·6%) investigated cardiorespiratory/aerobic training; 20/280 (7·1%) investigated strength/resistance training, while 91/280 (32·5%) investigated mixed training and for 5/280 (1·8%) the type of exercise undertaken was unclear. For 200/280 (71·4%) studies the total dose of training was more than 20 h, and for 47/280 (16·8%) studies it was less than this. We were not able to determine dose for 33/280 (11·8%) studies. The median intervention duration was 12 weeks, varying from 6 to 520 weeks; most studies (170/280 (60·7%)) had a duration of 6–12 weeks, 77/280 (27·5%) had a duration of 3–6 months, 27/280 (9·6%) had a duration of more than 6 months (up to 12 months), and 4/280 (1·4%) had a duration of more than 1 year; duration was unclear for 2/280 studies (0·7%). Some studies described an intervention duration followed up by a period of self-exercise which was difficult to document. Intervention intensity varied; 100/280 (35·7%) studies reported a ‘high’ target intensity, 108/280 (38·6%) studies reported a ‘low/light or moderate’ intensity target intensity, whilst 48/280 (17·1%) studies did not report the target intensity. This information was unclear in 24/280 (8·6%) studies.

The no exercise (control) arm received usual care or attention control. As expected, this varied between studies depending on the index condition as usual care differs between conditions and from country to country. Twenty-three (8·2%) studies further described a co-intervention which was balanced in both the exercise and no exercise arms. Co-interventions included education on lifestyle (6/23); specific diet prescription, counselling or food supplements (7/23); acupuncture (1/23); relaxation (1/23); pharmaceuticals (simvastatin; dipyridamole; GM-CSF; testosterone; placebo; prindolol (6/23)); additional skill or cognitive training (2/23). Pharmaceuticals were included as co-interventions when they were not considered best medical therapy at the time of the study. As planned at protocol stage, these studies were analysed in a separate comparison.

Overall, 243/280 (86·8%) studies reported at least one of our critical or additional outcomes with 234 included in the meta-analyses. Nine studies did not report data in a useable form.

### Exercise vs no exercise

#### All-cause mortality

Overall, exercise may result in little to no difference in all-cause mortality between the exercise and no exercise groups at end of intervention (exercise: 302/6787 (4·5%); no exercise: 322/6024 (5·4%); risk ratio (RR) 0·92, 95% confidence interval (CI) 0·80–1·07; P = 0·30; 143 studies, 12,811 participants; low-certainty evidence); medium term (exercise: 339/5405 (6·3%) vs no exercise: 378/4878 (7·8%; RR 0·89, 95% CI 0·78–1·03; P = 0·11; 79 studies, 10,246 participants; low-certainty evidence); or long term (exercise: 413/2100 (19·7%) vs no exercise: 422/2062 (20·5%); RR 0·93, 95% CI 0·74–1·15; P = 0·49; 14 studies, 4162 participants). The median follow-up time in months, reported at medium (minimum to maximum) and long term timepoints were 9 months (6–30) and 49 months (30–120) respectively.

The test for subgroup differences indicates there is no statistically significant subgroup effect between polyvascular/mixed/single/unclear populations at end of intervention or medium timepoints (Test for subgroup differences P = 0·23; P = 0·59 respectively). At long term, the Test for subgroup differences did indicate a reduction in all-cause mortality for the single subgroup with exercise compared to no exercise (P = 0·01). Since the number of trials included in some subgroups is small, we do not have enough evidence to confidently conclude that this is a true subgroup effect. At medium term the Test for subgroup differences did indicate a reduction in all-cause mortality for ischaemic populations compared to mixed causes (P = 0·04). See Supplementary Table 6 and Summary of Findings Table 1 and 2.

The test for subgroup differences indicates there is no statistically significant subgroup effect of index condition (coronary artery disease, angina, heart failure, stroke (including TIA), and PAD), types of exercise (cardiorespiratory/aerobic alone or resistance/strength alone or mixed), total dose of exercise (≤20 h or > 20 h or unclear), or exercise intensity (high or low/light/moderate or not reported or unclear) at end of intervention, medium or long timepoints (Test for subgroup differences: P > 0·05).

#### Cardiovascular-specific mortality (including fatal MI and fatal stroke)

Overall, exercise may result in little to no difference in cardiovascular mortality between the exercise and no exercise groups at end of intervention (exercise: 164/3804 (4·3%) vs no exercise: 179/3515 (5·1%); RR 0·92, 95% CI 0·75–1·12; P = 0·41; 77 studies, 7319 participants; low-certainty evidence); or medium term (exercise: 188/3276 (5·7%) vs no exercise: 206/3108 (6·6%); RR 0·91, 95% CI 0·76–1·10; P = 0·35; 37 studies, 6384 participants; low-certainty evidence). At long term there may be a difference favouring exercise in cardiovascular mortality (exercise: 273/1976 (13·8%) vs no exercise: 318/1959 (16·2%); RR 0·81, 95% CI 0·64–1·01; P = 0·06; 10 studies, 3935 participants).

The test for subgroup differences indicates there is no statistically significant subgroup effect between polyvascular/mixed/single/unclear populations at end of intervention or medium term timepoints (Test for subgroup differences P = 0·61; P = 0·62 respectively). At long term, the Test for subgroup differences indicated a reduction for the single condition subgroup with exercise compared to no exercise (P = 0·04). Since the number of trials included in subgroups is small, we do not have enough evidence to confidently conclude that there is a true subgroup effect. See Supplementary Table 6.

The test for subgroup differences indicates there is no statistically significant subgroup effect for index condition, aetiology, types of exercise, total dose of exercise, or exercise intensity at end of intervention, medium or long timepoints (Test for subgroup differences: P > 0·05).

#### MI (non-fatal)

Overall, exercise may result in little to no difference in non-fatal MI between the exercise and no exercise groups at end of intervention (exercise: 69/2820 (2·5%) vs no exercise: 78/2491 (3·1%); RR 0·84, 95% CI 0·62–1·16; P = 0·29; 43 studies, 5311 participants; low-certainty evidence); medium term (exercise: 102/3165 (3·2%) vs no exercise: 100/2897 (3·5%); RR 0·92, 95% CI 0·70–1·20; P = 0·53; 34 studies, 6062 participants; low-certainty evidence); or long term (exercise: 76/1521 (5·0%) vs no exercise: 93/1502 (6·2%); RR 0·79, 95% CI 0·57–1·09; P = 0·15; 6 studies, 3023 participants).

The test for subgroup differences indicates there is no statistically significant subgroup effect for polyvascular/single populations, index condition, aetiology, type of exercise, total dose of exercise, or exercise intensity at end of intervention, medium or long timepoints (Test for subgroup differences: P > 0·05).

#### Stroke (non-fatal)

Overall, exercise may result in little to no difference in non-fatal stroke between the exercise and no exercise groups at end of intervention (exercise: 68/2896 (2·4%) vs no exercise: 62/2410 (2·6%); RR 1·03, 95% CI 0·73–1·45; P = 0·86; 43 studies, 5306 participants; low-certainty evidence); medium term (exercise: 74/2301 (3·2%) vs no exercise: 66/2004 (3·3%); RR 1·00, 95% CI 0·72–1·38; P = 1·00; 23 studies, 4305 participants; low-certainty evidence); or long term (exercise: 54/1274 (4·2%) vs no exercise: 54/1254 (4·3%); RR 1·01, 95% CI 0·70–1·46; P = 0·97; 3 studies, 2528 participants).

The test for subgroup differences indicates there is no statistically significant subgroup effect for polyvascular/single populations, index condition, aetiology, type of exercise, total dose of exercise, or exercise intensity at end of intervention or medium timepoints (Test for subgroup differences: P > 0·05).

#### Hospitalisations (vascular)

**Fig. 2:**
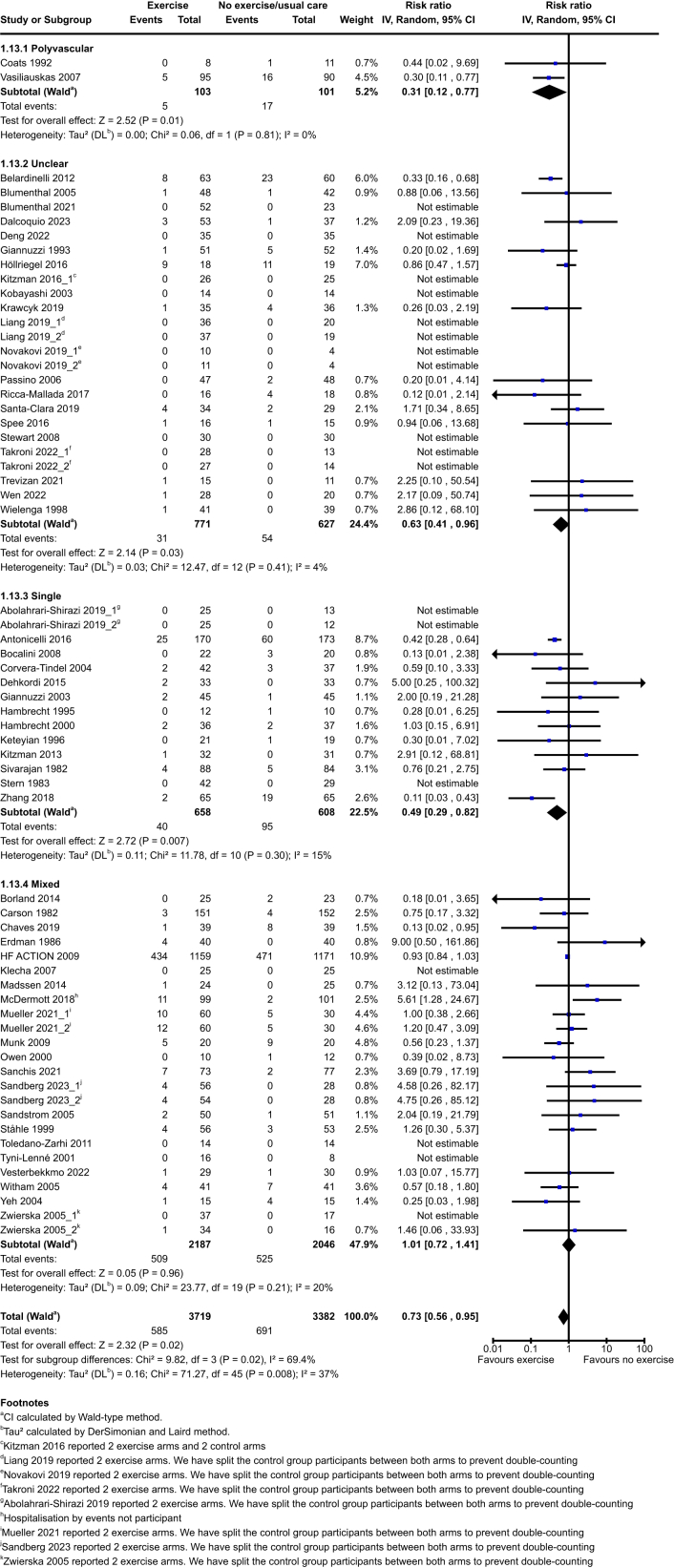
Vascular hospitalisations at end of intervention.

**Fig. 3:**
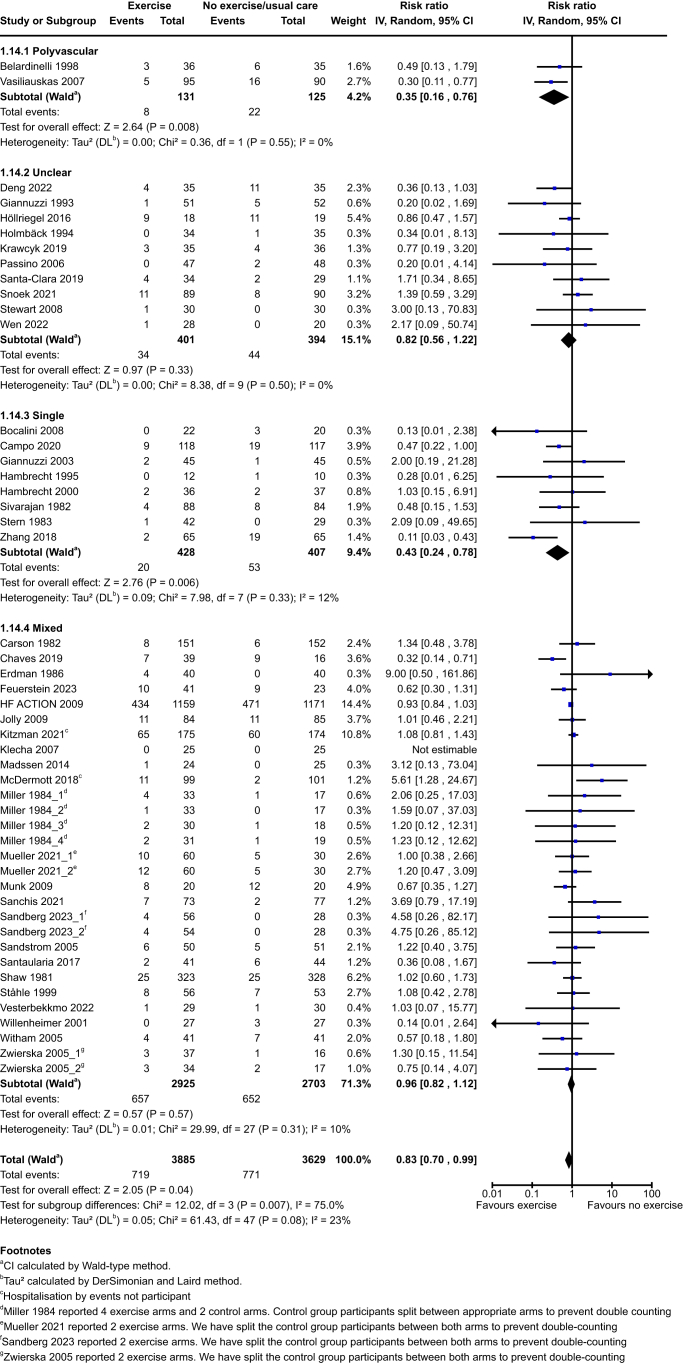
Vascular hospitalisations at medium term.

Exercise probably reduces vascular hospitalisations in the exercise group compared to the no exercise group at end of intervention (exercise: 585/3719 (15·7%) vs no exercise: 691/3382 (20·4%); RR 0·73, 95% CI 0·56–0·95; P = 0·02; 64 studies, 7101 participants; moderate-certainty evidence) and at medium term (exercise: 719/3885 (18·5%) vs no exercise: 771/3629 (21·3%); RR 0·83, 95% CI 0·70–0·99; P = 0·04; 49 studies, 7514 participants; low-certainty evidence). See Figs. 2 and 3. Exercise may result in little to no difference at long term (exercise: 486/1520 (32·0%) vs no exercise: 554/1499 (37·0%); RR 0·66, 95% CI 0·42–1·05; P = 0·08; 8 studies, 3019 participants).

The test for subgroup differences did indicate a statistically significant subgroup effect of polyvascular/single populations et end of intervention and medium term with differences in the size of benefit effect on hospitalisations between subgroups (Test for subgroup differences P = 0·02; P = 0·007 respectively). See Supplementary Table 6. We cannot conclude this is a true subgroup effect as high heterogeneity was detected (I^2^ > 69·4%).

The test for subgroup differences did indicate a statistically significant subgroup effect of index condition at end of intervention with differences in both size and direction of effect on hospitalisations between subgroups (Test for subgroup differences P = 0·009). Since the number of trials included in some subgroups is small, we do not have enough evidence to confidently conclude that there is a true subgroup effect. See Supplementary Table 6. The test for subgroup differences indicates there is no statistically significant subgroup effect at medium or long term (Test for subgroup differences P = 0·09; P = 0·63 respectively).

The test for subgroup differences indicates there is no statistically significant subgroup effect of aetiology, type of exercise, total dose of exercise, or exercise intensity at end of intervention or medium term (Test for subgroup differences P > 0·05).

#### Amputation

The evidence is very uncertain about the effect of exercise on amputation at end of intervention (exercise: 0/135 (0·0%) vs no exercise: 2/136 (1·5%); RR 0·20, 95% CI 0·01–4·15; P = 0·30; 3 studies, 271 participants; very low-certainty evidence); and medium term (exercise: 0/135 (0·0%) vs no exercise: 2/136 (1·5%); RR 0·20, 95% CI 0·01–4·15; P = 0·30; 3 studies, 271 participants; very low-certainty evidence). No studies reported amputation at long term.

#### Acute limb ischaemia

The evidence is very uncertain about the effect of exercise on ALI at end of intervention (exercise: 6/364 (1·7%) vs no exercise: 1/191 (0·5%); RR 1·12, 95% CI 0·20–6·45; P = 0·90; 4 studies, 555 participants; very low-certainty evidence); and medium term (exercise: 6/364 (1·7%) vs no exercise: 1/191 (0·5%); RR 1·12, 95% CI 0·20–6·45; P = 0·90; 4 studies, 555 participants; very low-certainty evidence). No studies reported ALI at long term.

#### Health-related QoL

Exercise probably increases HRQoL: EQ-5D compared to no exercise at end of intervention (mean difference (MD) 6·20, 95% CI 2·21–10·20; P = 0·002; 8 studies, 805 participants; moderate-certainty evidence). Exercise may result in little or no difference at medium term (MD 2·23, 95% CI –3·19 to 7·66; P = 0·42; 7 studies, 707 participants; moderate-certainty evidence) and long term (MD 6·00, 95% CI –2·05 to 14·05; P = 0·14; 1 study, 73 participants).

**Fig. 4:**
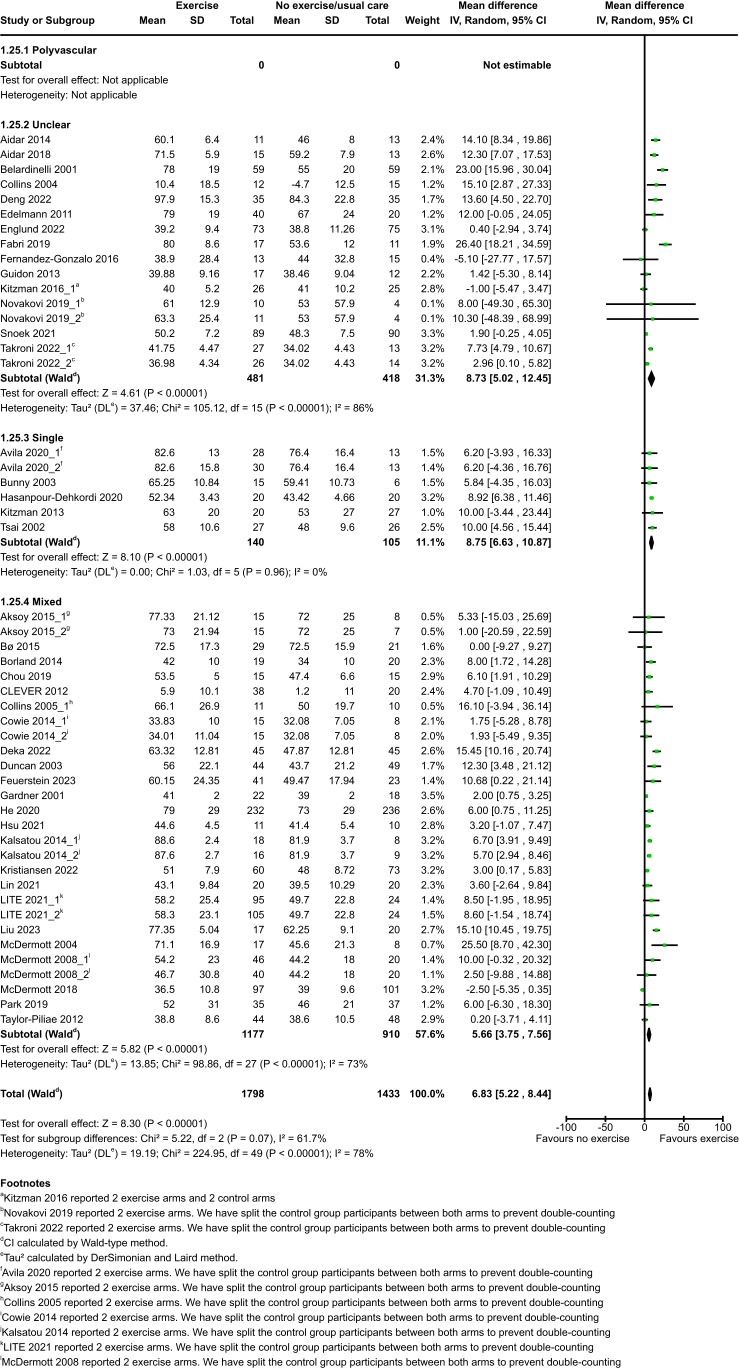
HRQoL: SF-36 at end of intervention.

**Fig. 5:**
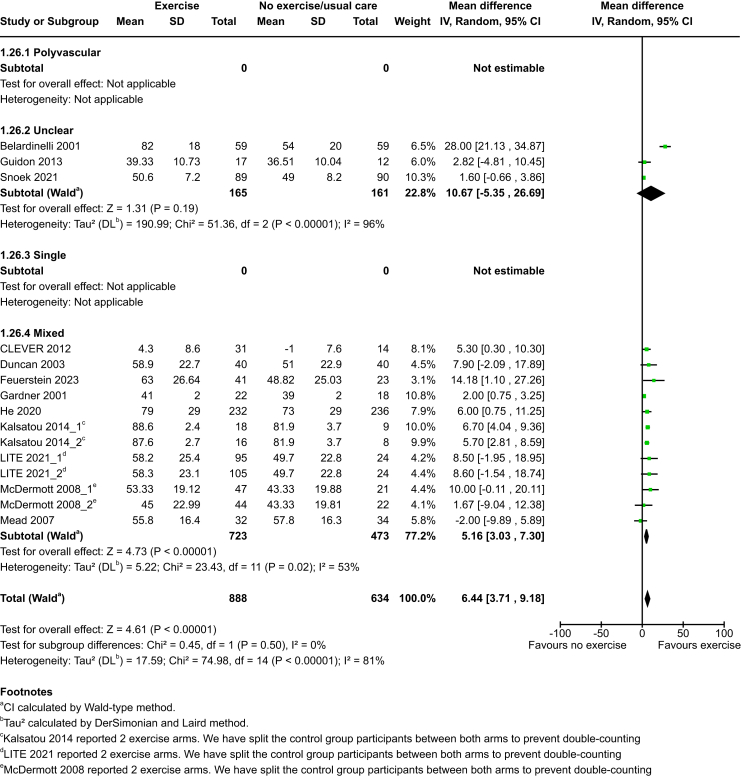
HRQoL: SF-36 at medium term.

Exercise probably increases HRQoL: SF-36 compared to no exercise at end of intervention (MD 6·83, 95% CI 5·22–8·44; P < 0·0001; 50 studies, 3231 participants; moderate-certainty evidence) and medium term (MD 6·44, 95% CI 3·71–9·18; P < 0·0001; 15 studies, 1522 participants; moderate-certainty evidence). No studies reported HRQoL: SF-36 at long term. See Figs. 4 and 5.

The test for subgroup differences indicates there is no statistically significant subgroup effect of polyvascular/single populations, aetiology, total dose of exercise, or intensity (Test for subgroup differences P > 0·05).

The test for subgroup differences did indicate a statistically significant subgroup effect in the size of benefit due to index condition and type of exercise at end of intervention (Test for subgroup differences P = 0·03; P = 0·0002 respectively). See Supplementary Table 6. We cannot conclude this is a true subgroup effect as high heterogeneity was detected (I^2^ = 65·8%, I^2^ = 85·1% respectively).

#### Additional outcomes

Exercise significantly reduced adverse events reported as MACE at end of intervention (exercise: 5/161 (3·1%) vs no exercise: 16/118 (13·56%); RR 0·27, 95% CI 0·10–0·72; P = 0·008; 2 studies, 279 participants) and at medium term (exercise: 16/191 (8·4%) vs no exercise: 36/177 (20·3%); RR 0·42, 95% CI 0·22–0·82; P = 0·01; 2 studies, 368 participants), but no difference was detected at long term (exercise: 13/52 (25·0%) vs no exercise: 6/23 (26·1%); RR 0·96, 95% CI 0·42–2·21; P = 0·92; 1 study, 75 participants).

Exercise may result in little to no difference in adverse events reported as MALE at end of intervention (exercise: 5/16 (31·3%) vs no exercise: 7/21 (33·3%); RR 0·94, 95% CI 0·36–2·41; P = 0·89; 1 study, 37 participants) or at medium term (exercise: 5/16 (31·3%) vs no exercise: 7/21 (33·3%); RR 0·94, 95% CI 0·36–2·41; P = 0·89; 1 study, 37 participants). No studies reported MALE at long term.

In terms of physical fitness, exercise significantly improved exercise tolerance at end of intervention as measured by 6 MWT (s): (MD 48·78, 95% CI 40·08–57·48; P < 0·0001; 78 studies, 4904 participants); VO2 max (ml/min/kg): MD 3·21, 95% CI 2·12–4·29; P < 0·0001; 11 studies, 488 participants); VO2 peak (ml/min/kg): MD 2·81, 95% CI 2·42–3·21; P < 0·0001; 97 studies, 4503 participants); repetition maximum test (1-RM): MD 2·92, 95% CI 2·42–3·43; P < 0·0001; 8 studies, 337 participants; and grip strength (kg): MD 1·98, 95% CI 0·47–3·49; P = 0·01; 11 studies, 745 participants).

Due to considerable levels of heterogeneity, we did not undertake meta-analyses for MWD or MWT.

#### Exercise plus co-intervention vs no exercise plus co-intervention

Evidence was limited for this comparison as only 8/23 (34·8%) studies contributed data to any outcome. Results have been reported fully in Supplementary information. Briefly, no differences were detected in all-cause mortality, cardiovascular-specific mortality, MI, stroke, hospitalisations or HRQoL: SF-36 between the exercise plus co-intervention and no exercise plus co-intervention groups. Exercise improved measures of fitness (6 MWT, VO2 peak). No studies reported on HRQoL: EQ-5D, amputation, ALI, MACE, MALE, MWD, VO2 max, or the Repetition maximum test.

#### Costs

We planned to report when studies carried out healthcare resource use, costs or cost effectiveness to facilitate future economic evaluations without commenting on the findings of these. Eight studies reported relevant information, and an additional three studies stated they planned to report. See Supplementary Table 7 for further details.

## Discussion

This systematic review is the first to comprehensively investigate the effects of exercise on the secondary prevention of major adverse cardiovascular events and HRQoL in people with one or more vascular condition. We also explored any differential effects of exercise in specific populations, as well as any differential effects of exercise parameters.

The main findings indicate that exercise may have little or no effect on all-cause mortality or cardiovascular mortality at end of intervention or medium term, but cardiovascular-specific deaths may be reduced by long term. The findings summarised above were irrespective of participants’ vascular diagnoses or exercise intervention parameters. Although this low-certainty evidence requires to be strengthened, it suggests that exercise interventions—delivered according to the type and dosage (including type, total number of hours, intensity) described in the included studies—may not be sufficient to affect secondary prevention. However, given that the duration of most interventions was no longer than 12 weeks, this is perhaps not surprising. Furthermore, there was a general paucity of follow-up data beyond 30 months, which made it difficult to draw firm conclusions about the longer-term impact of exercise. Importantly, this review also found moderate-certainty evidence that exercise reduced vascular hospitalisations, both at the end of the intervention and in the medium term. This effect dissipated in the longer term. This was irrespective of the aetiology of the vascular condition, or the exercise intervention parameters.

This review also found moderate-certainty evidence that exercise improved HRQoL at the end of the intervention when measured with either EQ-5D or SF-36 questionnaires. This is an important finding as it has been established that cardiovascular disease is associated with impaired HRQoL and HRQoL is a predictor of long-term mortality in patients with coronary heart disease.^60^^,^^61^ HR-QoL is often overlooked by researchers, while being a priority to patients.^62^ Improved HRQoL reported with EQ-5D had dissipated at medium and long-term follow-up. In contrast, benefits reported with the SF-36 were sustained at medium term (there were no long-term data). These findings were independent of whether participants had one or more vascular condition, the aetiology of the condition, or exercise dose or intensity.

Evidence was limited and therefore uncertain for important clinical endpoints of amputation and ALI. Similarly, composite cardiovascular endpoints MACE and MALE were poorly reported by studies, reducing our ability to investigate an effect of exercise or differential effects of exercise in specific populations or exercise parameters in pre-planned subgroup analysis.

This review found that exercise also resulted in a range of fitness benefits at the end of the intervention, i.e., improvements in walking endurance, maximum oxygen uptake capacity, repetition maximum and grip strength. The mean difference in walking endurance following exercise exceeds the Minimal Clinically Important Difference (MCID) for people with stroke and may therefore be considered a meaningful improvement.^63^^,^^64^ The change in grip strength however did not reach the MCID for people with stroke.^65^

Thus, overall the evidence included in this review indicates that exercise may not affect mortality but probably reduces vascular hospitalisation whilst improving health-related quality of life and a range of physical fitness parameters.

These findings align with other systematic reviews on exercise and fitness training after stroke. Saunders et al., reported low-certainty evidence that fitness training did not impact on mortality, whilst finding low-moderate certainty evidence for multiple, training-specific improvements in fitness (including oxygen uptake capacity, strength, walking speed and balance) in a population that was mostly able to walk independently.^25^ Similarly, our findings are consistent with those reported by Lloyd et al., who focused on fitness training for stroke survivors who were unable to walk independently.^66^ Their review found no difference in mortality between intervention and control groups and few adverse events, whilst finding a range of improvements in fitness outcomes as a result of assisted walking and cycling. English et al., in their review of circuit training after stroke, did not report findings on mortality but did find moderate-certainty evidence for training-specific, clinically meaningful benefits (including walking capacity and speed) as a result of the training.^67^ Across these reviews, evidence for adverse events was uncertain, due to generally poor reporting.

The strengths of this systematic review include comprehensive searches, inclusion of studies which meet the ACSM definition of exercise, combining vascular conditions, assessing the quality of the included studies, using GRADE to assess the certainty of our findings, and adhering to our prospectively registered protocol. We were limited by issues with poor descriptions of exercise by studies, and by poor, inconsistently reported study inclusion and exclusion criteria, especially limiting our ability to categorise included populations as polyvascular/single. Although we included studies undertaken in over 50 countries worldwide, ethnicity was very poorly reported. Many studies did not report clinical outcomes, and these were rarely reported at longer follow-up times. Our median long-term follow-up was 49 months (30–120 months), with only 14 studies providing data at this point. It is possible that differences in all-cause and cardiovascular mortality may be detected at later time-points. Data were often extracted from participant flow diagrams and descriptions of losses to follow-up and exclusions, rather than as prespecified reported outcomes. Despite using accepted, validated scales for continuous outcomes, some continuous data might include data with skewed distributions. We explored the impact of potentially skewed distributions by sensitivity analysis and no changes to overall effects were seen (Supplementary Figures 2 and 3).

Further limitations arise from inclusion of studies with small-sample sizes and that clinical outcomes were rare, which may result in small-sample bias and sparse-data bias. We reported fitness parameters at end of intervention only and so could not determine if benefits were maintained over the long term. We reported studies investigating exercise plus co-intervention compared to no exercise plus co-intervention separately as planned in our protocol. Retrospectively, including these in our main analysis may strengthen the evidence. We will consider this, and undertaking meta-regression, in the future. Adherence to the exercise programmes were poorly measured, so it is not possible to account for the exercise actually undertaken by the exercise groups. This may impact the effect seen. As planned in our protocol, we only extracted HRQoL data when studies reported using either the EQ-5D or SF-36 questionnaires. Many studies measured HRQoL using condition specific questionnaires which we were unable to investigate due to the number of additional analyses required. Risk of bias assessment demonstrated that methodological quality varied across studies and only 8·2% of studies were classified as low risk of bias in all five domains. Detection bias was a common issue; given the nature of the intervention, participants and intervention providers cannot be blinded and it is crucial to ensure that outcome assessors are blinded. However, this was only clearly reported in 109/280 (38·9%) of studies. Ensuring and clearly reporting blinded outcome assessment is a key recommendation for improving the quality of future trials. We assessed many studies as awaiting classification or ongoing. The results may change once this evidence is available to include.

Traditionally, clinical trials on exercise have largely been undertaken in condition-specific ‘silos’, where eligibility criteria are set to include populations with a single index condition, whilst excluding participants with additional co-morbidities—especially those considered to pose a risk. Although the evidence requires strengthening, findings from this review, regarding vascular conditions, call this condition-specific approach to clinical trials into question. Given demographic changes across the globe, which mean that a growing proportion of the world population will have more than one vascular condition, high-quality clinical trials are needed that are representative of the actual clinical population.^68^ Therefore, more rigorous trials with appropriate risk mitigation procedures in place, are needed that include participants with polyvascular conditions in order to strengthen the evidence on safety of this intervention and to inform clinical guidelines. Further recommendations may be proposed to advance the quality of research in this important area. It was difficult to establish the number of vascular conditions in 41% of the studies included. This lack of clarity affects the generalisability of the findings of this review to populations with polyvascular conditions. Therefore, the number and type of vascular conditions needs to be reported more comprehensively in future trials.^37^^,^^69^ Additionally, reporting of ethnicity needs to be expanded. The reporting of exercise intervention parameters was lacking in detail, especially in relation to intensity. Future studies should endeavour to follow the CERT guidelines to enhance transparency and replicability of the interventions.^70^ Most of the exercise programmes were between 6 and 12 weeks' duration. Given the need to establish long term exercise routines, future trials should endeavour to provide longer exercise intervention periods, coupled with self-management programmes. It will be important to measure physical activity during these self-management activities, as this is a potential confounding factor, which was poorly reported in the studies included. Similarly, other lifestyle factors such as diet and medication adherence are also a crucial part of long-term self-management and potential confounders. Additionally, studies with follow-up periods beyond 30 months are recommended in order to strengthen the evidence about secondary prevention.^71^^,^^72^ Likewise, qualitative studies and long-term evaluations of HRQoL are needed to further understand the underlying mechanisms of how exercise interventions can contribute to improve quality of life in cardiovascular disease patients.^62^

Similar to clinical trials, many physical activity referral pathways follow ‘silos’, i.e., they are designed for people with a single index condition (e.g., cardiac rehabilitation), often to the exclusion of those with co-morbidities (e.g., people with stroke). However, findings from this review indicate that exercise is unlikely to cause major adverse cardiac or limb events in people with a wide range of vascular conditions. Thus, these findings also question the condition-specific nature of some physical activity referral pathways. Moreover, given that many people affected by vascular conditions are concerned that exercise may trigger a major adverse event, our findings may offer some reassurance.^73^ Together with the benefits of exercise on HRQoL and a range of fitness parameters, findings on risk could be highlighted by practitioners in order to encourage people to engage with/maintain their physical activity, as suggested by World Health Organisation's global guidelines and the World Stroke Organisation guidelines, European Society of Cardiology management guidance for chronic coronary syndromes and HF and peripheral arterial disease.^7^^,^^13^^,^^38^^,^^39^^,^^74^ Whilst strengthening the evidence for exercise in people with a range and combination of vascular conditions, it is imperative that the available evidence be implemented, to avoid deconditioning associated with a lack of physical activity, and optimise the benefits of exercise, along the entire pathway of people with one or more vascular conditions.

## Contributors

Conceptualization: GEM, RT, CB, ATB, LM, FvW, CK, SC, GS, MS; Methodology: GEM, CB, ATB, LM, FvW, RT, CK, SC, GS, MS, CF; Software: CF, CS, CB, WX; Validation: N/A; Formal analysis: CB, CS, WX, MS; Investigation: CB, CSJ, MS, KC, KT, CS, WX, JC, ATB, LR, MN, VV, PK, JP, CF, GEM, LM; Resources: N/A; Data Curation: CB, CS, MS, WX; Writing–Original Draft: GEM, CB, KT, JC, ATB, CF, MS; Writing–Review & Editing: All authors; Visualization: CB, CS, KT, WX; Supervision: GEM, CB, MS; Project administration: CB, MS, GEM; Funding acquisition: N/A.

## Data sharing statement

All data requests should be submitted to the corresponding author for consideration. Access to all anonymised data may be granted following review by the NESSIE investigators and completion of a data sharing agreement.

## Declaration of interests

Cathryn Broderick: none known. This project was funded by the NIHR Evidence Synthesis Programme (programme grant: NIHR153425, project number NIHR162044). Payment to institution.

Marlene Stewart: none known. NIHR Evidence Synthesis Programme (programme grant: NIHR153425, project number NIHR162044). Payment to institution.

Katie Thomson: none known.

Ceri Sellers: none known.

Candida Fenton: none known.

Julie Cowie: none known.

Wei Xu: none known.

Christa St Jean: none known. NIHR Evidence Synthesis Programme (programme grant: NIHR153425, project number NIHR162044). Payment to institution.

Keira Charteris: none known.

Madhurima Nundy: none known.

Vaishali Vardhan: none known.

Prerna Krishan: none known.

Jolie Pistol: volunteer member of UNCOVER.

Leonor Rodríguez: none known.

Alex Todhunter-Brown: NIHR Evidence Synthesis Programme funds NESSIE (NIHR Evidence Synthesis Scotland Initiative). I am co–lead investigator on NESSIE. Funding to institutions for NESSIE funds researcher time to conduct the work presented in this manuscript. Co–Lead Cochrane Heart, Stroke & Circulation Thematic Group. This group supports the planning, conduct and dissemination of Cochrane systematic reviews. This is an unpaid role, with no financial involvement in any Cochrane activities or reviews.

Frederike van Wijck: receives royalties for publication of Mead G & van Wijck F (Eds). Exercise and fitness training after stroke: a handbook for evidence-based practice (2013).

Sheila Cameron: none known.

Catriona Keerie: none known.

Rod S Taylor: none known.

Gerry Stansby: none known.

Gillian Mead: Around £20 a year for a book published by Elsevier about exercise training after stroke.
