# Supplementary material for: The effects of exercise on secondary prevention and health-related quality of life in people with existing vascular disease: systematic review and meta-analysis of randomised controlled trials
Source: eClinicalMedicine. 2025 May 9;83:103201. doi: 10.1016/j.eclinm.2025.103201 (PMC12235401; doi:10.1016/j.eclinm.2025.103201)
Supplement: Supplementary Figures and Tables [file mmc1.docx]

**The effects of exercise on secondary prevention and health-related quality of life in people with existing vascular disease: systematic review and meta-analysis of randomised controlled trials**

**Supplementary material**

**Contents**

[S1: Protocol: The effects of exercise on secondary prevention and quality of life in people with existing vascular disease: systematic review and meta-analysis of randomised controlled trials 2](#_Toc195533138)

[S2: Search strategy 19](#_Toc195533139)

[S3: Supplementary Table 1. Table of excluded studies with reasons 28](#_Toc195533140)

[S4: Supplementary Table 2: Table detailing records identified by updated searches (January 2025) 435](#_Toc195533141)

[S5: Included study references by study 443](#_Toc195533142)

[S6: Supplementary Figure 1: Risk of Bias 476](#_Toc195533143)

[S7: Supplementary Table 3: Table of study characteristics - participants 477](#_Toc195533144)

[S8: Supplementary Table 4: Table of study characteristics - exercise programme 488](#_Toc195533145)

[S9: Supplementary Table 5: Table showing types of exercise 496](#_Toc195533146)

[S10: Supplementary Table 6: Subgroup analysis 497](#_Toc195533147)

[S11: Summary of Findings Table 1 499](#_Toc195533148)

[S12: Summary of Findings Table 2 501](#_Toc195533149)

[S13: Exercise plus co-intervention versus no exercise plus co-intervention   503](#_Toc195533150)

[S14: Supplementary Table 7: Studies reporting cost effectiveness analysis 505](#_Toc195533151)

[S15: Patient and public involvement (PPI) 506](#_Toc195533152)

[S16: Supplementary Figures 2 and 3: Sensitivity analysis to investigate impact of skewed data 506](#_Toc195533153)

# S1: Protocol**:** The effects of exercise on secondary prevention and quality of life in people with existing vascular disease: systematic review and meta-analysis of randomised controlled trials

**Registered authors**

Cathryn Broderick (joint first)

Rod S Taylor (joint first)

Alex Todhunter-Brown

Catriona Keerie

Frederike van Wijck

Luciana Mendes

Sheila Cameron

Candida Fenton

Marlene Stewart

Gerry Stansby

Gillian Mead FMedSci

**Funding**

This study is funded by the NIHR Evidence Synthesis Programme.

**Background**

There are around 7.6 million people living with heart and vascular diseases in the UK. This figure includes conditions such as coronary heart disease, atrial fibrillation, heart failure, stroke, amputation and vascular dementia.^1^ Together they cause around a quarter of all deaths in the UK (> 160,000 deaths each year, of which around 48,000 are people under the age of 75).^1^

Cardiovascular diseases (CVDs) are a group of disorders which include coronary heart disease, cerebrovascular disease, and peripheral arterial disease. In 2017, ≈17.8 million deaths were attributed to CVD globally, making them the leading cause of death.^2, 3^ The impact of stroke and coronary heart disease on population health is considerable, with a recent report calculating they account for an estimated 70 million disability-adjusted life years (DALYs) across European Society of Cardiology (ESC) member countries in 2019.^4^ The same report in the ESC Atlas Project highlights that despite some risk factors being reversible, there are persisting inequalities in disease burden by sex and national income status.^4^

A significant factor to each of these conditions is a build-up of fatty deposits on the inner walls of the arteries that supply the heart, brain and limbs which reduces the blood and oxygen supply to these areas (ischaemia).^2^ This is known as atherosclerosis and can lead to ischaemic stroke, cardiac ischemia, cardiac failure, myocardial infarction (MI), peripheral arterial disease (PAD) and amputation. ^5, 6^

Atherosclerosis is considered a systemic process mediated through inflammation, cholesterol deposition, and thrombosis.^7^ Subclinical cellular changes in arteries over time result in the subendothelial accumulation of low-density lipoprotein (LDL)-derived cholesterol. This area of impaired endothelial function (known as plaques), promotes recruitment of immune cells leading to a pro-inflammatory, disease-propagating environment.^8^ Progressive development of atherosclerotic plaques results in narrowing of the vessel lumen and plaque erosion/rupture causing atherothrombosis with total luminal occlusion. This may result in life-threatening cardiovascular events such as MI, stroke or limb loss.^9-13^

The presence of atherosclerosis in two or more arterial beds is termed polyvascular disease.^3^ Large scale registry studies have enabled the evaluation of polyvascular disease across populations, and have indicated an increased risk of major adverse cardiovascular events (cardiovascular death, MI, and ischaemic stroke) and major adverse limb events (defined as severe limb ischaemia leading to an intervention or major vascular amputation) in this group of patients compared to those with disease in a single territory only.^14, 15^

Additionally, cerebrovascular small vessel disease (SVD) causes about 25% of ischaemic strokes and is a major contributor to vascular dementia.^16^ SVD is an umbrella term of disease processes affecting the small arteries, arterioles, venules, and capillaries of the brain.^17^

Following diagnosis of coronary heart disease, cerebrovascular disease or peripheral arterial disease, disease-specific guidelines recommend physical activity including exercise as part of long-term management and rehabilitation for these conditions.^18-27^ This is intended to improve the physical functioning of people living in each of these specific cohorts and to reduce the risk of further adverse vascular events (secondary prevention).

**Description of the intervention**

Exercise is defined as ‘‘…a type of Physical Activity consisting of planned, structured, and repetitive bodily movement done to improve and/ or maintain one or more components of physical fitness.’’ ^28-30^ Some researchers may use the term ‘physical fitness training’ as meaning the same as ‘exercise’. While many programmes aim to increase physical activity, these may not meet the duration, intensity or frequency necessary to meet the definition of exercise as reported above. It is often necessary to individualise programmes to meet the specific conditions and capabilities of individual patients. Exercise training can include aerobic or strength aspects. Some exercise programmes may include exercise training alone or in combination with psychological or educational interventions.^31^

Exercise has a protective effect on CVD risk through the modulation of multiple risk factors including lipid metabolism, vascular biology, inflammatory and stress response, all of which can impact the process of atherosclerosis.^9, 32, 33^ The associated collective benefits of regular exercise relevant to cardiovascular conditions include lower blood pressure, improved lipoprotein profile, weight management, reduced mental stress and improved quality of life.^6, 29, 33^ Research investigating the biological mechanisms involved indicates that benefits likely result from improved endothelium-dependent vasodilation, reduced levels of inflammatory cells which may dampen the pro-inflammatory environment and improved aerobic capacity.^9, 34-37^ In patients with cerebral SVD, exercise may not reduce the progression of disease as seen on brain magnetic resonance imaging, but it does reduce the risk of all-cause mortality and cerebrovascular events.^38^

**Why it is important to do this review?**

This systematic review and meta-analysis aims to review the available evidence about the effectiveness of exercise undertaken as part of rehabilitation or management programs in preventing further/secondary adverse vascular events in people with one or more arterial vascular diseases, including ischaemic heart conditions, stroke, and people with peripheral arterial disease. Current relevant Cochrane reviews of exercise (also called ‘physical fitness training’) focus on the effects of interventions on populations with an index event of stroke, angina, coronary artery disease, heart failure or peripheral arterial disease.^31, 39-42^ We know from these reviews and other non-Cochrane reviews that the included trials may have excluded people with other, or multiple vascular diseases, meaning that the evidence base is generally only applicable to patients with a single index event.^43-49^ Similarly, there is a lack of systematic reviews investigating the effectiveness of exercise for secondary prevention in populations with polyvascular disease.^50^ As polyvascular disease becomes more prevalent, it is important to understand the effect of exercise in people with more than one vascular disease, as well as in people with one index condition. This systematic review presents an opportunity to determine the extent to which polyvascular populations have been included in exercise intervention studies which focused on a single vascular condition. There are no, to our knowledge, systematic reviews of exercise in people with polyvascular disease as the index condition, nor a Cochrane overview of exercise for a range of vascular conditions to date. This partly relates to the previous existence of disease-specific Cochrane groups, which meant that reviews in heart, stroke and peripheral vascular disease were done independently, without any sharing of disease specific expertise. Also, the definitions of exercise differed across the disease specific reviews: the Cochrane Stroke review focused on ‘exercise’ whilst the cardiac rehabilitation reviews had a broader focus of ‘exercise-based interventions’. This leads to difficulty in understanding the relative effects of exercise in different vascular conditions, what components of complex interventions are most important for rehabilitation, management and secondary prevention of future cardiovascular events in people with a range of different index vascular events, and which intervention components would be safe and effective for people with more than one arterial vascular disease - which is increasingly common. It is important to understand to what extent people with more than one vascular disease were included or excluded from trials. This will help clinicians determine the applicability of trial data to the ‘typical’ patient who may well have more than one vascular disease and the impact of more than one vascular diagnosis on the treatment impact of exercise.

We also wish to determine if there are differences between types of exercise programmes, and whether the addition of education/psychological interventions influences the effect of exercise. It is possible that different types of exercise are more effective for different types of vascular disease. This will help inform clinical guidelines, policy and practice in relation to implementation of exercise services for populations similar to those included in this review. Currently these services may be suboptimal as they either provide for people with singular diseases only or provide for people with multi-morbidity - but without adequate knowledge of optimal exercise parameters, which could affect outcomes. Given the epidemiology of cardiovascular and cerebrovascular disease, services could potentially be optimised, in terms of clinical effects and cost-effectiveness, if larger groups of people with a range of vascular disease could take part in evidence-based exercise interventions. We know that currently most patients after stroke do not receive exercise programmes either in hospital or in the community, and many patients with coronary heart disease also do not participate in exercise even though these are generally part of the pathway for the management of these patients.^51^ Thus, a high-quality review and meta-analysis could provide the necessary impetus for clinicians and commissioners to work more closely to ensure that patients with vascular diseases (as defined above) will receive optimum care. Our review has major potential to influence clinical practice.

**Objectives**

We aim to determine the effect of exercise in the secondary prevention of major adverse cardiovascular events (MACE) or major adverse limb events (MALE) and health related quality of life (HRQoL) for people with existing vascular disease as the index condition leading to recruitment into the trial (at least one or more of coronary artery disease (including angina), heart failure, stroke (including transient ischaemic attack (TIA)), and peripheral arterial disease (PAD)). We have carefully considered our primary and secondary outcomes. Clinicians have a strong focus on minimising the risk of future vascular events, and quality of life is of major importance to patients and their families.^52-54^

We aim to investigate additional condition focused objectives by subgroup analysis:

1. What is the effect of exercise in people with a single vascular disease and what is the effect in people with polyvascular disease?
2. What is the effect of exercise in different vascular conditions (coronary artery disease (including angina), heart failure, stroke (including TIA), and PAD)?

We aim to investigate additional exercise focused objectives by subgroup analysis:

1. What is the effect of exercise in people with a single vascular disease and what is the effect in people with polyvascular disease?
2. What is the effect of different types of exercise on our pre-defined outcomes? We will categorise the types of exercise as strength (often the term resistance or power training is used interchangeably) or aerobic (also called cardiorespiratory training) or mixed training (both aerobic and strength).
3. What influence does the intensity, frequency and duration of exercise session have on outcomes? We will categorise these using descriptions of intensity (high/low/unclear). Low intensity may also be known as light or moderate. We will categorise number of exercise sessions in programme and duration of individual exercise sessions as total dose. See subgroup analysis section for more detail.

**Study design**

We will register this protocol on PROSPERO, the International Prospective Register of Systematic Reviews. We plan to use a two-way approach to undertake this systematic review and meta-analysis. First, we will identify relevant randomised controlled trials (RCTs) from five published key Cochrane reviews as the high-quality comprehensive searches will have identified relevant trials.^31, 39-42^ We plan to use data already extracted from the original RCTs to reduce duplication of effort and speed up the review process. Secondly, we will undertake new searches to identify RCTs published after the search dates of the five Cochrane reviews as well as for conditions not covered by the original searches i.e., TIA.

We will design one inclusive search based on the five key Cochrane reviews which include our populations of interest (see below) to identify all relevant studies published since 2016 (the search date of oldest relevant Cochrane review).^31, 39-42^ We will cross-check the results of this with the RCTs from the five published key Cochrane reviews. ^31, 39-42^ In addition, we will design a separate search to identify studies involving the TIA population which was not within the scope of the original Cochrane reviews. This will be over a longer period of time (from 1990 onwards).

We will utilise the existing key Cochrane reviews to obtain 1) characteristics of the relevant included studies and 2) outcome data. We will extract data on study characteristics and relevant outcome measurements for eligible studies reported in the published Cochrane reviews (sourced from open access RevMan files). When additional data is required from studies included in the key Cochrane reviews but not reported in the reviews, we will extract this *de novo* from original study publications. Data required from trials that are not included in the key Cochrane reviews will be extracted *de novo* from original study publications; this will include characteristics of studies and outcome data.

The methods for conducting and reporting this review will follow the Cochrane Handbook for Systematic Reviews of Interventions, and PRISMA reporting guidelines.^55, 56^

**Key terms used throughout this protocol**

We use the following definitions throughout this document:

Exercise is defined by the ACSM as “Exercise is a type of physical activity consisting of planned, structured, and repetitive bodily movement done to improve and/or maintain one or more components of physical fitness”.^30^ For this review, an intervention described as exercise-based or exercise as one part of a therapy programme will meet our definition of exercise providing it involves strength (also called resistance or power training) or aerobic (also called cardiorespiratory training) or mixed training. This is important because these exercise types are thought to impact on biological mechanisms involved in vascular physiology. Therefore ‘exercises’ aimed only to improve specific impairments such as balance or co-ordination, which meet the ACSM definition, will not meet our definition of an exercise intervention for the purpose of this review. This is particularly relevant in trials in stroke, where patients frequently have residual neurological impairments that can be improved by ‘exercises’ prescribed by therapists which may focus on skill related fitness factors. We have restricted our definition of exercise (and therefore scope) to focus on aerobic and strength training because these are biologically most likely to influence the risk of recurrent vascular events.

**Methods**

**Criteria for considering studies in this review**

**Types of studies**

We will include randomised controlled trials (RCTs). This includes cluster-randomised and cross-over trials. If cross-over trials are included, we will include only the first phase of the trial. We will include RCTs which compare an exercise intervention group with a no exercise group. Provided an exercise intervention fulfils our definition as described above, we will include it, irrespective of how it is delivered e.g. group or individual. We will not restrict study inclusion by length of follow-up. We will include all geographical settings including high, upper-middle, lower-middle- and low-income countries. We will exclude studies which use a quasi-randomisation process (e.g., alternate allocation).

**Population of interest**

We will include adults (18 and over), from any country, of any ethnic background or gender, diagnosed with a vascular condition due to arterial disease. This includes stroke, TIA, coronary artery disease (including stable angina, acute coronary syndrome, coronary revascularisation), heart failure, and PAD.

Stroke may be of ischaemic or haemorrhagic origin. Less common causes of ischaemic stroke include vertebral or carotid dissection. Around 13% of strokes are due to intracranial haemorrhage; these can be caused by SVD, hypertension, or less commonly other intracranial abnormalities such as arteriovenous malformations.^57^ For the purposes of this review, we will include trials where the authors include people with ‘stroke’ acknowledging that different trials may use different definitions - or may not provide a definition. In the UK, the term ‘stroke’ is usually not used for ‘silent cerebral infarcts’. We will exclude trials focused on silent stroke. This is in keeping with the Hatano 1976 definition of stroke.^58^ If populations are mixed, we will report this and include the trials in the review. Similarly, we will include participants with heart failure, acknowledging that not all heart failure is due to ischaemia, and may not be reported separately as ischaemic/non-ischaemic by trialists. We are including participants with TIA as the AHA definition of stroke includes TIA and in clinical practice the secondary prevention strategies applied after ischaemic stroke are generally the same as after TIA.^59^ We will exclude subarachnoid haemorrhage as the aetiology is generally not due to atheromatous disease. We will exclude studies focused on the primary conditions of atrial fibrillation, venous disease, diabetic microvascular disease, congenital heart disease, or adults after heart valve surgery, people with implantable ventricular assist devices, patients with an implantable cardioverter defibrillator, hypertension, and studies where pre-surgery exercise was to improve surgical outcomes. This is because these conditions are not generally caused by atheroma.

**Intervention**

We will include studies comparing an exercise group versus a no exercise comparator group.

For this review we define exercise intervention as consisting of planned, structured, and repetitive bodily movement undertaken to improve and/or maintain fitness, involving strength (resistance or power training) or aerobic (cardiorespiratory training) or mixed training. We will include studies with repeated sessions for a minimum duration of six weeks. We will include studies where the aim of the intervention is described as secondary prevention or improvement of physical fitness. We will exclude studies where the aim is to improve a specific skill (such as balance or co-ordination) unless they also meet our definition of exercise. As mentioned above, this is particularly relevant in trials in stroke. ‘Exercises’ prescribed by therapists to improve residual neurological impairments will be excluded as they do not meet our definition of exercise. We will exclude studies that lack an actual training component, e.g., in which interventions consist of advice to exercise only, or the promotion of physical activity only e.g. by information leaflets.

Interventions may be delivered in any setting (e.g., facilitated or independent, and group or individual). There will be no restriction to who delivers the exercise intervention. We will include studies where exercise is combined with co-interventions such as psychosocial or educational interventions, providing the exercise component meets our definition. We will include any type of technology incorporated in an exercise regime or setting such as immersive/ non-immersive virtual reality providing the exercise component meets our definition.

**Comparators**

As the focus of this review is to assess the question of efficacy, we will include studies with a no exercise intervention control. We will investigate two potential comparison groupings:

1. Exercise versus no exercise. This includes exercise delivered in addition to usual care versus usual care alone (where usual care is defined by the study and may involve advice to be active or exercise, but this is not part of a planned exercise programme). This also includes studies with an attention control group i.e. the control intervention is designed to provide similar amounts of social contact as the exercise.
2. Exercise interventions plus co-intervention versus co-intervention alone, provided that the effects of exercise can be determined (co-interventions can be education, diet advice and psychosocial).

**Types of outcome measures**

**Primary outcomes**

- All-cause mortality
- Cardiovascular mortality (including fatal MI and fatal stroke)
- Myocardial infraction (non-fatal)
- Stroke (non-fatal, including TIA)
- Hospitalisation due to cardiovascular events (including MI, stroke, TIA, unstable angina, revascularisation procedures including lower limb)
- Amputation
- Acute limb ischaemia
- Health-related quality of life – use of validated generic measurements (e.g. EQ-5D and SF36)

**Secondary outcomes**

- Exercise tolerance as assessed with submaximal or maximal exercise test e.g., 6-minute walk test, maximum walking distance (MWD), and cardiopulmonary testing (i.e. direct VO2max assessment)
- Muscle strength/power measured using validated tests as reported by the included studies
- MACE (Major Adverse Cardiovascular Events - composite outcome as defined by study)
- MALE (Major Adverse Limb Events - composite outcome as defined by study)

Outcome measurement points will be end of intervention period (note that we accept that this might vary depending on the duration of the intervention, but this is a pragmatic solution as most trials measure this time point and it will allow us to explore the effect of different durations of interventions), 12 months and five years follow up. If there are more or different follow-up points reported, we will include the one closest to 12 months and five years. We will include studies providing at least one time point is reported. When studies do not report our outcomes of interest, we will not calculate these from information in the studies. That is, we will not calculate composite MACE/MALE outcomes due to cardiovascular events by adding up events.

We also plan to record whether included studies reported on resource use, costs and cost effectiveness. Due to limited time resources, we will not analyse this as an outcome but will identify and report if studies performed a form of economic evaluation (cost consequence/cost effectiveness/cost utility) or not.

**Search methods for identification of studies**

Our search methods will involve identification of RCTs using five key existing Cochrane systematic reviews of exercise-based interventions in individual cardiovascular indications.^31, 39-42^

To ensure the contemporary nature of this review we will also search multiple databases for RCTs not included in the key Cochrane reviews (published after the search dates of the five key reviews, that is, 2016), or addressing cardiovascular populations not covered by the five Cochrane systematic reviews (people with TIA).

- MEDLINE via OVID
- Embase via OVID
- CINAHL via EBSCO
- The Cochrane Central Register of Controlled Trials (CENTRAL)
- World Health Organization International Clinical Trials Registry Platform (ICTRP)
- ClinicalTrials.gov

Appendix 1 includes the MEDLINE search strategy. This search will be adapted for other databases.

**Date and language limitations**

For search strategies addressing cardiovascular populations not covered by the five Cochrane systematic reviews, that is, people with TIA, we will limit the searches to post-1990. This is because treatments and usual care for vascular diseases have changed since then; this will ensure that our evidence is contemporaneous. We will exclude non-English language publications. We will also exclude studies where abstracts are in English but full texts are not. An exception to this is where non-English language publications are included in the Cochrane systematic reviews. If additional data is required, we will exclude.

**Data collection and analysis**

**Selection of studies**

We will consider the included studies of the five key Cochrane reviews according to the inclusion criteria of this review. If a study is relevant for inclusion, the related study references will be uploaded to Covidence.^60^ This will allow the team to keep track of data which are and are not available from the key Cochrane reviews, what still needs to be extracted and to have it in the same format as the data extracted from newly identified studies.

We will screen all references identified from the additional searches by title/abstract in Covidence and remove clearly non-relevant articles. ^60^ We will utilise the Covidence classifier function. Two review authors will independently assess all references assessed as potentially relevant by full text. Disagreements will be resolved by discussion with a third review author.

**Dealing with multiple related publications**

In the event of multiple publications relating to a study, we will combine the publications and extract the characteristics and data required from the relevant publications. If it is unclear whether two publications are reporting the same study/patients we will contact the author. Additional publications to studies included in the five Cochrane reviews identified in the new bibliographic searches will be matched with the previously included studies. They will then be assessed for any additional relevant information such as long-term follow-up not previously reported.

**Data extraction, coding and management**

One review author will extract study characteristics and data required for analysis from the selected included studies from the five key Cochrane reviews using a pre‐developed data extraction form within Covidence and this will be cross-checked by a second review author. Where data relevant for this review are not available in the Cochrane reviews, two review authors will independently extract study characteristics and data required for analysis from the original study publications into data extraction forms within Covidence.

Two review authors will independently extract study characteristics and data required for analysis from the included studies identified from the additional searches, using a pre‐developed data extraction form, within Covidence. We will resolve disagreements in discussion with a third review author.

We include a data extraction template for the information required for this review. The extraction form will be piloted on at least five studies from each index condition (PAD, stroke, coronary artery disease and heart failure) prior to use. This template will be used for the extraction of information and data from newly identified studies. This template will also be used to check information and data available in the five key Cochrane reviews to identify information and data which needs to be extracted *de novo*.

We will extract and categorise data on the following items:

- Author and year
- Study design (randomisation method)
- Publication status
- Aim (verbatim)
- Number of participants/dropouts or losses to follow up
- Inclusion and exclusion criteria (i.e., were people with multiple events or polyvascular conditions specifically excluded?)
- Geographical setting (countries)
- Demographic characteristics: sex, gender, ethnicity, age
- Condition: index condition leading to inclusion in the trial, time since index event, polyvascular conditions, mixed populations (ischaemic/non-ischaemic), comorbidities (singular vascular conditions will be indicated if polyvascular conditions have been excluded at the recruitment stage)
- Intervention characteristics:
- Number of intervention groups
- Setting where exercise delivered e.g., hospital, outpatient, home, community settings
- Mode of delivery e.g., face to face, virtual or mobile technology; individual or group
- Length of training (i.e. duration and programme length)
- Type of training (i.e. cardiorespiratory/aerobic, strength/resistance, or mixed)
- Training mode (e.g. treadmill walking, weight training, cycling, circuit training)
- Dose: frequency (number of sessions), intensity (low/light/moderate, high or not specified/unclear) and duration (length of session)
- Timing (i.e. during or after usual care)
- Length of training adherence to intervention (i.e. attendance, compliance)
- Exercise provider (e.g., professional, supervised or self-led)
- Personalisation or modifications during study
- Co-interventions (e.g., usual care (report how this was defined by study), education, diet in addition to usual care)
- Comparator characteristics: description of control
- Details of usual care
- Additional co-interventions
- Outcomes: in addition to the relevant outcomes of interest for this review (see section ‘Outcomes’), we will extract a list of all outcomes reported, outcome tools used, and time points reported
- Baseline and follow‐up results data (mean and standard deviation, or other summary statistics as appropriate). We will extract data for an 'immediate' time point – recorded at the end of the intervention period; and for a 'follow‐up' time point. Where multiple follow‐up time points are available, we will extract data closest to 12 months and closest to 5 years
- Study funding and conflict of interest
- Did study report costs/economic evaluation (yes/no/unclear and what type) – this information could be used to inform future economic evaluation synthesis
- Extraction method e.g., de novo, from published Cochrane review, or mixed.

**Assessment of risk of bias in included studies**

We will use existing risk of bias assessments of RCTs that were made by authors of the five key Cochrane reviews. We will undertake *de novo* risk of bias of new RCTs identified by our searches, using the Cochrane Risk of Bias 1 (RoB 1) method.^55^ For *de novo* risk of bias, two review authors will independently assess risk of bias. Any disagreements will be resolved through discussion with a third review author.

Each study will be judged as being at high, low or unclear risk of bias for the following five domains.

- Random sequence generation (selection bias)
- Allocation concealment (selection bias)
- Blinding of outcome assessment (detection bias)
- Incomplete outcome data (attrition bias, in particular non-completion of exercise programme)
- Selective outcome reporting (reporting bias)

Given the nature of the exercise interventions, it is not possible to blind participants or programme personnel. We will therefore not assess performance bias.

We will indicate where data is not provided in the original report and we will seek information from study authors if resources permit. We will use RevMan 5 to create risk of bias assessment visualisations.^61, 62^

**Measures of treatment effect**

We will report risk ratios (with 95% confidence intervals) for binary outcomes (all-cause mortality, cardiovascular mortality, non-fatal myocardial infraction, non-fatal stroke, hospitalisation, amputation, acute limb ischaemia, MACE, MALE). We will report mean differences (with 95% confidence intervals) for continuous outcomes (health-related quality of life (EQ-5D or SF-36), 6-minute walk test, maximum walking distance, and direct VO2max assessment) or standardised mean differences if different measures of the same outcomes have been used in different trials.

**Unit of analysis issues**

We anticipate that the vast majority of the included studies will have a parallel randomised design. The unit of analysis will be the individual participant. For the outcome hospitalisation, we plan to report the number of participants admitted to hospital at least once during follow-up, not the number of admissions. For studies reporting more than one active intervention arm which may be eligible for inclusion within the same comparison, we will divide the control group data between the pairwise comparisons in order to avoid double counting participants within an analysis. For cross‐over randomised studies we will analyse the data from the first phase of the trial. We will not extract data for later time points if it is not possible to determine if effects are due to first or second phase. For cluster‐randomised studies we will treat the group (or cluster) as the unit of analysis.^63^ If the intra‐cluster correlation coefficient (ICC) is not reported for a study and we are unable to obtain the ICC value from the study authors, then we will not include the study in our analyses. ^63^

**Dealing with missing data**

Where possible, we plan to contact study authors by email (with one reminder for non-responders) to obtain missing data relevant to our study characteristics, and primary and secondary outcomes. We will contact study authors when these data are missing from identified reports or where study reports do not provide means or standard deviations (or data from which these can be calculated by the review authors). We will only analyse available data and do not plan to impute or use replacement data.

**Assessment of heterogeneity**

We will assess heterogeneity between trials by inspecting forest plots and assessing the I^2^ statistic available in RevMan 5.^62^ We will consider an I² value of more than 50% to indicate substantial heterogeneity.^64^ Where we find substantial or considerable levels of heterogeneity, we will explore reasons for this heterogeneity using pre‐planned subgroup and sensitivity analyses.

**Assessment of reporting biases**

To assess whether trials included in any meta‐analysis are affected by reporting bias, we will construct funnel plots for meta-analyses of primary outcomes, when a meta‐analysis for an outcome includes results of at least ten trials.^65^

**Data synthesis**

We plan to conduct meta‐analyses when this is meaningful, that is if the interventions, participants, and the underlying clinical questions are similar enough for pooling to be appropriate. We will decide this by discussion involving at least three review authors. We will use the calculator in Review Manager to calculate effect estimates from individual studies.^62^ Due to expected heterogeneity, we plan to conduct a random‐effects meta‐analysis of included studies. We plan to perform meta-analyses which include participants with all vascular conditions combined (all key index conditions) to determine any effect of exercise on our outcomes (see outcomes). We will undertake meta-analyses at all time points for the primary outcomes only. We will undertake meta-analyses for the secondary outcomes at end of intervention time point only. If meta-analysis is not possible, we will consider by discussion with the review team if alternative synthesis are appropriate, using Cochrane guidance on synthesis without meta-analysis (SWIM).^66^

**Subgroup analysis and investigation of heterogeneity**

We plan to undertake the following subgroup analyses, to explore differences in effect estimates when more than 10 studies report an outcome in each subgroup:

- Polyvascular or single vascular condition (or unclear)
- Index condition (coronary artery disease (including angina), heart failure, stroke (including TIA), and peripheral arterial disease
- Exercise type e.g. aerobic alone, resistance alone, mixed (both)
- Total dose. We will use this to investigate exercise programmes as regards duration and frequency as categorising either in isolation may not provide an accurate picture (i.e., programmes may be for 30 minutes, once a week for 12 weeks or for one hour, twice a week for six weeks). We will calculate exercise dose by multiplying the number of weeks, by the number of sessions per week, by the session duration in hours (≤ 20 hours training in total vs > 20 hours in total)^67^
- Exercise intensity (high vs low/light/moderate vs unclear intensity)
- Studies with mixed populations (ischaemic vs ischaemic plus non-ischaemic (mixed))

We will use the test for subgroup interaction in RevMan 5 to perform these analyses. ^63^

**Sensitivity analysis**

We plan to undertake sensitivity analysis for our primary outcomes when more than 10 studies report an outcome. We plan to explore heterogeneity by carrying out sensitivity analyses to determine the impact of the following:

- Risk of bias: we plan to exclude studies at high risk of detection bias and attrition bias

**Summary of findings and assessment of the certainty of the evidence**

We will assess the certainty of the evidence for the effectiveness of exercise at end of intervention and at 12 months (or closest available time point) follow up using GRADE criteria.^68^ We will create one summary of findings table for each of our main: 1) Exercise vs no exercise and 2) Exercise plus co-interventions vs co-interventions. We will include the following outcomes in each SOF table:

All-cause mortality

Cardiovascular mortality (including fatal MI and fatal stroke)

Myocardial infraction (non-fatal)

Stroke (non-fatal, including TIA)

Hospitalisation due to cardiovascular events

Amputation

Acute limb ischaemia

HRQoL

**Stakeholder and patient and public involvement (PPI) engagement**

Meaningful PPI and stakeholder involvement is important for including expertise in key documents and activities. We will create a PPI and stakeholder plan that will describe their involvement and input throughout the project. We will contact people with lived experience and/or their carers, relevant third sector organisation, researchers, health professionals, commissioners and policy makers. We will gather feedback and advice from stakeholders on review findings, agreement on key implications and planning of dissemination of the findings of the review.

**Knowledge mobilisation / dissemination**

Our knowledge mobilisation plan will be developed with our PPI/ and stakeholder group throughout the course of the review. We will use appropriate traditional communication methods (e.g. research reports, scientific posters, and meeting presentations) and more innovative / creative methods (for example, elevator pitch / 2-3-minute talks, Tailored Talks, podcasts, webinars, story boards, infographics, visual abstracts, blogs, social media posts). A key aim of this plan will be how we intend to engage with and communicate the findings of this review to effectively reach the appropriate audience.

**Acknowledgements**

We would like to acknowledge Stefano Ricci, Coordinator, Scientific Committee of Italian Stroke Association for feedback on the protocol, and Aileen Neilson, Usher Institute, University of Edinburgh for health economic input.

**Contributions of authors**

Please see Appendix 2 for the CRediT author statement.

**Declarations of interest**

Cathryn Broderick none

Rod S Taylor none, author on previous systematic reviews on cardiac rehabilitation (chronic heart disease and heart failure) and is a clinical investigator/co-applicant for randomised controlled trials on cardiac rehabilitation

Alex Todhunter-Brown none

Catriona Keerie none

Frederike van Wijck receives royalties for a book about exercise training and fitness after stroke (<£50 per year)

Luciana Mendes none

Sheila Cameron none

Candida Fenton none

Marlene Stewart none

Gerry Stansby none

Gillian Mead receives royalties for a book about exercise training and fitness after stroke (<£50 per year) and occasionally lectures on this topic and receives honoraria (paid into University of Edinburgh account) and expenses

**Figures and Tables**

Table 1. List of Cochrane five key reviews

| **Title of Cochrane review** | **Index condition** | **Reference** |
| --- | --- | --- |
| Exercise-based cardiac rehabilitation for coronary heart disease | Coronary artery disease | 31 |
| Physical fitness training for stroke patients | Stroke | 39 |
| Exercise for intermittent claudication | PAD | 40 |
| Exercise-based cardiac rehabilitation for adults with stable angina | Coronary artery disease - stable angina | 41 |
| Exercise-based cardiac rehabilitation for adults with heart failure | Heart failure | 42 |

**Appendices**

**Appendix 1 search strategy:**

1 "Intracranial Embolism and Thrombosis"/rh, th [Rehabilitation, Therapy]

2 Angina Pectoris/rh, th [Rehabilitation, Therapy]

3 Angina, Stable/rh, th [Rehabilitation, Therapy]

4 exp Angioplasty/rh, th [Rehabilitation, Therapy]

5 Arterial Occlusive Diseases/rh, th [Rehabilitation, Therapy]

6 Arteriolosclerosis/

7 exp Arteriosclerosis/

8 Arteriosclerosis Obliterans/

9 Atherectomy/

10 Atherosclerosis/

11 Basal Ganglia Cerebrovascular Disease/

12 exp Brain Infarction/

13 Brain Ischemia/

14 exp Carotid Artery Diseases/

15 Coronary Artery Bypass/

16 exp Coronary Disease/rh, th [Rehabilitation, Therapy]

17 exp Heart Failure/rh, th [Rehabilitation, Therapy]

18 Hemiplegia/

19 exp Intermittent Claudication/rh, th [Rehabilitation, Therapy]

20 exp Intracranial Arterial Diseases/

21 exp Intracranial Arteriovenous Malformations/

22 exp Intracranial Hemorrhages/

23 exp Myocardial Infarction/rh, th [Rehabilitation, Therapy]

24 Myocardial Ischemia/rh, th [Rehabilitation, Therapy]

25 exp Myocardial Revascularization/

26 Percutaneous Coronary Intervention/

27 exp Peripheral Vascular Diseases/rh, th [Rehabilitation, Therapy]

28 exp Stroke/pp, th [Physiopathology, Therapy]

29 exp Stroke Rehabilitation/mt [Methods]

30 Stroke, Lacunar/rh, th [Rehabilitation, Therapy]

31 Vasospasm, Intracranial/

32 Vertebral Artery Dissection/

33 "acute coronary syndrom*".ti,ab.

34 "angor pectoris".ti,ab.

35 "brain vasc*".ti,ab.

36 "cerebral vasc*".ti,ab.

37 "coronary heart disease".ti,ab.

38 "endoluminal repair*".ti,ab.

39 "heart failure".ti,ab.

40 "intermittent claudication".ti,ab.

41 "post‐stroke".ti,ab.

42 angina.ti,ab.

43 angioplast*.ti,ab.

44 apoplex*.ti,ab.

45 arteriosclero*.ti,ab.

46 atherectom*.ti,ab.

47 atherosclero*.ti,ab.

48 cerebrovasc*.ti,ab.

49 "intermittent claudic*".ti,ab.

50 hemipleg*.ti,ab.

51 hempar*.ti,ab.

52 poststroke.ti,ab.

53 stenocardia*.ti,ab.

54 stroke.ti,ab.

55 ((brain* or cerebr* or cerebell*or intracran* or intracerebral) adj (ischemi* or ischaemi* or infarct* or thrombo* or emboli* or occlus*)).ti,ab.

56 ((heart or myocard* or coronary) adj (ischemi* or ischaemi* or infarct* or failure or infarct*)).ti,ab.

57 (coronary adj (disease* or bypass or thrombo* or angioplast*)).ti,ab.

58 (percutaneous coronary adj (interven* or revascular*)).ti,ab.

59 ((arter* or vascular or vein* or veno* or peripher*) adj (occlus* or reocclus* or steno* or obstruct* or lesio* or block* or harden* or stiffen*)).ti,ab.

60 (peripheral adj2 dis*).ti,ab.

61 or/1-60

62 exp Exercise/

63 exp "Physical Education and Training"/

64 exp Physical Exertion/

65 exp Sports/

66 exp Walking/

67 exp Exercise Therapy/mt [Methods]

68 "circuit training".ti,ab.

69 cycling.ti,ab.

70 danc*.ti,ab.

71 exercise*.ti,ab.

72 kinesiotherap*.ti,ab.

73 running.ti,ab.

74 squat*.ti,ab.

75 strengthen*.ti,ab.

76 swim*.ti,ab.

77 training.ti,ab.

78 treadmill.ti,ab.

79 walk*.ti,ab.

80 (exercise* adj (activit* or Endurance or intervention* or program* or protocol* or regim* or therap* or treatment or fitness or strength or aerobic)).ti,ab.

81 (fitness adj (activit* or Endurance or intervention* or program* or protocol* or regim* or therap* or treatment or fitness or strength or aerobic)).ti,ab.

82 (physical adj (activit* or Endurance or intervention* or program* or protocol* or regim* or therap* or treatment or fitness or strength or aerobic)).ti,ab.

83 or/62-82

84 61 and 83

85 randomized controlled trial.pt.

86 controlled clinical trial.pt.

87 randomized.ab.

88 placebo.ab.

89 drug therapy.fs.

90 randomly.ab.

91 trial.ab.

92 groups.ab.

93 or/85-92

94 exp animals/ not humans.sh.

95 93 not 94

96 84 and 95

97 (2016* or 2017* or 2018* or 2019* or 2020* or 2021* or 2022* or 2023*).ed.

98 96 and 97

**Appendix 2: CRediT author statement**

| **Term** | **Definition** | **Contributors** |
| --- | --- | --- |
| Conceptualization | Ideas; formulation or evolution of overarching research goals and aims | GEM, CB, ATB, LM, FvW, RT, CK, SC, GS, MS |
| Methodology | Development or design of methodology; creation of models | GEM, CB, ATB, LM, FvW, RT, CK, SC, GS, MS |
| Software | Programming, software development; designing computer programs; implementation of the computer code and supporting algorithms; testing of existing code components; designing of search strategies | CF |
| Validation | Verification, whether as a part of the activity or separate, of the overall replication/ reproducibility of results/experiments and other research outputs | NA for protocol |
| Formal analysis | Application of statistical, mathematical, computational, or other formal techniques to analyse or synthesize study data | NA for protocol |
| Investigation | Conducting a research and investigation process, specifically performing the experiments, or data/evidence collection | NA for protocol |
| Resources | Provision of study materials, reagents, materials, patients, laboratory samples, animals, instrumentation, computing resources, or other analysis tools | NA for protocol |
| Data Curation | Management activities to annotate (produce metadata), scrub data and maintain research data (including software code, where it is necessary for interpreting the data itself) for initial use and later reuse | NA for protocol |
| Writing - Original Draft | Preparation, creation and/or presentation of the published work, specifically writing the initial draft (including substantive translation) | GEM, CB, ATB, LM FvW, RT, CK, SC, GS, MS |
| Writing - Review & Editing | Preparation, creation and/or presentation of the published work by those from the original research group, specifically critical review, commentary or revision – including pre-or postpublication stages | GEM, CB, ATB, LM, FvW, RT, CK, SC, GS, MS |
| Visualization | Preparation, creation and/or presentation of the published work, specifically visualization/ data presentation | N/A for protocol |
| Supervision | Oversight and leadership responsibility for the research activity planning and execution, including mentorship external to the core team | GEM, CB, MS |
| Project administration | Management and coordination responsibility for the research activity planning and execution | CB, MS |
| Funding acquisition | Acquisition of the financial support for the project leading to this publication | N/A |

**References**

1. British Heart Foundation. *Heart statisics*. 2023. URL: <https://www.bhf.org.uk/what-we-do/our-research/heart-statistics> (accessed 25 October, 2023).

2. WHO. *Cardiovascular diseases (CVDs)*. URL: <https://www.who.int/news-room/fact-sheets/detail/cardiovascular-diseases-(cvds>) (accessed 25 October, 2023).

3. Virani SS, Alonso A, Benjamin EJ, Bittencourt MS, Callaway CW, Carson AP*, et al.* Heart disease and stroke statistics - 2020 update: a report from the American Heart Association. *Circulation* 2020;**141**:e139-e596. <https://doi.org/10.1161/cir.0000000000000757>

4. Timmis A, Vardas P, Townsend N, Torbica A, Katus H, De Smedt D*, et al.* European Society of Cardiology: cardiovascular disease statistics 2021. *European Heart Journal* 2022;**43**:716-99.

5. Wang Y, Wu H, Sun J, Wei M, Wang J, Li H*, et al.* Effect of exercise on carotid artery intima-media thickness in adults: a systematic review and meta-analysis. *J Phys Act Health* 2022;**19**:855-67. <https://doi.org/10.1123/jpah.2022-0372>

6. Damay VA, Setiawan S, Lesmana R, Akbar MR, Lukito AA. Effects of moderate intensity aerobic exercise to FSTL-1 regulation in atherosclerosis: a systematic review. *Int J Angiol* 2023;**32**:1-10. <https://doi.org/10.1055/s-0042-1750184>

7. Arnett DK, Blumenthal RS, Albert MA, Buroker AB, Goldberger ZD, Hahn EJ*, et al.* 2019 ACC/AHA guideline on the primary prevention of cardiovascular disease: a report of the American College of Cardiology/American Heart Association Task Force on clinical practice guidelines. *Circulation* 2019;**140**:e596-e646. <https://doi.org/10.1161/cir.0000000000000678>

8. Libby P. The changing landscape of atherosclerosis. *Nature* 2021;**592**:524-33. <https://doi.org/10.1038/s41586-021-03392-8>

9. Meyer-Lindemann U, Moggio A, Dutsch A, Kessler T, Sager HB. The impact of exercise on immunity, metabolism, and atherosclerosis. *Int J Mol Sci* 2023;**24**. <https://doi.org/10.3390/ijms24043394>

10. Ross R. The pathogenesis of atherosclerosis: a perspective for the 1990s. *Nature* 1993;**362**:801-9. <https://doi.org/10.1038/362801a0>

11. Arnett DK, Khera A, Blumenthal RS. 2019 ACC/AHA guideline on the primary prevention of cardiovascular disease: Part 1, lifestyle and behavioral factors. *JAMA Cardiol* 2019;**4**:1043-4. <https://doi.org/10.1001/jamacardio.2019.2604>

12. Chen YC, Huang AL, Kyaw TS, Bobik A, Peter K. Atherosclerotic plaque rupture: identifying the straw that breaks the camel's back. *Arterioscler Thromb Vasc Biol* 2016;**36**:e63-72. <https://doi.org/10.1161/atvbaha.116.307993>

13. Fan J, Watanabe T. Atherosclerosis: known and unknown. *Pathol Int* 2022;**72**:151-60. <https://doi.org/10.1111/pin.13202>

14. Bhatt DL, Eagle KA, Ohman EM, Hirsch AT, Goto S, Mahoney EM*, et al.* Comparative determinants of 4-year cardiovascular event rates in stable outpatients at risk of or with atherothrombosis. *Jama* 2010;**304**:1350-7. <https://doi.org/10.1001/jama.2010.1322>

15. Gutierrez JA, Aday AW, Patel MR, Jones WS. Polyvascular disease: reappraisal of the current clinical landscape. *Circ Cardiovasc Interv* 2019;**12**:e007385. <https://doi.org/10.1161/circinterventions.119.007385>

16. Wardlaw JM, Smith C, Dichgans M. Small vessel disease: mechanisms and clinical implications. *Lancet Neurol* 2019;**18**:684-96. <https://doi.org/10.1016/s1474-4422(19)30079-1>

17. Mahammedi A, Wang LL, Williamson BJ, Khatri P, Kissela B, Sawyer RP*, et al.* Small vessel disease, a marker of brain health: what the radiologist needs to know. *Am J Neuroradiol* 2022;**43**:650-60. <https://doi.org/10.3174/ajnr.A7302>

18. Mead GE, Sposato LA, Sampaio Silva G, Yperzeele L, Wu S, Kutlubaev M*, et al.* A systematic review and synthesis of global stroke guidelines on behalf of the World Stroke Organization. *Int J Stroke* 2023;**18**:499-531. <https://doi.org/10.1177/17474930231156753>

19. NICE. *Chronic heart failure in adults: diagnosis and management NICE guideline [NG106]*. 2018. URL: <https://www.nice.org.uk/guidance/ng106> (accessed 22 November, 2023).

20. NICE. *Peripheral arterial disease: diagnosis and management Clinical guideline [CG147]*. 2020. URL: <https://www.nice.org.uk/guidance/cg147> (accessed 22 November 2023).

21. NICE. *Acute coronary syndromes NICE guideline [NG185]*. 2020. URL: <https://www.nice.org.uk/guidance/NG185> (accessed 22 November, 2023).

22. NICE. *Chronic heart failure in adults Quality standard [QS9]*. 2023. URL: <https://www.nice.org.uk/guidance/qs9> (accessed 22 November, 2023).

23. Knuuti J, Wijns W, Saraste A, Capodanno D, Barbato E, Funck-Brentano C*, et al.* 2019 ESC Guidelines for the diagnosis and management of chronic coronary syndromes. *Eur Heart J* 2020;**41**:407-77. <https://doi.org/10.1093/eurheartj/ehz425>

24. McDonagh TA, Metra M, Adamo M, Gardner RS, Baumbach A, Böhm M*, et al.* 2021 ESC duidelines for the diagnosis and treatment of acute and chronic heart failure: developed by the Task Force for the diagnosis and treatment of acute and chronic heart failure of the European Society of Cardiology (ESC) with the special contribution of the Heart Failure Association (HFA) of the ESC. *Rev Esp Cardiol (Engl Ed)* 2022;**75**:523. <https://doi.org/10.1016/j.rec.2022.05.005>

25. Heidenreich PA, Bozkurt B, Aguilar D, Allen LA, Byun JJ, Colvin MM*, et al.* 2022 AHA/ACC/HFSA guideline for the management of heart failure: a report of the American College of Cardiology/American Heart Association Joint Committee on clinical practice guidelines. *Circulation* 2022;**145**:e895-e1032. <https://doi.org/10.1161/cir.0000000000001063>

26. Virani SS, Newby LK, Arnold SV, Bittner V, Brewer LC, Demeter SH*, et al.* 2023 AHA/ACC/ACCP/ASPC/NLA/PCNA guideline for the management of patients with chronic coronary disease: a report of the American Heart Association/American College of Cardiology Joint Committee on clinical practice guidelines. *Circulation* 2023;**148**:e9-e119. <https://doi.org/10.1161/cir.0000000000001168>

27. Intercollegiate Stroke Working Party. *National Clinical Guideline for Stroke for the UK and Ireland*. London; 2023. URL: [www.strokeguideline.org](file:///C:/Users/mstewar7/AppData/Local/Microsoft/Windows/INetCache/Content.Outlook/CIQQNU8J/www.strokeguideline.org) (accessed 21 November, 2023).

28. Caspersen CJ, Powell KE, Christenson GM. Physical activity, exercise, and physical fitness: definitions and distinctions for health-related research. *Public Health Rep* 1985;**100**:126-31.

29. Garber CE, Blissmer B, Deschenes MR, Franklin BA, Lamonte MJ, Lee IM*, et al.* American College of Sports Medicine position stand. Quantity and quality of exercise for developing and maintaining cardiorespiratory, musculoskeletal, and neuromotor fitness in apparently healthy adults: guidance for prescribing exercise. *Med Sci Sports Exerc* 2011;**43**:1334-59. <https://doi.org/10.1249/MSS.0b013e318213fefb>

30. ACSM. *American College of Sports Medicine guidelines for exercise testing and prescription*: Sixth edition. Philadelphia: Lippincott Williams &amp; Wilkins; 2000.

31. Dibben G, Faulkner J, Oldridge N, Rees K, Thompson DR, Zwisler AD*, et al.* Exercise-based cardiac rehabilitation for coronary heart disease. *Cochrane Database Syst Rev* 2021;**11**:Cd001800. <https://doi.org/10.1002/14651858.CD001800.pub4>

32. Frodermann V, Rohde D, Courties G, Severe N, Schloss MJ, Amatullah H*, et al.* Exercise reduces inflammatory cell production and cardiovascular inflammation via instruction of hematopoietic progenitor cells. *Nat Med* 2019;**25**:1761-71. <https://doi.org/10.1038/s41591-019-0633-x>

33. Lavie CJ, Ozemek C, Carbone S, Katzmarzyk PT, Blair SN. Sedentary behavior, exercise, and cardiovascular health. *Circ Res* 2019;**124**:799-815. <https://doi.org/10.1161/circresaha.118.312669>

34. Andreozzi GM, Leone A, Laudani R, Deinite G, Martini R. Acute impairment of the endothelial function by maximal treadmill exercise in patients with intermittent claudication, and its improvement after supervised physical training. *Int Angiol* 2007;**26**:12-7.

35. Stewart KJ, Hiatt WR, Regensteiner JG, Hirsch AT. Exercise training for claudication. *N Engl J Med* 2002;**347**:1941-51. <https://doi.org/10.1056/NEJMra021135>

36. Tisi PV, Hulse M, Chulakadabba A, Gosling P, Shearman CP. Exercise training for intermittent claudication: does it adversely affect biochemical markers of the exercise-induced inflammatory response? *Eur J Vasc Endovasc Surg* 1997;**14**:344-50. <https://doi.org/10.1016/s1078-5884(97)80283-3>

37. Ehrman JK, Gardner AW, Salisbury D, Lui K, Treat-Jacobson D. Supervised exercise therapy for symptomatic peripheral artery disease: a review of current experience and practice-based recommendations. *J Cardiopulm Rehabil Prev* 2023;**43**:15-21. <https://doi.org/10.1097/hcr.0000000000000723>

38. Landman TR, Thijssen DH, Tuladhar AM, de Leeuw FE. Relation between physical activity and cerebral small vessel disease: a nine-year prospective cohort study. *Int J Stroke* 2021;**16**:962-71. <https://doi.org/10.1177/1747493020984090>

39. Saunders DH, Sanderson M, Hayes S, Johnson L, Kramer S, Carter DD*, et al.* Physical fitness training for stroke patients. *Cochrane Database Syst Rev* 2020;**3**:Cd003316. <https://doi.org/10.1002/14651858.CD003316.pub7>

40. Lane R, Harwood A, Watson L, Leng GC. Exercise for intermittent claudication. *Cochrane Database Syst Rev* 2017;**12**:Cd000990. <https://doi.org/10.1002/14651858.CD000990.pub4>

41. Long L, Anderson L, Dewhirst AM, He J, Bridges C, Gandhi M*, et al.* Exercise-based cardiac rehabilitation for adults with stable angina. *Cochrane Database Syst Rev* 2018;**2**:Cd012786. <https://doi.org/10.1002/14651858.CD012786.pub2>

42. Long L, Mordi IR, Bridges C, Sagar VA, Davies EJ, Coats AJ*, et al.* Exercise-based cardiac rehabilitation for adults with heart failure. *Cochrane Database Syst Rev* 2019;**1**:Cd003331. <https://doi.org/10.1002/14651858.CD003331.pub5>

43. Yamamoto S, Hotta K, Ota E, Mori R, Matsunaga A. Effects of resistance training on muscle strength, exercise capacity, and mobility in middle-aged and elderly patients with coronary artery disease: A meta-analysis. *J Cardiol* 2016;**68**:125-34. <https://doi.org/10.1016/j.jjcc.2015.09.005>

44. Verweij L, van de Korput E, Daams JG, Ter Riet G, Peters RJG, Engelbert RHH*, et al.* Effects of Postacute Multidisciplinary Rehabilitation Including Exercise in Out-of-Hospital Settings in the Aged: Systematic Review and Meta-analysis. *Arch Phys Med Rehabil* 2019;**100**:530-50. <https://doi.org/10.1016/j.apmr.2018.05.010>

45. Dibben GO, Faulkner J, Oldridge N, Rees K, Thompson DR, Zwisler AD*, et al.* Exercise-based cardiac rehabilitation for coronary heart disease: a meta-analysis. *Eur Heart J* 2023;**44**:452-69. <https://doi.org/10.1093/eurheartj/ehac747>

46. Mackie P, Eng JJ. The influence of seated exercises on balance, mobility, and cardiometabolic health outcomes in individuals living with a stroke: A systematic review and meta-analysis. *Clin Rehabil* 2023;**37**:927-41. <https://doi.org/10.1177/02692155221150002>

47. Zhang H, Chang R. Effects of Exercise after Percutaneous Coronary Intervention on Cardiac Function and Cardiovascular Adverse Events in Patients with Coronary Heart Disease: Systematic Review and Meta-Analysis. *J Sports Sci Med* 2019;**18**:213-22.

48. Gonzalez-Jaramillo N, Wilhelm M, Arango-Rivas AM, Gonzalez-Jaramillo V, Mesa-Vieira C, Minder B*, et al.* Systematic Review of Physical Activity Trajectories and Mortality in Patients With Coronary Artery Disease. *J Am Coll Cardiol* 2022;**79**:1690-700. <https://doi.org/10.1016/j.jacc.2022.02.036>

49. Kim M, Kim C, Kim E, Choi M. Effectiveness of Mobile Health-Based Exercise Interventions for Patients with Peripheral Artery Disease: Systematic Review and Meta-Analysis. *JMIR Mhealth Uhealth* 2021;**9**:e24080. <https://doi.org/10.2196/24080>

50. Brunt A, Albines D, Hopkins-Rosseel D. The Effectiveness of Exercise on Cognitive Performance in Individuals with Known Vascular Disease: A Systematic Review. *J Clin Med* 2019;**8**. <https://doi.org/10.3390/jcm8030294>

51. Dalal HM, Doherty P, Taylor RS. Cardiac rehabilitation. *BMJ* 2015;**351**:h5000. <https://doi.org/10.1136/bmj.h5000>

52. Ski CF, Thompson DR. Quality of life in cardiovascular disease: what is it and why and how should we measure it? *Eur J Cardiovasc Nurs* 2010;**9**:201-2. <https://doi.org/10.1016/j.ejcnurse.2010.08.002>

53. Raja A, Spertus J, Yeh RW, Secemsky EA. Assessing health-related quality of life among patients with peripheral artery disease: a review of the literature and focus on patient-reported outcome measures. *Vasc Med* 2021;**26**:317-25. <https://doi.org/10.1177/1358863x20977016>

54. Carod-Artal FJ, Egido JA. Quality of life after stroke: the importance of a good recovery. *Cerebrovasc Dis* 2009;**27 Suppl 1**:204-14. <https://doi.org/10.1159/000200461>

55. Higgins JPT. *Cochrane Handbook for Systematic Reviews of Interventions. Version 6.4 (updated August 2023)*. 2023. URL: [www.training.cochrane.org/handbook](file:///C:/Users/mstewar7/AppData/Local/Microsoft/Windows/INetCache/Content.Outlook/CIQQNU8J/www.training.cochrane.org/handbook) (accessed).

56. Page MJ, Higgins JPT, Sterne JAC, McKenzie JE, Bossuyt PM, Boutron I*, et al.* The PRISMA 2020 statement: an updated guideline for reporting systematic reviews. *BMJ* 2021;**372**:n71. <https://doi.org/10.1136/bmj.n71>

57. American Stroke Association. *Hemorrhagic stroke: what is hemorrhagic stroke?* URL: <https://www.stroke.org/en/about-stroke/types-of-stroke/hemorrhagic-strokes-bleeds> (accessed 16 November, 2023).

58. Hatano S. Experience from a multicentre stroke register: a preliminary report. *Bull World Health Organ* 1976;**54**:541-53.

59. WNO. *Implications of the AHA/ASA updated definition of stroke for the 21st Century*. 2013. URL: <https://worldneurologyonline.com/article/implications-of-the-ahaasa-updated-definition-of-stroke-for-the-21st-century/> (accessed 16 November, 2023).

60. Covidence. Covidence systematic review software. In. Melbourne, Australia: Veritas Health Innovation: Covidence systematic review software

61. McGuinness LA, Higgins JPT. Risk-of-bias VISualization (robvis): an R package and Shiny web app for visualizing risk-of-bias assessments. *Research Synthesis Methods* 2020;**n/a**. <https://doi.org/10.1002/jrsm.1411>

62. RevMan 5. Review Manager 5. In. Copenhagen: The Cochrane Collaboration; 2020.

63. Higgins JPT, Eldridge S, Li T. *Chapter 23: Including variants on randomized trials. Cochrane Handbook for Systematic Reviews of Interventions version 6.4 (updated August 2023)*. URL: [www.training.cochrane.org/handbook](file:///C:/Users/mstewar7/AppData/Local/Microsoft/Windows/INetCache/Content.Outlook/CIQQNU8J/www.training.cochrane.org/handbook). (accessed 22 November, 2023).

64. Deeks JJ, Higgins JPT, Altman DG. *Chapter 10: Analysing data and undertaking meta-analyses. Cochrane Handbook for Systematic Reviews of Interventions version 6.4 (updated August 2023)*. URL: [www.training.cochrane.org/handbook](file:///C:/Users/mstewar7/AppData/Local/Microsoft/Windows/INetCache/Content.Outlook/CIQQNU8J/www.training.cochrane.org/handbook) (accessed 22 November, 2023).

65. Page MJ, Higgins JPT, Sterne JAC. *Chapter 13: Assessing risk of bias due to missing results in a synthesis. Cochrane Handbook for Systematic Reviews of Interventions version 6.4 (updated August 2023)*. 2023. URL: [www.training.cochrane.org/handbook](file:///C:/Users/mstewar7/AppData/Local/Microsoft/Windows/INetCache/Content.Outlook/CIQQNU8J/www.training.cochrane.org/handbook) (accessed 22 November, 2023).

66. McKenzie JE BS. *Chapter 12: Synthesizing and presenting findings using other methods. In: Higgins JPT, Thomas J, Chandler J, Cumpston M, Li T, Page MJ, Welch VA (editors). Cochrane Handbook for Systematic Reviews of Interventions version 6.4 (updated August 2023). Cochrane*. 2023. URL: Available from [www.training.cochrane.org/handbook](file:///C:/Users/mstewar7/AppData/Local/Microsoft/Windows/INetCache/Content.Outlook/CIQQNU8J/www.training.cochrane.org/handbook) (accessed).

67. French B, Thomas LH, Coupe J, McMahon NE, Connell L, Harrison J*, et al.* Repetitive task training for improving functional ability after stroke. *Cochrane Database of Systematic Reviews* 2016; 10.1002/14651858.CD006073.pub3. <https://doi.org/10.1002/14651858.CD006073.pub3>

68. Schünemann HJ, Higgins JPT, Vist GE, Glasziou P, Akl EA, Skoetz N*, et al.* *Chapter 14: Completing ‘Summary of findings’ tables and grading the certainty of the evidence. Cochrane Handbook for Systematic Reviews of Interventions version 6.4 (updated August 2023)*. URL: [www.training.cochrane.org/handbook](file:///C:/Users/mstewar7/AppData/Local/Microsoft/Windows/INetCache/Content.Outlook/CIQQNU8J/www.training.cochrane.org/handbook) (accessed 22 November, 2023).

# S2: Search strategy

**Review title: The effects of exercise on secondary prevention and quality of life in people with existing vascular disease: systematic review and meta-analysis of randomised controlled trials**

Time frame: 1 Jan 2026 to 7 Feb 2024 
Updated search: 1 Jan 2024 to 20 Jan 2025

| **Contact Person:** |  |
| --- | --- |
| **E-mail:** |  |
| **E-mail 2:** |  |
| **Searches by:  Candida Fenton** |  |
| **Search results sent: current date** |  |
| **Results:** |  |
| **TOTAL:**  **Feb 2024**  **Jan 2025** | 55219  7187 |
| **Total after de-duplication in Endnote**  **Feb 2024**  **Jan 2025** | 34082  5618 |
| **Total after de-duplication and automatic screening in Covidence**  **Feb 2024**  **Jan 2025** | 25663  3566 |

| Source | Version/Platform/url | Date of Search | Records retrieved |
| --- | --- | --- | --- |
| 1. CENTRAL | CRSO | 7.2.24  20.1.25 | 18118  1596 |
| 2. MEDLINE In-process and other non-indexed citations and MEDLINE 1950-present | Ovid | 12.2.24  20.1.25 | 10625  1660 |
| 3. EMBASE  1974 to present | Ovid | 12.2.24  20.1.25 | 19163  2922 |
| 4. CINAHL | EBSCO | 7.2.24  20.1.25 | 4558  490 |
| 5. ClinicalTrials.gov | [www.clinicaltrials.gov](http://www.clinicaltrials.gov/) | 12.2.24  20.1.25 | 1782  359 |
| 6. ICTRP | <https://www.who.int/clinical-trials-registry-platform/the-ictrp-search-portal> | 12.2.24  20.1.25 | 973  160 |
| TOTAL before de-duplication |  | 12.2.24  20.1.25 | 55219  7187 |

**Search Strategies:**

| **Source** | **Search strategy** | **Hits retrieved** |
| --- | --- | --- |
| 1. CENTRAL via CRSO | #1 MESH DESCRIPTOR Intracranial Embolism and Thrombosis EXPLODE ALL TREES WITH QUALIFIERS RH,TH  #2 MESH DESCRIPTOR Angina Pectoris EXPLODE ALL TREES WITH QUALIFIERS RH,TH  #3 MESH DESCRIPTOR Angina, Stable EXPLODE ALL TREES WITH QUALIFIERS RH,TH  #4 MESH DESCRIPTOR Angioplasty EXPLODE ALL TREES WITH QUALIFIERS RH  #5 MESH DESCRIPTOR Arterial Occlusive Diseases WITH QUALIFIERS RH,TH  #6 MESH DESCRIPTOR Arteriolosclerosis EXPLODE ALL TREES  #7 MESH DESCRIPTOR Arteriosclerosis EXPLODE ALL TREES  #8 MESH DESCRIPTOR Arteriosclerosis Obliterans  #9 MESH DESCRIPTOR Atherectomy  #10 MESH DESCRIPTOR Atherosclerosis  #11 MESH DESCRIPTOR Basal Ganglia Cerebrovascular Disease  #12 MESH DESCRIPTOR Brain Infarction EXPLODE ALL TREES  #13 MESH DESCRIPTOR Brain Ischemia  #14 MESH DESCRIPTOR Carotid Artery Diseases EXPLODE ALL TREES  #15 MESH DESCRIPTOR Coronary Artery Bypass  #16 MESH DESCRIPTOR Coronary Disease EXPLODE ALL TREES WITH QUALIFIERS RH,TH  #17 MESH DESCRIPTOR Heart Failure EXPLODE ALL TREES WITH QUALIFIERS RH,TH  #18 MESH DESCRIPTOR Hemiplegia  #19 MESH DESCRIPTOR Intermittent Claudication EXPLODE ALL TREES WITH QUALIFIERS RH,TH  #20 MESH DESCRIPTOR Intracranial Arterial Diseases EXPLODE ALL TREES  #21 MESH DESCRIPTOR Intracranial Arteriovenous Malformations EXPLODE ALL TREES  #22 MESH DESCRIPTOR Intracranial Hemorrhages EXPLODE ALL TREES  #23 MESH DESCRIPTOR Myocardial Infarction EXPLODE ALL TREES WITH QUALIFIERS RH,TH  #24 MESH DESCRIPTOR Myocardial Ischemia EXPLODE ALL TREES WITH QUALIFIERS RH,TH  #25 MESH DESCRIPTOR Myocardial Revascularization EXPLODE ALL TREES  #26 MESH DESCRIPTOR Percutaneous Coronary Intervention  #27 MESH DESCRIPTOR Peripheral Vascular Diseases EXPLODE ALL TREES WITH QUALIFIERS RH,TH  #28 MESH DESCRIPTOR Stroke EXPLODE ALL TREES WITH QUALIFIERS PP,TH  #29 MESH DESCRIPTOR Stroke Rehabilitation EXPLODE ALL TREES WITH QUALIFIERS MT  #30 MESH DESCRIPTOR Stroke, Lacunar EXPLODE ALL TREES WITH QUALIFIERS RH,TH  #31 MESH DESCRIPTOR Vasospasm, Intracranial  #32 MESH DESCRIPTOR Vertebral Artery Dissection  #33 (acute coronary syndrom*):TI,AB,KY  #34 (angor pectoris):TI,AB,KY  #35 (brain vasc*):TI,AB,KY  #36 (cerebral vasc*):TI,AB,KY  #37 (coronary heart disease):TI,AB,KY  #38 (endoluminal repair*):TI,AB,KY  #39 (heart failure):TI,AB,KY  #40 (intermittent claudication):TI,AB,KY  #41 post?stroke:TI,AB,KY  #42 angina:TI,AB,KY  #43 angioplast*:TI,AB,KY  #44 apoplex*:TI,AB,KY  #45 arteriosclero*:TI,AB,KY  #46 atherectom*:TI,AB,KY  #47 atherosclero*:TI,AB,KY  #48 cerebrovasc*:TI,AB,KY  #49 ("intermittent claudic*"):TI,AB,KY  #50 hemipleg*:TI,AB,KY  #51 hempar*:TI,AB,KY  #52 poststroke:TI,AB,KY  #53 stenocardia*:TI,AB,KY  #54 stroke:TI,AB,KY  #55 (((brain* or cerebr* or cerebell*or intracran* or intracerebral) adj (ischemi* or ischaemi* or infarct* or thrombo* or emboli* or occlus*))):TI,AB,KY  #56 (((heart or myocard* or coronary) adj (ischemi* or ischaemi* or infarct* or failure or infarct*))):TI,AB,KY  #57 ((coronary adj (disease* or bypass or thrombo* or angioplast*))):TI,AB,KY  #58 ((percutaneous coronary adj (interven* or revascular*))):TI,AB,KY  #59 (((arter* or vascular or vein* or veno* or peripher*) adj (occlus* or reocclus* or steno* or obstruct* or lesio* or block* or harden* or stiffen*))):TI,AB,KY  #60 ((peripheral adj2 dis*)):TI,AB,KY  #61 #1 OR #2 OR #3 OR #4 OR #5 OR #6 OR #7 OR #8 OR #9 OR #10 OR #11 OR #12 OR #13 OR #14 OR #15 OR #16 OR #17 OR #18 OR #19 OR #20 OR #21 OR #22 OR #23 OR #24 OR #25 OR #26 OR #27 OR #28 OR #29 OR #30 OR #31 OR #32 OR #33 OR #34 OR #35 OR #36 OR #37 OR #38 OR #39 OR #40 OR #41 OR #42 OR #43 OR #44 OR #45 OR #46 OR #47 OR #48 OR #49 OR #50 OR #51 OR #52 OR #53 OR #54 OR #55 OR #56 OR #57 OR #58 OR #59 OR #60  #62 MESH DESCRIPTOR Exercise EXPLODE ALL TREES  #63 MESH DESCRIPTOR Physical Education and Training EXPLODE ALL TREES  #64 MESH DESCRIPTOR Physical Exertion EXPLODE ALL TREES  #65 MESH DESCRIPTOR Sports EXPLODE ALL TREES  #66 MESH DESCRIPTOR Walking EXPLODE ALL TREES  #67 MESH DESCRIPTOR Exercise Therapy EXPLODE ALL TREES WITH QUALIFIERS MT  #68 (circuit training):TI,AB,KY  #69 cycling:TI,AB,KY  #70 danc*:TI,AB,KY  #71 exercise*:TI,AB,KY  #72 kinesiotherap*:TI,AB,KY  #73 running:TI,AB,KY  #74 squat*:TI,AB,KY  #75 strengthen*:TI,AB,KY  #76 swim*:TI,AB,KY  #77 treadmill:TI,AB,KY  #78 walk*:TI,AB,KY  #79 ((exercise* adj (activit* or Endurance or intervention* or program* or protocol* or regim* or therap* or treatment or fitness or strength or aerobic))):TI,AB,KY  #80 ((fitness adj (activit* or Endurance or intervention* or program* or protocol* or regim* or therap* or treatment or fitness or strength or aerobic))):TI,AB,KY  #81 ((physical adj (activit* or Endurance or intervention* or program* or protocol* or regim* or therap* or treatment or fitness or strength or aerobic))):TI,AB,KY  #82 #62 OR #63 OR #64 OR #65 OR #66 OR #67 OR #68 OR #69 OR #70 OR #71 OR #72 OR #73 OR #74 OR #75 OR #76 OR #77 OR #78 OR #79 OR #80 OR #81  #83 #61 AND #82  #84 01/01/2016 TO 31/01/2024:CD  #85 #83 AND #84 | Feb 2024: 18118   Jan 2025: 1596 |
| 2. MEDLINE In-process and other non-indexed citations and MEDLINE 1950-present (Ovid) | 1 "Intracranial Embolism and Thrombosis"/rh, th [Rehabilitation, Therapy]  2 Angina Pectoris/rh, th [Rehabilitation, Therapy]  3 Angina, Stable/rh, th [Rehabilitation, Therapy]  4 exp Angioplasty/rh, th [Rehabilitation, Therapy]  5 Arterial Occlusive Diseases/rh, th [Rehabilitation, Therapy]  6 Arteriolosclerosis/  7 exp Arteriosclerosis/  8 Arteriosclerosis Obliterans/  9 Atherectomy/  10 Atherosclerosis/  11 Basal Ganglia Cerebrovascular Disease/  12 exp Brain Infarction/  13 Brain Ischemia/  14 exp Carotid Artery Diseases/  15 Coronary Artery Bypass/  16 exp Coronary Disease/rh, th [Rehabilitation, Therapy]  17 exp Heart Failure/rh, th [Rehabilitation, Therapy]  18 Hemiplegia/  19 exp Intermittent Claudication/rh, th [Rehabilitation, Therapy]  20 exp Intracranial Arterial Diseases/  21 exp Intracranial Arteriovenous Malformations/  22 exp Intracranial Hemorrhages/  23 exp Myocardial Infarction/rh, th [Rehabilitation, Therapy]  24 Myocardial Ischemia/rh, th [Rehabilitation, Therapy]  25 exp Myocardial Revascularization/  26 Percutaneous Coronary Intervention/  27 exp Peripheral Vascular Diseases/rh, th [Rehabilitation, Therapy]  28 exp Stroke/pp, th [Physiopathology, Therapy]  29 exp Stroke Rehabilitation/mt [Methods]  30 Stroke, Lacunar/rh, th [Rehabilitation, Therapy]  31 Vasospasm, Intracranial/  32 Vertebral Artery Dissection/  33 "acute coronary syndrom*".ti,ab.  34 "angor pectoris".ti,ab.  35 "brain vasc*".ti,ab.  36 "cerebral vasc*".ti,ab.  37 "coronary heart disease".ti,ab.  38 "endoluminal repair*".ti,ab.  39 "heart failure".ti,ab.  40 "intermittent claudication".ti,ab.  41 "post‐stroke".ti,ab.  42 angina.ti,ab.  43 angioplast*.ti,ab.  44 apoplex*.ti,ab.  45 arteriosclero*.ti,ab.  46 atherectom*.ti,ab.  47 atherosclero*.ti,ab.  48 cerebrovasc*.ti,ab.  49 "intermittent claudic*".ti,ab.  50 hemipleg*.ti,ab.  51 hemipar*.ti,ab.  52 poststroke.ti,ab.  53 stenocardia*.ti,ab.  54 stroke.ti,ab.  55 ((brain* or cerebr* or cerebell*or intracran* or intracerebral) adj (ischemi* or ischaemi* or infarct* or thrombo* or emboli* or occlus*)).ti,ab.  56 ((heart or myocard* or coronary) adj (ischemi* or ischaemi* or infarct* or failure or infarct*)).ti,ab.  57 (coronary adj (disease* or bypass or thrombo* or angioplast*)).ti,ab.  58 (percutaneous coronary adj (interven* or revascular*)).ti,ab.  59 ((arter* or vascular or vein* or veno* or peripher*) adj (occlus* or reocclus* or steno* or obstruct* or lesio* or block* or harden* or stiffen*)).ti,ab.  60 (peripheral adj2 dis*).ti,ab.  61 or/1-60  62 exp Exercise/  63 exp "Physical Education and Training"/  64 exp Physical Exertion/  65 exp Sports/  66 exp Walking/  67 exp Exercise Therapy/mt [Methods]  68 "circuit training".ti,ab.  69 cycling.ti,ab.  70 danc*.ti,ab.  71 exercise*.ti,ab.  72 kinesiotherap*.ti,ab.  73 running.ti,ab.  74 squat*.ti,ab.  75 strengthen*.ti,ab.  76 swim*.ti,ab.  77 training.ti,ab.  78 treadmill.ti,ab.  79 walk*.ti,ab.  80 (exercise* adj (activit* or Endurance or intervention* or program* or protocol* or regim* or therap* or treatment or fitness or strength or aerobic)).ti,ab.  81 (fitness adj (activit* or Endurance or intervention* or program* or protocol* or regim* or therap* or treatment or fitness or strength or aerobic)).ti,ab.  82 (physical adj (activit* or Endurance or intervention* or program* or protocol* or regim* or therap* or treatment or fitness or strength or aerobic)).ti,ab.  83 or/62-82  84 61 and 83  85 randomized controlled trial.pt.  86 controlled clinical trial.pt.  87 randomized.ab.  88 placebo.ab.  89 drug therapy.fs.  90 randomly.ab.  91 trial.ab.  92 groups.ab.  93 or/85-92  94 exp animals/ not humans.sh.  95 93 not 94  96 84 and 95  97 (2016* or 2017* or 2018* or 2019* or 2020* or 2021* or 2022* or 2023*).ed.  98 96 and 97 | Feb 2024: 10625  Jan 2025: 1660 |
| 3. EMBASE (OVID)  1974 to present | 1 "Intracranial Embolism and Thrombosis"/rh, th  2 Angina Pectoris/rh, th [Rehabilitation, Therapy]  3 Angina, Stable/rh, th [Rehabilitation, Therapy]  4 exp Angioplasty/rh, th [Rehabilitation, Therapy]  5 arteriolosclerosis/  6 arteriosclerosis/rh, th [Rehabilitation, Therapy]  7 arteriosclerosis obliterans/  8 atherectomy/rh [Rehabilitation]  9 atherosclerosis/rh, th [Rehabilitation, Therapy]  10 basal ganglion hemorrhage/  11 exp brain infarction/rh, th [Rehabilitation, Therapy]  12 exp brain ischemia/rh, th [Rehabilitation, Therapy]  13 exp carotid artery disease/rh, th [Rehabilitation, Therapy]  14 exp coronary artery disease/rh, th [Rehabilitation, Therapy]  15 exp heart failure/rh, th [Rehabilitation, Therapy]  16 hemiplegia/rh, th [Rehabilitation, Therapy]  17 exp intermittent claudication/rh, th [Rehabilitation, Therapy]  18 exp cerebral artery disease/rh, th [Rehabilitation, Therapy]  19 exp brain arteriovenous malformation/rh, th [Rehabilitation, Therapy]  20 exp brain hemorrhage/rh, th [Rehabilitation, Therapy]  21 exp heart infarction/rh, th [Rehabilitation, Therapy]  22 exp heart muscle ischemia/rh, th [Rehabilitation, Therapy]  23 exp heart muscle revascularization/rh [Rehabilitation]  24 exp percutaneous coronary intervention/rh [Rehabilitation]  25 exp peripheral vascular disease/rh, th [Rehabilitation, Therapy]  26 exp peripheral vascular disease/rh, th [Rehabilitation, Therapy]  27 exp cerebrovascular accident/rh, th [Rehabilitation, Therapy]  28 exp stroke rehabilitation/  29 exp lacunar stroke/rh, th [Rehabilitation, Therapy]  30 exp brain vasospasm/rh, th [Rehabilitation, Therapy]  31 exp vertebral artery dissection/th [Therapy]  32 "acute coronary syndrom*".ti,ab.  33 "angor pectoris".ti,ab.  34 "brain vasc*".ti,ab.  35 "cerebral vasc*".ti,ab.  36 "coronary heart disease".ti,ab.  37 "endoluminal repair*".ti,ab.  38 "heart failure".ti,ab.  39 "intermittent claudication".ti,ab.  40 "post‐stroke".ti,ab.  41 angina.ti,ab.  42 angioplast*.ti,ab.  43 apoplex*.ti,ab.  44 arteriosclero*.ti,ab.  45 atherectom*.ti,ab.  46 atherosclero*.ti,ab.  47 cerebrovasc*.ti,ab.  48 "intermittent claudic*".ti,ab.  49 hemipleg*.ti,ab.  50 hempar*.ti,ab.  51 poststroke.ti,ab.  52 stenocardia*.ti,ab.  53 stroke.ti,ab.  54 ((brain* or cerebr* or cerebell*or intracran* or intracerebral) adj (ischemi* or ischaemi* or infarct* or thrombo* or emboli* or occlus*)).ti,ab.  55 ((heart or myocard* or coronary) adj (ischemi* or ischaemi* or infarct* or failure or infarct*)).ti,ab.  56 (coronary adj (disease* or bypass or thrombo* or angioplast*)).ti,ab.  57 (percutaneous coronary adj (interven* or revascular*)).ti,ab.  58 ((arter* or vascular or vein* or veno* or peripher*) adj (occlus* or reocclus* or steno* or obstruct* or lesio* or block* or harden* or stiffen*)).ti,ab.  59 (peripheral adj2 dis*).ti,ab.  60 1 or 2 or 3 or 4 or 5 or 6 or 7 or 8 or 9 or 10 or 11 or 12 or 13 or 14 or 15 or 16 or 17 or 18 or 19 or 20 or 21 or 22 or 23 or 24 or 25 or 26 or 27 or 28 or 29 or 30 or 31 or 32 or 33 or 34 or 35 or 36 or 37 or 38 or 39 or 40 or 41 or 42 or 43 or 44 or 45 or 46 or 47 or 48 or 49 or 50 or 51 or 52 or 53 or 54 or 55 or 56 or 57 or 58 or 59  61 exp exercise/  62 exp physical education/  63 exp sport/  64 exp walking/  65 exp kinesiotherapy/  66 "circuit training".ti,ab.  67 cycling.ti,ab.  68 danc*.ti,ab.  69 exercise*.ti,ab.  70 kinesiotherap*.ti,ab.  71 running.ti,ab.  72 squat*.ti,ab.  73 strengthen*.ti,ab.  74 swim*.ti,ab.  75 treadmill.ti,ab.  76 walk*.ti,ab.  77 (exercise* adj (activit* or Endurance or intervention* or program* or protocol* or regim* or therap* or treatment or fitness or strength or aerobic)).ti,ab.  78 (fitness adj (activit* or Endurance or intervention* or program* or protocol* or regim* or therap* or treatment or fitness or strength or aerobic)).ti,ab.  79 (physical adj (activit* or Endurance or intervention* or program* or protocol* or regim* or therap* or treatment or fitness or strength or aerobic)).ti,ab.  80 or/61-79  81 60 and 80  82 randomized controlled trial/  83 controlled clinical trial/  84 random$.ti,ab.  85 randomization/  86 intermethod comparison/  87 placebo.ti,ab.  88 (compare or compared or comparison).ti.  89 ((evaluated or evaluate or evaluating or assessed or assess) and (compare or compared or comparing or comparison)).ab.  90 (open adj label).ti,ab.  91 ((double or single or doubly or singly) adj (blind or blinded or blindly)).ti,ab.  92 double blind procedure/  93 parallel group$1.ti,ab.  94 (crossover or cross over).ti,ab.  95 ((assign$ or match or matched or allocation) adj5 (alternate or group$1 or intervention$1 or patient$1 or subject$1 or participant$1)).ti,ab.  96 (assigned or allocated).ti,ab.  97 (controlled adj7 (study or design or trial)).ti,ab.  98 (volunteer or volunteers).ti,ab.  99 trial.ti.  100 or/82-99  101 81 and 100  102 (2016* or 2017* or 2016* or 2017* or 2018* or 2019* or 2020* or 2021* or 2022* or 2023* or 2024*).dc.  103 101 and 102 | Feb 2024: 19163  Jan 2025: 2922 |
| 4. CINAHL (EBSCOhost) | S90 S88 AND S89  S89 EM 2016 OR EM 2017 OR EM 2018 OR EM 2019 OR EM 2020 OR EM 2021 OR EM 2022 OR EM 2023 OR EM 2024  S88 S85 AND S86 AND S87  S87 S73 OR S74 OR S75 OR S76 OR S77 OR S78 OR S79 OR S80 OR S81 OR S82 OR S83 OR S84  S86 S55 OR S56 OR S57 OR S58 OR S59 OR S60 OR S61 OR S62 OR S63 OR S64 OR S65 OR S66 OR S67 OR S68 OR S69 OR S70 OR S71 OR S72  S85 S1 OR S2 OR S3 OR S4 OR S5 OR S6 OR S7 OR S8 OR S9 OR S10 OR S11 OR S12 OR S13 OR S14 OR S15 OR S16 OR S17 OR S18 OR S19 OR S20 OR S21 OR S22 OR S23 OR S24 OR S25 OR S26 OR S27 OR S28 OR S29 OR S30 OR S31 OR S32 OR S33 OR S34 OR S35 OR S36 OR S37 OR S38 OR S39 OR S40 OR S41 OR S42 OR S43 OR S44 OR S45 OR S46 OR S47 OR S48 OR S49 OR S50 OR S51 OR S52 OR S53 OR S54  S84 MH "Random Assignment"  S83 MH "Single-Blind Studies" or MH "Double-Blind Studies" or MH "Triple-Blind Studies"  S82 MH "Crossover Design"  S81 MH "Factorial Design"  S80 MH "Placebos"  S79 MH "Clinical Trials"  S78 TX "multi-centre study" OR "multi-center study" OR "multicentre study" OR "multicenter study" OR "multi-site study"  S77 TX crossover OR "cross-over"  S76 AB placebo*  S75 TX random*  S74 TX trial*  S73 TX "latin square"  S72 TX physical N (activit* or Endurance or intervention* or program* or protocol* or regim* or therap* or treatment or fitness or strength or aerobic)  S71 TX fitness N (activit* or Endurance or intervention* or program* or protocol* or regim* or therap* or treatment or fitness or strength or aerobic)  S70 TX exercise* N (activit* or Endurance or intervention* or program* or protocol* or regim* or therap* or treatment or fitness or strength or aerobic)  S69 TX walk*  S68 TX treadmill  S67 TX swim*  S66 TX squat*  S65 TX running  S64 TX kinesiotherap*  S63 TX exercise*  S62 TX danc*  S61 TX cycling  S60 TX "circuit training"  S59 (MH "Therapeutic Exercise+/MT")  S58 (MH "Walking+")  S57 (MH "Sports+")  S56 (MH "Physical Education and Training+")  S55 (MH "Exercise+")  S54 TX peripheral N2 dis*  S53 TX (arter* or vascular or vein* or veno* or peripher*) N (occlus* or reocclus* or steno* or obstruct* or lesio* or block*)  S52 TX "percutaneous coronary" N (interven* or revascular*)  S51 TX coronary N (disease* or bypass or thrombo* or angioplast*)  S50 TX (heart or myocard* or coronary) N (ischemi* or ischaemi* or infarct* or failure or infarct*)  S49 TX (brain* or cerebr* or cerebell*or intracran* or intracerebral) N (ischemi* or ischaemi* or infarct* or thrombo* or emboli* or occlus*)  S48 TX stroke  S47 TX stenocardia*  S46 TX poststroke  S45 TX hemipar*  S44 TX hemipleg*  S43 TX "intermittent claudic*"  S42 TX cerebrovasc*  S41 TX atherosclero*  S40 TX atherectom*  S39 TX arteriosclero*  S38 TX apoplex*  S37 TX angioplast*  S36 TX angina  S35 TX "post‐stroke"  S34 TX "intermittent claudication"  S33 TX "heart failure"  S32 TX "endoluminal repair*"  S31 TX "coronary heart disease"  S30 TX "cerebral vasc*"  S29 TX "brain vasc*"  S28 TX "angor pectoris"  S27 TX "acute coronary syndrom*"  S26 (MH "Vertebral Artery Dissections/TH/RH")  S25 (MH "Stroke, Lacunar/TH/RH")  S24 (MH "Stroke+/RH/TH")  S23 (MH "Peripheral Vascular Diseases+/RH/TH")  S22 (MH "Percutaneous Coronary Intervention/TH/RH")  S21 (MH "Myocardial Revascularization+/TH/RH")  S20 (MH "Myocardial Ischemia+/TH/RH")  S19 (MH "Myocardial Infarction+/RH/TH")  S18 (MH "Intracranial Hemorrhage+/TH/RH")  S17 (MH "Intracranial Arterial Diseases+/TH/RH")  S16 (MH "Intermittent Claudication/RH/TH")  S15 (MH "Hemiplegia/TH/RH")  S14 (MH "Heart Failure+/RH/TH")  S13 (MH "Coronary Disease+/TH/RH")  S12 (MH "Coronary Artery Bypass+/TH/RH")  S11 (MH "Carotid Artery Diseases+/TH/RH")  S10 (MH "Cerebral Ischemia+/TH/RH")  S9 (MH "Basal Ganglia Cerebrovascular Disease+")  S8 (MH "Atherosclerosis/TH/RH")  S7 (MH "Atherectomy/")  S6 (MH "Arteriosclerosis/RH/TH")  S5 (MH "Arterial Occlusive Diseases/RH/TH")  S4 (MH "Angioplasty/RH")  S3 (MH "Angina, Stable/RH/TH")  S2 (MH "Angina Pectoris/RH/TH")  S1 (MH "Intracranial Embolism and Thrombosis/RH/TH") | Feb 2024: 4558  Jan 2025: 490 |
| 5. ClinicalTrials.gov  ([www.clinicaltrials.gov](http://www.clinicaltrials.gov/)) | Stroke OR “Transient ischemic attack” OR TIA OR “coronary artery disease” OR “Stroke OR “Transient ischemic attack” OR TIA OR “coronary artery disease” OR “stable angina” OR “acute coronary syndrome” OR “coronary revascularisation” OR “heart failure” | Exercise OR “Physical Education and Training“ OR “Physical Exertion” OR Sports OR Walking OR “resistance training” OR “power training” OR “cardiorespiratory training” OR “mixed training” | Start date on or after 01/01/2016 | Last update posted on or before 02/12/2024 | Feb 2024: 1782  Jan 2025: 359 |
| 6. ICTRP | Stroke OR “Transient ischemic attack” OR TIA OR “coronary artery disease” OR “Stroke OR “Transient ischemic attack” OR TIA OR “coronary artery disease” OR “stable angina” OR “acute coronary syndrome” OR “coronary revascularisation” OR “heart failure” | Exercise OR “Physical Education and Training“ OR “Physical Exertion” OR Sports OR Walking OR “resistance training” OR “power training” OR “cardiorespiratory training” OR “mixed training” | Start date on or after 01/01/2016 | Last update posted on or before 02/12/2024 | Feb 2024: 976  Jan 2025: 160 |

# S3: Supplementary Table 1. Table of excluded studies with reasons

Ongoing studies references list available on request

| **Study ID** | **Reference** | | | | | | | **Exclusion reason** |
| --- | --- | --- | --- | --- | --- | --- | --- | --- |
|  | **Authors** | **Title** | **Journal** | **Year** | **Vol** | **Issue** | **Pages** |  |
| Hambrecht 2004 | Hambrecht, R.; Walther, C.; Möbius-Winkler, S.; Gielen, S.; Linke, A.; Conradi, K.; et al.  disease: a randomized trial. | Percutaneous coronary angioplasty compared with exercise training in patients with stable coronary artery disease: a randomized trial | Circulation | 2004 | 109 | 11 | 1371-8 | Wrong comparator - active control |
| Jiang 2007 | Jiang, X.; Sit, J. W.; Wong, T. K. | A nurse-led cardiac rehabilitation programme improves health behaviours and cardiac physiological risk parameters: evidence from Chengdu, China | Journal of Clinical Nursing | 2007 | 16 | 10 | 1886-97 | Wrong comparator - co-interventions not balanced between arms |
| Manchanda 2000 | Manchanda, S.; Narang R.; Reddy K.; Sachdeva U.; Prabhakaran D.; Dharmanand S.; Rajani MBijlani R. | Retardation of coronary atherosclerosis with yoga lifestyle intervention | Journal of the Association of Physicians of India | 2000 | 48 | 7 | 687-94 | Wrong comparator - co-interventions not balanced between arms |
| Niebauer 1995 | Niebauer, J.; Hambrecht, R.; Marburger, C.; Hauer, K.; Velich, T.; von Hodenberg, E.; Schlierf, G.; Kübler, W.; Schuler, G. | Impact of intensive physical exercise and low-fat diet on collateral vessel formation in stable angina pectoris and angiographically confirmed coronary artery disease | American Journal of Cardiology | 1995 | 76 | 11 | 771-5 | Wrong comparator - co-interventions not balanced between arms |
| Schuler 1992 | Schuler, G.; Hambrecht, R.; Schlierf, G.; Niebauer, J.; Hauer, K.; Neumann, J.; Hoberg, E.; Drinkmann, A.; Bacher, F.; Grunze, M. | Regular physical exercise and low-fat diet. Effects on progression of coronary artery disease | Circulation | 1992 | 86 | 1 | 1-11 | Wrong comparator - co-interventions not balanced between arms |
| WHO 1983 | World Health Organization Regional Office for Europe Copenhagen | Rehabilitation and comprehensive secondary prevention after acute myocardial infaction | EURO Reports and Sutdies | 1983 | 84 |  |  | Wrong intervention - does not meet exercise definition |
| VHSG 2003 | The Vestfold Heartcare Study Group | Influence on lifestyle measures and five-year coronary risk by a comprehensive lifestyle intervention programme in patients with coronary heart disease | European Journal of Cardiovascular Prevention and Rehabilitation | 2003 | 10 | 6 | 429-37 | Wrong comparator - co-interventions not balanced between arms |
| Dorje 2019 | Dorje, T.; Zhao, G.; Tso, K.; Wang, J.; Chen, Y.; Tsokey, L.; Tan, B. K.; Scheer, A.; Jacques, A.; Li, Z.; et al. | Correction to Smartphone and social media-based cardiac rehabilitation and secondary prevention in China (SMART-CR/SP): a parallel-group, single-blind, randomised controlled trial (The Lancet Digital Health (2019) 1(7) (e363-“e374), (S2589750019301517), (10.1016/S2589-7500(19)30151-7)) | The Lancet Digital Health | 2020 | 2 | 1 | e15 | Wrong comparator - co-interventions not balanced between arms |
| Andersen 1981 | Andersen, G. S.; Christiansen, P.; Madsen, S.; Schmidt, G. | Value of regular supervised physical training after acute myocardial infarction | Ugeskrift for laeger | 1981 | 143 | 45 | 2952-5 | Full text not in English |
| Aronov 2010 | Aronov, D. M.; Krasnitskij, V. B.; Bubnova, M. G. | Efficacy of physical training and analysis of lipid-lowering therapy in patients with ischemic heart disease after acute coronary incidents | Rational Pharmacotherapy Cardiology | 2010 | 6 | 1 | 9-19 | Wrong study design - not RCT |
| Bengtsson 1983 | Bengtsson, K. | Rehabilitation after myocardial infarction. A controlled study | Scandinavian Journal of Rehabilitation Medicine | 1983 | 15 | 1 | 1-9 | Wrong comparator - co-interventions not balanced between arms |
| Bettencourt 2005 | Bettencourt, N.; Dias, C.; Mateus, P.; Sampaio, F.; Santos, L.; Adao, L.; Mateus, C.; Salomé, N.; Miranda, F.; Teixeira, M.; et al., | Impact of cardiac rehabilitation on quality of life and depression after acute coronary syndrome | Revista portuguesa de Cardiologia [Portuguese Journal of Cardiology] | 2005 | 24 | 5 | 687-96 | Full text not in English |
| Briffa 2005 | Briffa, T. G.; Eckermann, S. D.; Griffiths, A. D.; Harris, P. J.; Heath, M. R.; Freedman, S. B.; Donaldson, L. T.; Briffa, N. K.; Keech, A. C. | Cost-effectiveness of rehabilitation after an acute coronary event: a randomised controlled trial | Medical Journal of Australia | 2005 | 183 | 9 | 450-55 | Wrong comparator - co-interventions not balanced between arms |
| Bubnova 2019 | Bubnova, M. G.; Aronov, D. M. | Clinical effects of a one-year cardiac rehabilitation program using physical training after myocardial infarction in patients of working age with different rehabilitation potentials | Cardiovascular Therapy and Prevention (russian federation) | 2019 | 18 | 5 | 27-37 | Full text not in English |
| Bubnova 2020 | Bubnova, M. G.; Aronov, D. M. | Physical rehabilitation after acute myocardial infarction: focus on body weight | Russian Journal of Cardiology | 2020 | 25 | 5 | 3867 | Full text not in English |
| Carlsson 1998 | Carlsson, R. | Serum cholesterol, lifestyle, working capacity and quality of life in patients with coronary artery disease. Experiences from a hospital-based secondary prevention programme | Scandinavian Cardiovascular Journal | 1998 | 50 |  | 1-20 | Wrong comparator - co-interventions not balanced between arms |
| Chandrasekaran 2019 | Chandrasekaran, A. M.; Kinra, S.; Ajay, V. S.; Chattopadhyay, K.; Singh, K.; Singh, K.; Praveen, P. A.; Soni, D.; Devarajan, R.; Kondal, D.; et al., | Effectiveness and cost-effectiveness of a Yoga-based Cardiac Rehabilitation (Yoga-CaRe) program following acute myocardial infarction: study rationale and design of a multi-center randomized controlled trial | International Journal of Cardiology | 2019 | 280 |  | 14-18 | Wrong comparator - co-interventions not balanced between arms |
| Dorje 2018 | Dorje, T.; Zhao, G.; Scheer, A.; Tsokey, L.; Wang, J.; Chen, Y.; Tso, K.; Tan, B. K.; Ge, J.; Maiorana, A. | SMARTphone and social media-based Cardiac Rehabilitation and Secondary Prevention (SMART-CR/SP) for patients with coronary heart disease in China: a randomised controlled trial protocol | BMJ Open | 2018 | 8 | 6 |  | Wrong intervention - does not meet exercise definition |
| Dorje 2019 | Dorje, T.; Zhao, G.; Tso, K.; Wang, J.; Chen, Y.; Tsokey, L.; Tan, B. K.; Scheer, A.; Jacques, A.; Li, Z.; et al., | Smartphone and social media-based cardiac rehabilitation and secondary prevention in China (SMART-CR/SP): a parallel-group, single-blind, randomised controlled trial | The lancet. Digital health | 2019 | 1 | 7 | e363-e374 | Wrong comparator - co-interventions not balanced between arms |
| Engblom 1992 | Engblom, E.; Hämäläinen, H.; Lind, J.; Mattlar, C. E.; Ollila, S.; Kallio, V.; Inberg, M.; Knuts, L. R. | Quality of life during rehabilitation after coronary artery bypass surgery | Quality of Life Research | 1992 | 1 | 3 | 167-75 | Wrong intervention < 6 weeks |
| Engblom 1997 | Engblom, E.; Korpilahti, K.; Hämäläinen, H.; Rönnemaa, T.; Puukka, P. | Quality of life and return to work 5 years after coronary artery bypass surgery. Long-term results of cardiac rehabilitation | Journal of Cardiopulmonary Rehabilitation | 1997 | 17 | 1 | 29-36 | Wrong intervention < 6 weeks |
| Fridlund 1991 | Fridlund, B.; Högstedt, B.; Lidell, E.; Larsson, P. A. | Recovery after myocardial infarction. Effects of a caring rehabilitation programme | Scandinavian Journal of Caring Sciences | 1991 | 5 | 1 | 23-32 | Wrong comparator - co-interventions not balanced between arms |
| Giallauria 2008 | Giallauria, F.; Cirillo, P.; Lucci, R.; Pacileo, M.; De Lorenzo, A.; D'Agostino, M.; Moschella, S.; Psaroudaki, M.; Del Forno, D.; Orio, F.; et al., | Left ventricular remodelling in patients with moderate systolic dysfunction after myocardial infarction: favourable effects of exercise training and predictive role of N-terminal pro-brain natriuretic peptide | European Journal of Cardiovascular Prevention and Rehabilitation | 2008 | 15 | 1 | 113-18 | Wrong comparator - co-interventions not balanced between arms |
| Hall 2002 | Hall, J. P.; Wiseman, V. L.; King, M. T.; Ross, D. L.; Kovoor, P.; Zecchin, R. P.; Moir, F. M.; Denniss, A. R. | Economic evaluation of a randomised trial of early return to normal activities versus cardiac rehabilitation after acute myocardial infarction | Heart lung and circulation | 2002 | 11 | 1 | 10-18 | Wrong intervention < 6 weeks |
| Hambrecht 1993 | Hambrecht, R.; Niebauer, J.; Marburger, C.; Grunze, M.; Kälberer, B.; Hauer, K.; Schlierf, G.; Kübler, W.; Schuler, G. | Various intensities of leisure time physical activity in patients with coronary artery disease: effects on cardiorespiratory fitness and progression of coronary atherosclerotic lesions | Journal of the American College of Cardiology | 1993 | 22 | 2 | 468-477 | Wrong comparator - co-interventions not balanced between arms |
| Haskell 1994 | Haskell, W. L.; Alderman, E. L.; Fair, J. M.; Maron, D. J.; Mackey, S. F.; Superko, H. R.; Williams, P. T.; Johnstone, I. M.; Champagne, M. A.; Krauss, R. M. | Effects of intensive multiple risk factor reduction on coronary atherosclerosis and Clinical cardiac events in men and women with coronary artery disease. The Stanford Coronary Risk Intervention Project (SCRIP) | Circulation | 1994 | 89 | 3 | 975-90 | Wrong comparator - co-interventions not balanced between arms |
| Heller 1993 | Heller, R. F.; Knapp, J. C.; Valenti, L. A.; Dobson, A. J. | Secondary prevention after acute myocardial infarction | American Journal of Cardiology | 1993 | 72 | 11 | 759-62 | Wrong intervention - does not meet exercise definition |
| Higgins 2001 | Higgins, H. C.; Hayes, R. L.; McKenna, K. T. | Rehabilitation outcomes following percutaneous coronary interventions (PCI) | Patient Education & Counseling | 2001 | 43 | 3 | 219-30 | Wrong comparator - co-interventions not balanced between arms |
| Hofman-Bang 1999 | Hofman-Bang, C.; Lisspers, J.; Nordlander, R.; Nygren, A.; Sundin, O.; Ohman, A.; Rydén, L. | Two-year results of a controlled study of residential rehabilitation for patients treated with percutaneous transluminal coronary angioplasty. A randomized study of a multifactorial programme | European Heart Journal | 1999 | 20 | 20 | 1465-74 | Wrong comparator - co-interventions not balanced between arms |
| Houle 2012 | Houle, J.; Doyon, O.; Vadeboncoeur, N.; Turbide, G.; Diaz, A.; Poirier, P. | Effectiveness of a pedometer-based program using a socio-cognitive intervention on physical activity and quality of life in a setting of cardiac rehabilitation | Canadian Journal of Cardiology | 2012 | 28 | 1 | 27-32 | Wrong comparator - co-interventions not balanced between arms |
| Kallio 1979 | Kallio, V.; Hamalainen, H.; Hakkila, J.; Luurila, O. J. | Reduction in sudden deaths by a multifactorial intervention program after acute myocardial infarction | Lancet | 1979 | 2 | 8152 | 1091-94 | Wrong comparator - co-interventions not balanced between arms |
| Kruse 2006 | Kruse, M.; Hochstrasser, S.; Zwisler, A. D.; Kjellberg, J. | Comprehensive cardiac rehabilitation: a cost assessment based on a randomized Clinical trial | International Journal of technology assessment in health care | 2006 | 22 | 4 | 478-483 | Wrong comparator - co-interventions not balanced between arms |
| Lear 2014 | Lear, S. A.; Singer, J.; Banner-Lukaris, D.; Horvat, D.; Park, J. E.; Bates, J.; Ignaszewski, A. | Randomized trial of a virtual cardiac rehabilitation program delivered at a distance via the internet | Circulation: Cardiovascular Quality and Outcomes | 2014 | 7 | 6 | 952-59 | Wrong intervention - does not meet exercise definition |
| Lear 2015 | Lear, S. A.; Singer, J.; Banner-Lukaris, D.; Horvat, D.; Park, J. E.; Bates, J.; Ignaszewski, A. | Improving access to cardiac rehabilitation using the internet: a randomized trial | Studies in health technology and informatics | 2015 | 209 |  | 58-66 | Wrong intervention - does not meet exercise definition |
| Lewin 1992 | Lewin, B.; Robertson, I. H.; Cay, E. L.; Irving, J. B.; Campbell, M. | Effects of self-help post-myocardial-infarction rehabilitation on psychological adjustment and use of health services | Lancet | 1992 | 339 | 8800 | 1036-40 | Wrong intervention - does not meet exercise definition |
| Lidell 1996 | Lidell, E.; Fridlund, B. | Long-term effects of a comprehensive rehabilitation programme after myocardial infarction | Scandinavian Journal of Caring Sciences | 1996 | 10 | 2 | 67-74 | Wrong comparator - co-interventions not balanced between arms |
| Lisspers 1999 | Lisspers, J.; Sundin, O.; Hofman-Bang, C.; Nordlander, R.; Nygren, A.; Rydén, L.; Ohman, A. | Behavioral effects of a comprehensive, multifactorial program for lifestyle change after percutaneous transluminal coronary angioplasty: a prospective, randomized controlled study | Journal of psychosomatic Research | 1999 | 46 | 2 | 143-154 | Wrong comparator - co-interventions not balanced between arms |
| Lisspers 2005 | Lisspers, J.; Sundin, O.; Ohman, A.; Hofman-Bang, C.; Rydén, L.; Nygren, A. | Long-term effects of lifestyle behavior change in coronary artery disease: effects on recurrent coronary events after percutaneous coronary intervention | Health Psychology | 2005 | 24 | 1 | 41-48 | Wrong comparator - co-interventions not balanced between arms |
| Ma 2020 | Ma, L.; Deng, L.; Yu, H. | The effects of a comprehensive rehabilitation and intensive education program on anxiety, depression, quality of life, and major adverse cardiac and cerebrovascular events in unprotected left main coronary artery disease patients who underwent coronary artery bypass grafting | Irish Journal of Medical Science | 2020 | 189 | 2 | 477-88 | Wrong comparator - co-interventions not balanced between arms |
| Marchionni 2003 | Marchionni, N.; Fattirolli, F.; Fumagalli, S.; Oldridge, N.; Del Lungo, F.; Morosi, L.; Burgisser, C.; Masotti, G. | Improved exercise tolerance and quality of life with cardiac rehabilitation of older patients after myocardial infarction: results of a randomized, controlled trial | Circulation | 2003 | 107 | 17 | 2201-6 | Wrong comparator - co-interventions not balanced between arms |
| MarotoMontero 2005 | Maroto Montero, J. M.; Ramirez, R. A.; Morales Duran, M. D.; de Pablo Zarzosa, C.; Abraira, V. | Rehabilitación cardí­aca en pacientes con infarto de miocardio. Resultados tras 10 años de seguimiento | Revista espanola de Cardiologia | 2005 | 58 | 10 | 1181-1187 | Wrong comparator - co-interventions not balanced between arms |
| Mutwalli 2012 | Mutwalli, H. A.; Fallows, S. J.; Arnous, A. A.; Zamzami, M. S. | Randomized controlled evaluation shows the effectiveness of a home-based cardiac rehabilitation program | Saudi Medical Journal | 2012 | 33 | 2 | 152-9 | Wrong comparator - co-interventions not balanced between arms |
| Niebauer 1997 | Niebauer, J.; Hambrecht, R.; Velich, T.; Hauer, K.; Marburger, C.; Kälberer, B.; Weiss, C.; von Hodenberg, E.; Schlierf, G.; Schuler, G.; et al., | Attenuated progression of coronary artery disease after 6 years of multifactorial risk intervention: role of physical exercise | Circulation | 1997 | 96 | 8 | 2534-2541 | Wrong comparator - co-interventions not balanced between arms |
| Niebauer 1996 | Niebauer, J.; Hambrecht, R.; Velich, T.; Marburger, C.; Hauer, K.; Kreuzer, J.; Zimmermann, R.; von Hodenberg, E.; Schlierf, G.; Schuler, G.; et al., | Predictive value of lipid profile for salutary coronary angiographic changes in patients on a low-fat diet and physical exercise program | American Journal of Cardiology | 1996 | 78 | 2 | 163-167 | Wrong comparator - co-interventions not balanced between arms |
| Nikolaus 1991 | Nikolaus, T.; Schlierf, G.; Vogel, G.; Schuler, G.; Wagner, I. | Treatment of coronary heart disease with diet and exercise--problems of compliance | Annals of nutrition & metabolism | 1991 | 35 | 1 | 1-7 | Wrong comparator - co-interventions not balanced between arms |
| Oerkild 2012 | Oerkild, B.; Frederiksen, M.; Hansen, J. F.; Prescott, E. | Home-based cardiac rehabilitation is an attractive alternative to no cardiac rehabilitation for elderly patients with coronary heart disease: results from a randomised Clinical trial | BMJ Open | 2012 | 2 | 6 | e001820 | Wrong comparator - co-interventions not balanced between arms |
| Oldridge 1993 | Oldridge, N.; Furlong, W.; Feeny, D.; Torrance, G.; Guyatt, G.; Crowe, J.; Jones, N. | Economic evaluation of cardiac rehabilitation soon after acute myocardial infarction | American Journal of Cardiology | 1993 | 72 | 2 | 154-161 | Wrong comparator - co-interventions not balanced between arms |
| Oldridge 1991 | Oldridge, N.; Guyatt, G.; Jones, N.; Crowe, J.; Singer, J.; Feeny, D.; McKelvie, R.; Runions, J.; Streiner, D.; Torrance, G. | Effects on quality of life with comprehensive rehabilitation after acute myocardial infarction | American Journal of Cardiology | 1991 | 67 | 13 | 1084-9 | Wrong comparator - co-interventions not balanced between arms |
| Oldridge 1995 | Oldridge, N.; Streiner, D.; Hoffmann, R.; Guyatt, G. | Profile of mood states and cardiac rehabilitation after acute myocardial infarction | Medicine and Science in sports and exercise | 1995 | 27 | 6 | 900-905 | Wrong comparator - co-interventions not balanced between arms |
| Ornish 1990 | Ornish, D.; Brown, S. E.; Scherwitz, L. W.; Billings, J. H.; Armstrong, W. T.; Ports, T. A.; McLanahan, S. M.; Kirkeeide, R. L.; Brand, R. J.; Gould, K. L. | Can lifestyle changes reverse coronary heart disease? The Lifestyle Heart Trial | Lancet | 1990 | 336 | 8708 | 129-133 | Wrong comparator - co-interventions not balanced between arms |
| Ornish 1998 | Ornish, D.; Scherwitz, L. W.; Billings, J. H.; Brown, S. E.; Gould, K. L.; Merritt, T. A.; Sparler, S.; Armstrong, W. T.; Ports, T. A.; Kirkeeide, R. L.; et al., | Intensive lifestyle changes for reversal of coronary heart disease | Jama | 1998 | 280 | 23 | 2001-2007 | Wrong comparator - co-interventions not balanced between arms |
| Pal 2013 | Pal, A.; Srivastava, N.; Narain, V. S.; Agrawal, G. G.; Rani, M. | Effect of yogic intervention on the autonomic nervous system in the patients with coronary artery disease: a randomized controlled trial | Eastern Mediterranean Health Journal | 2013 | 19 | 5 | 452-8 | Wrong intervention - does not meet exercise definition |
| Pomeshkina 2015 | Pomeshkina, S.; Loktionova, E.; Arkhipova, N.; Barbarash, O. | Home-based walking training and adherence to Medical therapy in patients undergoing coronary artery bypass grafting | European Heart Journal | 2015 | 36 |  | 634 | Full text not in English |
| Pomeshkina 2015 | Pomeshkina, S. A.; Loktionova, E. B.; Bezzubova, V. A.; Arkhipova, N. V.; Borovik, I.; Barbarash, O. L. | The comparative analysis of the influence of the supervised exercise training and home-based exercise training on the psychological status of the following coronary artery bypass grafting | Physiotherapy, and Exercise Therapy | 2017 | 94 | 6 | 10-7 | Full text not in English |
| Pomeshkina 2019 | Pomeshkina, S. A.; Barbarash, O. L.; Pomeshkin, E. V. | Exercise training and erectile dysfunction in patients after coronary artery bypass grafting | Therapeutic Archive | 2019 | 91 | 9 | 16-20 | Full text not in English |
| Prabhakaran 2020 | Prabhakaran, D.; Chandrasekaran, A. M.; Singh, K.; Mohan, B.; Chattopadhyay, K.; Chadha, D. S.; Negi, P. C.; Bhat, P.; Sadananda, K. S.; Ajay, V. S.; et al., | Yoga-Based Cardiac Rehabilitation After Acute Myocardial Infarction: a Randomized Trial | Journal of the American College of Cardiology | 2020 | 75 | 13 | 1551-61 | Wrong comparator - co-interventions not balanced between arms |
| Reid 2012 | Reid, D. R.; Morrin, L. I.; Beaton, L. J.; Papadakis, S.; Kocourek, J.; McDonnell, L. | Randomized trial of an internet-based computer-tailored expert system for physical activity in patients with heart disease | European Journal of Preventive Cardiology | 2012 | 19 | 6 | 1357-64 | Wrong comparator - co-interventions not balanced between arms |
| Schumacher 2006 | Schumacher, A.; Peersen, K.; Sommervoll, L.; Seljeflot, I.; Arnesen, H.; Otterstad, J. E. | Physical performance is associated with markers of vascular inflammation in patients with coronary heart disease | European Journal of Cardiovascular Prevention and Rehabilitation | 2006 | 13 | 3 | 356-362 | Wrong comparator - co-interventions not balanced between arms |
| Seki 2008 | Seki, E.; Watanabe, Y.; Shimada, K.; Sunayama, S.; Onishi, T.; Kawakami, K.; Sato, M.; Sato, H.; Mokuno, H.; Daida, H. | Effects of a phase III cardiac rehabilitation program on physical status and lipid profiles in elderly patients with coronary artery disease: juntendo Cardiac Rehabilitation Program (J-CARP) | Circulation Journal | 2008 | 72 | 8 | 1230-4 | Wrong comparator - co-interventions not balanced between arms |
| Seki 2003 | Seki, E.; Watanabe, Y.; Sunayama, S.; Iwama, Y.; Shimada, K.; Kawakami, K.; Sato, M.; Sato, H.; Mokuno, H.; Daida, H. | Effects of phase III cardiac rehabilitation programs on health-related quality of life in elderly patients with coronary artery disease: juntendo Cardiac Rehabilitation Program (J-CARP) | Circulation Journal | 2003 | 67 | 1 | 73-7 | Wrong comparator - co-interventions not balanced between arms |
| Taylor 1997 | Taylor, C. B.; Miller, N. H.; Smith, P. M.; DeBusk, R. F. | The effect of a home-based, case-managed, multifactorial risk-reduction program on reducing psychological distress in patients with cardiovascular disease | Journal of Cardiopulmonary Rehabilitation | 1997 | 17 | 3 | 157-162 | Wrong comparator - co-interventions not balanced between arms |
| Uddin 2020 | Uddin, J.; Joshi, V. L.; Moniruzzaman, M.; Karim, R.; Uddin, J.; Siraj, M.; Rashid, M. A.; Rossau, H. K.; Taylor, R. S.; Zwisler, A. D. | Effect of Home-Based Cardiac Rehabilitation in a Lower-Middle Income Country: RESULTS FROM A CONTROLLED TRIAL | Journal of Cardiopulmonary Rehabilitation and Prevention | 2020 | 40 | 1 | 29-34 | Wrong study design - not RCT |
| Vecchio 1981 | Vecchio, C.; Cobelli, F.; Opasich, C. | Early functional evaluation and physical rehabilitation in patients with wide myocardial infarction | Giornale italiano di Cardiologia | 1981 | 11 | 4 | 419-29 | Full text not in English |
| Vermeulen 1983 | Vermeulen, A.; Lie, K. I.; Durrer, D. | Effects of cardiac rehabilitation after myocardial infarction: changes in coronary risk factors and long-term prognosis | American Heart Journal | 1983 | 105 | 5 | 798-801 | Wrong comparator - co-interventions not balanced between arms |
| West 2012 | West, R. R.; Jones, D. A.; Henderson, A. H. | Rehabilitation after myocardial infarction trial (RAMIT): multi-centre randomised controlled trial of comprehensive cardiac rehabilitation in patients following acute myocardial infarction | Heart | 2012 | 98 | 8 | 637-644 | Wrong comparator - co-interventions not balanced between arms |
| Yu 2004 | Yu, C. M.; Lau, C. P.; Chau, J.; McGhee, S.; Kong, S. L.; Cheung, B. M.; Li, L. S. | A short course of cardiac rehabilitation program is highly cost effective in improving long-term quality of life in patients with recent myocardial infarction or percutaneous coronary intervention | Archives of Physical Medicine and Rehabilitation | 2004 | 85 | 12 | 1915-22 | Wrong comparator - co-interventions not balanced between arms |
| Yu 2004 | Yu, C. M.; Li, L. S.; Lam, M. F.; Siu, D. C.; Miu, R. K.; Lau, C. P. | Effect of a cardiac rehabilitation program on left ventricular diastolic function and its relationship to exercise capacity in patients with coronary heart disease: experience from a randomized, controlled study | American Heart Journal | 2004 | 147 | 5 | e24 | Wrong comparator - co-interventions not balanced between arms |
| Zwisler 2008 | Zwisler, A. D.; Soja, A. M.; Rasmussen, S.; Frederiksen, M.; Abedini, S.; Appel, J.; Rasmussen, H.; Gluud, C.; Iversen, L.; Sigurd, B.; et al., | Hospital-based comprehensive cardiac rehabilitation versus usual care among patients with congestive heart failure, ischemic heart disease, or high risk of ischemic heart disease: 12-month results of a randomized Clinical trial | American Heart Journal | 2008 | 155 | 6 | 1106-13 | Wrong comparator - co-interventions not balanced between arms |
| Austin 2005 | Austin, J.; Williams, R.; Ross, L.; Moseley, L.; Hutchison, S. | Randomised controlled trial of cardiac rehabilitation in elderly patients with heart failure | European Journal of Heart Failure | 2005 | 7 | 3 | 411-7 | Wrong comparator - co-interventions not balanced between arms |
| Austin 2009 | Austin, J.; Williams, W. R.; Hutchison, S. | Multidisciplinary management of elderly patients with chronic heart failure: five year outcome measures in death and survivor groups | European Journal of Cardiovascular Nursing: Journal of the Working Group on Cardiovascular Nursing of the European Society of Cardiology | 2009 | 8 | 1 | 34-9 | Wrong comparator - co-interventions not balanced between arms |
| Austin 2008 | Austin, J.; Williams, W. R.; Ross, L.; Hutchison, S. | Five-year follow-up findings from a randomized controlled trial of cardiac rehabilitation for heart failure | European Journal of Cardiovascular Prevention and Rehabilitation | 2008 | 15 | 2 | 162-7 | Wrong comparator - co-interventions not balanced between arms |
| Davidson 2010 | Davidson, P. M.; Cockburn, J.; Newton, P. J.; Webster, J. K.; Betihavas, V.; Howes, L.; Owensby, D. O. | Can a heart failure-specific cardiac rehabilitation program decrease hospitalizations and improve outcomes in high-risk patients? | European Journal of Cardiovascular Prevention and Rehabilitation | 2010 | 17 | 4 | 393-402 | Wrong comparator - co-interventions not balanced between arms |
| Du 2018 | Du, H.; Newton, P. J.; Budhathoki, C.; Everett, B.; Salamonson, Y.; Macdonald, P. S.; Davidson, P. M. | The Home-Heart-Walk study, a self-administered walk test on perceived physical functioning, and self-care behaviour in people with stable chronic heart failure: a randomized controlled trial | European Journal of Cardiovascular Nursing | 2018 | 17 | 3 | 235-245 | Wrong intervention - does not meet exercise definition |
| Eyre 2016 | Eyre, V.; Lang, C. C.; Smith, K.; Jolly, K.; Davis, R.; Hayward, C.; Wingham, J.; Abraham, C.; Green, C.; Warren, F. C.; et al., | Rehabilitation Enablement in Chronic Heart Failure-a facilitated self-care rehabilitation intervention in patients with heart failure with preserved ejection fraction (REACH-HFpEF) and their caregivers: rationale and protocol for a single-centre pilot randomised controlled trial | BMJ Open | 2016 | 6 | 10 | e012853 | Wrong comparator - co-interventions not balanced between arms |
| Giallauria 2006 | Giallauria, F.; De Lorenzo, A.; Pilerci, F.; Manakos, A.; Lucci, R.; Psaroudaki, M.; D'Agostino, M.; Del Forno, D.; Vigorito, C. | Reduction of N terminal-pro-brain (B-type) natriuretic peptide levels with exercise-based cardiac rehabilitation in patients with left ventricular dysfunction after myocardial infarction | European Journal of Cardiovascular Prevention and Rehabilitation | 2006 | 13 | 4 | 625-31 | Wrong comparator - co-interventions not balanced between arms |
| Lang 2018 | Lang, C. C.; Smith, K.; Wingham, J.; Eyre, V.; Greaves, C. J.; Warren, F. C.; Green, C.; Jolly, K.; Davis, R. C.; Doherty, P. J.; et al., | A randomised controlled trial of a facilitated home-based rehabilitation intervention in patients with heart failure with preserved ejection fraction and their caregivers: the REACH-HFpEF Pilot Study | BMJ Open | 2018 | 8 | 4 | e019649 | Wrong comparator - co-interventions not balanced between arms |
| Mehani 2013 | Mehani, S. H. M. | Correlation between changes in diastolic dysfunction and health-related quality of life after cardiac rehabilitation program in dilated cardiomyopathy | Journal of Advanced Research | 2013 | 4 |  | 189-200 | Wrong study design - not RCT |
| Myers 2000 | Myers, J.; Goebbels, U.; Dzeikan, G.; Froelicher, V.; Bremerich, J.; Mueller, P.; Buser, P.; Dubach, P. | Exercise training and myocardial remodeling in patients with reduced ventricular function: one-year follow-up with magnetic resonance imaging | American Heart Journal | 2000 | 139 | 2 Pt 1 | 252-61 | Wrong comparator - co-interventions not balanced between arms |
| Nilsson 2008 | Nilsson, B. B.; Westheim, A.; Risberg, M. A. | Long-term effects of a group-based high-intensity aerobic interval-training program in patients with chronic heart failure | American Journal of Cardiology | 2008 | 102 | 9 | 1220-4 | Wrong comparator - co-interventions not balanced between arms |
| Norman 2012 | Norman, J. F.; Pozehl, B. J.; Duncan, K. A.; Hertzog, M. A.; Krueger, S. K. | Effects of Exercise Training versus Attention on Plasma B-type Natriuretic Peptide, 6-Minute Walk Test and Quality of Life in Individuals with Heart Failure | Cardiopulmonary Physical Therapy Journal | 2012 | 23 |  | 19-25 | Wrong comparator - co-interventions not balanced between arms |
| Pozehl 2008 | Pozehl, B.; Duncan, K.; Hertzog, M. | The effects of exercise training on fatigue and dyspnea in heart failure | European Journal of Cardiovascular Nursing | 2008 | 7 | 2 | 127-32 | Wrong comparator - co-interventions not balanced between arms |
| Pozehl 2010 | Pozehl, B.; Duncan, K.; Hertzog, M.; Norman, J. F. | Heart Failure Exercise And Training Camp: effects of a multicomponent exercise training intervention in patients with heart failure | Heart & lung | 2010 | 39 | 6 Suppl | S1-13 | Not relevant |
| Witham 2012 | Witham, M. D.; Fulton, R. L.; Greig, C. A.; Johnston, D. W.; Lang, C. C.; van der Pol, M.; Boyers, D.; Struthers, A. D.; McMurdo, M. E. | Efficacy and cost of an exercise program for functionally impaired older patients with heart failure: a randomized controlled trial | Circulation: Heart Failure | 2012 | 5 | 2 | 209-16 | Wrong comparator - co-interventions not balanced between arms |
| Zwisler 2005 | Zwisler, A. D.; Schou, L.; Soja, A. M.; Brønnum-Hansen, H.; Gluud, C.; Iversen, L. | A randomized Clinical trial of hospital-based, comprehensive cardiac rehabilitation versus usual care for patients with congestive heart failure, ischemic heart disease, or high risk of ischemic heart disease (the DANREHAB trial) - design, intervention, and population | American Heart Journal | 2005 | 899 |  | e16 | Wrong comparator - co-interventions not balanced between arms |
| Ciuffetti 1994 | Ciuffetti, G.; Paltriccia, R.; Lombardini, R.; Lupattelli, G.; Pasqualini, L.; Mannarino, E. | Treating peripheral arterial occlusive disease: pentoxifylline vs exercise | International Angiology | 1994 | 13 | 1 | 33-9 | Wrong comparator - active control |
| Cucato 2013 | Cucato, G. G.; Chehuen Mda, R.; Costa, L. A.; Ritti-Dias, R. M.; Wolosker, N.; Saxton, J. M.; Forjaz, C. L. | Exercise prescription using the heart of claudication pain onset in patients with intermittent claudication | Clinics | 2013 | 68 | 7 | 974-8 | Wrong comparator - active control |
| Jansen 1991 | Jansen, T.; Weiss, T.; Amendt, K.; Hsu, E.; Hubsch-Muller, C.; Diehm, C. | Effect of a 2-year ambulatory vascular sports program on walking distance in claudication patients--a controlled study | Vasa Supplementum | 1991 | 33 |  | 175 | Full text not in English |
| McDermott 2013 | McDermott, M.; Criqui, M.; Domanchuk, K.; Ferrucci, L.; Guralnik, J.; Kibbe, M.; Liu, K.; Liao, Y.; Lloyd-Jones, D.; Spring, B.; et al., | A home-based exercise intervention significantly improves walking performance in peripheral arterial disease: one-year follow-up from a randomized controlled trial | Circulation | 2013 | 128 |  | A14789 | Wrong comparator - co-interventions not balanced between arms |
| McDermott 2014 | McDermott, M.; Criqui, M.; Domanchuk, K.; Guralnik, J.; Kibbe, M.; Liu, K.; Liao, Y.; Lloyd-Jones, D.; Spring, B.; Tian, L.; et al., | Home-based exercise improves walking speed and prevents mobility loss in peripheral artery disease: a randomized controlled trial | Circulation | 2014 | 130 |  | A11744 | Wrong comparator - co-interventions not balanced between arms |
| McDermott 2015 | McDermott, M. M.; Guralnik, J. M.; Criqui, M. H.; Ferrucci, L.; Liu, K.; Spring, B.; Tian, L.; Domanchuk, K.; Kibbe, M.; Zhao, L.; et al., | Unsupervised exercise and mobility loss in peripheral artery disease: a randomized controlled trial | Journal of the American Heart Association | 2015 | 4 | 5 |  | Wrong comparator - co-interventions not balanced between arms |
| McDermott 2014 | McDermott, M. M.; Guralnik, J. M.; Criqui, M. H.; Ferrucci, L.; Zhao, L.; Liu, K.; Domanchuk, K.; Spring, B.; Tian, L.; Kibbe, M.; et al., | Home-based walking exercise in peripheral artery disease: 12-month follow-up of the GOALS randomized trial | Journal of the American Heart Association | 2014 | 3 | 3 | e000711 | Wrong comparator - co-interventions not balanced between arms |
| McDermott 2013 | McDermott, M. M.; Liu, K.; Guralnik, J. M.; Criqui, M. H.; Spring, B.; Tian, L.; Domanchuk, K.; Ferrucci, L.; Lloyd-Jones, D.; Kibbe, M.; et al., | Home-based walking exercise intervention in peripheral artery disease: a randomized Clinical trial | Jama | 2013 | 310 | 1 | 57-65 | Wrong comparator - co-interventions not balanced between arms |
| Rejeski 2014 | Rejeski, W. J.; Spring, B.; Domanchuk, K.; Tao, H.; Tian, L.; Zhao, L.; McDermott, M. M. | A group-mediated, home-based physical activity intervention for patients with peripheral artery disease: effects on social and psychological function | Journal of Translational Medicine | 2014 | 12 | 29 | 1-8 | Wrong comparator - co-interventions not balanced between arms |
| Roitman 2010 | Roitman, J. L. | Treadmill exercise and resistance training in patients with peripheral arterial disease with and without in intermittent claudication: a randomized controlled trial | Journal of Cardiopulmonary Rehabilitation and Prevention | 2010 | 30 | 1 | 62 | Not relevant |
| Tisi 1997 | Tisi, P.; Shearman, C. | The impact of treatment of intermittent claudication on subjective health of the patient | Health trends 1998/99 | 1997 | 30 |  | 109-14 | Wrong intervention < 6 weeks |
| da Cunha 2001 | da Cunha, I. T.; Lim, P. A.; Qureshy, H.; Henson, H.; Monga, T.; Protas, E. J. | A comparison of regular rehabilitation with supported treadmill ambulation training for acute stroke patients | Journal of Rehabilitation Research and development | 2001 | 38 | 2 | 245-255 | Wrong intervention < 6 weeks |
| Dean 2016 | Dean, S.; Calitri, R.; Shepherd, A.; Hollands, L.; Poltawski, L.; James, M. | Community-based rehabilitation training after stroke (ReTrain): results of a pilot randomised control trial (RCT) | International Journal of Stroke | 2016 | 11 | 4 (Suppl 1) | 13 | Wrong comparator - co-interventions not balanced between arms |
| Dean 2016 | Dean, S. G.; Poltawski, L.; Forster, A.; Taylor, R. S.; Spencer, A.; James, M.; Allison, R.; Stevens, S.; Norris, M.; Shepherd, A. I.; et al., | Community-based Rehabilitation Training after stroke: protocol of a pilot randomised controlled trial (ReTrain) | BMJ Open | 2016 | 6 | 10 | e012375 | Wrong comparator - co-interventions not balanced between arms |
| Dean 2018 | Dean, S. G.; Poltawski, L.; Forster, A.; Taylor, R. S.; Spencer, A.; James, M.; Allison, R.; Stevens, S.; Norris, M.; Shepherd, A. I.; et al., | Community-based rehabilitation training after stroke: results of a pilot randomised controlled trial (ReTrain) investigating acceptability and feasibility | BMJ Open | 2018 | 8 | 2 | e018409 | Wrong comparator - co-interventions not balanced between arms |
| Duncan 1998 | Duncan, P.; Richards, L.; Wallace, D.; Stoker-Yates, J.; Pohl, P.; Luchies, C.; Ogle, A.; Studenski, S. | A randomized, controlled pilot study of a home-based exercise program for individuals with mild and moderate stroke | Stroke | 1998 | 29 | 10 | 2055-60 | Wrong comparator - active control |
| Eich 2003 | Eich, H. J.; Hesse, S.; Mach, H. | Aerobic endurance training of hemiparetic patients who are able to walk. Results of a prospective randomised study | Deutsche zeitschrift für sportmedizin | 2003 | 54 | 7/8 | S98 | Wrong comparator - co-interventions not balanced between arms |
| Eich 2004 | Eich, H. J.; Mach, H.; Werner, C.; Hesse, S. | Aerobic treadmill plus Bobath walking training improves walking in subacute stroke: a randomized controlled trial | Clinical Rehabilitation | 2004 | 18 | 6 | 640-51 | Wrong comparator - active control |
| Eich 2004 | Eich, H. J.; Parchmann, H.; Hesse, S.; Mach, H.; Werner, C. | Aerobic treadmill training plus physiotherapy improves walking ability in subacute stroke patients. A randomized controlled study | Neurologie und Rehabilitation | 2004 | 10 | 4 | 187-216 | Wrong comparator - co-interventions not balanced between arms |
| Fitch 2005 | Fitch, S.; Elkins, M.; Moseley, A. | Was CAP summary faithful? | Australian Journal of Physiotherapy | 2005 | 51 |  | 265-266 | Wrong comparator - active control |
| Globas 2012 | Globas, C.; Becker, C.; Cerny, J.; Lam, J. M.; Lindemann, U.; Forrester, L. W.; Macko, R. F.; Luft, A. R. | Chronic stroke survivors benefit from high-intensity aerobic treadmill exercise: a randomized control trial | NeuroRehabilitation and Neural Repair | 2012 | 26 | 1 | 85-95 | Wrong comparator - active control |
| Hesse 2005 | Hesse, S.; Eich, H. J.; Mach, H.; Parchmann, H.; Werner, C. | Aerobic treadmill training plus physiotherapy improves walking speed and capacity in subacute, moderately affected patients after stroke | Neurologie und Rehabilitation | 2005 | 11 | 1 | 7-12 | Wrong comparator - active control |
| Hollands 2016 | Hollands, H.; Calitri, R.; Shepherd, A.; Poltawski, L.; Norris, M.; Taylor, R. | The ReTrain trial: evaluating intervention fidelity via video analysis of the independently getting up off the floor (IGO) technique | International Journal of Stroke | 2016 | 11 | (4 Suppl 1) | 58-59 | Wrong study design - not RCT |
| Inaba 1973 | Inaba, M.; Edberg, E.; Montgomery, J.; Gillis, M. K. | Effectiveness of functional training, active exercise, and resistive exercise for patients with hemiplegia | Physical Therapy | 1973 | 53 | 1 | 28-35 | Wrong intervention < 6 weeks |
| Ivey 2010 | Ivey, F. M.; Hafer-Macko, C. E.; Ryan, A. S.; Macko, R. F. | Impaired leg vasodilatory function after stroke: adaptations with treadmill exercise training | Stroke | 2010 | 41 | 12 | 2913-2917 | Wrong comparator - active control |
| Ivey 2017 | Ivey, F. M.; Prior, S. J.; Hafer-Macko, C. E.; Katzel, L. I.; Macko, R. F.; Ryan, A. S. | Strength Training for Skeletal Muscle Endurance after Stroke | Journal of Stroke and Cerebrovascular Diseases | 2017 | 26 | 4 | 787-94 | Wrong comparator - active control |
| Ivey 2011 | Ivey, F. M.; Ryan, A. S.; Hafer-Macko, C. E.; Macko, R. F. | Improved cerebral vasomotor reactivity after exercise training in hemiparetic stroke survivors | Stroke | 2011 | 42 | 7 | 1994-2000 | Wrong comparator - active control |
| Jin 2013 | Jin, H.; Jiang, Y.; Wei, Q.; Chen, L.; Ma, G. | Effects of aerobic cycling training on cardiovascular fitness and heart rate recovery in patients with chronic stroke | NeuroRehabilitation | 2013 | 32 | 2 | 327-35 | Wrong comparator - active control |
| Katz-Leurer 2006 | Katz-Leurer, M.; Sender, I.; Keren, O.; Dvir, Z. | The influence of early cycling training on balance in stroke patients at the subacute stage. Results of a preliminary trial | Clinical Rehabilitation | 2006 | 20 | 5 | 398-405 | Wrong intervention < 6 weeks |
| Kim 2001 | Kim, C. M.; Eng, J. J.; MacIntyre, D. L.; Dawson, A. S. | Effects of isokinetic strength training on walking in persons with stroke: a double-blind controlled pilot study | Journal of Stroke and Cerebrovascular Diseases | 2001 | 10 | 6 | 265-273 | Wrong comparator - active control |
| Kim 2017a | Kim, J.; Yim, J. | Effects of an exercise protocol for improving handgrip strength and walking speed on cognitive function in patients with chronic stroke | Medical Science monitor | 2017 | 23 |  | 5402-9 | Wrong comparator - active control |
| Kim 2016a | Kim, S. M.; Han, E. Y.; Kim, B. R.; Hyun, C. W. | Clinical application of circuit training for subacute stroke patients: a preliminary study | Journal of Physical Therapy Science | 2016 | 28 | 1 | 169-74 | Wrong intervention < 6 weeks |
| Lennon 2008 | Lennon, O.; Carey, A.; Gaffney, N.; Stephenson, J.; Blake, C. | A pilot randomized controlled trial to evaluate the benefit of the cardiac rehabilitation paradigm for the non-acute ischaemic stroke population | Clinical Rehabilitation | 2008 | 22 | 2 | 125-33 | Wrong comparator - co-interventions not balanced between arms |
| Lim 2000 | Lim, P. A. C.; Henson, H.; Cunha, I.; Qureshy, H.; Monga, T. N.; Protas, E. J. | Body weight-supported gait training in stroke patients | American Journal of Physical Medicine & Rehabilitation | 2000 | 79 | 2 | 203 | Wrong intervention < 6 weeks |
| MacKay-Lyons 2012 | MacKay-Lyons, Marilyn | Aerobic treadmill training effectively enhances cardiovascular fitness and gait function for older persons with chronic stroke | Journal of Physiotherapy | 2012 | 58 | 4 | 271 | Not relevant |
| Mackay-Lyons 2013 | Mackay-Lyons, M.; McDonald, A.; Matheson, J.; Eskes, G.; Klus, M. A. | Dual effects of body-weight supported treadmill training on cardiovascular fitness and walking ability early after stroke: a randomized controlled trial | NeuroRehabilitation and neural repair | 2013 | 27 | 7 | 644-53 | Wrong comparator - active control |
| Mao 2015 | Mao, Y. R.; Lo, W. L.; Lin, Q.; Li, L.; Xiao, X.; Raghavan, P. | The effect of body weight support treadmill training on gait recovery, proximal lower limb motor pattern, and balance in patients with subacute stroke | BioMed Research International | 2015 |  |  | 175719 | Wrong intervention < 6 weeks |
| Mead 2005 | Mead, G. | Exercise or relaxation after stroke? | British Medical Journal | 2005 | 330 | 7503 | 1337 | Not relevant |
| Moore 2014 | Moore, S.; Hallsworth, K.; Jakovljevic, D.; Blamire, A.; He, J.; Ford, G.; Rochester, L.; Trenell, M. | Effects of exercise therapy on metabolic risk factors brain atrophy and cerebral blood flow following stroke: a randomised controlled trial | International Journal of Stroke | 2014 | 9 | 4 | 32 | Wrong comparator - active control |
| Moore 2015 | Moore, S. A.; Hallsworth, K.; Jakovljevic, D. G.; Blamire, A. M.; He, J.; Ford, G. A.; Rochester, L.; Trenell, M. I. | Effects of Community Exercise Therapy on Metabolic, Brain, physical, and Cognitive Function Following Stroke: a Randomized Controlled Pilot Trial | NeuroRehabilitation and neural repair | 2015 | 29 | 7 | 623-635 | Wrong comparator - active control |
| Moore 2016 | Moore, S. A.; Jakovljevic, D. G.; Ford, G. A.; Rochester, L.; Trenell, M. I. | Exercise Induces Peripheral Muscle But Not Cardiac Adaptations After Stroke: a Randomized Controlled Pilot Trial | Archives of Physical Medicine and Rehabilitation | 2016 | 97 | 4 | 596-603 | Wrong comparator - active control |
| Moseley 2005 | Moseley, A. | Treadmill training more effective than Bobath training in improving walking following stroke | Australian Journal of Physiotherapy | 2005 | 51 | 3 | 192 | Not relevant |
| Mudge 2007 | Mudge, S. | ACTRN12607000081415: The impact of a group exercise programme on usual walking performance in adults who are at least 6 months post stroke: a single blinded randomised controlled trial | Australian New Zealand Clinical trials registry (ANZCTR) http://www.anzctr.org.au/ | 2007 |  |  |  | Wrong intervention < 6 weeks |
| Ouellette 2004 | Ouellette, M. M.; LeBrasseur, N. K.; Bean, J. F.; Phillips, E.; Stein, J.; Frontera, W. R.; Fielding, R. A. | High-intensity resistance training improves muscle strength, self-reported function, and disability in long-term stroke survivors | Stroke | 2004 | 35 | 6 | 1404-9 | Wrong comparator - active control |
| Perera 2006 | Perera, S.; Mody, S. H.; Woodman, R. C.; Studenski, S. A. | Meaningful change and responsiveness in common physical performance measures in older adults | Journal of the American Geriatrics Society | 2006 | 54 |  | 743-749 | Not relevant |
| Potempa 1995 | Potempa, K.; Lopez, M.; Braun, L. T.; Szidon, J. P.; Fogg, L.; Tincknell, T. | Physiological outcomes of aerobic exercise training in hemiparetic stroke patients | Stroke | 1995 | 26 | 1 | 101-105 | Wrong comparator - active control |
| Richards 2004 | Richards, C. L.; Malouin, F.; Bravo, G.; Dumas, F.; Wood-Dauphinee, S. | The role of technology in task-oriented training in persons with subacute stroke: a randomized controlled trial | NeuroRehabilitation and neural repair | 2004 | 18 | 4 | 199-211 | Wrong comparator - active control |
| Rose 2001 | Rose, D. K.; Winstein, C. J.; Tan, S. M.; Azen, S. P.; Chui, H. C. | Comparison of upper extremity intervention strategies at six and nine months post-stroke | Neurology report | 2001 | 25 | 4 | 130 | Wrong intervention < 6 weeks |
| Rose 1999 | Rose, D. K.; Winstein, C. J.; Yang, A. N.; Weiss, W. B.; Tan, S. M.; Azen, S. P.; Chui, H. C. | Relationship between upper extremity function and impairment in individuals with unilateral stroke | Neurology report | 1999 | 23 | 5 | 186 | Wrong study design - not RCT |
| Salbach 2005 | Salbach, N. M.; Mayo, N. E.; Robichaud-Ekstrand, S.; Hanley, J. A.; Richards, C. L.; Wood-Dauphinee, S. | The effect of a task-oriented walking intervention on improving balance self-efficacy poststroke: a randomized, controlled trial | Journal of the American Geriatrics Society | 2005 | 53 | 4 | 576-582 | Wrong comparator - active control |
| Salbach 2004 | Salbach, N. M.; Mayo, N. E.; Wood-Dauphinee, S.; Hanley, J. A.; Richards, C. L.; Côté, R. | A task-orientated intervention enhances walking distance and speed in the first year post stroke: a randomized controlled trial | Clinical Rehabilitation | 2004 | 18 | 5 | 509-519 | Wrong comparator - active control |
| Schmid 2007 | Schmid, A.; Duncan, P. W.; Studenski, S.; Lai, S. M.; Richards, L.; Perera, S.; Wu, S. S. | Improvements in speed-based gait classifications are meaningful | Stroke | 2007 | 38 | 7 | 2096-2100 | Not relevant |
| Shin 2011 | Shin, W. S.; Lee, S. W.; Lee, Y. W.; Choi, S. B.; Song, C. H. | Effects of combined exercise training on balance of hemiplegic stroke patients | Journal of Physical Therapy Science | 2011 | 23 | 4 | 639-643 | Wrong intervention < 6 weeks |
| Takami 2010 | Takami, Akiyoshi; Wakayama, Saichi | Effects of Partial Body Weight Support while Training Acute Stroke Patients to Walk Backwards on a Treadmill--A Controlled Clinical Trial Using Randomized Allocation | Journal of Physical Therapy Science | 2010 | 22 | 2 | 177-87 | Wrong intervention < 6 weeks |
| Teixeira 1998 | Teixeira, L.; Nadeau, S.; Olney, S.; McBride, I.; Culham, E.; Zee, B. | The impact of a muscle strengthening and physical conditioning program on gait and stairclimbing performance in chronic stroke subjects | Gait and Posture | 1998 | 7 | 2 | 144-145 | Wrong study design - not RCT |
| Teixeira-Salmela 2001 | Teixeira-Salmela, L. F.; Nadeau, S.; McBride, I.; Olney, S. J. | Effects of muscle strengthening and physical conditioning training on temporal, kinematic and kinetic variables during gait in chronic stroke survivors | Journal of Rehabilitation Medicine | 2001 | 33 | 2 | 53-60 | Wrong study design - not RCT |
| Wang 2014 | Wang, Z.; Wang, L.; Fan, H.; Lu, X.; Wang, T. | Effect of low-intensity ergometer aerobic training on glucose tolerance in severely impaired nondiabetic stroke patients | Journal of Stroke and Cerebrovascular Diseases | 2014 | 23 | 3 | e187-93 | Wrong comparator - active control |
| Winstein 2001 | Winstein, C. J.; Rose, D. K.; Chui, H. C.; Yang, A. N.; Weiss, W. B.; Tan, S. M.; Azen, S. P. | Recovery and rehabilitation of arm use after stroke | Journal of Stroke and Cerebrovascular Diseases | 2001 | 10 | 4 | 197 | Wrong intervention < 6 weeks |
| Winstein 2004 | Winstein, C. J.; Rose, D. K.; Tan, S. M.; Lewthwaite, R.; Chui, H. C.; Azen, S. P. | A randomized controlled comparison of upper-extremity rehabilitation strategies in acute stroke: a pilot study of immediate and long-term outcomes | Archives of Physical Medicine and Rehabilitation | 2004 | 85 | 4 | 620-8 | Wrong intervention < 6 weeks |
| Zou 2015 | Zou, J.; Wang, Z.; Qu, Q.; Wang, L. | Resistance training improves hyperglycemia and dyslipidemia, highly prevalent among nonelderly, nondiabetic, chronically disabled stroke patients | Archives of Physical Medicine and Rehabilitation | 2015 | 96 | 7 | 1291-6 | Wrong comparator - active control |
| Linxue 1999 | Linxue, L.; Nohara, R.; Makita, S.; Hosokawa, R.; Hata, T.; Okuda, K.; Hamazaki, H.; Fujita, M.; Sasayama, S. | Effect of long-term exercise training on regional myocardial perfusion changes in patients with coronary artery disease | Japanese circulation Journal | 1999 | 63 | 2 | 73-8 | Wrong study design - not RCT |
| Myers 1987 | Myers, J.; Ahnve, S.; Froelicher, V.; Sullivan, M.; Friis, R. | Influence of exercise training on spatial R-wave amplitude in patients with coronary artery disease | Journal of Applied Physiology | 1987 | 62 | 3 | 1231-5 | Wrong comparator - active control |
| Sullivan 1985 | Sullivan, M.; Ahnve, S.; Froelicher, V. F.; Meyers, J. | The influence of exercise training on the ventilatory threshold of patients with coronary heart disease | American Heart Journal | 1985 | 109 | 3 | 458-463 | Wrong comparator - active control |
| Weberg 2013 | Weberg, M; Hjermstad, MJ; Hilmarsen, CW; Oldervoll, L | Inpatient cardiac rehabilitation and changes in self-reported health related quality of life-“a pilot study | Annals of Physical and Rehabilitation Medicine | 2013 | 56 | 5 | 342-55 | Wrong study design - not RCT |
| Asbury 2012 | Asbury, E. A.; Webb, C. M.; Probert, H.; Wright, C.; Barbir, M.; Fox, K.; Collins, P. | Cardiac rehabilitation to improve physical functioning in refractory angina: a pilot study | Cardiology | 2012 | 122 | 3 | 170-177 | Wrong comparator - co-interventions not balanced between arms |
| Bubnova 2014 | Bubnova, MG; Aronov, DM; KrasnitskiÄ­, VB; Ioseliani, DG; Novikova, NK; Rodzinskaia, EM | A home exercise training program after acute coronary syndrome and/or endovascular coronary intervention: efficiency and a patient motivation problem | Terapevticheskii arkhiv | 2014 | 86 | 1 | 23-32 | Wrong comparator - active control |
| Butler 2009 | Butler, L.; Furber, S.; Phongsavan, P.; Mark, A.; Bauman, A. | Effects of a pedometer-based intervention on physical activity levels after cardiac rehabilitation: a randomized controlled trial | Journal of Cardiopulmonary Rehabilitation and Prevention | 2009 | 29 | 2 | 105-114 | Wrong intervention - does not meet exercise definition |
| Carlsson 1997 | Carlsson, R.; Lindberg, G.; Westin, L.; Israelsson, B. | Influence of coronary nursing management follow up on lifestyle after acute myocardial infarction | Heart (British Cardiac Society) | 1997 | 77 | 3 | 256-259 | Wrong comparator - co-interventions not balanced between arms |
| Chatian 2014 | Chatian, Monika; Tarchalski, Janusz Lech; Lisowski, Jacek; Poziomska-Piatkowska, Elzbieta | The influence of the outpatient cardiologic rehabilitation on the physical fitness at patients after a STEMI | Polski Merkuriusz Lekarski: Organ Polskiego Towarzystwa Lekarskiego | 2014 | 36 | 212 | 88-91 | Full text not in English |
| Claes 2020 | Claes, Jomme; Cornelissen, Véronique; McDermott, Clare; Moyna, Niall; Pattyn, Nele; Cornelis, Nils; Gallagher, Anne; McCormack, Ciara; Newton, Helen; Gillain, Alexandra | Feasibility, acceptability, and Clinical effectiveness of a technology-enabled cardiac rehabilitation platform (Physical activity Toward Health-I): randomized controlled trial | Journal of Medical Internet Research | 2020 | 22 | 2 | e14221 | Wrong comparator - co-interventions not balanced between arms |
| Clark 2017 | Clark, Imogen N; Baker, Felicity A; Peiris, Casey L; Shoebridge, Georgie; Taylor, Nicholas F | Participant-selected music and physical activity in older adults following cardiac rehabilitation: A randomized controlled trial | Clinical Rehabilitation | 2017 | 31 | 3 | 329-339 | Wrong intervention - does not meet exercise definition |
| Giannuzzi 2008 | Giannuzzi, P.; Temporelli, P. L.; Marchioli, R.; Maggioni, A. P.; Balestroni, G.; Ceci, V.; Chieffo, C.; Gattone, M.; Griffo, R.; Schweiger, C.; et al., | Global secondary prevention strategies to limit event recurrence after myocardial infarction: results of the GOSPEL study, a multicenter, randomized controlled trial from the Italian Cardiac Rehabilitation Network | Archives of Internal Medicine | 2008 | 168 | 20 | 2194-2204 | Wrong comparator - co-interventions not balanced between arms |
| Pakrad 2021 | IRCT20130211012439N3 | Comparative evaluation of discharge planning and cardiac rehabilitation on the health outcomes of patients undergoing coronary artery bypass graft surgery | Archives of Physical Medicine and Rehabilitation | 2021 | 102 |  | 2091-101 | Wrong comparator - co-interventions not balanced between arms |
| Izawa 2006 | Izawa, Kazuhiro P; Watanabe, Satoshi; Oka, Koichiro; Kobayashi, Toru; Osada, Naohiko; Omiya, Kazuto | The effects of unsupervised exercise training on physical activity and physiological factors after supervised cardiac rehabilitation | Journal of the Japanese Physical Therapy Association | 2006 | 9 | 1 | 1-8 | Wrong comparator - active control |
| Li 2004 | Li, H.; Guo, L.; Sun, J. Z.; Feng, J. Z.; Wang, P.; Wu, G. L. | Effect of exercise therapy on the quality of life in patients after successful percutaneous transluminal coronary angioplasty | Chinese Journal of Clinical Rehabilitation | 2004 | 8 | 9 | 1601-1603 | Full text not in English |
| Liao 2003 | Liao, X.; Ma, H.; Dong, Y. | Effects of early rehabilitation programme on heart rate variability and quality of life in patients with un-complicated acute myocardial infarction | Chinese Journal of Rehabilitative Medicine | 2003 | 18 | 3 | 153-155 | Wrong intervention < 6 weeks |
| Martinez 2011 | Martinez, D. G.; Nicolau, J. C.; Lage, R. L.; Toschi-Dias, E.; de Matos, L. D.; Alves, M. J.; Trombetta, I. C.; Dias da Silva, V. J.; Middlekauff, H. R.; Negrão, C. E.; et al., | Effects of long-term exercise training on autonomic control in myocardial infarction patients | Hypertension | 2011 | 58 | 6 | 1049-1056 | Wrong study design - not RCT |
| Murphy 2012 | Murphy, Simon Mark; Edwards, Rhiannon Tudor; Williams, Nefyn; Raisanen, Larry; Moore, Graham; Linck, Pat; Hounsome, Natalia; Din, Nafees Ud; Moore, Laurence | An evaluation of the effectiveness and cost effectiveness of the National Exercise Referral Scheme in Wales, UK: a randomised controlled trial of a public health policy initiative | J Epidemiol Community Health | 2012 | 66 | 8 | 745-753 | Wrong intervention - does not meet exercise definition |
| NCT04294940 2020 | NCT04294940 | Impact of a digital solution (CardiCare^TM^) on cardiorespiratory fitness improvement in patients discharged from a phase 2 cardiac rehabilitation following an acute coronary syndrome |  |  |  |  |  | Not relevant |
| NCT04441086 2020 | NCT04441086 | Emotion regulation intervention to sustain physical activity in rural-dwelling women and men after myocardial infarction |  |  |  |  |  | Wrong intervention - does not meet exercise definition |
| Pozehl 2018 | Pozehl, B. J.; McGuire, R.; Duncan, K.; Kupzyk, K.; Norman, J.; Artinian, N. T.; Deka, P.; Krueger, S. K.; Saval, M. A.; Keteyian, S. J. | Effects of the HEART Camp Trial on Adherence to Exercise in Patients With Heart Failure | Journal of Cardiac Failure | 2018 | 24 | 10 | 654-660 | Not relevant |
| Pratesi 2016 | Pratesi, A.; Baldasseroni, S.; Barucci, R.; Pallante, R.; Foschini, A.; Venturini, S.; Mannarino, G.; Nucci, V.; Burgisser, C.; Marchionni, N.; et al., | Effects of cardiac rehabilitation in maintaining physical performance of patients aged >75 years over the long-term period, after an acute coronary syndrome or heart surgery | European Geriatric Medicine | 2016 | 7 |  | S20 | Wrong comparator - co-interventions not balanced between arms |
| Pratesi 2019 | Pratesi, A.; Baldasseroni, S.; Burgisser, C.; Orso, F.; Barucci, R.; Silverii, M. V.; Venturini, S.; Ungar, A.; Marchionni, N.; Fattirolli, F. | Long-term functional outcomes after cardiac rehabilitation in older patients. Data from the Cardiac Rehabilitation in Advanced aGE: eXercise TRaining and Active follow-up (CR-AGE EXTRA) randomised study | European Journal of Preventive Cardiology | 2019 | 26 | 14 | 1470-1478 | Wrong comparator - active control |
| Sadeghi 2013 | Sadeghi, M.; Garakyaraghi, M.; Khosravi, M.; Taghavi, M.; Sarrafzadegan, N.; Roohafza, H. | The impacts of cardiac rehabilitation program on echocardiographic parameters in coronary artery disease patients with left ventricular dysfunction | Cardiology Research and Practice | 2013 | 1 | 1 |  | Wrong study design - not RCT |
| Shabani 2010 | Shabani, Ramin; Gaeini, Abas A; Nikoo, Mohamad R; Nikbackt, Hojatollah; Sadegifar, Majid | Effect of cardiac rehabilitation program on exercise capacity in women undergoing coronary artery bypass graft in Hamadan-Iran | International Journal of Preventive Medicine | 2010 | 1 | 4 | 247 | Wrong study design - not RCT |
| Wang 2019 | Wang, Zhaoping; Ji, Linlin; Xiang, Aixia; Wang, Yingcui; Jiang, Dajun; Zhang, Ning; Wang, Yabo; Yang, Wenjing; Hui, Sun; Wang, Ge | Effect of cardiac rehabilitation on cardiopulmonary function in patients with diabetes mellitus complicated with acute myocardial infarction and heart failure |  | 2019 |  |  |  | Wrong comparator - co-interventions not balanced between arms |
| Wood 2008 | Wood, D. A.; Kotseva, K.; Connolly, S.; Jennings, C.; Mead, A.; Jones, J.; Holden, A.; De Bacquer, D.; Collier, T.; De Backer, G.; et al., | Nurse-coordinated multidisciplinary, family-based cardiovascular disease prevention programme (EUROACTION) for patients with coronary heart disease and asymptomatic individuals at high risk of cardiovascular disease: a paired, cluster-randomised cont | Lancet | 2008 | 371 | 9629 | 1999-2012 | Wrong comparator - co-interventions not balanced between arms |
| Bronas 2011 | Bronas, U. G.; Treat-Jacobson, D.; Leon, A. S. | Comparison of the effect of upper body-ergometry aerobic training vs treadmill training on central cardiorespiratory improvement and walking distance in patients with claudication | Journal of Vascular Surgery | 2011 | 53 | 6 | 1557-64 | Wrong comparator - exercise v exercise |
| Brotons 2011 | Brotons, C.; Soriano, N.; Moral, I.; Rodrigo, M. P.; Kloppe, P.; Rodríguez, A. I.; González, M. L.; Ariño, D.; Orozco, D.; Buitrago, F.; et al., | Randomized Clinical trial to assess the efficacy of a comprehensive programme of secondary prevention of cardiovascular disease in general Practice: the PREseAP study | Revista Española de Cardiología | 2011 | 64 | 1 | 13-20 | Wrong intervention - does not meet exercise definition |
| Dittmar 1977 | Dittmar, K.; Krause, D. | Success of physiotherapeutic interval training combined with a standardized hemoderivative in intermittent claudication | Munchener medizinische Wochenschrift | 1977 | 119 | 11 | 369-72 | Not relevant |
| Gardner 2011 | Gardner, A. W.; Parker, D. E.; Montgomery, P. S.; Scott, K. J.; Blevins, S. M. | Efficacy of quantified home-based exercise and supervised exercise in patients with intermittent claudication: a randomized controlled trial | Circulation | 2011 | 123 | 5 | 491-8 | Wrong comparator - exercise v exercise |
| Kono 2013 | Kono, Y.; Yamada, S.; Yamaguchi, J.; Hagiwara, Y.; Iritani, N.; Ishida, S.; Araki, A.; Hasegawa, Y.; Sakakibara, H.; Koike, Y. | Secondary prevention of new vascular events with lifestyle intervention in patients with noncardioembolic mild ischemic stroke: a single-center randomized controlled trial | Cerebrovascular Diseases | 2013 | 36 | 2 | 88-97 | Wrong comparator - co-interventions not balanced between arms |
| Leon 2005 | Leon, A. S. | Does long-term aerobic exercise slow progression of atherosclerosis? | Clinical Journal of Sport Medicine | 2005 | 15 | 4 | 285-6 | Not relevant |
| Mays 2014 | Mays, R. J.; Casserly, I.; Rogers, R.; Main, D.; Hiatt, W.; Kohrt, W.; Ho, P.; Regensteiner, J. | Community-based walking exercise for patients with peripheral artery disease: a pilot study moderated | Journal of the American College of Cardiology | 2014 | 63 | 12 | A2035 | Wrong comparator - co-interventions not balanced between arms |
| Mays 2015 | Mays, R. J.; Hiatt, W. R.; Casserly, I. P.; Rogers, R. K.; Main, D. S.; Kohrt, W. M.; Ho, P. M.; Regensteiner, J. G. | Community-based walking exercise for peripheral artery disease: an exploratory pilot study | Vascular Medicine | 2015 | 20 | 4 | 339-47 | Wrong comparator - co-interventions not balanced between arms |
| McDermott 2014 | McDermott, Mary; Bonds, Denise; Buford, Thomas; Church, Tim; Dodson, John; Fielding, Roger; Guralnik, Jack; Kritchevsky, Steve; Lovato, Laura; Pahor, Marco | A physical activity Intervention Improves 400-Meter Walking Velocity as Compared to a Successful Aging Intervention in Patients with Peripheral Artery Disease: The LIFE Study Randomized Trial | Circulation | 2014 | 130 | 2 | A19294-A19294 | Not relevant |
| NCT01065740 2010 | NCT01065740 | Optimized supervised education program for peripheral arterial disease (POPART) |  |  |  |  |  | Wrong intervention - does not meet exercise definition |
| Mays 2014 | NCT02075502 | Community walking exercise for patients with peripheral artery disease (GAIT) |  |  |  |  |  | Wrong comparator - co-interventions not balanced between arms |
| Nordanstig 2011 | Nordanstig, J.; Gelin, J.; Hensäter, M.; Taft, C.; Österberg, K.; Jivegård, L. | Walking performance and health-related quality of life after surgical or endovascular invasive versus non-invasive treatment for intermittent claudication--a prospective randomised trial | European Journal of Vascular and Endovascular Surgery | 2011 | 42 | 2 | 220-227 | Wrong comparator - active control |
| Treat-Jacobson 2009 | Treat-Jacobson, D.; Bronas, U. G.; Leon, A. S. | Efficacy of arm-ergometry versus treadmill exercise training to improve walking distance in patients with claudication | Vascular Medicine | 2009 | 14 | 3 | 203-213 | Wrong comparator - exercise v exercise |
| Frimpong 2014 | Frimpong, Eea; Olawale, OA; Antwi, DA; Antwi-Boasiako, C; Dzudzor, B | Task-oriented circuit training improves ambulatory functions in acute stroke: a randomized controlled trial |  | 2014 |  |  |  | Wrong comparator - exercise v exercise |
| Matsumoto 2016 | Matsumoto, S.; Uema, T.; Ikeda, K.; Miyara, K.; Nishi, T.; Noma, T.; Shimodozono, M. | Effect of Underwater Exercise on Lower-Extremity Function and Quality of Life in Post-Stroke Patients: a Pilot Controlled Clinical Trial | Journal of alternative and complementary Medicine (New York, N.Y.) | 2016 | 22 | 8 | 635-641 | Wrong study design - not RCT |
| Barrow 2007 | Barrow, DE; Bedford, A; Ives, G; O'Toole, L; Channer, KS | An evaluation of the effects of Tai Chi Chuan and Chi Kung training in patients with symptomatic heart failure: a randomised controlled pilot study | Postgraduate Medical Journal | 2007 | 83 | 985 | 717-721 | Wrong intervention - does not meet exercise definition |
| Bittencourt 2015 | Bittencourt, L; Servante, D; Javaheri, S; Tufik, S | Effects of exercise training and CPAP in patients with heart failure and obstructive sleep apnea | Sleep | 2015 | 38 |  | A177 | Wrong study design - not RCT |
| Jónsdóttir 2006 | Jónsdóttir, Sólrún; Andersen, Karl K; Sigurðsson, Axel F; Sigurðsson, Stefáán B | The effect of physical training in chronic heart failure | European Journal of Heart Failure | 2006 | 8 | 1 | 97-101 | Wrong comparator - co-interventions not balanced between arms |
| Oliveira 2015 | Oliveira, Mayron F; Santos, Rita L; Mendez, Vanessa M; Sperandio, Priscila A; Umeda, Iracema I; Correa, Edileide B; Ferraz, Almir S | Safety and Efficacy of Non-invasive Ventilation During Exercise Training in Patients With Acute Heart Failure. A Randomized Prospective Controlled Study | Circulation | 2015 | 132 | 3 | A15821-A15821 | Wrong intervention < 6 weeks |
| Piotrowicz 2015 | Piotrowicz, Ewa; Zieliński, Tomasz; Bodalski, Robert; Rywik, Tomasz; Dobraszkiewicz-Wasilewska, Barbara; Sobieszczańska-Małek, Małgorzata; Stepnowska, Monika; Przybylski, Andrzej; Browarek, Aldona; Szumowski, ŁŁukasz | Home-based telemonitored Nordic walking training is well accepted, safe, effective and has high adherence among heart failure patients, including those with cardiovascular implantable electronic devices: a randomised controlled study | European Journal of Preventive Cardiology | 2015 | 22 | 11 | 1368-1377 | Wrong comparator - co-interventions not balanced between arms |
| Ponikowski 1997 | Ponikowski, Piotr Paweł; Szelemej, R; Kowalska-Superlak, M; Kratochwil, D; Sobkowicz, Bożena; Sebzda, T; Hanczyc, H; Wrabec, K | Exercise rehabilitation in patients with moderate-severe chronic heart failure | Kardiologia Polska | 1997 | 47 |  |  | Full text not in English |
| Smart 2007 | Smart, Neil; Haluska, Brian; Jeffriess, Leanne; Marwick, Thomas H | Exercise training in systolic and diastolic dysfunction: effects on cardiac function, functional capacity, and quality of life | American Heart Journal | 2007 | 153 | 4 | 530-536 | Wrong study design - not RCT |
| Soska 2014 | Soska, Vladimir; Dobsak, Petr; Pohanka, Michal; Spinarova, Lenka; Vitovec, Jiri; Krejci, Jan; Hude, Petr; Homolka, Pavel; Novakova, Marie; Eicher, Jean-Christophe | Exercise training combined with electromyostimulation in the rehabilitation of patients with chronic heart failure: A randomized trial | BioMedical Papers of the Medical Faculty of Palacky University in Olomouc | 2014 | 158 | 1 |  | Wrong comparator - active control |
| Suna 2015 | Suna, Jessica M; Mudge, Alison; Stewart, Ian; Marquart, Louise; O'Rourke, Peter; Scott, Adam | The effect of a supervised exercise training programme on sleep quality in recently discharged heart failure patients | European Journal of Cardiovascular Nursing | 2015 | 14 | 3 | 198-205 | Wrong comparator - active control |
| Wisløff 2007 | Wisløff, Ulrik; Støylen, Asbjørn; Loennechen, Jan P; Bruvold, Morten; Rognmo, Øivind; Haram, Per Magnus; Tjønna, Arnt Erik; Helgerud, Jan; Slørdahl, Stig A; Lee, Sang Jun | Superior cardiovascular effect of aerobic interval training versus moderate continuous training in heart failure patients: a randomized study | Circulation | 2007 | 115 | 24 | 3086-3094 | Wrong comparator - exercise v exercise |
| Zhang 2003 | Zhang, F; Lui, J; Zhang, S; Yuan, M | Effect of walking movement on plasma TNF and receptor in chronic heart failure patients | Chinese Journal of Clinical Rehabilitation | 2003 | 7 | 24 | 2248-9 | Full text not in English |
| Taylor 2015 | Taylor, Rod S; Hayward, C; Eyre, V; Austin, J; Davies, Russell; Doherty, P; Jolly, Kate; Wingham, J; Van Lingen, R; Abraham, Charles | Clinical effectiveness and cost-effectiveness of the Rehabilitation Enablement in Chronic Heart Failure (REACH-HF) facilitated self-care rehabilitation intervention in heart failure patients and caregivers: rationale and protocol for a multicentre randomised controlled trial | BMJ Open | 2015 | 5 | 12 | e009994 | Wrong comparator - co-interventions not balanced between arms |
| Chattopadhyay 2019 | Chattopadhyay, Kaushik; Chandrasekaran, Ambalam M; Praveen, Pradeep A; Manchanda, Subhash C; Madan, Kushal; Ajay, Vamadevan S; Singh, Kavita; Tillin, Therese; Hughes, Alun D; Chaturvedi, Nishi | Development of a yoga-based cardiac rehabilitation (Yoga-CaRe) programme for secondary prevention of myocardial infarction | Evidence-Based Complementary and Alternative Medicine | 2019 | 2019 |  |  | Wrong comparator - co-interventions not balanced between arms |
| Dorje 2019 | ChiCTR-INR-16009598 | SMARTphone-based Home Cardiac Rehabilitation and Secondary Prevention in Chinese Coronary Heart Disease Patients (SMART-CR/SP): a randomized controlled trial. |  | date of registration 25 October 2016 |  |  |  | Wrong comparator - co-interventions not balanced between arms |
| Prabhakaran 2020 | CTRI/2012/02/002408 | A study on effectiveness of yoga based cardiac rehabilitation programme in India and United Kingdom. |  | date of registration 8 February 2012 |  |  |  | Wrong comparator - co-interventions not balanced between arms |
| Dean 2012 | Dean, Catherine | Group task-specific circuit training for patients discharged home after stroke may be as effective as individualised physiotherapy in improving mobility | Journal Of Physiotherapy | 2012 | 58 | 4 | 269 | Not relevant |
| DeBusk 1994 | DeBusk, Robert F; Miller, Nancy Houston; Superko, H Robert; Dennis, Charles A; Thomas, Randal J; Lew, Henry T; Berger, Walter E; Heller, Robert S; Rompf, Jonathan; Gee, David | A case-management system for coronary risk factor modification after acute myocardial infarction | Annals of Internal Medicine | 1994 | 120 | 9 | 721-9 | Wrong comparator - co-interventions not balanced between arms |
| Fridlund 1992 | Fridlund, Bengt; Pihlgren, Christina; WANNESTIG, LENA-BRITT | A supportive-“educative caring rehabilitation programme; improvements of physical health after myocardial infarction | Journal of Clinical Nursing | 1992 | 1 | 3 | 141-146 | Wrong comparator - co-interventions not balanced between arms |
| JRRD 2016 | Multiple | JRRD at a Glance | Journal of Rehabilitation Research & Development | 2016 | 53 | 6 | xi-xx | Not relevant |
| ECNR 2017 | European Congress of NeuroRehabilitation 2017 | 25. Jahrestagung der Deutschen Gesellschaft fur Neurorehabilitation e. V., DGNR 2017 | Neurologie und Rehabilitation | 2017 | 23 |  |  | Not relevant |
| WKW 2017 | Wiener Klinische Wochenschrift. The Central European Journal of Medicine. | 41. Jahrestagung der Osterreichischen Gesellschaft fur Pneumologie, 1. Jahrestagung der Osterreichischen Gesellschaft fur Thoraxchirurgie | Wiener klinische Wochenschrift | 2017 | 129 | 19-20 |  | Not relevant |
| EuroPRevent 2017 | Multiple | Abstract Book EuroPRevent 2017 | European Journal of Preventive Cardiology | 2017 | 24 | 1 |  | Not relevant |
| Zhao 2017 | Zhao, Q-Z.;Liu, H-M. | Influences of long-term aerobic exercise combined with resistance training on the cardiac function, exercise tolerance, and living quality of patients with myocardial ischemia induced by coronary heart disease | BioMedical Research (India) | 2017 | 28 | 10 | 4539-4542 | Wrong comparator - exercise v exercise |
| SOFPEL 2017 | SOFPEL 2017 | Resumes du 24e Congres Annuel de la Societe Francophone Posture Equilibre Locomotion, SOFPEL 2017 | Neurophysiologie clinique | 2017 | 47 |  |  | Exclude - not able to retrieve full text |
| AAGBI 2018 | Multiple | Abstracts of AAGBI Annual Congress 2018 | Anaesthesia | 2018 | 73 |  |  | Not relevant |
| Kim 2018 | Kim, C-H.; Kim, Y-N. | Effects of Proprioceptive Neuromuscular Facilitation and Treadmill Training on the Balance and Walking Ability of Stroke Patients | J korean phys ther | 2018 | 30 | 3 | 79-83 | Not relevant |
| Choi 2018 | Choi, M. S.; Lee, J.S., Kim, K.; Kim, Y. M. | Effects of Treadmill Gait Training According to Different Inclination on Postural Balance in Patients with Chronic Stroke | J korean phys ther | 2018 | 30 | 6 | 205-210 | Not relevant |
| Ryu 2018 | Ryu, H.Y.; Kim, K.S.; Jeon, I.C. | Influence of Home Based Exercise Intensity on the Aerobic Capacity and 1 Year Re-Hospitalization Rate in Patients with Chronic Heart Failure | J korean phys ther | 2018 | 30 | 5 | 181-186 | Full text not in English |
| Anon 2018 | Anon | Introductory paragraph | Clinical Rehabilitation | 2018 | 32 | 2 | 130-130 | Not relevant |
| Choi 2018 | Choi, H-Y.; Han, H-J.; Choi, J-W.; Jung, H-Y., Joa, K-L. | Superior Effects of High-Intensity Interval Training Compared to Conventional Therapy on Cardiovascular and Psychological Aspects in Myocardial Infarction | Annals of Rehabilitation Medicine | 2018 | 42 | 1 | 145-153 | Wrong comparator - exercise v exercise |
| ASMBS 2019 | Multiple | ASMBS 36th Annual Meeting at Obesity Week 2019 Abstracts | Surgery for Obesity and Related Diseases | 2019 | 15 | 10 | S1-S268 | Not relevant |
| Mun 2019 | Mun, B.M.; Kin, T.H. | The Effect of Lower Extremity Strengthening Exercise Using Sliding Stander on Balance and Spasticity in Chronic Stroke: a Randomized Clinical Trial | J korean phys ther | 2019 | 31 | 5 | 311-316 | Wrong intervention - does not meet exercise definition |
| Kim 2019 | Kim, H., Chung, Y. | Effect of Underwater Gait Training with a Progressive Increase in Speed on Balance, Gait, and Endurance in Stroke Patients | J korean phys ther | 2019 | 31 | 4 | 204-211 | Wrong comparator - exercise v exercise |
| Lee 2019 | Lee, D-K., Hwang, T-Y. | Effects of Aquatic Proprioceptive Neuromuscular Facilitation Pattern Exercise on Balance, Gait ability and Depression in Patients with Chronic Stroke | J korean phys ther | 2019 | 31 | 4 | 236-241 | Wrong intervention - does not meet exercise definition |
| Youn 2019 | Youn, S.Y.; Park, S.J. | Immediate Effect of Sustained Stretching Exercises with Far Infrared on the Ankle Range of Motion and Muscle Tone in Patients with Stroke | J korean phys ther | 2019 | 31 | 1 | 56-61 | Full text not in English |
| Stroke 2022 | Multiple | Abstracts From the American Stroke Association 2022 International Stroke Conference and State-of-the-Science Stroke Nursing Symposium 2022 | Stroke | 2022 | 53 | 1 |  | Not relevant |
| Aamot 2016 | Aamot, I. L.; Karlsen, T.; Dalen, H.; Støylen, A. | Long-term Exercise Adherence After High-intensity Interval Training in Cardiac Rehabilitation: a Randomized Study | Physiotherapy Research International | 2016 | 21 | 1 | 54-64 | Wrong comparator - exercise v exercise |
| Aarhus 2020 | Aarhus, University of | Weight-Adjusted Dosing of 3-OHB in Patients With Chronic Heart Failure |  | 2020 |  |  |  | Not relevant |
| Aarhus 2018 | Aarhus, University of; University, Aalborg; Hospital, North Denmark Regional | Music Listening for Cardiorespiratory Exercise in Inpatient Stroke Rehabilitation |  | 2018 |  |  |  | Wrong study design - not RCT |
| Abdeen 2023 | Abdeen, H. A.; Helmy, Z. M.; Elnaggar, M. I.; Aldhahi, M. I.; Taha, M. M.; Marques-Sule, E.; Amin, D. I.; Ibrahim, B. S.; Aziz, A. A.; Castiglione, V.; Atef, H. | Different Continuous Training Intensities Improve Echocardiographic Parameters, Quality of Life, and Functional Capacity in Heart Failure Patients with Reduced Ejection Fraction | International Journal of General Medicine | 2023 | 16 |  | 3933-3945 | Wrong comparator - exercise v exercise |
| Abdelhalem 2018 | Abdelhalem, A. M.; Shabana, A. M.; Onsy, A. M.; Gaafar, A. E. | High intensity interval training exercise as a novel protocol for cardiac rehabilitation program in ischemic Egyptian patients with mild left ventricular dysfunction | Egyptian Heart Journal | 2018 | 70 | 4 | 287-294 | Wrong comparator - exercise v exercise |
| Abdelnour 2022 | Abdelnour, N.; Maynard, L.; Dubost, J.; Assi, A.; Tomb, R.; Mesure, S. | Effet d'un programme de facilitation proprioceptive neuromusculaire (concept PNF) sur la marcheet sur la force musculairechez des patients hémiparétiquesÂ : essai contrôlé randomisé | Kinésithérapie Revue | 2022 | 22 | 246 | 3-11 | Full text not in English |
| AbdollahpourAlni 2022 | Abdollahpour Alni, M.; Nikookheslat, S. D. | The effect of 12 weeks aerobic, resistance and combined trainings on peripheral vascular disease in type 2 diabetes with peripheral neuropathy in men | Obesity Medicine | 2022 | 34 |  |  | Not relevant |
| AbilityLab 2016 | AbilityLab, Shirley Ryan | Ventilatory Muscle Training in Stroke |  | 2016 |  |  |  | Wrong intervention - does not meet exercise definition |
| AbilityLab 2023 | AbilityLab, Shirley Ryan | Muscle Coordination-Based Feedback for Stroke Rehabilitation |  | 2023 |  |  |  | Wrong comparator - active control |
| AbilityLab 2024 | AbilityLab, Shirley Ryan | Comparison of Gait Training Methods in Sub-acute Stroke and Spinal Cord Injury |  | 2024 |  |  |  | Wrong study design - not RCT |
| Abo 2017 | Abo, M. | Effect of the transcranial magnetism stimulation treatment. [Japanese] | Tokyo Jikeikai Medical Journal | 2017 | 132(2) |  | 31-36 | Full text not in English |
| Abraha 2018 | Abraha, B.; Chaves, A. R.; Kelly, L. P.; Wallack, E. M.; Wadden, K. P.; McCarthy, J.; Ploughman, M. | A bout of high intensity interval training lengthened nerve conduction latency to the non-exercised affected limb in chronic stroke | Frontiers of Physiology | 2018 | 9 |  | 827 | Not relevant |
| Abraham 2018 | Abraham, W. T.; Kuck, K. H.; Goldsmith, R. L.; Lindenfeld, J.; Reddy, V. Y.; Carson, P. E.; Mann, D. L.; Saville, B.; Parise, H.; Chan, R.; Wiegn, P.; Hastings, J. L.; Kaplan, A. J.; Edelmann, F.; Luthje, L.; Kahwash, R.; Tomassoni, G. F.; Gutterman, D. D.; Stagg, A.; Burkhoff, D.; HasenfuíŸ, G. | A Randomized Controlled Trial to Evaluate the Safety and Efficacy of Cardiac Contractility Modulation | JACC. Heart Failure | 2018 | 6 | 10 | 874-883 | Not relevant |
| Abreu 2016 | Abreu, A. | Comment on "variables measured during cardiopulmonary exercise testing as predictors of mortality in chronic systolic heart failure". [Portuguese] | Revista Portuguesa de Cardiologia | 2016 | 35(5) |  | 317-318 | Wrong study design - not RCT |
| Abroskina 2016 | Abroskina, M.; Kaygorodceva, S.; Prokopenko, S.; Lyapin, A. | Comparative efficiency of the posture correction methods in patients after stroke | International Journal of Stroke | 2016 | 11 |  | 123-124 | Wrong intervention - does not meet exercise definition |
| AcarOzkoslu 2017 | Acar Ozkoslu, M.; Sahin Sonmezer, I. E.; Kose, B.; Polat, G.; Yigit, S.; Eren, H.; Cicek, T.; Balci, E. G. | The immediate effects of proprioceptive neuromuscular facilitation (PNF) techniques and classic exercises in stroke patients: A pilot study | Fizyoterapi Rehabilitasyon | 2017 | 28(2) |  | S35 | Not relevant |
| Actrn 2016 | Actrn, | The effect of exercise on brain volume and function after stroke | http://www.who.int/trialsearch/Trial2.aspx?TrialID=ACTRN12616000942459 | 2016 |  |  |  | Wrong comparator - active control |
| Actrn 2016 | Actrn, | The effects of Ai-Chi intervention on balance and gait performance in individuals with chronic stroke | http://www.who.int/trialsearch/Trial2.aspx?TrialID=ACTRN12616000769482 | 2016 |  |  |  | Wrong comparator - exercise v exercise |
| Actrn 2016 | Actrn, | The effects of different intensity exercise training in patients with ventricular assist devices | https://trialsearch.who.int/Trial2.aspx?TrialID=ACTRN12616001596493 | 2016 |  |  |  | Wrong comparator - exercise v exercise |
| Actrn 2016 | Actrn, | The efficacy of gait training using a body weight support treadmill and visual biofeedback in patients with subacute stroke: a randomized controlled trial | https://trialsearch.who.int/Trial2.aspx?TrialID=ACTRN12616001283460 | 2016 |  |  |  | Not relevant |
| Actrn 2016 | Actrn, | An electromechanical gait trainer for ambulation training after stroke | https://trialsearch.who.int/Trial2.aspx?TrialID=ACTRN12616000844448 | 2016 |  |  |  | Not relevant |
| Actrn 2016 | Actrn, | High Intensity Aerobic Interval and Training within Supervised Cardiac Rehabilitation | http://www.who.int/trialsearch/Trial2.aspx?TrialID=ACTRN12616001338459 | 2016 |  |  |  | Wrong comparator - exercise v exercise |
| Actrn 2016 | Actrn, | Promoting physical activity after stroke via self-management: a pilot randomised trial | https://trialsearch.who.int/Trial2.aspx?TrialID=ACTRN12616000325404 | 2016 |  |  |  | Not relevant |
| Actrn 2016 | Actrn, | Tele-rehabilitation for the arm after stroke | http://www.who.int/trialsearch/Trial2.aspx?TrialID=ACTRN12616001724460 | 2016 |  |  |  | Wrong intervention - does not meet exercise definition |
| Actrn 2016 | Actrn, | Water-based exercise in people with stable coronary heart disease (Study A) | https://trialsearch.who.int/Trial2.aspx?TrialID=ACTRN12616000113459 | 2016 |  |  |  | Not relevant |
| Actrn 2017 | Actrn, | Biofeedback gait training in stroke patients | http://www.who.int/trialsearch/Trial2.aspx?TrialID=ACTRN12617000250336 | 2017 |  |  |  | Not relevant |
| Actrn 2017 | Actrn, | Brain blood flow, type 2 diabetes, and high-intensity interval training | http://www.who.int/trialsearch/Trial2.aspx?TrialID=ACTRN12617001240336 | 2017 |  |  |  | Not relevant |
| Actrn 2017 | Actrn, | Core muscles strengthening for balance and gait performance in individuals with chronic stroke | http://www.who.int/trialsearch/Trial2.aspx?TrialID=ACTRN12617000452392 | 2017 |  |  |  | Wrong comparator - exercise v exercise |
| Actrn 2017 | Actrn, | GotRhythm: a tailored music therapy and real-time biofeedback mobile phone app to promote rehabilitation following stroke | http://www.who.int/trialsearch/Trial2.aspx?TrialID=ACTRN12617000488303 | 2017 |  |  |  | Wrong intervention - does not meet exercise definition |
| Actrn 2017 | Actrn, | Improving arm function after stroke using task specific training | http://www.who.int/trialsearch/Trial2.aspx?TrialID=ACTRN12617001631392 | 2017 |  |  |  | Wrong intervention - does not meet exercise definition |
| Actrn 2017 | Actrn, | Interactive Virtual Therapy for community-dwelling Stroke survivors | https://trialsearch.who.int/Trial2.aspx?TrialID=ACTRN12617000745347 | 2017 |  |  |  | Wrong intervention - does not meet exercise definition |
| Actrn 2017 | Actrn, | Physical activity education in community rehabilitation | http://www.who.int/trialsearch/Trial2.aspx?TrialID=ACTRN12617000519358 | 2017 |  |  |  | Not relevant |
| Actrn 2017 | Actrn, | A pilot study of tongue stimulation to improve balance and gait in stroke survivors | http://www.who.int/trialsearch/Trial2.aspx?TrialID=ACTRN12617000013369 | 2017 |  |  |  | Not relevant |
| Actrn 2017 | Actrn, | Walking away fatigue and disease after stroke | http://www.who.int/trialsearch/Trial2.aspx?TrialID=ACTRN12617000746336 | 2017 |  |  |  | Wrong comparator - co-interventions not balanced between arms |
| Actrn 2017 | Actrn,; Hunter Medical Research Institute, | HELLEN: A robot to assist patients with acquired brain injury to stand and exercise |  | 2017 |  |  |  | Not relevant |
| Actrn 2017 | Actrn; Univeristy of Newcastle, | Determining the minimum dose of exercise required to improve cardiorespiratory fitness in stroke survivors (ExDose) |  | 2017 |  |  |  | Wrong comparator - exercise v exercise |
| Actrn 2017 | Actrn,; Medical Research Institute, South Australian, Health | A physician-led l(L)ifestyle i(I)nterv(V)entional program with goals of weight loss and e(E)xercise participation in overweight and obese patients with heart failure and reduced (REDUCED) ejection fraction |  | 2017 |  |  |  | Wrong comparator - co-interventions not balanced between arms |
| Actrn 2017 | Actrn,; Hunter Stroke Service, John Hunter Hospital | Supporting Lifestyle and activity Modification after Transient Ischaemic Attack (TIA) (and mild stroke)&#x0D |  | 2017 |  |  |  | Wrong study design - not RCT |
| Actrn 2017 | Actrn,; Meredith King, | The effect of Aquatic "water-based" exercise on exercise capacity and quality of life in people with Chronic Heart Failure and/or a chronic respiratory disease |  | 2017 |  |  |  | Not relevant |
| Actrn 2017 | Actrn; Royal Melbourne Hospital Approved 30/11/ Melbourne, Health; Professor Colin Royse, | The Supervised Early Resistance Training (SEcReT) Study: progressive resistance training following open heart surgery |  | 2017 |  |  |  | Wrong comparator - exercise v exercise |
| Actrn 2017 | Actrn,; University of Newcastle, | Breaking Up Sitting Time After Stroke (BUST-BP-Dose) |  | 2017 |  |  |  | Not relevant |
| Actrn 2018 | Actrn, | Effectiveness of a external skeletal device for the rehabilitation of patients following stroke | http://www.who.int/trialsearch/Trial2.aspx?TrialID=ACTRN12618001132235 | 2018 |  |  |  | Not relevant |
| Actrn 2018 | Actrn, | Effects of myofascial release techniques in patients after coronary revascularization | http://www.who.int/trialsearch/Trial2.aspx?TrialID=ACTRN12618000470291 | 2018 |  |  |  | Not relevant |
| Actrn 2018 | Actrn, | (M)ultifactorial (I)ntervention in Patients with (P)eripheral (A)rterial (D)isease - A Randomised Controlled Trial | https://trialsearch.who.int/Trial2.aspx?TrialID=ACTRN12618000250235 | 2018 |  |  |  | Not relevant |
| Actrn 2018 | Actrn, | A pilot study exploring the effectiveness of the Lifestyle approach to reducing Falls through Exercise (LiFE) in the stroke population | http://www.who.int/trialsearch/Trial2.aspx?TrialID=ACTRN12618001361291 | 2018 |  |  |  | Wrong comparator - exercise v exercise |
| Actrn 2018 | Actrn,; Universiti Kebangsaan Malaysia, No | Effectiveness of virtual reality games as an adjunct in improving upper limb function and general health among stroke survivors |  | 2018 |  |  |  | Wrong study design - not RCT |
| Actrn 2018 | Actrn,; University Hospital Brno, | Cardiovascular rehabilitation and teleMedicine technology |  | 2018 |  |  |  | Wrong comparator - exercise v exercise |
| Actrn 2018 | Actrn,; University of Auckland, | RELIEF: Feasibility of the Re-Link Trainer for walking rehabilitation after stroke |  | 2018 |  |  |  | Not relevant |
| Actrn 2018 | Actrn,; Macquarie University, | A Self-Managed Exercise Program for People with Stroke - THE TASK PROJECT&#x0D |  | 2018 |  |  |  | Wrong study design - not RCT |
| Actrn 2018 | Actrn,; Edith Cowan University, | Effects of a weight training exercise program of the 'good arm' on the 'bad arm' after stroke |  | 2018 |  |  |  | Wrong study design - not RCT |
| Actrn 2018 | Actrn,; University of Tasmania, | CArdiac REhabilitation for the Secondary prevention of Stroke (CARESS) |  | 2018 |  |  |  | Not relevant |
| Actrn 2019 | Actrn, | Effectiveness of Autogenic Relaxation Training in Addition to Usual Physiotherapy on Emotional State and Functional Independence of Stroke Survivors | https://trialsearch.who.int/Trial2.aspx?TrialID=ACTRN12619001664134 | 2019 |  |  |  | Not relevant |
| Actrn 2019 | Actrn, | Evaluation of the ELEMENTS tablet for post-stroke rehabilitation | https://trialsearch.who.int/Trial2.aspx?TrialID=ACTRN12619001557123 | 2019 |  |  |  | Wrong intervention - does not meet exercise definition |
| Actrn 2019 | Actrn, | Exercise as Medicine for heart failure | https://trialsearch.who.int/Trial2.aspx?TrialID=ACTRN12619000174189 | 2019 |  |  |  | Wrong comparator - exercise v exercise |
| Actrn 2019 | Actrn, | A health-coaching program for stroke survivors and their family caregivers during the hospital to home transition in Chongqing, China | https://trialsearch.who.int/Trial2.aspx?TrialID=ACTRN12619000321145 | 2019 |  |  |  | Wrong comparator - co-interventions not balanced between arms |
| Actrn 2019 | Actrn, | High-intensity interval training within cardiac rehabilitation: a multi-center randomized controlled trial | https://trialsearch.who.int/Trial2.aspx?TrialID=ACTRN12619001737123 | 2019 |  |  |  | Wrong comparator - exercise v exercise |
| Actrn 2019 | Actrn,; National Ageing Research Institute, | CardiacRehabPlus: Innovation to improve outcomes for TIA and cardiac rehabilitation patients |  | 2019 |  |  |  | Wrong study design - not RCT |
| Actrn 2020 | Actrn, | Effect of a 8 and 12-week exercise program on aerobic capacity after percutaneous revascularization in subjects with Acute Coronary Syndromes | https://trialsearch.who.int/Trial2.aspx?TrialID=ACTRN12620000363987 | 2020 |  |  |  | Wrong comparator - exercise v exercise |
| Actrn 2020 | Actrn, | iN home Telerehabilitation to ENhance phySIcal activiTY after Stroke (iNTENSITY - Stroke) | https://trialsearch.who.int/Trial2.aspx?TrialID=ACTRN12620000418976 | 2020 |  |  |  | Wrong comparator - exercise v exercise |
| Actrn 2020 | Actrn, | Increasing weight-bearing exercise to prevent bone loss in post-stroke patients using robot-assisted rehabilitation | https://trialsearch.who.int/Trial2.aspx?TrialID=ACTRN12620000083998 | 2020 |  |  |  | Not relevant |
| Actrn 2020 | Actrn, | PREDICTive value of aggressive risk factor modification on the development of atrial fibrillation in Embolic Stroke of Undetermined Source. Staging the Atrial Fibrillation Substrate: the PREDICT-ESUS study | https://trialsearch.who.int/Trial2.aspx?TrialID=ACTRN12620000771954 | 2020 |  |  |  | Not relevant |
| Actrn 2020 | Actrn, | PREDICTive value of aggressive risk factor modification on the occurrence of major cardiovascular events in patients with embolic STROKE: PREDICT-STROKE | https://trialsearch.who.int/Trial2.aspx?TrialID=ACTRN12620000704998 | 2020 |  |  |  | Not relevant |
| Actrn 2020 | Actrn, | A randomised controlled trial to evaluate an intensive lifestyle program for reversal of coronary heart disease | https://trialsearch.who.int/Trial2.aspx?TrialID=ACTRN12620001151921 | 2020 |  |  |  | Wrong comparator - co-interventions not balanced between arms |
| Actrn 2020 | Actrn, | A research study to determine the effect of a smartphone application (app) on management of patients with heart failure in isolation due to COVID-19 | https://trialsearch.who.int/Trial2.aspx?TrialID=ACTRN12620000860965 | 2020 |  |  |  | Not relevant |
| Actrn 2020 | Actrn,; University of Melbourne, | DOSE ranging in UPper limb rehabilitation post stroke (DOSE-UP) |  | 2020 |  |  |  | Not relevant |
| Actrn 2020 | Actrn,; Women's Hospital, ; Royal, Brisbane | Feasibility, Reliability and Validity of Sit to Stand Tests in People with Heart Failure |  | 2020 |  |  |  | Not relevant |
| Actrn 2021 | Actrn, | Cardiac Rehabilitation for transient ischaemic Attack and Mild-Stroke: the CRAMS randomised controlled trial | https://trialsearch.who.int/Trial2.aspx?TrialID=ACTRN12621001586808 | 2021 |  |  |  | Wrong comparator - co-interventions not balanced between arms |
| Actrn 2021 | Actrn, | The effect of game-based in comparison to conventional circuit exercise on functions, motivation level, self-efficacy and quality of life among stroke survivors | https://trialsearch.who.int/Trial2.aspx?TrialID=ACTRN12621001489886 | 2021 |  |  |  | Wrong comparator - exercise v exercise |
| Actrn 2021 | Actrn, | Effects of physical rehabilitation on muscle function and gait parameters of patients with intermittent claudication | https://trialsearch.who.int/Trial2.aspx?TrialID=ACTRN12621000780853 | 2021 |  |  |  | Wrong comparator - exercise v exercise |
| Actrn 2021 | Actrn, | A mobile health application for people in remote and rural Queensland who have experienced a cardiovascular event | https://trialsearch.who.int/Trial2.aspx?TrialID=ACTRN12621001229864 | 2021 |  |  |  | Not relevant |
| Actrn 2021 | Actrn, | Personal activity Intelligence (PAI) e-health Program for Defence Force and Ex-Service Personnel and immediate family following heart rehabilitation | https://trialsearch.who.int/Trial2.aspx?TrialID=ACTRN12621000830897 | 2021 |  |  |  | Wrong comparator - co-interventions not balanced between arms |
| Actrn 2021 | Actrn,; The Royal Melbourne Hospital, | A telehealth-supported falls prevention program for people with stroke returning home from hospital |  | 2021 |  |  |  | Wrong study design - not RCT |
| Actrn 2022 | Actrn, | The effect of a dance program in addition to the traditional physiotherapy on physical function and mobility in older people in rehabilitation with recent acquired brain injury | https://trialsearch.who.int/Trial2.aspx?TrialID=ACTRN12622000481774 | 2022 |  |  |  | Wrong comparator - active control |
| Actrn 2022 | Actrn, | Effect of a targeted exercise intervention in post-stroke older people | https://trialsearch.who.int/Trial2.aspx?TrialID=ACTRN12622000169741 | 2022 |  |  |  | Wrong intervention - does not meet exercise definition |
| Actrn 2022 | Actrn, | The effects of a 12-week telehealth delivered environmental enrichment program for young stroke survivors | https://trialsearch.who.int/Trial2.aspx?TrialID=ACTRN12622000599774 | 2022 |  |  |  | Wrong comparator - co-interventions not balanced between arms |
| Actrn 2022 | Actrn, | Effects of Virtual Exercise Gaming on Upper Limb Function in Stroke Survivors | https://trialsearch.who.int/Trial2.aspx?TrialID=ACTRN12622000363785 | 2022 |  |  |  | Wrong comparator - active control |
| Actrn 2022 | Actrn, | Evaluation of an Educational Intervention In Cardiac Rehabilitation Through Whatsapp Using A Randomized Controlled Clinical Trial | https://trialsearch.who.int/Trial2.aspx?TrialID=ACTRN12622001446752 | 2022 |  |  |  | Not relevant |
| Actrn 2022 | Actrn, | Investigating telehealth delivery of exercise prescription in regional cardiac rehabilitation programs | https://trialsearch.who.int/Trial2.aspx?TrialID=ACTRN12622000872730 | 2022 |  |  |  | Wrong comparator - exercise v exercise |
| Actrn 2022 | Actrn, | A micro randomised trial (MRT) to test the effects of app-based motivational messages on physical activity and heart rate variability | https://trialsearch.who.int/Trial2.aspx?TrialID=ACTRN12622000731796 | 2022 |  |  |  | Wrong comparator - co-interventions not balanced between arms |
| Actrn 2022 | Actrn, | PeRsonalised Exercise for Priming Post-stroke (PREPP): a randomised trial | https://trialsearch.who.int/Trial2.aspx?TrialID=ACTRN12622000092796 | 2022 |  |  |  | Wrong intervention < 6 weeks |
| Actrn 2022 | Actrn, | Trial of an Individualised Intervention for the Prevention of Stroke (TIIPS)- using health and wellness coaching | https://trialsearch.who.int/Trial2.aspx?TrialID=ACTRN12622000939796 | 2022 |  |  |  | Not relevant |
| Actrn 2022 | Actrn,; Australian Catholic University, | Using muscle strength as a predictor for walking ability after stroke |  | 2022 |  |  |  | Not relevant |
| Actrn 2022 | Actrn,; The University of Melbourne, | Fit 4 Me After Stroke (Mild) |  | 2022 |  |  |  | Not relevant |
| Actrn 2022 | Actrn,; The University of Melbourne, | Fit 4 Me After Stroke (Moderate) |  | 2022 |  |  |  | Not relevant |
| Actrn 2023 | Actrn, | Community-based cardiac rehabilitation for Chinese migrants in Australia | https://trialsearch.who.int/Trial2.aspx?TrialID=ACTRN12623000175673 | 2023 |  |  |  | Wrong comparator - co-interventions not balanced between arms |
| Actrn 2023 | Actrn, | Does the addition of high-intensity single muscle group training improve exercise training efficiency in heart failure? | https://trialsearch.who.int/Trial2.aspx?TrialID=ACTRN12623000055606 | 2023 |  |  |  | Wrong comparator - exercise v exercise |
| Actrn 2023 | Actrn, | The effect of non invasive auricular vagus nerve stimulation (taVNS) combined with physiotherapy on mobility and balance impairment after stroke | https://trialsearch.who.int/Trial2.aspx?TrialID=ACTRN12623000376640 | 2023 |  |  |  | Not relevant |
| Actrn 2023 | Actrn, | Kicking Goals for Men?s Heart Health: a Multi-State/Territory Trial of the Aussie-FIT Program | https://trialsearch.who.int/Trial2.aspx?TrialID=ACTRN12623000437662 | 2023 |  |  |  | Not relevant |
| Actrn 2023 | Actrn, | A mobility booster program (HiWalk) in long-term community stroke rehabilitation | https://trialsearch.who.int/Trial2.aspx?TrialID=ACTRN12623000316606 | 2023 |  |  |  | Wrong intervention < 6 weeks |
| Actrn 2023 | Actrn, | A risk-guided strategy for Acute Decompensated Heart Failure using mHealth | https://trialsearch.who.int/Trial2.aspx?TrialID=ACTRN12623001142628 | 2023 |  |  |  | Not relevant |
| Actrn 2023 | Actrn, | Saving Legs & Lives: cardiovascular rehabilitation following leg vascular surgery | https://trialsearch.who.int/Trial2.aspx?TrialID=ACTRN12623000190606 | 2023 |  |  |  | Wrong comparator - co-interventions not balanced between arms |
| Actrn 2023 | Actrn, | The Supervised Home-based exercise program for Peripheral Artery Disease Trial | https://trialsearch.who.int/Trial2.aspx?TrialID=ACTRN12623000421639 | 2023 |  |  |  | Wrong comparator - exercise v exercise |
| Actrn 2023 | Actrn,; Ms Tanya Palmer Approved 12/01/ Metro North Health Human Research Ethics Committee, B.; Professor Norman Morris, No | Does the Dyspnea Challenge detect changes in exertional breathlessness? |  | 2023 |  |  |  | Not relevant |
| Adams 2023 | Adams, R. J.; Ellington, A. L.; Kuccera, K. A.; Leaman, H.; Smithson, C.; Patrie, J. T. | Telehealth-Guided Virtual Reality for Recovery of Upper Extremity Function Following Stroke | OTJR : occupation, participation and health | 2023 | 43 | 3 | 446-456 | Wrong intervention - does not meet exercise definition |
| Adans-Dester 2020 | Adans-Dester, C.; Fasoli, S. E.; Fabara, E.; Menard, N.; Fox, A. B.; Severini, G.; Bonato, P. | Can kinematic parameters of 3D reach-to-target movements be used as a proxy for Clinical outcome measures in chronic stroke rehabilitation? An exploratory study | Journal of Neuroengineering & Rehabilitation | 2020 | 17 | 1 | 106 | Not relevant |
| Aday 2019 | Aday, A. W.; Kinlay, S.; Gerhard-Herman, M. D. | Reply to: 'Post-exercise criteria to diagnose lower extremity peripheral artery disease: Which one should I use in my Practice?' by Stivalet et al | Vascular Medicine (United Kingdom) | 2019 | 24 | 1 | 78 | Not relevant |
| Addison 2017 | Addison, O.; Ryan, A. S.; Prior, S. J.; Katzel, L. I.; Kundi, R.; Lal, B. K.; Gardner, A. W. | Changes in Function After a 6-Month Walking Intervention in Patients With Intermittent Claudication Who Are Obese or Nonobese | Journal of Geriatric Physical Therapy | 2017 | 40 | 4 | 190-196 | Not relevant |
| Adie 2017 | Adie, K.; Schofield, C.; Berrow, M.; Wingham, J.; Humfryes, J.; Pritchard, C.; James, M.; Allison, R. | Does the use of Nintendo Wii SportsTM improve arm function? Trial of WiiTM in Stroke: a randomized controlled trial and economics analysis | Clinical Rehabilitation | 2017 | 31 | 2 | 173-185 | Wrong intervention - does not meet exercise definition |
| Adjetey 2023 | Adjetey, C.; Davis, J.; Eng, J.; Falck, R. S.; Dao, E.; Best, J. R.; Bennett, K.; McGuire, K.; Hu, M.; Hsiung, G. Y. R.; Middleton, L. E.; Graf, P.; Sakakibara, B.; Liu-Ambrose, T. | EE458 Economic Evaluation of Lifestyle Interventions to Promote Cognition in Older Adults with Chronic Stroke | Value in health | 2023 | 26 | 6 | S143 | Wrong comparator - active control |
| Adjetey 2023 | Adjetey, C.; Davis, J. C.; Falck, R. S.; Best, J. R.; Dao, E.; Bennett, K.; Tai, D.; McGuire, K.; Eng, J. J.; Hsiung, G. R.; Middleton, L. E.; Hall, P. A.; Hu, M.; Sakakibara, B. M.; Liu-Ambrose, T. | Economic Evaluation of Exercise or Cognitive and Social Enrichment Activities for Improved Cognition After Stroke | JAMA Network Open | 2023 | 6 | 11 | e2345687 | Wrong comparator - active control |
| Adsett 2017 | Adsett, J.; Morris, N.; Kuys, S.; Hwang, R.; Mullins, R.; Khatun, M.; Paratz, J.; Mudge, A. | Aquatic Exercise Training is Effective in Maintaining Exercise Performance in Trained Heart Failure Patients: A Randomised Crossover Pilot Trial | Heart, Lung & Circulation | 2017 | 26 | 6 | 572-579 | Wrong comparator - exercise v exercise |
| Adsett 2019 | Adsett, J. A.; Morris, N. R.; Mudge, A. M. | Predictors of Exercise Training and physical activity Adherence in People Recently Hospitalized With Heart Failure: A BRIEF REPORT | Journal of Cardiopulmonary Rehabilitation & Prevention | 2019 | 39 | 3 | E12-E16 | Wrong comparator - active control |
| Adsett 2021 | Adsett, J. A.; Morris, N. R.; Mudge, A. M. | Impact of exercise training program attendance and physical activity participation on six minute walk distance in patients with heart failure | Physiotherapy Theory & Practice | 2021 | 37 | 9 | 1051-1059 | Wrong comparator - active control |
| Afzal 2018 | Afzal, M. R.; Pyo, S.; Oh, M. K.; Park, Y. S.; Yoon, J. | Evaluating the effects of delivering integrated kinesthetic and tactile cues to individuals with unilateral hemiparetic stroke during overground walking | Journal of Neuroengineering & Rehabilitation | 2018 | 15 | 1 | 33 | Not relevant |
| Agapitou 2018 | Agapitou, V.; Tzanis, G.; Dimopoulos, S.; Karatzanos, E.; Karga, H.; Nanas, S. | Effect of combined endurance and resistance training on exercise capacity and serum anabolic steroid concentration in patients with chronic heart failure | Hjc Hellenic Journal of Cardiology | 2018 | 59 | 3 | 179-181 | Not relevant |
| Aggarwal 2022 | Aggarwal, D.; Trivedi, V.; Goyal, D.; Mahajan, R. | Effect of Distal Irradiation and Task Related Circuit Training on Locomotor Tasks in Post Stroke Hemiparetic Patients | NeuroQuantology | 2022 | 20(5) |  | 4840-4848 | Wrong intervention - does not meet exercise definition |
| Aghamohammadi 2019 | Aghamohammadi, T.; Khaleghipour, M.; Shahboulaghi, F.; Dalvandi, A.; Maddah, S. | Effect of self-management program on health status of elderly patients with heart failure: a single-blind, randomized Clinical trial | Journal of Acute Disease | 2019 | 8 | 5 | 179-184 | Not relevant |
| Aguiar 2018 | Aguiar, L.; Nadeau, S.; Britto, R.; Teixeira-Salmela, L.; Martins, J.; Quintino, L.; Brito, S.; Garcia, L.; Reis, M. T.; Faria, C. | Effects of aerobic training on physical activity in people with stroke: Preliminary results of a randomized controlled trial | International Journal of Stroke | 2018 | 13(2) | 1 | 175 | Wrong comparator - exercise v exercise |
| Aguiar 2018 | Aguiar, L. T.; Nadeau, S.; Britto, R. R.; Teixeira-Salmela, L. F.; Martins, J. C.; Faria, Cdcm | Effects of aerobic training on physical activity in people with stroke: protocol for a randomized controlled trial | Trials [Electronic Resource] | 2018 | 19 | 1 | 446 | Wrong comparator - exercise v exercise |
| Aguiar 2020 | Aguiar, L. T.; Nadeau, S.; Britto, R. R.; Teixeira-Salmela, L. F.; Martins, J. C.; Samora, G. A. R.; da Silva Junior, J. A.; Faria, Cdcm | Effects of aerobic training on physical activity in people with stroke: A randomized controlled trial | NeuroRehabilitation | 2020 | 46 | 3 | 391-401 | Wrong comparator - exercise v exercise |
| Aguilar-Ferrándiz 2021 | Aguilar-Ferrándiz, Marí­a Encarnación; Toledano-Moreno, Sonia; Garcí­a-Rí­os, Marí­a Carmen; Tapia-Haro, Rosa Marí­a; Barrero-Hernández, Francisco Javier; Casas-Barragán, Antonio; Pérez-Mármol, José Manuel | Effectiveness of a Functional Rehabilitation Program for Upper Limb Apraxia in Poststroke Patients: A Randomized Controlled Trial | Archives of Physical Medicine & Rehabilitation | 2021 | 102 | 5 | 940-950 | Not relevant |
| Aguilar-Ferrándiz 2022 | Aguilar-Ferrándiz, M. E.; Toledano-Moreno, S.; Garcí­a-Rí­os, M. C.; Tapia-Haro, R. M.; Barrero-Hernández, F. J.; Casas-Barragán, A.; Pérez-Mármol, J. M. | Response to Letter to the Editor: effectiveness of a Functional Rehabilitation Program for Upper Limb Apraxia in Poststroke Patients: a Randomized Controlled Trial | Archives of Physical Medicine and Rehabilitation | 2022 | 103 | 5 | 1047 | Not relevant |
| Aguirre 2018 | Aguirre, L. G.; Urrunaga-Pastor, D.; Lazo-Porras, M.; Taype-Rondan, A. | Post-stroke rehabilitation devices offered via the Internet: Based on randomized controlled evidence? | Annals of Physical and Rehabilitation Medicine | 2018 | 61 | 1 | 54-55 | Not relevant |
| Ahc 2018 | Ahc, Media | CT Calcium Score vs. Stress Testing | Internal Medicine Alert | 2018 | 40 | 2 | 11-12 | Not relevant |
| Ahmad 2023 | Ahmad, A. M.; Elshenawy, A. I.; Abdelghany, M.; Elghaffar, H. A. A. | Effects of early mobilisation program on functional capacity, daily living activities, and N-terminal prohormone brain natriuretic peptide in patients hospitalised for acute heart failure. A randomised controlled trial | Hong Kong Physiotherapy Journal | 2023 | 43 | 1 | 19-31 | Wrong intervention < 6 weeks |
| Ahmad 2022 | Ahmad, A. M.; Hassan, M. H. | Effects of Addition of Inspiratory Muscle Training to Exercise-Based Cardiac Rehabilitation on Inspiratory Muscle Strength, Peak Oxygen Consumption, and Selected Hemodynamics in Chronic Heart Failure | Acta Cardiologica Sinica | 2022 | 38 | 4 | 485-494 | Not relevant |
| Ahmad 2021 | Ahmad, A. M.; Mahmoud, R. R. | An eight-week pulsed electromagnetic field improves physical functional performance and ankle-brachial index in men with Fontaine stage II peripheral artery disease | Advances in Rehabilitation | 2021 | 35 | 4 | 1-8 | Not relevant |
| Ahmad 2022 | Ahmad, F.; Fountotos, R.; Bharaj, N.; Munir, H.; Hagerty, K.; Hedjazi, M.; Marsala, J.; Rudski, L. G.; Goldfarb, M.; Afilalo, J. | MULTICOMPONENT GERIATRIC INTERVENTION TO DE-FRAIL HOSPITALIZED OLDER ADULTS WITH CARDIOVASCULAR DISEASE: SUBSTUDY OF THE TARGET-EFT RANDOMIZED CLINICAL TRIAL | Journal of the American College of Cardiology | 2022 | 79 | 9 | 1592 | Wrong intervention < 6 weeks |
| Ahmad 2023 | Ahmad, F.; Fountotos, R.; Goldfarb, M.; Bharaj, N.; Munir, H.; Marsala, J.; Rudski, L. G.; Afilalo, J. | De-frailing intervention for hospitalized cardiovascular patients in the TARGET-EFT randomized Clinical trial | European Heart Journal. Quality of care & Clinical outcomes | 2023 | 9 | 5 | 482-489 | Wrong intervention < 6 weeks |
| Ahmadi 2019 | Ahmadi, M.; Laumeier, I.; Ihl, T.; Steinicke, M.; Ferse, C.; Endres, M.; Grau, A.; Hastrup, S.; Poppert, H.; Palm, F.; Schoene, M.; Seifert, C.; Weber, J. E.; Von Weitzel-Mudersbach, P.; Wimmer, M.; Kandil, F. I.; Algra, A.; Amarenco, P.; Greving, J. P.; Busse, O.; Kohler, F.; Marx, P.; Audebert, H. J. | Effects of a multicomponent support program for intensified secondary prevention in patients with transient ischemic attack and minor stroke the inspire-TMS trial | European Stroke Journal | 2019 | 4 |  | 782 | Not relevant |
| Ahmadizad 2016 | Ahmadizad, S.; Nouri-Habashi, A.; Rahmani, H.; Maleki, M.; Naderi, N.; Lotfian, S.; Salimian, M. | Platelet activation and function inÂ responseÂ to high intensity interval exercise and moderate continuous exerciseÂ in CABG and PCI patients | Clinical Hemorheology and Microcirculation | 2016 | 64 | 4 | 911-919 | Not relevant |
| AhmedBurq 2021 | Ahmed Burq, H. S. I.; Karimi, H.; Ahmad, A.; Gilani, S. A.; Hanif, A. | Effect of whole-body vibration on obstacle clearance and stair negotiation time in chronic stroke patients; A randomized controlled trial | Journal of Bodywork and Movement Therapies | 2021 | 27 |  | 698-704 | Not relevant |
| Ahmed 2018 | Ahmed, G. M.; El Gohary, A. M.; Al-Azab, I. M.; Marzouk, S.; Youssef, T. M. | Effect of transcranial direct current stimulation on gait of stroke patients | Clinical neurophysiology | 2018 | 129 |  | e122 | Not relevant |
| Ahmed 2023 | Ahmed, G. M.; Fahmy, E. M.; Ibrahim, M. F.; Nassief, A. A.; Elshebawy, H.; Mahfouz, M. M.; Elzanaty, M. Y. | Efficacy of rhythmic auditory stimulation on gait parameters in hemiplegic stroke patients: a randomized controlled trial | Egyptian Journal of Neurology, Psychiatry and Neurosurgery | 2023 | 59 | 1 |  | Not relevant |
| Ahmed 2022 | Ahmed, I.; Mustafaoglu, R.; Erhan, B. | The Effects of Low-intensity Resistance Training with Blood Flow Restriction Versus Traditional Resistance Exercise on Lower Extremity Muscle Strength, Walking Capacity, and Balance in Ischemic Stroke Survivors: A Study Protocol for the BFR-Stroke RESILIENCE Trial | Haseki Tip Bulteni | 2022 | 60(4) |  | 287-294 | Wrong intervention < 6 weeks |
| Ahmed 2023 | Ahmed, I.; Mustafaoglu, R.; Erhan, B. | The effects of low-intensity resistance training with blood flow restriction versus traditional resistance exercise on lower extremity muscle strength and motor functionin ischemic stroke survivors: a randomized controlled trial | Topics in Stroke Rehabilitation | 2023 |  |  | 1-12 | Wrong intervention < 6 weeks |
| AhmedIbrahim 2017 | Ahmed Ibrahim, M.; Mousa Ahmed, G.; Mohamed Fahmy, E. | Effect of rhythmic auditory stimulation on gait in patients with stroke | Neurology. Conference: 69th American Academy of Neurology Annual Meeting, AAN | 2017 | 88 | 16(1) |  | Not relevant |
| Ahmed 2018 | Ahmed, K.; Hernon, S.; Mohammed, S.; Tubassum, M.; Newell, M.; Walsh, S. | Remote ischemic preconditioning in the management of intermittent claudication randomized controlled trial | Irish Journal of Medical Science | 2018 | 187 | 4 | S163 | Wrong intervention < 6 weeks |
| Ahmed 2019 | Ahmed, K. M. T.; Hernon, S.; Mohamed, S.; Tubassum, M.; Newell, M.; Walsh, S. R. | Remote Ischemic Pre-conditioning in the Management of Intermittent Claudication: A Pilot Randomized Controlled Trial | Annals of Vascular Surgery | 2019 | 55 |  | 122-130 | Wrong intervention < 6 weeks |
| Ahmed 2021 | Ahmed, S. I.; Obaya, H. E.; Elhamed, S. S. A.; Elbanna, R. H. | The effect of the warm whirlpool on peripheral arterial insufficiency in lower limb | Turkish Journal of Physiotherapy and Rehabilitation | 2021 | 32 | 3 | 5790-5797 | Not relevant |
| Ahmed 2021 | Ahmed, U.; Karimi, H.; Amir, S.; Ahmed, A. | Effects of intensive multiplanar trunk training coupled with dual-task exercises on balance, mobility, and fall risk in patients with stroke: a randomized controlled trial | Journal of International Medical Research | 2021 | 49 | 11 | 1-20 | Wrong intervention - does not meet exercise definition |
| Ahmedy 2022 | Ahmedy, F.; Mohamad Hashim, N.; Lago, H.; Plijoly, L. P.; Ahmedy, I.; Idna Idris, M. Y.; Gani, A.; Sybil Shah, S.; Chia, Y. K. | Comparing Neuroplasticity Changes Between High and Low Frequency Gait Training in Subacute Stroke: Protocol for a Randomized, Single-Blinded, Controlled Study | JMIR Research protocols | 2022 | 11 | 1 | e27935 | Wrong intervention - does not meet exercise definition |
| Ahn 2019 | Ahn, J.; Lee, B. J.; Kim, B. K.; Kim, J. Y. | The role of cardiac rehabilitation in patients undergoing pacemaker implantation: a preliminary data from single center randomized controlled trial | Journal of Arrhythmia | 2019 | 35 |  | 379 | Wrong population - under 80% vascular |
| Ahn 2019 | Ahn, S. N. | Effectiveness of occupation-based interventions on performance's quality for hemiparetic stroke in community-dwelling: A randomized Clinical trial study | NeuroRehabilitation | 2019 | 44 | 2 | 275-282 | Not relevant |
| Ain 2021 | Ain, Q. U.; Khan, S.; Ilyas, S.; Yaseen, A.; Tariq, I.; Liu, T.; Wang, J. | Additional Effects of Xbox Kinect Training on Upper Limb Function in Chronic Stroke Patients: A Randomized Control Trial | Healthcare (Basel, Switzerland) | 2021 | 9 | 3 |  | Not relevant |
| Ain 2022 | Ain, Q. U. L.; Hassan, Z.; Ashraf, S.; Mahjabeen, H.; Kousar, F.; Waris, M.; Waris, S. | Comparison Between Effects of Functional Training Program and Conventional Therapy on Postural Control and Functional Mobility in Chronic Stroke | Pakistan Journal of Medical and Health Sciences | 2022 | 16 | 2 | 610-613 | Wrong intervention - does not meet exercise definition |
| Ajiboye 2013 | Ajiboye, O. A.; Anigbogu, C. N.; Ajuluchukwu, J. N.; Jaja, S. I. | Therapeutic Effects of Exercise Training On Selected Cardio-Pulmonary Parameters and Body Composition of Nigerians with Chronic Heart Failure (A Preliminary Study) | Nigerian Quarterly Journal of Hospital Medicine | 2013 | 23 | 4 | 295-301 | Not relevant |
| Akerman 2019 | Akerman, A. P.; Thomas, K. N.; van Rij, A. M.; Body, E. D.; Alfadhel, M.; Cotter, J. D. | Heat therapy vs. supervised exercise therapy for peripheral arterial disease: a 12-wk randomized, controlled trial | American Journal of Physiology - Heart & Circulatory Physiology | 2019 | 316 | 6 | H1495-H1506 | Wrong comparator - active control |
| Akkus 2020 | Akkus, O.; Huzmeli, I.; Bekler, O.; Yalcin, F.; Ozer, A. Y. | Effects of inspiratory muscle training on cardiac functions, exercise capacity and functional capacity in patients with stable angina: preliminary results | Anatolian Journal of Cardiology | 2020 | 24 | 1 | 81-82 | Wrong intervention - does not meet exercise definition |
| Akter 2019 | Akter, R.; Ahmed, S.; Chatterjee, S. | Comments on: does electromyographic biofeedback improve exercise effects in hemiplegic patients? A pilot randomized controlled trial | Journal of Rehabilitation Medicine | 2019 | 51 | 6 | 471 | Not relevant |
| Al-Hchaim 2023 | Al-Hchaim, Mohammed Hakim Shamran; Mohammed, Shatha Saadi | Effectiveness of Regular Resistance Exercise on Muscle Strength of Patients with Stroke | HIV Nursing | 2023 | 23 | 3 | 388-396 | Wrong study design - not RCT |
| Al-Khazraji 2018 | Al-Khazraji, B. K.; Lingum, N. R.; Vording, J. L.; Matushewski, B. J.; Shoemaker, J. K. | Cerebrovascular control after cardiac rehabilitation in ischemic heart disease patients | FASEB Journal. Conference: Experimental Biology | 2018 | 32 | 1(1) |  | Wrong study design - not RCT |
| Al-Lamee 2020 | Al-Lamee, R.; Jacobs, A. K. | ISCHEMIA trial: was it worth the wait? | Circulation | 2020 | 142 | 6 | 517-519 | Not relevant |
| Alaca 2022 | Alaca, N.; Ocal, N. M. | Proprioceptive based training or modified constraint-induced movement therapy on upper extremity motor functions in chronic stroke patients: A randomized controlled study | NeuroRehabilitation | 2022 | 51 | 2 | 271-282 | Not relevant |
| Albart 2021 | Albart, S. A.; Loh, H. C.; Chin, M.; Looi, I. | P-OT016. The comparison of robotic glove training and conventional training in post-stroke hand impairment patients: a pilot randomized controlled trial protocol | Clinical neurophysiology | 2021 | 132 | 8 | e126 | Wrong intervention - does not meet exercise definition |
| Albustami 2023 | Albustami, M.; Hartfiel, N.; Charles, J. M.; Powell, R.; Begg, B.; Birkett, S. T.; Nichols, S.; Ennis, S.; Hee, S. W.; Banerjee, P.; Ingle, L.; Shave, R.; McGregor, G.; Edwards, R. T. | Cost-effectiveness of High-Intensity Interval Training (HIIT) vs Moderate Intensity Steady-State (MISS) Training in UK Cardiac Rehabilitation | Archives of Physical Medicine and Rehabilitation | 2023 |  |  |  | Not relevant |
| Alcala 2023 | Alcala, University of; Cajal, Hospital Universitario Ramon y | Effectiveness of Physiotherapy After Stroke |  | 2023 |  |  |  | Wrong intervention - does not meet exercise definition |
| Alcantara 2015 | Alcantara, C. C.; Silva-Couto, M. A.; Prado-Medeiros, C. L.; Salvini, T. F.; Russo, T. L. | Evaluation of knee flexors and extensors isokinetic torque and IGF-1/IGFBP-3 concentrations after eccentric training in chronic hemiparetic subjects | Gait and Posture | 2015 | 42 | 3 | S58-S59 | Wrong study design - not RCT |
| Alegre 2019 | Alegre, Federal University of Health Science of Porto | Mirror Therapy in Sensorimotor Recovery of Paretic Upper Extremity After Chronic Stroke |  | 2019 |  |  |  | Not relevant |
| Alegre 2020 | Alegre, Federal University of Health Science of Porto | Effects of Robotic Rehabilitation in Post-Stroke Patients |  | 2020 |  |  |  | Not relevant |
| Alegre 2019 | Alegre, Hospital de Clinicas de Porto | Effects of Neuromuscular Electrical Stimulation in Muscle Architecture and Functionality of Patients After Acute Stroke |  | 2019 |  |  |  | Not relevant |
| Alegre 2021 | Alegre, Hospital de Clinicas de Porto | Aerobic Exercise and Telomere Length in Patients With Heart Failure |  | 2021 |  |  |  | Wrong comparator - exercise v exercise |
| Alegret 2017 | Alegret, J. M.; Martinez-Micaelo, N.; La Gerche, A.; Franco-Bonafonte, L.; Rubio-Perez, F.; Calvo, N.; Montero, M. | Acute effect of static exercise in patients with aortic regurgitation assessed by cardiovascular magnetic resonance: role of left ventricular remodelling | European Radiology | 2017 | 27 | 4 | 1424-1430 | Not relevant |
| AlexandraLima 2019 | Alexandra Lima, Acgb; Silva, N. T.; Lira, A. O. V.; Negrao, E. M.; Avilla, Lbod; Cipriano, G. | The effect of high intensity interval training on left atrial volume index in heart failure patients | European Journal of Preventive Cardiology | 2019 | 26 |  | S34 | Wrong comparator - exercise v exercise |
| AlexandraLima 2019 | Alexandra Lima, Acgb; Silva, N. T.; Lira, A. O. V.; Negrao, E. M.; Cipriano, G. | The behavior of cardiorespiratory capacity after a semi-supervised cardiovascular rehabilitation program in heart failure patients-A pilot study | European Journal of Preventive Cardiology | 2019 | 26 |  | S41 | Wrong comparator - exercise v exercise |
| AlexandraLima 2017 | Alexandra Lima, A. C. G. B.; Silva, F. M. F.; Teixeira, F. M.; Nakata, C. H.; Thomaz, S. R.; Missias, A. A.; Ramalho, S. H. R.; Melo, P. F.; Freitas, L. A. O.; Cipriano, G. | Novel cardiac rehabilitation program modalities - High intensity interval training (HIIT) and circuit resistance training (CRT) - Improve cardiorespiratory fitness with safety and compliance | European Journal of Preventive Cardiology | 2017 | 24 | 1 | S150 | Wrong study design - not RCT |
| Ali 2022 | Ali, A. A. S.; Elhady, Aaea; Elaskry, N.; Elnahhas, N. G. | SHOCK WAVE VERSUS DYNAMIC TRAINING FOR INTERMITTENT CLAUDICATION IN DIABETIC PATIENTS TYPE II | Journal of Pharmaceutical Negative Results | 2022 | 13 |  | 5234-5241 | Wrong comparator - co-interventions not balanced between arms |
| Ali 2020 | Ali, M.; Khan, S. U.; Asim, H. A. B. | Effects of individual task specific training verses group circuit training on balance and ambulation in sub-acute stroke | Rawal Medical Journal | 2020 | 45 | 1 | 233-235 | Wrong comparator - exercise v exercise |
| Alibakhshi 2016 | Alibakhshi, H.; Samaei, A.; Khalili, M. A.; Siminghalam, M. | A comparetive study on the effects of mirror therapy and bilateral arm training on hand function of chronichemiparetic patients. [Persian] | Koomesh | 2016 | 17 | 3 | 589-595 | Full text not in English |
| Alim 2016 | Alim, M.; Lindley, R.; Felix, C.; Gandhi, D.B.C.; Verma, S.J.; Tugnawat, D.K.; Syrigapu, A.; Anderson, C.S; Ramamurthy, R.K.; et al. | Family-led rehabilitation after stroke in India: the ATTEND trial, study protocol for a randomized controlled trial | Trials | 2016 | 17 |  | 13-13 | Wrong intervention - does not meet exercise definition |
| Alipsatici 2020 | Alipsatici, C.; Alaca, N.; Canbora, M. K. | Comparison of the effects of treadmill trainings on walking and balance functions by increasing the speed and incline in chronic patients with stroke | Turk noroloji dergisi [Turkish Journal of neurology] | 2020 | 26 | 4 | 316-321 | Wrong comparator - exercise v exercise |
| Alisar 2020 | Alisar, D. C.; Ozen, S.; Sozay, S. | Effects of Bihemispheric Transcranial Direct Current Stimulation on Upper Extremity Function in Stroke Patients: a randomized Double-Blind Sham-Controlled Study | Journal of Stroke and Cerebrovascular Diseases | 2020 | 29 | 1 | 104454 | Not relevant |
| AlkanKayhan 2024 | Alkan Kayhan, S.; Nural, N. | The effect of web-based education delivered as part of cardiac rehabilitation on healthy lifestyle behaviors, and quality of life in patients with coronary artery disease in Turkey: a randomised controlled trial | Patient Education and Counseling | 2024 | 119 |  | 108082 | Not relevant |
| Allahbakhshian 2023 | Allahbakhshian, A.; Khalili, A. F.; Gholizadeh, L.; Esmealy, L. | Comparison of early mobilization protocols on postoperative cognitive dysfunction, pain, and length of hospital stay in patients undergoing coronary artery bypass graft surgery: A randomized controlled trial | Applied Nursing Research | 2023 | 73 |  | 151731 | Not relevant |
| Allan 2023 | Allan, L.; Silvera-Tawil, D.; Cameron, J.; Li, J.; Smallbon, V.; Varnfield, M.; Bomke, J.; Redd, C.; Lannin, N. A.; Olaiya, M. T.; Cadilhac, D. A. | A feasibility study of a multicomponent digital Care Assistant and support Program for people after Stroke or transient ischaemic attack (CAPS) | International Journal of Stroke | 2023 | 18 | 2 | 64-65 | Not relevant |
| Almeria 2024 | Almeria, Universidad de; Ministerio de Ciencia e Innovación, Spain; Union, European | Virtual Reality Software for Patients With Stroke |  | 2024 |  |  |  | Not relevant |
| Almhdawi 2016 | Almhdawi, K. A.; Mathiowetz, V. G.; White, M.; delMas, R. C. | Efficacy of Occupational Therapy Task-oriented Approach in Upper Extremity Post-stroke Rehabilitation | Occupational Therapy International | 2016 | 23 | 4 | 444-456 | Wrong intervention - does not meet exercise definition |
| Aloghareh 2021 | Aloghareh, S. B.; Salehi Tali, S.; Hasanpour Dehkordi, A.; Gangi, H.; Sedehi, M. | The effects of a self-management program based on 5 A`s model on the quality of life and self-efficacy in the myocardial infarction patients | Przeglad Epidemiologiczny | 2021 | 75 | 4 | 556-563 | Not relevant |
| Alonso 2022 | Alonso, W.; Kupzyk, K.; Norman, J.; Lundgren, S.; Lindsey, M.; Fisher, A.; Bills, S.; Keteyian, S.; Pozehl, B. | Long-term Adherence To Exercise In Adults With Preserved Ejection Fraction Heart Failure | Journal of Cardiac Failure | 2022 | 28 | 5 | S5-S6 | Not relevant |
| Alonso 2023 | Alonso, W. W.; Bills, S.; Lundgren, S.; Norman, J.; Keteyian, S. J.; Fisher, A.; Kupzyk, K.; Zheng, C.; Wilson, F. A.; Salahshurian, E.; Pozehl, B. J. | Study Protocol: heart Camp Connect - Promoting Adherence to Exercise in Adults With Heart Failure With Preserved Ejection Fraction | Circulation | 2023 | 148 |  |  | Wrong comparator - exercise v exercise |
| Alonso 2021 | Alonso, W. W.; Kupzyk, K.; Norman, J.; Bills, S. E.; Bosak, K.; Dunn, S. L.; Deka, P.; Pozehl, B. | Negative Attitudes, Self-efficacy, and Relapse Management Mediate Long-Term Adherence to Exercise in Patients With Heart Failure | Annals of Behavioral Medicine | 2021 | 55 | 10 | 1031-1041 | Not relevant |
| Alonso 2021 | Alonso, W. W.; Kupzyk, K.; Norman, J. F.; Lundgren, S.; Faulkner, K.; Keteyian, S. J.; Bills, S.; Pozehl, B. J. | Predictors of long-term adherence to exercise in adults with heart failure enrolled in an exercise Clinical trial | Circulation | 2021 | 144 | 1 |  | Not relevant |
| Alonso 2022 | Alonso, W. W.; Kupzyk, K. A.; Norman, J. F.; Lundgren, S. W.; Fisher, A.; Lindsey, M. L.; Keteyian, S. J.; Pozehl, B. J. | The HEART Camp Exercise Intervention Improves Exercise Adherence, physical Function, and Patient-Reported Outcomes in Adults With Preserved Ejection Fraction Heart Failure | Journal of Cardiac Failure | 2022 | 28 | 3 | 431-442 | Not relevant |
| Aloraini 2022 | Aloraini, S. M. | Effects of constraint-induced movement therapy for the lower extremity among individuals post-stroke: A randomized controlled Clinical trial | NeuroRehabilitation | 2022 | 51 | 3 | 421-431 | Not relevant |
| Alshamari 2023 | Alshamari, M.; Kourek, C.; Sanoudou, D.; Delis, D.; Dimopoulos, S.; Rovina, N.; Nanas, S.; Karatzanos, E.; Philippou, A. | Does the Addition of Strength Training to a High-Intensity Interval Training Program Benefit More the Patients with Chronic Heart Failure? | Reviews in Cardiovascular Medicine | 2023 | 24 | 1 |  | Wrong comparator - exercise v exercise |
| Alsubiheen 2022 | Alsubiheen, A. M.; Choi, W.; Yu, W.; Lee, H. | The Effect of Task-Oriented Activities Training on Upper-Limb Function, Daily Activities, and Quality of Life in Chronic Stroke Patients: A Randomized Controlled Trial | International Journal of Environmental Research & Public Health [Electronic Resource] | 2022 | 19 | 21 | 29 | Wrong intervention - does not meet exercise definition |
| Alvarenga 2023 | Alvarenga, M. T. M.; Ada, L.; Preston, E.; Caetano, L. C. G.; Teixeira-Salmela, L. F.; Scianni, A. A. | Home-based self-management for sedentary individuals with mild walking disability after stroke: protocol for a randomised pilot study | BMC Neurology | 2023 | 23 | 1 | 412 | Not relevant |
| Alvarez 2015 | Alvarez, O. M.; Wendelken, M. E.; Markowitz, L.; Comfort, C. | Effect of High-pressure, Intermittent Pneumatic Compression for the Treatment of Peripheral Arterial Disease and Critical Limb Ischemia in Patients Without a Surgical Option | Wounds-A Compendium of Clinical Research & Practice | 2015 | 27 | 11 | 293-301 | Wrong intervention - does not meet exercise definition |
| Alwhaibi 2022 | Alwhaibi, Reem M.; Mahmoud, Noha F.; Zakaria, Hoda M.; Ragab, Walaa M.; Al Awaji, Nisreen N.; Elserougy, Hager R. | Effect of compressive therapy on sensorimotor function of the more affected upper extremity in chronic stroke patients: A randomized Clinical trial | Medicine | 2022 | 101 | 38 | e30657-e30657 | Not relevant |
| Ambreen 2021 | Ambreen, H.; Tariq, H.; Amjad, I. | Effects of bilateral arm training on upper extremity function in right and left hemispheric stroke | JPMA - Journal of the Pakistan Medical Association | 2021 | 71 | 1(B) | 302-305 | Not relevant |
| Ambrosetti 2019 | Ambrosetti, Marco; La Rovere, Maria Teresa; Scalvini, Simonetta; Pedretti, Roberto F. E. | Cardiac rehabilitation in heart failure after the ExTraMATCH II study: who still believes?...Taylor RS, Walker S, Smart NA, et al. Impact of exercise-based cardiac rehabilitation in patients with heart failure (ExTraMATCH II) on mortality and hospitalisation: an individual patient data meta-analysis of randomised trials. European Journal of Heart Failure. 2018; 20(12):1735-1743 |  | 2019 | 21 |  | 257-257 | Not relevant |
| Ambrosini 2021 | Ambrosini, E.; Gasperini, G.; Zajc, J.; Immick, N.; Augsten, A.; Rossini, M.; Ballarati, R.; Russold, M.; Ferrante, S.; Ferrigno, G.; Bulgheroni, M.; Baccinelli, W.; Schauer, T.; Wiesener, C.; Gfoehler, M.; Puchinger, M.; Weber, M.; Weber, S.; Pedrocchi, A.; Molteni, F.; Krakow, K. | A Robotic System with EMG-Triggered Functional Eletrical Stimulation for Restoring Arm Functions in Stroke Survivors | NeuroRehabilitation & Neural Repair | 2021 | 35 | 4 | 334-345 | Not relevant |
| Ambrosini 2020 | Ambrosini, E.; Parati, M.; Peri, E.; De Marchis, C.; Nava, C.; Pedrocchi, A.; et al., | Changes in leg cycling muscle synergies after training augmented by functional electrical stimulation in subacute stroke survivors: a pilot study | Journal of NeuroEngineering and Rehabilitation | 2020 | 17 | 1 | 35 | Not relevant |
| Ambrosini 2020 | Ambrosini, E.; Peri, E.; Nava, C.; Longoni, L.; Monticone, M.; Pedrocchi, A.; Ferriero, G.; Ferrante, S. | A multimodal training with visual biofeedback in subacute stroke survivors: a randomized controlled trial | European Journal of Physical & Rehabilitation Medicine. | 2020 | 56 | 1 | 24-33 | Wrong comparator - active control |
| Ambrosini 2019 | Ambrosini, E.; Zajc, J.; Ferrante, S.; Ferrigno, G.; Gasperina, S. D.; Bulgheroni, M.; Baccinelli, W.; Schauer, T.; Wiesener, C.; Russold, M.; Gfoehler, M.; Puchinger, M.; Weber, M.; Becker, S.; Krakow, K.; Immick, N.; Augsten, A.; Rossini, M.; Proserpio, D.; Gasperini, G.; Molteni, F.; Pedrocchi, A. | A Hybrid Robotic System for Arm Training of Stroke Survivors: Concept and First Evaluation | IEEE Transactions on BioMedical Engineering | 2019 | 66 | 12 | 3290-3300 | Not relevant |
| Amiri 2022 | Amiri, F. S.; Abolhassani, S.; Alimohammadi, N.; Roghani, T. | Investigating the effect of self-management program on stroke's patients' self-efficacy | BMC Neurology | 2022 | 22 | 1 | 360 | Not relevant |
| Amiripour 2023 | Amiripour, M. S.; Amiripour, A.; Nasiri, S.; Omidi, S.; Raisi, F. | Comparison of Combined Aerobic-Resistance and Aerobic Training on Heart Systolic Function in Heart Failure Patients after Coronary Artery Bypass Graft Surgery (CABG) | Journal of Kermanshah University of Medical Sciences | 2023 | 27 | 1 |  | Wrong comparator - exercise v exercise |
| Amorim 2019 | Amorim, H.; Cadilha, R.; Parada, F.; Rocha, A. | Progression of aerobic exercise intensity in a cardiac rehabilitation program | Revista Portuguesa De Cardiologia | 2019 | 38 | 4 | 281-286 | Wrong study design - not RCT |
| Amreen 2021 | Amreen, M.; Manikandan, N.; Coralie, E.; John, S. | Development and testing of adherence-enhancing strategies for home-based exercise program among community-living stroke survivors | Cerebrovascular Diseases (Basel, Switzerland) | 2021 | 50 | 1 | 83 | Not relevant |
| AmsterdamUmc 2017 | Amsterdam Umc, location VUmc; Utrecht, U. M. C.; Hospital, Maasstad; Alkmaar, Northwest Clinics | Rate Adaptive Atrial Pacing in Heart Failure |  | 2017 |  |  |  | Not relevant |
| An 2020 | An, B.; Woo, Y.; Park, K.; Kim, S. | Effects of insole on the less affected side during execution of treadmill walking training on gait ability in chronic stroke patients: a preliminary study | Restorative Neurology and Neuroscience | 2020 | 38 | 5 | 375-384 | Not relevant |
| An 2016 | An, C. M.; Won, J. I. | Effects of ankle joint mobilization with movement and weight-bearing exercise on knee strength, ankle range of motion, and gait velocity in patients with stroke: a pilot study | Journal of Physical Therapy Science | 2016 | 28 | 2 | 689-694 | Not relevant |
| Anandan 2020 | Anandan, D.; Tamil Nidhi, P. K.; Arun, B.; Priya, V. | Effect of task specific training with proprioceptive neuromuscular facilitation on stroke survivors | BioMedicine (India) | 2020 | 40 | 3 | 363-366 | Not relevant |
| AnastasiaInozemtseva 2019 | Anastasia Inozemtseva, A. A.; Argunova, Y. A.; Pomeshkina, S. A.; Barbarash, O. L. | Exercise training with selected loads in the rehabilitation of patients after coronary artery bypass grafting | European Journal of Preventive Cardiology | 2019 | 26 |  | S159-S160 | Wrong intervention < 6 weeks |
| AndersonDonelliSilveira 2018 | Anderson Donelli Silveira, A. D.; Lima, J. B.; Piardi, D. S.; Horn, T. L.; Macedo, D. S.; Zanini, M.; Nery, R. M.; Stein, R. | High intensity interval training is effective and superior to moderate continuous training in patients with heart failure with preserved ejection fraction: a randomized Clinical trial | European Journal of Preventive Cardiology | 2018 | 25 | 2 | S91 | Wrong comparator - exercise v exercise |
| Ando 2020 | Ando, D.; Yokota, C.; Koshino, K.; Yasuno, F.; Sato, T.; Yamamoto, A.; Odani, H.; Nakajima, T.; Higuchi, T.; Tatsumi, E. | Microstructural white matter changes following gait training with Hybrid Assistive Limb initiated within 1 week of stroke onset | Journal of the Neurological Sciences | 2020 | 415 |  | 116939 | Not relevant |
| Andrade 2022 | Andrade, C. C. F.; Silva, R. T.; Brunherotti, M. A. D. A. | Effects of Inspiratory Muscle Training in Patients With Class III and IV Heart Failure | Current Problems in Cardiology | 2022 | 47 (no pagination) | 10 |  | Not relevant |
| Andrade 2019 | Andrade, G.; Meireles, A. F. X.; Lopes, E. B.; Lima, F. O.; Maia, F. | A protocol for acute neurofunctional Rehabilitative therapy following ischemic stroke | Neurology. Conference: 71st Annual Meeting of the American Academy of Neurology, AAN | 2019 | 92 | 15(1) |  | Not relevant |
| Andrade 2021 | Andrade, G. N.; Umeda, I. I. K.; Fuchs, Arcn; Mastrocola, L. E.; Rossi-Neto, J. M.; Moreira, D. A. R.; Oliveira, P. A.; Andre, C. D. S.; Cahalin, L. P.; Nakagawa, N. K. | Home-based training program in patients with chronic heart failure and reduced ejection fraction: a randomized pilot study | Clinics (Sao Paulo, Brazil) | 2021 | 76 |  | e2550 | Wrong comparator - exercise v exercise |
| Andrade-Lima 2021 | Andrade-Lima, A.; Silva Junior, N.; Chehuen, M.; Miyasato, R.; Souza, R. W. A.; Leicht, A. S.; Brum, P. C.; de Oliveira, E. M.; Wolosker, N.; Forjaz, C. L. M. | Walking Training Improves Systemic and Local Pathophysiological Processes in Intermittent Claudication | European Journal of Vascular and Endovascular Surgery | 2021 | 61 | 6 | 954-963 | Wrong comparator - active control |
| Andrushko 2023 | Andrushko, J. W.; Rinat, S.; Larssen, B.; Greeley, B.; Jones, C. B.; Rubino, C.; Denyer, R.; Ferris, J.; Boyd, L. | Improved cognitive-motor processing speed is related to a decrease in functional connectivity between the dorsolateral prefrontal cortex and the sensorimotor network after aerobic exercise in chronic stroke | NeuroRehabilitation and Neural Repair | 2023 | 37 | 5 | 30S | Wrong intervention < 6 weeks |
| Anglia 2019 | Anglia, University of East | Dose-Finding of Lower Limb Mirror Therapy After Stroke |  | 2019 |  |  |  | Not relevant |
| Anglia 2021 | Anglia, University of East; Norwich Clinical Trials Unit, U. K.; The Stroke Association, United Kingdom | Can we Use c-SIGHT for Spatial Neglect in Stroke Survivors' Homes? |  | 2021 |  |  |  | Wrong intervention - does not meet exercise definition |
| Anne 2018 | Anne, Centre Hospitalier St | Evaluation of the Benefit of the Training of the Manual Dexterity Post Stroke |  | 2018 |  |  |  | Not relevant |
| Anonymous 2016 | Anonymous, | 2016 Chinese Diabetes Society Meeting | The Lancet Diabetes and Endocrinology. Conference | 2016 | 4 | SPEC. ISSUE 3 |  | Not relevant |
| Anonymous 2016 | Anonymous, | CAMSTRAND Conference 2016 Abstracts | European Journal of Integrative Medicine. Conference: CAMSTRAND Conference | 2016 | 8 | 4 |  | Not relevant |
| Anonymous 2016 | Anonymous, | ESC Congress 2016 | European Heart Journal. Conference: European Society of Cardiology, ESC Congress | 2016 | 37 | 1 |  | Not relevant |
| Anonymous 2016 | Anonymous, | Abstracts of the Heart Failure 2016 and the 3rd World Congress on Acute Heart Failure | European Journal of Heart Failure. Conference: Heart Failure | 2016 | 18 | 1 |  | Not relevant |
| Anonymous 2017 | Anonymous, | Corrigendum to: UK Stroke Forum abstracts (Int J Stroke, (2016), 11, (S6-S63), 10.1177/1747493016669275) | International Journal of Stroke | 2017 | 12 | 3 | NP8-NP9 | Not relevant |
| Anonymous 2017 | Anonymous, | Cirse 2017 | CardioVascular and Interventional Radiology. Conference: Cardiovascular and Interventional Radiological Society of Europe, CIRSE | 2017 | 40 | 2(1) |  | Not relevant |
| Anonymous 2018 | Anonymous, | Exercise is an important intervention for treating intermittent claudication | Drug and Therapeutics Bulletin | 2018 | 56 | 11 | 130 | Wrong study design - not RCT |
| Anonymous 2018 | Anonymous, | Canadian Association of Cardiovascular Prevention and Rehabilitation 2018 Fall Conference Abstracts | Journal of Cardiopulmonary Rehabilitation and Prevention. Conference | 2018 | 38 | 6 |  | Not relevant |
| Anonymous 2018 | Anonymous, | Global Health Challenges to Community-Based and Individual Psychosocial Intervention Strategies - Gemeinsamer Kongress der DGMP und der DGMS | PPmP Psychotherapie Psychosomatik Medizinische Psychologie. Conference: Global Health Challenges to Community Based and Individual Psychosocial Intervention Strategies Gemeinsamer Kongress der DGMP und der DGMS. Leipzig Germany | 2018 | 68 | 8 |  | Not relevant |
| Anonymous 2019 | Anonymous, | Esnr 2019 | Neuroradiology. Conference: 42nd Annual Meeting of the European Society of Neuroradiology Diagnostic and Interventional, ESNR | 2019 | 61 | 1 |  | Not relevant |
| Anonymous 2019 | Anonymous, | Abstracts From the American Heart Association's Hypertension 2019 Scientific Sessions | Hypertension. Conference: American Heart Association's Hypertension | 2019 | 74 | 1 |  | Not relevant |
| Anonymous 2019 | Anonymous, | Abstracts to be presented at the Society for Vascular Medicine 2019 Annual Scientific Sessions | Vascular Medicine. Conference: 30th Annual Scientific Sessions of the Society for Vascular Medicine, SVM | 2019 | 24 | 3 |  | Not relevant |
| Anonymous 2020 | Anonymous, | 28th Annual Scientific Congress Hong Kong College of Cardiology | Journal of the Hong Kong College of Cardiology. Conference: 28th Annual Scientific Congress of the Hong Kong College of Cardiology. Virtual. | 2020 | 28 | 1 |  | Not relevant |
| Anonymous 2020 | Anonymous, | CardioEgypt 2020 - Conference | European Heart Journal, Supplement. Conference: 47th Annual International Congress of the Egyptian Society of Cardiology, CardioEgypt | 2020 | 22 | SUPPL K |  | Not relevant |
| Anonymous 2020 | Anonymous, | CAEP/ACMU 2020 Scientific Abstracts | Canadian Journal of Emergency Medicine. Conference | 2020 | 22 | 1 |  | Not relevant |
| Anonymous 2020 | Anonymous, | Erratum: Effects of home-based cardiac exercise rehabilitation with remote electrocardiogram monitoring in patients with chronic heart failure: A study protocol for a randomised controlled trial (BMJ Open (2019) 9 (e023923) DOI: 10.1136/bmjopen-2018-023923) | BMJ Open | 2020 | 10(no pagination) | 7 |  | Not relevant |
| Anonymous 2021 | Anonymous, | Svm 2021 | Vascular Medicine. Conference: Annual Scientific Sessions of the Society for Vascular Medicine, SVM | 2021 | 26 | 5 |  | Not relevant |
| Anonymous 2021 | Anonymous, | Cacpr 2021 | Journal of Cardiopulmonary Rehabilitation and Prevention. Conference: Canadian Association of Cardiovascular Prevention and Rehabilitation Annual Meeting, CACPR | 2021 | 41 | 2 |  | Not relevant |
| Anonymous 2021 | Anonymous, | Correction: physical activity after Cardiac EventS (PACES): a group education programme with subsequent text message support designed to increase physical activity in individuals with diagnosed coronary heart disease: a randomised controlled trial | Open Heart | 2021 | 8 | 2 | 1 | Not relevant |
| Anonymous 2022 | Anonymous, | CardioAlex 2022 | European Heart Journal, Supplement | 2022 | 24 | G |  | Not relevant |
| Anonymous 2023 | Anonymous, | European Congress of NeuroRehabilitation, ECNR 2023 | Neurologie und Rehabilitation. Conference: European Congress of NeuroRehabilitation, ECNR | 2023 | 29 | 1 |  | Not relevant |
| Anonymous 2023 | Anonymous, | BACPR Annual Conference Abstracts | Heart | 2023 | 109 | 5 |  | Not relevant |
| Anonymous 2023 | Anonymous, | British Cardiovascular Society Annual Conference, 'Future-proofing Cardiology for the next 10 years' | Heart | 2023 | 109 | 3 |  | Not relevant |
| Antonio 2023 | Antonio, B. A.; Bonuzzi, G. M. G.; Alves, C. M. P.; Polese, J. C.; Mochizuki, L.; Torriani-Pasin, C. | Does dual task merged in a mixed physical exercise protocol impact the mobility under dual task conditions in mild impaired stroke survivors? A feasibility, safety, randomized, and controlled pilot trial | Disability and Rehabilitation | 2023 | 45 | 5 | 814-821 | Not relevant |
| Antoniou 2022 | Antoniou, V.; Xanthopoulos, A.; Giamouzis, G.; Davos, C.; Batalik, L.; Stavrou, V.; Gourgoulianis, K. I.; Kapreli, E.; Skoularigis, J.; Pepera, G. | Efficacy, efficiency and safety of a cardiac telerehabilitation programme using wearable sensors in patients with coronary heart disease: the TELEWEAR-CR study protocol | BMJ Open | 2022 | 12 | 6 | e059945 | Not relevant |
| Antunes-Correa 2017 | Antunes-Correa, L. M.; Ueno-Pardi, L. M.; Trevizan, P. F.; Santos, M. R.; da Silva, C. H.; Franco, F. G.; Alves, M. J.; Rondon, M. U.; Negrao, C. E. | The influence of aetiology on the benefits of exercise training in patients with heart failure | European Journal of Preventive Cardiology | 2017 | 24 | 4 | 365-372 | Wrong study design - not RCT |
| Antwerpen 2018 | Antwerpen, Universiteit; University Hospital, Antwerp | What do Stroke Survivors Actually Learn When Regaining Walking Ability After Stroke? The TARGET Phase I Study |  | 2018 |  |  |  | Not relevant |
| Anwar 2022 | Anwar, N.; Karimi, H.; Ahmad, A.; Gilani, S. A.; Khalid, K.; Aslam, A. S.; Hanif, A. | Virtual Reality Training Using Nintendo Wii Games for Patients With Stroke: Randomized Controlled Trial | JMIR sSerious Games | 2022 | 10 | 2 | e29830 | Wrong intervention - does not meet exercise definition |
| Anwar 2021 | Anwar, N.; Karimi, H.; Ahmad, A.; Mumtaz, N.; Saqulain, G.; Gilani, S. A. | A Novel Virtual Reality Training Strategy for Poststroke Patients: A Randomized Clinical Trial | Journal of Healthcare Engineering | 2021 | 2021 |  | 6598726 | Wrong intervention - does not meet exercise definition |
| Anwar 2022 | Anwar, S.; Fayyaz, M. U.; Saleem, S.; Imran, A.; Noman, H.; Shah, S. S. A. | Effectiveness of Motor Imagery Training to Improve Gait Abilities of Patients with Sub-Acute Stroke | Pakistan Journal of Medical and Health Sciences | 2022 | 16 | 3 | 504-505 | Not relevant |
| Ao-xue 2023 | Ao-xue, L. I.; Yun-wen, L. I.; Cui-hong, Zhang | Effect of progressive resistance training on hemorheology and exercise tolerance in patients with upper limb spastic hemiplegia after cerebral infarction | Chinese Journal of Convalescent Medicine / Zhongguo Liaoyang Yixue | 2023 | 32 | 7 | 747-750 | Full text not in English |
| Ap 2022 | Ap, D. E. Lima; Pereira, D. G.; Nascimento, I. O.; Martins, T. H.; Oliveira, A. C.; Nogueira, T. S.; Britto, R. R. | Cardiac telerehabilitation in a middle-income country: analysis of adherence, effectiveness and cost through a randomized Clinical trial | European Journal of Physical and Rehabilitation Medicine | 2022 | 58 | 4 | 598-605 | Wrong comparator - exercise v exercise |
| Aprile 2020 | Aprile, I.; Germanotta, M.; Cruciani, A.; Loreti, S.; Pecchioli, C.; Cecchi, F.; Montesano, A.; et al. | Upper Limb Robotic Rehabilitation After Stroke: A Multicenter, Randomized Clinical Trial | Journal of Neurologic Physical Therapy | 2020 | 44 | 1 | 3-14 | Not relevant |
| Aprile 2017 | Aprile, I.; Iacovelli, C.; Padua, L.; Galafate, D.; Criscuolo, S.; Gabbani, D.; Cruciani, A.; Germanotta, M.; Di Sipio, E.; De Pisi, F.; Franceschini, M. | Efficacy of Robotic-Assisted Gait Training in chronic stroke patients: Preliminary results of an Italian bi-centre study | NeuroRehabilitation | 2017 | 41 | 4 | 775-782 | Not relevant |
| Arce-Esquivel 2018 | Arce-Esquivel, Arturo A.; Ballard, Joyce E.; Hermanns, Melinda L.; Rath, Linda R.; Murley, Brittany; Wang, Yong T.; Haas, Barbara K. | Long-term Effects of Tai Chi on Muscle Strength and physical Function in Patients with Peripheral Neuropathy: 1982 Board #243 May 31 3:30 PM - 5:00 PM...American College of Sports Medicine Annual Meeting, May 29-June 2, 2018, Minneapolis, Minnesota | Medicine & Science in Sports & Exercise | 2018 | 50 |  | 480-480 | Not relevant |
| Ardestani 2019 | Ardestani, M. M.; Henderson, C. E.; Hornby, T. G. | Improved walking function in laboratory does not guarantee increased community walking in stroke survivors: Potential role of gait biomechanics | Journal of Biomechanics | 2019 | 91 |  | 151-159 | Wrong comparator - exercise v exercise |
| Ardestani 2020 | Ardestani, M. M.; Henderson, C. E.; Mahtani, G.; Connolly, M.; Hornby, T. G. | Locomotor Kinematics and Kinetics Following High-Intensity Stepping Training in Variable Contexts Poststroke | NeuroRehabilitation and Neural Repair | 2020 | 34 | 7 | 652-660 | Wrong intervention - does not meet exercise definition |
| Arezomand 2024 | Arezomand, M.; Dehghan, M.; Rigi, Z. E.; Fatehi, F.; Shahrbabaki, P. M. | The effect of using a sports application on the quality of sleep in patients with heart failure: a randomized Clinical trial study | BMC sports Science, Medicine & Rehabilitation | 2024 | 16 | 1 | 15 | Wrong comparator - co-interventions not balanced between arms |
| AriasLabrador 2023 | Arias Labrador, E.; Vilaró Casamitjana, J.; Blanco Dí­az, S.; Brugué Pascual, E.; Buxó Pujolrí s, M.; Grau, J. I.; Ramos Blanes, R.; Brugada Terradellas, R. | Efectos de un programa de rehabilitación cardí­aca fase iii en la capacidad funcional y composición corporal en pacientes con cardiopatí­a isquémica | Rehabilitacion | 2023 | 57 | 3 |  | Full text not in English |
| Arjunan 2021 | Arjunan, P.; D'Souza, M. S. | Efficacy of nurse-led cardiac rehabilitation on health care behaviours in adults with chronic heart failure: an experimental design | Clinical Epidemiology and Global Health | 2021 | 12 |  |  | Not relevant |
| Arnao 2019 | Arnao, V.; Riolo, M.; Carduccio, F.; Tuttolomondo, A.; D'Amelio, M.; Brighina, F.; Gangitano, M.; Salemi, G.; Ragonese, P.; Aridon, P. | Effects of transcranial random noise stimulation combined with Graded Repetitive Arm Supplementary Program (GRASP) on motor rehabilitation of the upper limb in sub-acute ischemic stroke patients: a randomized pilot study | Journal of neural transmission (Vienna, Austria : 1996) | 2019 | 126 | 12 | 1701-1706 | Not relevant |
| Arnar 2022 | Arnar, D. O.; Oddsson, S.; Gunnarsdottir, T.; Olafsdottir, I. V.; Gudmundsdottir, M. V.; Loftsson, K. H.; Gudmundsson, E. F.; Kaernested, B.; Amundadottir, M. L.; Sigurdardottir, S.; Mogensen, B.; Libungan, B.; Thorgeirsson, T. | A Digital Therapeutic Intervention Is Feasible for Outpatient Care in Coronary Artery Disease | Circulation. Conference: American Heart Association's Epidemiology and Prevention/Lifestyle and Cardiometabolic Health | 2022 | 145 | 1 |  | Not relevant |
| Aronov 2017 | Aronov, D. M.; Bubnova, M. G.; Ioseliani, D. G.; Krasnitsky, V. B.; Shovkun, T. V.; Novikova, N. K.; Yarnykh, E. V. | The Complex Program of Rehabilitation of Patients With Ischemic Heart Disease After Coronary Artery Bypass Surgery in Ambulatory Cardiorehabilitational Department: Clinical Effects of Third Stage of Rehabilitation. [Russian] | Kardiologiia | 2017 | 57 | 3 | 10-19 | Exclude - not able to retrieve full text |
| Aronov 2006 | Aronov, D. M.; Bubnova, M. G.; Pogosova, G. V.; Novikova, N. K.; Krasnitskii, V. B.; Pozdniakov Iu, M.; Zhidko, N. I.; Akhmedzhanov, N. M. | Rehabilitation of patients with ischemic heart disease at outpatient stage. [Russian] | Kardiologiia | 2006 | 46 | 2 | 86-99 | Exclude - not able to retrieve full text |
| Arshad 2022 | Arshad, H.; Khattak, H. G.; Anwar, K.; Majeed, Y.; Malakandi, H. B. | COMPARISON OF EXERGAMES VERSUS TRADITIONAL BALANCE EXERCISE TO IMPROVE BALANCE AND REDUCE RISK OF FALLS IN CHRONIC STROKE PATIENTS | Journal of Medical Sciences (Peshawar) | 2022 | 30 | 2 | 134-138 | Wrong intervention - does not meet exercise definition |
| Aruin 2018 | Aruin, A. S.; Rao, N. | The effect of a single textured insole in gait rehabilitation of individuals with stroke | International Journal of Rehabilitation Research | 2018 | 41 | 3 | 218-223 | Not relevant |
| Arumugam 2020 | Arumugam, Kumaresan; Bose, Senthilkumar Cennappan; Sahu, Rama Kumar | Effect of Repetitive Task Training to Improve Sit to Stand Performance and Activities of Daily Living Skills in Patients with Stroke | Indian Journal of Physiotherapy & Occupational Therapy | 2020 | 14 | 4 | 57-62 | Wrong intervention - does not meet exercise definition |
| Arya 2019 | Arya, Kamal Narayan; Pandian, Shanta | Interlimb Coupling in Poststroke Rehabilitation: A Randomized Controlled Trial...56th All India Occupational Therapists-™ Association (AIOTA) Conference (OTICON-™2019), February 8-10, 2019, at PGIMER, Chandigarh, India | Indian Journal of Occupational Therapy (Wolters Kluwer India Pvt Ltd) | 2019 | 51 | 2 | 66-66 | Wrong intervention - does not meet exercise definition |
| Arya 2019 | Arya, K. N.; Pandian, S.; Kumar, V. | Effect of activity-based mirror therapy on lower limb motor-recovery and gait in stroke: A randomised controlled trial | Neuropsychological Rehabilitation | 2019 | 29 | 8 | 1193-1210 | Not relevant |
| Arya 2020 | Arya, K. N.; Pandian, S.; Sharma, A.; Kumar, V.; Kashyap, V. K. | Interlimb coupling in poststroke rehabilitation: a pilot randomized controlled trial | Topics in Stroke Rehabilitation | 2020 | 27 | 4 | 272-289 | Wrong intervention - does not meet exercise definition |
| Asghar 2021 | Asghar, M.; Fatima, A.; Warner, S.; Khan, M. H. U.; Ahmad, A.; Siddique, K. | Effectiveness of proprioceptive neuromuscular facilitation on balance in chronic stroke patients | Rawal Medical Journal | 2021 | 46 | 1 | 212-215 | Not relevant |
| Ashizawa 2023 | Ashizawa, Ryota; Honda, Hiroya; Take, Koki; Yoshizawa, Kohei; Kameyama, Yuto; Yoshimoto, Yoshinobu | Effects on sedentary behaviour of an approach to reduce sedentary behaviour in patients with minor ischaemic stroke: A randomised controlled trial | Clinical Rehabilitation | 2023 | 37 | 4 | 545-556 | Not relevant |
| Ashizawa 2022 | Ashizawa, R.; Honda, H.; Take, K.; Yoshizawa, K.; Ooba, Y.; Kameyama, Y.; Yoshimoto, Y. | Approaches to Promote Reduction in Sedentary Behavior in Patients With Minor Ischemic Stroke: A Randomized Controlled Trial | Archives of Physical Medicine & Rehabilitation | 2022 | 103 | 2 | 255-262.e4 | Not relevant |
| Ashok 2023 | Ashok, A.; Ku, D. K.; Mundayat, G. | Management of Anxiety in Coronary Artery Bypass Grafting Patients: the Influence of Chair Aerobics and Nadisodhana Pranayama-A Pilot Randomized Clinical Trial | Indian Journal of Pharmaceutical education and Research | 2023 | 57 | 2 | s405-s410 | Wrong intervention < 6 weeks |
| Ashok 2021 | Ashok, A.; Kumar, K. U. D.; Gopalakrishnan, M. | Outcome of Chair Aerobics & Pranayama on Anxiety and Exercise Tolerance in Coronary Artery Bypass Grafting Patients: study Protocol of a Randomized Clinical Trial | International Journal of Surgery protocols | 2021 | 25 | 1 | 238-243 | Wrong intervention < 6 weeks |
| Askim 2023 | Askim, T.; Hokstad, A.; Bergh, E.; Dohl, O.; Ellekjaer, H.; Ihle-Hansen, H.; Indredavik, B.; Leer, A. S. M.; Lydersen, S.; Saltvedt, I.; Seljeseth, Y.; Thommessen, B. | Multimodal individualised intervention to prevent functional decline after stroke: protocol of a randomised controlled trial on long-term follow-up after stroke (LAST-long) | BMJ Open | 2023 | 13 | 5 | e069656 | Wrong intervention - does not meet exercise definition |
| Askim 2018 | Askim, T.; Langhammer, B.; Ihle-Hansen, H.; Gunnes, M.; Lydersen, S.; Indredavik, B.; Group, Last Collaboration | Efficacy and Safety of Individualized Coaching After Stroke: the LAST Study (Life After Stroke): a Pragmatic Randomized Controlled Trial | Stroke | 2018 | 49 | 2 | 426-432 | Wrong comparator - exercise v exercise |
| Askim 2018 | Døhl, Ø.; Halsteinli, V.; Askim, T.; Gunnes, M.; Ihle-Hansen, H.; Indredavik, B.; Langhammer, B.; Phan, A.; Magnussen, J. | Factors contributing to post-stroke health care utilization and costs, secondary results from the life after stroke (LAST) study | BMC Health Services Research | 2020 | 20 | 1 | 1-Oct | Wrong comparator - exercise v exercise |
| Askim 2018 | Langhammer, B.; Gunnes, M.; Ihle-Hansen, H.; Indredavik, B.; Askim, T. | A physical activity program is no more effective than no intervention at maintaining upper limb activity in community-dwelling people with stroke, the last trial | European Stroke Journal | 2019 | 4 |  | 57-58 | Wrong comparator - exercise v exercise |
| Askim 2018 | Askim, T.; Langhammer, B.; Ihle-Hansen, H.; Lydersen, S.; Gunnes, M.; Indredavik, B. | Effect of an individualized prolonged follow up programme for maintanance of motor function after stroke-the last study. A multisite randomised controlled trial | International Journal of Stroke | 2016 | 11 |  | 21 | Wrong comparator - exercise v exercise |
| Askim 2018 | Gunnes, M.; Indredavik, B.; Langhammer, B.; Lydersen, S.; Ihle-Hansen, H.; Dahl, A. E.; Askim, T. | Associations Between Adherence to the physical activity and Exercise Program Applied in the LAST Study and Functional Recovery After Stroke | Archives of Physical Medicine and Rehabilitation | 2019 | 100 | 12 | 2251-2259 | Wrong comparator - exercise v exercise |
| Askim 2018 | Askim, T.; Langhammer, B.; Hege, I. H.; Mari, G.; Stian, L.; Bent, I. | A randomized controlled trial assessing the effect of a long-term follow-up programme aiming to maintain motor function after stroke: the life after stroke (last) study | European Stroke Journal | 2016 | 1 | 1 | 727 | Wrong comparator - exercise v exercise |
| Askim 2018 | Gunnes, M.; Langhammer, B.; Aamot, I. L.; Lydersen, S.; Schroeter, W.; Reneflot, K.; Askim, T. | How well do stroke survivors adhere to an 18-month physical activity and exercise programme? secondary results from a randomised controlled trial | Gait & posture | 2017 | 57 |  | 166 | Wrong comparator - exercise v exercise |
| Askim 2018 | Gunnes, M.; Langhammer, B.; Aamot, I. L.; Lydersen, S.; Ihle-Hansen, H.; Indredavik, B.; Reneflot, K. H.; Schroeter, W.; Askim, T. | Adherence to a Long-Term physical activity and Exercise Program After Stroke Applied in a Randomized Controlled Trial | Physical Therapy | 2019 | 99 | 1 | 74-85 | Wrong comparator - exercise v exercise |
| Askim 2018 | Langhammer, B.; Ada, L.; Gunnes, M.; Ihle-Hansen, H.; Indredavik, B.; Askim, T. | A physical activity program is no more effective than standard care at maintaining upper limb activity in community-dwelling people with stroke: secondary outcomes from a randomized trial | Clinical Rehabilitation | 2019 | 33 | 10 | 1607-1613 | Wrong comparator - exercise v exercise |
| Aslam 2021 | Aslam, M.; Ain, Q. U.; Fayyaz, P.; Malik, A. N. | Exer-gaming reduces fall risk and improves mobility after stroke | JPMA - Journal of the Pakistan Medical Association | 2021 | 71 | 6 | 1673-1675 | Wrong intervention - does not meet exercise definition |
| Assareh 2022 | Assareh, A. R.; Jafarpor, M.; Haghighzadeh, M. H.; Akiash, N. | Effect of cardiac rehabilitation on endothelial function in smoker patients with ischemic heart disease. [Persian] | Tehran University Medical Journal | 2022 | 80 | 6 | 454-461 | Full text not in English |
| Atef 2020 | Atef, H.; Helmy, Z.; Abdelhameed, A.; Elameen, S. | Effect of different types of exercise on sleep deprivation and functional capacity in middle aged patients after coronary artery bypass grafting | European Journal of Cardiovascular Nursing | 2020 | 19 | SUPPL 1 | S50 | Wrong comparator - exercise v exercise |
| Atef 2020 | Atef, H.; Helmy, Z.; Farghaly, A. | Effect of different types of exercise on sleep deprivation and functional capacity in middle aged patients after coronary artery bypass grafting | Sleep Science | 2020 | 13 | 2 | 113-118 | Wrong comparator - exercise v exercise |
| AttarzadehHosseini 2020 | Attarzadeh Hosseini, S. R.; Moazzami, M.; Farahati, S.; Bahremand, M.; Sadegh Eghbali, F. | Effects of high-intensity interval training versus moderate-intensity continuous training on the total antioxidant capacity, malondialdehyde, and superoxide dismutase in obese/overweight middle-aged women. [Persian] | Iranian Journal of Endocrinology and Metabolism | 2020 | 22 | 3 | 207-213 | Full text not in English |
| Auclair 2011 | Auclair, A.; Poirier, P.; Valera, B.; Vadeboncoeur, N.; Desgagnés, P.; Houle, J. | Pedometer'S Target Of 7500 Steps/day Improves Health Following An Acute Coronary Syndrome | Medicine & Science in Sports & Exercise | 2011 | 43 |  | 282-283 | Not relevant |
| Aung 2022 | Aung, N.; Hiengkaew, V.; Tretriluxana, J.; Bryant, M. S.; Bovonsunthonchai, S. | Effectiveness of Motor Imagery Combined with Structured Progressive Circuit Class Training on Functional Mobility in Post-Stroke Individuals: A Randomized Controlled Trial | Journal of Rehabilitation Medicine | 2022 | 54 |  |  | Not relevant |
| Austin 2012 | Austin, J.; Williams, W. R.; Hutchison, S. | Patterns of fatigue in elderly heart failure patients measured by a quality of life scale (Minnesota living with heart failure) | European Journal of Cardiovascular Nursing | 2012 | 11 | 4 | 439-444 | Wrong comparator - co-interventions not balanced between arms |
| Austin 2022 | Austin, University of Texas at; University of Maryland, Baltimore; University, Texas State | Treadmill Oscillation Walking to Improve Weight Transfer During Gait Following Stroke |  | 2022 |  |  |  | Not relevant |
| Authority 2016 | Authority, Nova Scotia Health | Stepping up Aerobic Exercise to Improve Health Outcomes After Stroke |  | 2016 |  |  |  | Not relevant |
| Avazpour 2023 | Avazpour, S.; Amini, A.; Shirvani, H.; Arabzadeh, E. | Exercise modulation in inflammation and metabolic hormonal disorders of COVID-19 to decrease risk factors in coronary heart disease | Hormone Molecular Biology & Clinical Investigation | 2023 | 44 | 2 | 199-206 | Wrong study design - not RCT |
| Awad 2016 | Awad, L. N.; Reisman, D. S.; Pohlig, R. T.; Binder-Macleod, S. A. | Reducing The Cost of Transport and Increasing Walking Distance After Stroke: a Randomized Controlled Trial on Fast Locomotor Training Combined With Functional Electrical Stimulation | NeuroRehabilitation and Neural Repair | 2016 | 30 | 7 | 661-670 | Not relevant |
| Aydogdu 2018 | Aydogdu, Y. T.; Aydogdu, O.; Serap Inal, H. | The Effects of Dual-Task Training on Patient Outcomes of Institutionalized Elderly Having Chronic Stroke | Dementia and Geriatric cognitive disorders extra | 2018 | 8 | 3 | 328-332 | Wrong study design - not RCT |
| Azim 2021 | Azim, Derya; University, BandÄ±rma Onyedi Eylül | Investigation Of Two Different İnterventions İn Hemiplegic Patients |  | 2021 |  |  |  | Not relevant |
| R 2020 | R. B. R. | Effects of physical exercise in the fadigue of the patient with heart and lung disease |  | 2020 |  |  |  | Wrong study design - not RCT |
| Babaeipour 2018 | Babaeipour, H. | The effects of aquatic exercises in shallow and deep water on balance, proprioception, muscle strength and quality of life in chronic ischemic stroke male patients |  | 2018 |  |  |  | Full text not in English |
| Babaeipour 2018 | Babaeipour, H.; Al Zamani, M. S.; Mohammadipour, F.; Vakilian, A. | The effect of 6-week water exercise on quality of life in patients with chronic ischemic stroke: a randomized Clinical trial | Journal of rafsanjan university of Medical Sciences | 2018 | 17 | 8 | 699-714 | Full text not in English |
| Babber 2020 | Babber, A.; Ravikumar, R.; Onida, S.; Lane, T. R. A.; Davies, A. H. | Effect of footplate neuromuscular electrical stimulation on functional and quality-of-life parameters in patients with peripheral artery disease: pilot, and subsequent randomized Clinical trial | British Journal of Surgery | 2020 | 107 | 4 | 355-363 | Not relevant |
| Babu 2016 | Babu, A. S.; Desai, C. V.; Maiya, A. G.; Guddattu, V.; Padmakumar, R. | Changes in derived measures from six-minute walk distance following home-based exercise training in congestive heart failure: A preliminary report | Indian Heart Journal | 2016 | 68 | 4 | 527-8 | Not relevant |
| Bachmann 2015 | Bachmann, C. | Effect of endurance training and hawthorn extract WS 1442 in patients with heart failure with preserved ejection fraction - A randomized controlled trial | Schweizerische Zeitschrift fur GanzheitsMedizin | 2015 | 27 | 5 | 264-267 | Full text not in English |
| Back 2023 | Bäck, M.; Leosdottir, M.; Ekstrom, M.; Hambraeus, K.; Ravn-Fischer, A.; Oberg, B.; Ostlund, O.; James, S. | The remote exercise SWEDEHEART study-Rationale and design of a multicenter registry-based cluster randomized crossover Clinical trial (RRCT) | American Heart Journal | 2023 | 262 |  | 110-118 | Wrong comparator - exercise v exercise |
| Baer 2018 | Baer, G. D.; Salisbury, L. G.; Smith, M. T.; Pitman, J.; Dennis, M. | Treadmill training to improve mobility for people with sub-acute stroke: a phase II feasibility randomized controlled trial | Clinical Rehabilitation | 2018 | 32 | 2 | 201-212 | Wrong intervention - does not meet exercise definition |
| Baer 2018 | Baer, G. D.; Salisbury, L. G.; Smith, M. T.; Pitman, J.; Dennis, M. | Treadmill training to improve mobility for people with sub-acute stroke: a phase ii feasibility randomised controlled trial |  | 2017 |  |  |  | Wrong intervention - does not meet exercise definition |
| Baert 2018 | Baert, A.; Clays, E.; Bolliger, L.; De Smedt, D.; Lustrek, M.; Vodopija, A.; Bohanec, M.; Puddu, P. E.; Ciancarelli, M. C.; Schiariti, M.; Derboven, J.; Tartarisco, G.; Pardaens, S.; HeartMan, consortium | A Personal Decision Support System for Heart Failure Management (HeartMan): study protocol of the HeartMan randomized controlled trial | BMC Cardiovascular disorders | 2018 | 18 | 1 | 186 | Wrong comparator - co-interventions not balanced between arms |
| Baghaei 2021 | Baghaei, R.; Parizad, N.; Sharifi, A.; Alinejad, V. | The effect of continuous nursing care program on anxiety level, episodes of chest pain, and readmission rate after myocardial infarction: a randomized controlled trial | International Cardiovascular Research Journal | 2021 | 15 | 1 | 21-28 | Wrong comparator - co-interventions not balanced between arms |
| Bahrami 2019 | Bahrami, H.; Homaei, H.; Maleki, M.; Naderi, N. | Study design: the effects of photobiomodulation therapy combined with exercise training on functional capacity and quality of life in patients with heart failure (double-blind randomized) | Research in Cardiovascular Medicine | 2019 | 8 | 4 | 114-117 | Not relevant |
| Bailly 2018 | Bailly, L.; Mosse, P.; Diagana, S.; Fournier, M.; d'Arripe-Longueville, F.; Diagana, O.; Gal, J.; Grebet, J.; Moncada, M.; Domerego, J. J.; Radel, R.; Fabre, R.; Fuch, A.; Pradier, C. | "As du Coeur" study: a randomized controlled trial on quality of life impact and cost effectiveness of a physical activity program in patients with cardiovascular disease | BMC Cardiovascular Disorders | 2018 | 18 | 1 | 225 | Wrong comparator - exercise v exercise |
| Bakker 2018 | Bakker, E. A.; Snoek, J. A.; Meindersma, E. P.; Hopman, M. T. E.; Bellersen, L.; Verbeek, A. L. M.; Thijssen, D. H. J.; Eijsvogels, T. M. H. | Absence of Fitness Improvement Is Associated with Outcomes in Heart Failure Patients | Medicine and Science in sports and exercise | 2018 | 50 | 2 | 196-203 | Not relevant |
| Balling 2018 | Balling, L.; Thomsen, J. H.; Wolsk, E.; Hassager, C.; Boesgaard, S.; Goldsmith, S. R.; Gustafsson, F. | Hemodynamic effects of short-term infusion of a vasopressin V1A/V2 receptor antagonist conivaptan in patients withchronic heart failure during submaximal exercise | American Heart Journal | 2018 | 203 |  | 101-104 | Not relevant |
| Bandai 2022 | Bandai, Y.; Ariie, T. | Letter to the Editor: effectiveness of a Functional Rehabilitation Program for Upper Limb Apraxia in Poststroke Patients: a Randomized Controlled Trial | Archives of Physical Medicine and Rehabilitation | 2022 | 103 | 5 | 1046 | Not relevant |
| Bang 2016 | Bang, D. H.; Son, Y. L. | Effect of intensive aerobic exercise on respiratory capacity and walking ability with chronic stroke patients: a randomized controlled pilot trial | Journal of Physical Therapy Science | 2016 | 28 | 8 | 2381-2384 | Wrong intervention < 6 weeks |
| Banovic 2016 | Banovic, M.; Nikolic, S. D.; Putnik, S. | A Randomized Trial in Patients With Asymptomatic Severe Aortic Stenosis: a Future Has Begun! | Journal of the American College of Cardiology | 2016 | 67 | 16 | 1970-1971 | Not relevant |
| Barakat 2016 | Barakat, H. M.; Shahin, Y.; Khan, J. A.; McCollum, P. T.; Chetter, I. C. | Preoperative Supervised Exercise Improves Outcomes After Elective Abdominal Aortic Aneurysm Repair: a Randomized Controlled Trial | Annals of Surgery | 2016 | 264 | 1 | 47-53 | Not relevant |
| Barker 2018 | Barker, K.; Holland, A. E.; Lee, A. L.; Haines, T.; Ritchie, K.; Boote, C.; Saliba, J.; Lowe, S.; Pazsa, F.; Thomas, L.; Turczyniak, M.; Skinner, E. H. | Multimorbidity rehabilitation versus disease-specific rehabilitation in people with chronic diseases: a pilot randomized controlled trial | Pilot and Feasibility Studies | 2018 | 4 | 1 | 181 | Wrong population - under 80% vascular |
| Barts 2022 | Barts,; Trust, The London N. H. S. | A Mechanistic Exploratory Study of AF-induced Cardiac Dysfunction and Symptoms |  | 2022 |  |  |  | Wrong study design - not RCT |
| Barzideh 2020 | Barzideh, A.; Marzolini, S.; Danells, C.; Jagroop, D.; Huntley, A. H.; Inness, E. L.; Mathur, S.; Mochizuki, G.; Oh, P.; Mansfield, A. | Effect of reactive balance training on physical fitness poststroke: study protocol for a randomised non-inferiority trial | BMJ Open | 2020 | 10 | 6 | e035740 | Wrong comparator - active control |
| Bas-Sarmiento 2022 | Bas-Sarmiento, P.; Fernández-Gutiérrez, M.; Poza-Méndez, M.; Marí­n-Paz, A. J.; Paloma-Castro, O.; Romero-Sánchez, J. M.; Team, A. SyAG Ppic | Development and Effectiveness of a Mobile Health Intervention in Improving Health Literacy and Self-management of Patients With Multimorbidity and Heart Failure: Protocol for a Randomized Controlled Trial | JMIR Research protocols | 2022 | 11 | 4 | e35945 | Wrong comparator - co-interventions not balanced between arms |
| Bashir 2023 | Bashir, K.; Aravind, G.; Cameron, J. I.; Bayley, M. T.; Teasell, R.; Howe, J. A.; Tee, A.; Jaglal, S. B.; Hunter, S.; Salbach, N. M. | Experiences of recreation and healthcare managers and providers with first-time implementation of a community-based exercise program for people post-stroke: A theory-based qualitative study and cost analysis | NeuroRehabilitation and Neural Repair | 2023 | 37 | 5 | 50S-51S | Not relevant |
| Batalik 2018 | Batalik, L.; Dosbaba, F.; Hartman, M.; Batalikova, K.; Spinar, J. | Rationale and design of randomized controlled trial protocol of cardiovascular rehabilitation based on the use of teleMedicine technology in the Czech Republic (CR-GPS) | Medicine | 2018 | 97 | 37 | e12385 | Wrong comparator - exercise v exercise |
| Batalik 2021 | Batalik, L.; Dosbaba, F.; Hartman, M.; Konecny, V.; Batalikova, K.; Spinar, J. | Long-term exercise effects after cardiac telerehabilitation in patients with coronary artery disease: 1-year follow-up results of the randomized study | European Journal of Physical & Rehabilitation Medicine. | 2021 | 57 | 5 | 807-814 | Wrong comparator - exercise v exercise |
| Batalik 2021 | Batalik, L.; Pepera, G.; Papathanasiou, J.; Rutkowski, S.; Liska, D.; Batalikova, K.; Hartman, M.; Felsoci, M.; Dosbaba, F. | Is the training intensity in phase two cardiovascular rehabilitation different in telehealth versus outpatient rehabilitation? | Journal of Clinical Medicine | 2021 | 10 | 18 |  | Wrong comparator - exercise v exercise |
| Battesha 2022 | Battesha, H. H. M.; Wadee, A. N.; Shafeek, M. M.; Tawfick, A. M.; Ibrahim, H. M. | Maze Control Training on Kinesthetic Awareness in Patients with Stroke: a Randomized Controlled Trial | Rehabilitation Research and Practice | 2022 | 2022 |  |  | Wrong intervention - does not meet exercise definition |
| Baumgarten 2017 | Baumgarten, H.; Steinmetz, C.; Borst, C.; Walther, T.; Walther, C. | Preoperative exercise training before elective coronary artery bypass graft surgery: a prospective randomized evaluation on feasibility and effects on operative outcomes | Thoracic and Cardiovascular Surgeon | 2017 | 65 |  |  | Not relevant |
| Bayer 2018 | Bayer, | REALIsM-HF Pilot Study |  | 2018 |  |  |  | Not relevant |
| Bayındır 2022 | Bayındır, O.; Akyüz, G.; Sekban, N. | The effect of adding robot-assisted hand rehabilitation to conventional rehabilitation program following stroke: A randomized-controlled study | Turkish Journal of Physical Medicine & Rehabilitation (2587-1250) | 2022 | 68 | 2 | 254-261 | Not relevant |
| bdmsk 2018 | bdmsk, R. B. R. | Encourage the Practice of physical activity in patients after stroke via a Program of physical Exercises performed in the community: a feasibility study | https://trialsearch.who.int/Trial2.aspx?TrialID=RBR-6bdmsk | 2018 |  |  |  | Wrong study design - not RCT |
| Bearne 2019 | Bearne, L.; Galea Holmes, M.; Bieles, J.; Eddy, S.; Fisher, G.; Modarai, B.; Patel, S.; Peacock, J. L.; Sackley, C.; Volkmer, B.; Weinman, J. | Motivating Structured walking activity in people with Intermittent Claudication (MOSAIC): protocol for a randomised controlled trial of a physiotherapist-led, behavioural change intervention versus usual care in adults with intermittent claudication | BMJ Open | 2019 | 9 | 8 | e030002 | Not relevant |
| Bearne 2022 | Bearne, L.; Volkmer, B.; Dhouri, A.; Farran, D.; Fisher, G.; Galea Holmes, M.; Modarai, B.; Patel, S.; Peacock, J.; Sackley, C.; Weinman, J.; Bieles, J. | A physiotherapist-led, home-based walking intervention for peripheral arterial disease: mOtivating Structured walking activity for Intermittent Claudication (MOSAIC) randomised controlled trial | Physiotherapy (United Kingdom) | 2022 | 114 |  | e166 | Not relevant |
| Bearne 2022 | Bearne, L.M.; Bieles, J.; Peacock, J. | Home-Based, Walking Exercise Behavior Change Intervention vs Usual Care for Adults With Peripheral Artery Disease-Reply | JAMA: Journal of the American Medical Association | 2022 | 328 | 6 | 584-585 | Not relevant |
| Bearne 2022 | Bearne, L. M.; Volkmer, B.; Peacock, J.; Sekhon, M.; Fisher, G.; Galea Holmes, M. N.; Douiri, A.; Amirova, A.; Farran, D.; Quirke-McFarlane, S.; Modarai, B.; Sackley, C.; Weinman, J.; Bieles, J.; Collaboration, Mosaic Trial | Effect of a Home-Based, Walking Exercise Behavior Change Intervention vs Usual Care on Walking in Adults With Peripheral Artery Disease: the MOSAIC Randomized Clinical Trial | JAMA | 2022 | 327 | 14 | 1344-1355 | Not relevant |
| Beckie 2024 | Beckie, T. M.; Sengupta, A.; Dey, A. K.; Dutta, K.; Ji, M.; Chellappan, S. | A Mobile Health Behavior Change Intervention for Women With Coronary Heart Disease: a RANDOMIZED CONTROLLED PILOT STUDY | Journal of Cardiopulmonary Rehabilitation and Prevention | 2024 | 44 | 1 | 40-48 | Not relevant |
| Begrambekova 2022 | Begrambekova, Y. L.; Karanadze, N. A.; Plisyuk, A. G.; Orlova, Y. A. | Comprehensive physical rehabilitation of patients with heart failure: impact on Clinical and functional status and analysis of problems related to the enrollment. [Russian] | Russian Journal of Cardiology | 2022 | 27 | 2 | 21-28 | Full text not in English |
| Bei 2023 | Bei, N.; Long, D.; Bei, Z.; Chen, Y.; Chen, Z.; Xing, Z. | Effect of Water Exercise Therapy on Lower Limb Function Rehabilitation in Hemiplegic Patients with the First Stroke | Alternative Therapies in Health & Medicine | 2023 | 29 | 7 | 429-433 | Wrong comparator - co-interventions not balanced between arms |
| Beigien 2021 | Beigienė, A.; Petruševičiė, D.; Barasaitė, V.; Kubilius, R.; Macijauskienė, J. | Frailty and Different Exercise Interventions to Improve Gait Speed in Older Adults after Acute Coronary Syndrome | Medicina (Kaunas, Lithuania) | 2021 | 57 | 12 |  | Wrong intervention < 6 weeks |
| Beigiene 2021 | Beigienė, A.; Petruševičiė, D.; Barasaitė, V.; Kubilius, R.; Macijauskienė, J. | Cardiac Rehabilitation and Complementary physical Training in Elderly Patients after Acute Coronary Syndrome: A Pilot Study | Medicina | 2021 | 57 | 6 | 25 | Wrong intervention < 6 weeks |
| BelasDosSantos 2018 | Belas Dos Santos, M.; Barros de Oliveira, C.; Dos Santos, A.; Garabello Pires, C.; Dylewski, V.; Arida, R. M. | A Comparative Study of Conventional Physiotherapy versus Robot-Assisted Gait Training Associated to Physiotherapy in Individuals with Ataxia after Stroke | Behavioural Neurology | 2018 | 2018 |  |  | Not relevant |
| Belikova 2016 | Belikova, N. A.; Indyka, S. Y. | [The investigation into dynamics of depression level and the quality of life in the patients after myocardial infarction under the influence of the program of physical rehabilitation] | Voprosy Kurortologii, Fizioterapii i Lechebnoi Fizicheskoi Kultury | 2016 | 93 | 3 | 18-22 | Full text not in English |
| Bellomo 2020 | Bellomo, R.G.; Paolucci, T.; Saggino, A.; Pezzi, L.; Bramanti, A.; Cimino, V.; Tommasi, M.; Saggini, R. | The WeReha Project for an Innovative Home-Based Exercise Training in Chronic Stroke Patients: A Clinical Study | Journal of Central Nervous System Disease | 2020 |  |  | 1-12 | Wrong study design - not RCT |
| Benda 2015 | Benda, N. M.; Seeger, J. P.; Stevens, G. G.; Hijmans-Kersten, B. T.; van Dijk, A. P.; Bellersen, L.; Lamfers, E. J.; Hopman, M. T.; Thijssen, D. H. | Effects of High-Intensity Interval Training versus Continuous Training on physical Fitness, Cardiovascular Function and Quality of Life in Heart Failure Patients | PLoS ONE [Electronic Resource] | 2015 | 10 | 10 | e0141256 | Wrong comparator - exercise v exercise |
| Bennett 2017 | Bennett, J.; Coleman, B.; Gupta, S.; Sin, B.; Gilbert, C.; Hannibal, E.; Grove, T. | Football, a novel exercise pilot study in male phase iv cardiac rehabilitation patients | European Journal of Preventive Cardiology | 2017 | 24 | 1 | S40-S41 | Wrong study design - not RCT |
| Bensoussan 2018 | Bensoussan, L. | Effects of combined treatment by botulinum toxin and Lokomat on walking ability in chronic stroke (Lokomat) |  | 2018 |  |  |  | Not relevant |
| BerenguelSenen 2017 | Berenguel Senen, A.; Gallango Brejano, M.; Lazaro Salvador, M.; Chamon Sanchez De Los Silos, R.; Lozano Lazaro, G.; Diaz Jimenez, M.; Puentes Gutierrez, A.; Castillo Martin, J.; Abeytua Jimenez, M.; Rodriguez Padial, L. | Muscle efficiency: A new concept for cardiologist; Muscle efficiency improvement due to cardiac rehabilitation and its relationship with oxygen uptake | European Journal of Preventive Cardiology | 2017 | 24 | 1 | S35 | Wrong study design - not RCT |
| Bergfeldt 2019 | Bergfeldt, U.; Ingolfsdottir, E.; Berthold-Lindstedt, M.; Eriksson, M.; Julin, P. | Effects of aerobic training on memory, attention, and working memory in patients with stroke and traumatic brain injury | European Stroke Journal | 2019 | 4 |  | 793 | Not relevant |
| Bergqvist 2023 | Bergqvist, M.; Möller, M. C.; Björklund, M.; Borg, J.; Palmcrantz, S. | The impact of visuospatial and executive function on activity performance and outcome after robotic or conventional gait training, long-term after stroke-as part of a randomized controlled trial | PLoS ONE | 2023 | 18 | 3 | e0281212 | Not relevant |
| Bernhardt 2023 | Bernhardt, J.; Churilov, L.; Dewey, H.; Donnan, G.; Ellery, F.; English, C.; Gao, L.; Hayward, K.; Horgan, F.; Indredavik, B.; Johns, H.; Langhorne, P.; Lindley, R.; Martins, S.; Ali Katijjahbe, M.; Middleton, S.; Moodie, M.; Pandian, J.; Parsons, B.; Robinson, T.; Srikanth, V.; Thijs, V. | A phase III, multi-arm multi-stage covariate-adjusted response-adaptive randomized trial to determine optimal early mobility training after stroke (AVERT DOSE) | International Journal of Stroke | 2023 | 18 | 6 | 745-750 | Wrong intervention - does not meet exercise definition |
| Bernhardt 2015 | Bernhardt, J.; Langhorne, P.; Lindley, R. I.; Thrift, A. G.; Churilov, L.; Moodie, M.; Collier, J.; Ellery, F.; Dewey, H.; Donnan, G. | What 'dose' of mobilisation improves outcome? Dose response analysis of a very early rehabilitation trial (AVERT) | International Journal of Stroke | 2015 | 3) |  | 34-35 | Wrong intervention - does not meet exercise definition |
| Bernocchi 2018 | Bernocchi, P.; Vitacca, M.; La Rovere, M. T.; Volterrani, M.; Galli, T.; Baratti, D.; Paneroni, M.; Campolongo, G.; Sposato, B.; Scalvini, S. | Home-based telerehabilitation in older patients with chronic obstructive pulmonary disease and heart failure: a randomised controlled trial | Age and Ageing | 2018 | 47 | 1 | 82-88 | Wrong comparator - co-interventions not balanced between arms |
| Best 2018 | Best, J. R.; Eng, J. J.; Davis, J. C.; Hsiung, R.; Hall, P. A.; Middleton, L. E.; Graf, P.; Goldsmith, C. H.; Liu-Ambrose, T. | Study protocol for Vitality: a proof-of-concept randomised controlled trial of exercise training or complex mental and social activities to promote cognition in adults with chronic stroke | BMJ Open | 2018 | 8 | 3 | e021490 | Wrong comparator - active control |
| Betschart 2020 | Betschart, Martina; McFayden, Bradford J.; Nadeau, Sylvie | Lower limb joint moments on the fast belt contribute to a reduction of step length asymmetry over ground after split-belt treadmill training in stroke: A pilot study |  | 2020 | 36 |  | 989-999 | Not relevant |
| bgcs 2020 | bgcs, R. B. R. | Early physical exercise in ischemic stroke patients undergoing thrombolytic treatment | https://trialsearch.who.int/Trial2.aspx?TrialID=RBR-8bgcs3 | 2020 |  |  |  | Wrong intervention - does not meet exercise definition |
| Bhalla 2020 | Bhalla, N.; Shergill, N. | Comparison of Task Oriented Therapy and Modified Constraint Induced Movement Therapy along with Functional Electrical Stimulation to Improve Hand Function In Sub Acute Stroke survivors: a Randomized Control Trial | Indian Journal of Physiotherapy & Occupational Therapy | 2020 | 14 | 4 | 84-90 | Not relevant |
| Bian 2022 | Bian, M.; Shen, Y.; Huang, Y.; Wu, L.; Wang, Y.; He, S.; Huang, D.; Mao, Y. | A non-immersive virtual reality-based intervention to enhance lower-extremity motor function and gait in patients with subacute cerebral infarction: a pilot randomized controlled trial with 1-year follow-up | Frontiers in Neurology | 2022 | 13 |  |  | Wrong intervention < 6 weeks |
| Bills 2020 | Bills, S. E.; Pozehl, B. J.; Kupzyk, K.; Alonso, W.; Norman, J. F. | Heart camp: long-term outcomes on physical function and symptoms in patients with heart failure | Cardiopulmonary Physical Therapy Journal | 2020 | 31 | 1 | e8 | Not relevant |
| Bird 2016 | Bird, M. L.; Cannell, J.; Callisaya, M.; Moles, E.; Lane, K.; Tyson, A.; Rathjen, A.; Smith, S. | Study protocol of ?find technology?: a randomised controltrial investigating the feasibility and efficacy of controller-free interactive digitaltechnology in an inpatient stroke population | European Stroke Journal | 2016 | 1 | 1 | 339-340 | Wrong comparator - exercise v exercise |
| Bird 2016 | Bird, M. L.; Cannell, J.; Callisaya, M. L.; Moles, E.; Rathjen, A.; Lane, K.; Tyson, A.; Smith, S. | "FIND Technology": investigating the feasibility, efficacy and safety of controller-free interactive digital rehabilitation technology in an inpatient stroke population: study protocol for a randomized controlled trial | Trials | 2016 | 17 | 1 | 203 | Wrong comparator - exercise v exercise |
| Bird 2017 | Bird, M. L.; Cannell, J.; Jovic, E.; Rathjen, A.; Lane, K.; Tyson, A.; Callisaya, M.; Smith, S. | A randomized controlled trial investigating the efficacy of virtual reality in inpatient stroke rehabilitation | Archives of Physical Medicine and Rehabilitation | 2017 | 98 | 10 | e27 | Wrong intervention - does not meet exercise definition |
| Birke 2022 | Birke, H.; Foxvig, I.; Burns, K.; Toft, U.; Hansen, A. B. G.; Hauge, P. I.; Foghmar, S.; Mindegaard, R. B.; Jakobsen, L. M. | Heart Rehabilitation for All (HeRTA): protocol for a feasibility study and pilot randomized trial | PLoS ONE | 2022 | 17 | 6 | e0270159 | Not relevant |
| Birkett 2022 | Birkett, S. T.; Sinclair, J.; Seed, S. A.; Pymer, S.; Caldow, E.; Ingle, L.; Harwood, A. E.; Egun, A. | Effects of exercise prescribed at different levels of claudication pain on walking performance in patients with intermittent claudication: a protocol for a randomised controlled trial | Therapeutic Advances in Cardiovascular Disease | 2022 | 16 |  | 1-8 | Not relevant |
| Birmingham 2017 | Birmingham, University of Alabama at; Health, National Institutes of | Multimodal Exercise Training Poststroke |  | 2017 |  |  |  | Wrong comparator - exercise v exercise |
| Bispebjerg 2020 | Bispebjerg, University Hospital; Frederiksberg,; Laranjeiras, Instituto Nacional de Cardiologia de; Copenhagen, University of; Janeiro, Universidade Federal do Rio de | Brazilian Heart Insufficiency With TeleMedicine |  | 2020 |  |  |  | Not relevant |
| Bittencourt 2017 | Bittencourt, H. S.; Cruz, C. G.; David, B. C.; Rodrigues, E., Jr.; Abade, C. M.; Junior, R. A.; Carvalho, V. O.; Dos Reis, F. B. F.; Gomes Neto, M. | Addition of non-invasive ventilatory support to combined aerobic and resistance training improves dyspnea and quality of life in heart failure patients: a randomized controlled trial | Clinical Rehabilitation | 2017 | 31 | 11 | 1508-1515 | Not relevant |
| Bizovièar 2016 | Bizovièar, N.; Rudolf, M.; Javh, M.; Goljar, N.; Rudel, D.; Obr?an, D.; et al., | Effects of home exercise assisted by written and video instructions in patients after stroke | Rehabilitacija | 2016 | 3 |  | 26-32 | Full text not in English |
| Blumberg 2018 | Blumberg, Y.; Amon, E.; Hijazi, B.; Ertracht, O.; Goldenberg, I.; Klempfner, R.; Atar, S. | The Effect of Two Training Protocols on Post Exercise Lactate Clearance in Heart Failure Patients: 1020 Board #281 May 30 3:30 PM - 5:00 PM...American College of Sports Medicine Annual Meeting, May 29-June 2, 2018, Minneapolis, Minnesota | Medicine & Science in Sports & Exercise | 2018 | 50 |  | 242-242 | Wrong comparator - exercise v exercise |
| Blumenthal 2016 | Blumenthal, J. A.; Sherwood, A.; Smith, P. J.; Watkins, L.; Mabe, S.; Kraus, W. E.; Ingle, K.; Miller, P.; Hinderliter, A. | Enhancing Cardiac Rehabilitation With Stress Management Training: A Randomized, Clinical Efficacy Trial | Circulation | 2016 | 133 | 14 | 1341-50 | Not relevant |
| Bo-Kyoung 2020 | Bo-Kyoung, S.; So-Young, H.; Jung-Woo, J.; Ha-Na, K. | The Effect of Task-based upper Limb Training on Activities of Daily Living and Upper Limb Function in Chronic Stroke Patients | Medico-Legal Update | 2020 | 20 | 1 | 2069-2073 | Wrong intervention - does not meet exercise definition |
| BoaSorteSilva 2017 | Boa Sorte Silva, N. C.; Gregory, M. A.; Gill, D. P.; Petrella, R. J. | Multiple-modality exercise and mind-motor training to improve cardiovascular health and fitness in older adults at risk for cognitive impairment: A randomized controlled trial | Archives of Gerontology and Geriatrics | 2017 | 68 |  | 149-160 | Not relevant |
| Bobenko 2018 | Bobenko, A.; Bartels, I.; Munch, M.; Trippel, T.; Lindhorst, R.; Nolte, K.; Herrmann-Lingen, C.; Halle, M.; Duvinage, A.; Dungen, H. D.; Gelbrich, G.; Tschope, C.; Hasenfuss, G.; Wachter, R.; Pieske, B.; Edelmann, F. | Amount or intensity? Potential targets of exercise interventions in patients with heart failure with preserved ejection fraction | ESC Heart Failure | 2018 | 5 | 1 | 53-62 | Not relevant |
| Boidin 2021 | Boidin, M.; David, L. P.; Trachsel, L. D.; Gayda, M.; Tremblay, J.; Lalonge, J.; Juneau, M.; Nigam, A.; Henri, C. | Impact of 2 different aerobic periodization training protocols on left ventricular function in patients with stable coronary artery disease: an exploratory study | Applied Physiology, Nutrition, & Metabolism = Physiologie Appliquee, Nutrition et Metabolisme | 2021 | 46 | 5 | 436-442 | Wrong comparator - exercise v exercise |
| Boidin 2019 | Boidin, M.; Gayda, M.; Henri, C.; Hayami, D.; Trachsel, L. D.; Besnier, F.; Lalonge, J.; Juneau, M.; Nigam, A. | Effects of interval training on risk markers for arrhythmic death: a randomized controlled trial | Clinical Rehabilitation | 2019 | 33 | 8 | 1320-1330 | Wrong comparator - exercise v exercise |
| Boidin 2019 | Boidin, M.; Trachsel, L.; Nigam, A.; Juneau, M.; Tremblay, J.; Gayda, M. | NON-LINEAR IS NOT SUPERIOR TO LINEAR AEROBIC PERIODIZATION IN CORONARY PATIENTS | Canadian Journal of Cardiology | 2019 | 35 | 10 | S86 | Wrong comparator - exercise v exercise |
| Boidin 2020 | Boidin, M.; Trachsel, L. D.; Nigam, A.; Juneau, M.; Tremblay, J.; Gayda, M. | Non-linear is not superior to linear aerobic training periodization in coronary heart disease patients | European Journal of Preventive Cardiology | 2020 | 27 | 16 | 1691-1698 | Wrong comparator - exercise v exercise |
| Bond 2016 | Bond, D. | The Stroke and Exercise Program (StEP) |  | 2016 |  |  |  | Wrong comparator - exercise v exercise |
| Bonino-Pulejo 2016 | Bonino-Pulejo, Irccs Centro Neurolesi | BTs Nirvana and Post-stroke Rehab |  | 2016 |  |  |  | Not relevant |
| Borg 2023 | Borg, S.; Oberg, B.; Nilsson, L.; Alfredsson, J.; Soderlund, A.; Back, M. | Effectiveness of a behavioral Medicine intervention in physical therapy on secondary psychological outcomes and health-related quality of life in exercise-based cardiac rehabilitation: a randomized, controlled trial | BMC sports Science, Medicine & Rehabilitation | 2023 | 15 | 1 | 42 | Not relevant |
| Borg 2017 | Borg, S.; Oberg, B.; Nilsson, L.; Soderlund, A.; Back, M. | The role of a behavioural Medicine intervention in physiotherapy for the effects of rehabilitation outcomes in exercise-based cardiac rehabilitation (ECRA) - the study protocol of a randomised, controlled trial | BMC Cardiovascular Disorders | 2017 | 17 | 1 | 134 | Not relevant |
| Borg 2020 | Borg, S.; Oberg, B.; Nilsson, L.; Soderlund, A.; Back, M. | The added value of a behavioral Medicine intervention in physiotherapy on adherence and physical fitness in exercise-based cardiac rehabilitation (ECRA): A randomised, controlled trial | Patient Preference and Adherence | 2020 | 14 |  | 2517-2529 | Not relevant |
| Borges 2016 | Borges, D. L.; Silva, M. G.; Silva, L. N.; Fortes, J. V.; Costa, E. T.; Assuncao, R. P.; Lima, C. M.; da Silva Nina, V. J.; Bernardo-Filho, M.; Caputo, D. S. | Effects of Aerobic Exercise Applied Early After Coronary Artery Bypass Grafting on Pulmonary Function, Respiratory Muscle Strength, and Functional Capacity: A Randomized Controlled Trial | Journal of Physical Activity & Health | 2016 | 13 | 9 | 946-51 | Wrong intervention < 6 weeks |
| Borges 2018 | Borges, J. P.; Nascimento, A. R.; Lopes, G. O.; Medeiros-Lima, D. J. M.; Coelho, M. P.; Nascimento, P. M. C.; Kopiler, D. A.; Matsuura, C.; Mediano, M. F. F.; Tibirica, E. | The impact of exercise frequency upon microvascular endothelium function and oxidative stress among patients with coronary artery disease | Clinical Physiology & Functional Imaging | 2018 | 38 | 5 | 840-846 | Wrong study design - not RCT |
| Borstad 2022 | Borstad, A.; Nichols-Larsen, D.; Uswatte, G.; Strahl, N.; Simeo, M.; Proffitt, R.; Gauthier, L. | Tactile Sensation Improves Following Motor Rehabilitation for Chronic Stroke: The VIGoROUS Randomized Controlled Trial | NeuroRehabilitation & Neural Repair | 2022 | 36 | 8 | 525-534 | Not relevant |
| Bortsova 2021 | Bortsova, M. A.; Demchenko, E. A.; Bautin, A. E.; Fedotov, P. A.; Ganenko, O. S.; Lelyavina, T. A.; Simonenko, M. A.; Korneva, L. O.; Fedorova, M. A.; Sitnikova, M. Yu | Impact of physical training on functional and haemodynamic characteristics of "inotrope-dependent" patients with chronic heart failure at class III-IV. [Russian] | Arterial Hypertension (Russian Federation) | 2021 | 25 | 6 | 526-542 | Full text not in English |
| Bortsova 2023 | Bortsova, M. A.; Demchenko, E. A.; Fedotov, P. A.; Ganenko, O. S.; Osipova, M. A.; Korneva, L. O.; Musaeva, B. B.; Sazonova, Y. V.; Simonenko, M. A.; Sitnikova, M. Y. | Tolerability of an Individualized physical Rehabilitation Program in Patients Dependent on Inotropic Support With End-Stage Chronic Heart Failure | Kardiologiia | 2023 | 63 | 11 | 36-45 | Full text not in English |
| Bortsova 2021 | Bortsova, M. A.; Sitnikova, M. Y. U.; Demchenko, E. A.; Bautin, A. E.; Fedotov, P.; Fedorova, M. A. | Lactate and oxygen blood levels during physical rehabilitation in inotrope-dependent patients with chronic heart failure stabilized of NYHA class III-IV | European Journal of Heart Failure | 2021 | 23 | 2 | 141 | Full text not in English |
| Bosomworth 2019 | Bosomworth, N. John | Impediments to Clinical application of exercise interventions in the treatment of cardiometabolic disease | Canadian Family Physician / Médecin de Famille Canadien | 2019 | 65 | 3 | 164-170 | Not relevant |
| Bouisset 2020 | Bouisset, F.; Ruidavets, J. B.; Bongard, V.; Taraszkiewicz, D.; Berard, E.; Galinier, M.; Carrie, D.; Elbaz, M.; Ferrieres, J. | Long-term Prognostic Impact of physical activity in Patients With Stable Coronary Heart Disease | American Journal of Cardiology | 2020 | 125 | 2 | 176-181 | Wrong study design - not RCT |
| Boumer 2019 | Boumer, T. C.; Firmino, T. C.; Devetak, G. F.; Martello, S. K.; de Lima Moser, A.; Manffra, E. F. | EFEITOS DO TREINO DE MARCHA COM SUPORTE PARCIAL DE PESO CORPORAL ASSOCIADO A FISIOTERAPIA CONVENCIONAL SOBRE O EQUILÍBRIO FUNCIONAL E A INDEPENDÊNCIA DA MARCHA PÓS-AVC: ESTUDO CLÍNICO RANDOMIZADO | Revista Inspirar Movimento & Saude | 2019 | 19 | 4 | 1-21 | Full text not in English |
| Bove 2020 | Bove, K. B.; Nilsson, M.; Pedersen, L. R.; Mikkelsen, N.; Suhrs, H. E.; Astrup, A.; Prescott, E. | Comprehensive treatment of microvascular angina in overweight women - a randomized controlled pilot trial | PLoS ONE [Electronic Resource] | 2020 | 15 | 11 | e0240722 | Wrong comparator - co-interventions not balanced between arms |
| Bovonsunthonchai 2020 | Bovonsunthonchai, S.; Aung, N.; Hiengkaew, V.; Tretriluxana, J. | A randomized controlled trial of motor imagery combined with structured progressive circuit class therapy on gait in stroke survivors | Scientific Reports | 2020 | 10 | 1 | 6945 | Wrong intervention - does not meet exercise definition |
| Boyne 2022 | Boyne, P.; Billinger, S. A.; Reisman, D. S.; Awosika, O. O.; Buckley, S.; Burson, J.; Carl, D.; DeLange, M.; et al. | A Multicenter Randomized Comparison of High-Intensity Interval Training and Moderate-Intensity Exercise to Recover Walking Post-Stroke: Results of the HIT-Stroke Trial | medRxiv. | 2022 | 2 |  |  | Wrong comparator - exercise v exercise |
| Boyne 2023 | Boyne, P.; Billinger, S. A.; Reisman, D. S.; Awosika, O. O.; Buckley, S.; Burson, J.; Carl, D.; DeLange, M.; et al. | Optimal Intensity and Duration of Walking Rehabilitation in Patients With Chronic Stroke: A Randomized Clinical Trial | JAMA Neurology | 2023 | 80 | 4 | 342-351 | Wrong comparator - exercise v exercise |
| Boyne 2023 | Boyne, P.; Miller, A.; Schwab, S. M.; Sucharew, H.; Carl, D.; Billinger, S. A.; Reisman, D. S. | Training parameters and longitudinal adaptations that most strongly mediate walking capacity gains from high-intensity interval training post-stroke | medRxiv. | 2023 | 23 |  |  | Wrong comparator - exercise v exercise |
| Brainin 2015 | Brainin, M.; Matz, K.; Nemec, M.; Teuschl, Y.; Dachenhausen, A.; Asenbaum-Nan, S.; Bancher, C.; Kepplinger, B.; Oberndorfer, S.; Pinter, M.; Schnider, P.; Tuomilehto, J. | Prevention of poststroke cognitive decline: ASPIS--a multicenter, randomized, observer-blind, parallel group Clinical trial to evaluate multiple lifestyle interventions--study design and baseline characteristics | International Journal of Stroke | 2015 | 10 | 4 | 627-35 | Not relevant |
| Brainin 2016 | Brainin, M.; Teuschl, Y.; Matz, K.; Dachenhausen, A.; Firlinger, B.; Reiter, M.; Tuomilehto, J. | Testing the intensity of multiple, complex interventions for life-style modification in randomized stroke trials | Stroke. Conference: American Heart Association/American Stroke Association | 2016 | 47 | 1 |  | Not relevant |
| Brauer 2022 | Brauer, S. G.; Kuys, S. S.; Ada, L.; Paratz, J. D. | IMproving physical ACtivity after stroke via Treadmill training (IMPACT) and self-management: A randomized trial | International Journal of Stroke | 2022 | 17 | 10 | 1137-1144 | Wrong comparator - active control |
| Brauer 2018 | Brauer, S. G.; Kuys, S. S.; Paratz, J. D.; Ada, L. | Improving physical activity after stroke via treadmill training and self management (IMPACT): a protocol for a randomised controlled trial | BMC Neurology | 2018 | 18 | 1 | 13 | Wrong comparator - active control |
| Brauer 2021 | Brauer, S. G.; Kuys, S. S.; Paratz, J. D.; Ada, L. | High-intensity treadmill training and self-management for stroke patients undergoing rehabilitation: a feasibility study | Pilot and Feasibility Studies | 2021 | 7 | 1 |  | Wrong study design - not RCT |
| Bravo-Escobar 2017 | Bravo-Escobar, R.; Gonzalez-Represas, A.; Gomez-Gonzalez, A. M.; Montiel-Trujillo, A.; Aguilar-Jimenez, R.; Carrasco-Ruiz, R.; Salinas-Sanchez, P. | Effectiveness and safety of a home-based cardiac rehabilitation programme of mixed surveillance in patients with ischemic heart disease at moderate cardiovascular risk: A randomised, controlled Clinical trial | BMC Cardiovascular Disorders | 2017 | 17 | 1 | 66 | Wrong comparator - exercise v exercise |
| Bricca 2022 | Bricca, A.; Jager, M.; Dideriksen, M.; Rasmussen, H.; Nyberg, M.; Pedersen, J. R.; Zangger, G.; Andreasson, K. H.; Skou, S. T. | Personalised exercise therapy and self-management support for people with multimorbidity: development of the MOBILIZE intervention | Pilot and Feasibility Studies | 2022 | 8 | 1 |  | Not relevant |
| Brigham 2024 | Brigham,; Hospital, Women's | Reducing Heart Failure Risk in Late-Life With physical activity |  | 2024 |  |  |  | Not relevant |
| Brito 2020 | Brito, S. A. F.; Aguiar, L. T.; Garcia, L. N.; Peniche, P. D. C.; Reis, Mtfd; Faria, Cdcm | Cardiopulmonary exercise testing and aerobic treadmill training after stroke: feasibility of a controlled trial | Journal of Stroke and Cerebrovascular Diseases | 2020 | 29 | 7 | 104854 | Wrong comparator - exercise v exercise |
| Britto 2017 | Britto, R. R.; Chaves, G.; Verardo, L. P.; Servio, T. C.; Martins, T. H. S.; Loures, J. B.; Correa, U. A. C.; Reis, M. T. F.; Pantuso, D.; Pereira, D. A. G. | Effects of high-intensity interval training versus moderate continuous training on functional capacity and quality of life in heart failure patients: a pilot study | Journal of Cardiopulmonary Rehabilitation and Prevention | 2017 | 37 | 6 | 456 | Wrong comparator - exercise v exercise |
| Britto 2019 | Britto, R. R.; Lima, A. P.; Nascimento, I. O.; Malagoli, R. C.; Santos, A. E. P.; Nogueira, T. S.; Oliveira, A. C. A.; Pereira, D. A. G. | Effectiveness of home-based cardiac rehabilitation in functional capacity: a pilot study in a middle-income country | European Journal of Preventive Cardiology | 2019 | 26 |  | S50 | Wrong comparator - exercise v exercise |
| Brock 2022 | Brock, K. A.; Luke, C.; Tillyard, J.; Simondson, J.; Black, S. | Improving Directional Control of the Upper Limb in Severe Stroke: efficacy of the Bobath Concept: a Pilot Randomised Trial | New zealand Journal of Physiotherapy | 2022 | 50 | 3 | 117-125 | Wrong intervention - does not meet exercise definition |
| Broderick 2021 | Broderick, M.; Almedom, L.; Burdet, E.; Burridge, J.; Bentley, P. | Self-Directed Exergaming for Stroke Upper Limb Impairment Increases Exercise Dose Compared to Standard Care | NeuroRehabilitation and Neural Repair | 2021 | 35 | 11 | 974-985 | Not relevant |
| Broderick 2020 | Broderick, M.; Bentley, P.; Burridge, J. | A multicentre pilot randomised control of an adapted mobile rehabilitation system to increase self-directed rehabilitation in stroke survivors with upper limb weakness | International Journal of Stroke | 2020 | 15 | 1 | 607-608 | Not relevant |
| Broderick 2020 | Broderick, M.; Bentley, P.; Burridge, J.; Burdet, E. | Self-administered gaming exercises for stroke arm disability increase exercise duration by more than two-fold and repetitions more than ten-fold compared to standard care | International Journal of Stroke | 2020 | 15 | 1 | 255 | Not relevant |
| Broderick 2024 | Broderick, M.; Burridge, J.; Demain, S.; Johnson, L.; Brereton, J.; O'Shea, R.; Bentley, P. | Multicentre pilot randomised control trial of a self-directed exergaming intervention for poststroke upper limb rehabilitation: Research protocol | BMJ Open | 2024 | 14 | 1 |  | Not relevant |
| Brodtmann 2017 | Brodtmann, A. | The effect of prescribed exercise intervention on brain volume and function after stroke |  | 2017 |  |  |  | Wrong comparator - active control |
| Brodtmann 2022 | Brodtmann, A. | TARGETING EXERCISE TO PREVENT VASCULAR COGNITIVE IMPAIRMENT: THE POST-ISCHAEMIC STROKE CARDIOVASCULAR EXERCISE STUDY | Neuroepidemiology | 2022 | 56 | 1 | 16 | Wrong comparator - active control |
| Brough 2019 | Brough, L. G.; Kautz, S. A.; Bowden, M. G.; Gregory, C. M.; Neptune, R. R. | Merged plantarflexor muscle activity is predictive of poor walking performance in post-stroke hemiparetic subjects | Journal of Biomechanics | 2019 | 82 |  | 361-367 | Not relevant |
| Brouwers 2021 | Brouwers, R.; Kraal, J. J.; Regis, M.; Spee, R. F.; Kemps, H. M. C. | Effectiveness of cardiac telerehabilitation with relapse prevention compared to centrebased cardiac rehabilitation: results from the Smart Care-CAD randomised controlled trial | European Journal of Preventive Cardiology | 2021 | 28 | 1 | i320 | Wrong comparator - active control |
| Brouwers 2017 | Brouwers, R. W.; Kraal, J. J.; Traa, S. C.; Spee, R. F.; Oostveen, L. M.; Kemps, H. M. | Effects of cardiac telerehabilitation in patients with coronary artery disease using a personalised patient-centred web application: protocol for the SmartCare-CAD randomised controlled trial | BMC Cardiovascular Disorders | 2017 | 17 | 1 | 46 | Wrong comparator - active control |
| Brouwers 2021 | Brouwers, R. W. M.; van der Poort, E. K. J.; Kemps, H. M. C.; van den Akker-van Marle, M. E.; Kraal, J. J. | Cost-effectiveness of Cardiac Telerehabilitation With Relapse Prevention for the Treatment of Patients With Coronary Artery Disease in the Netherlands | JAMA Network Open | 2021 | 4 | 12 | e2136652 | Wrong comparator - active control |
| Brown 2021 | Brown, J. D.; Sato, R.; Morley, J. E. | Association between pneumonia, fracture, stroke, heart attack and other hospitalizations with changes in mobility disability and gait speed in older adults | Journal of Clinical Medicine | 2021 | 10 | 17 |  | Wrong population - under 80% vascular |
| Brubaker 2022 | Brubaker, P. H.; Nicklas, B.; Houston, D.; Hundley, W. G.; Chen, H.; Molina, A.; Lyles, M.; Nelson, M.; Upadhya, B.; Newland, R.; Kitzman, D. W. | A Randomized, Controlled Trial of Resistance Training Added to Caloric Restriction Plus Aerobic Exercise Training in Obese Heart Failure With Preserved Ejection Fraction | Circulation | 2022 | 146 |  |  | Wrong comparator - exercise v exercise |
| Brubaker 2023 | Brubaker, P. H.; Nicklas, B. J.; Houston, D. K.; Hundley, W. G.; Chen, H.; Molina, A. J. A.; Lyles, W. M.; Nelson, B.; Upadhya, B.; Newland, R.; Kitzman, D. W. | A Randomized, Controlled Trial of Resistance Training Added to Caloric Restriction Plus Aerobic Exercise Training in Obese Heart Failure With Preserved Ejection Fraction | Circulation: Heart Failure | 2023 | 16 | 2 | e010161 | Wrong comparator - exercise v exercise |
| BrunoMiguelDelgado 2022 | Bruno Miguel Delgado, B. M.; Re Novo, A. N. D.; Lopes, I. V. O.; Sousa, Luis; Klompstra, Leonie | Effectiveness and reproducibility of an exercise training program: THE ERIC-HF-multicenter randomized controlled trial | European Journal of Heart Failure | 2022 | 24 |  | 261 | Wrong intervention < 6 weeks |
| Bruyneel 2023 | Bruyneel, A. V.; Pourchet, T.; Reinmann, A. | Dance after stroke improves motor recovery in the subacute phase: a randomized controlled trial | Heliyon | 2023 | 9 | 11 | e22275 | Wrong intervention - does not meet exercise definition |
| Bubnova 2019 | Bubnova, M. G.; Aronov, D. M. | Clinical effects of a one-year cardiac rehabilitation program using physical training after myocardial infarction in patients of working age with different rehabilitation potentials. [Russian] | Cardiovascular Therapy and Prevention (Russian Federation) | 2019 | 18 | 5 | 27-37 | Full text not in English |
| Bubnova 2020 | Bubnova, M. G.; Aronov, D. M. | Physical rehabilitation after acute myocardial infarction: Focus on body weight. [Russian] | Russian Journal of Cardiology | 2020 | 25 | 5 | 15-23 | Full text not in English |
| Buccellato 2020 | Buccellato, K. H.; Nordstrom, M.; Murphy, J. M.; Burdea, G. C.; Polistico, K.; House, G.; Kim, N.; Grampurohit, N.; Sorensen, J.; Isaacson, B. M.; Pasquina, P. F. | A Randomized Feasibility Trial of a Novel, Integrative, and Intensive Virtual Rehabilitation Program for Service Members Post-Acquired Brain Injury | Military Medicine | 2020 | 185 | 1-2 | e203-e211 | Wrong population - under 80% vascular |
| Buffalo 2018 | Buffalo, State University of New York at; Health, Eunice Kennedy Shriver National Institute of Child; Development, Human | Smartphone and 3D Printing Based Home Rehabilitation System for Chronic Stroke |  | 2018 |  |  |  | Wrong study design - not RCT |
| Buick 2016 | Buick, A. R.; Kowalczewski, J.; Carson, R. G.; Prochazka, A. | Tele-Supervised FES-Assisted Exercise for Hemiplegic Upper Limb | IEEE Transactions on Neural Systems and Rehabilitation Engineering | 2016 | 24 | 1 | 79-87 | Not relevant |
| Buli?ska 2016 | Buli?ska, K.; Kropielnicka, K.; Jasi?ski, T.; Wojcieszczyk-Latos, J.; Pilch, U.; G, D. browska; Skórkowska-Telichowska, K.; Ka?ka, D.; Zywar, K.; Paszkowski, R.; Wo?niewski, M.; Szuba, A.; Jasi?ski, R. | Nordic pole walking improves walking capacity in patients with intermittent claudication: a randomized controlled trial | Disability and Rehabilitation | 2016 | 38 | 13 | 1318-1324 | Wrong comparator - exercise v exercise |
| Burger 2021 | Burger, H.; Erjavec, T.; Kriznar, A.; Kostanjsek, L.; Robida, T.; Vipavec, B.; Karan, K. | Aerobic Training in the Initial Prosthetic Training Phase for Patients with Lower Limb Loss due to Peripheral Vascular Disease | Prosthetics and Orthotics International | 2021 | 45 | 6 | 63 | Wrong comparator - exercise v exercise |
| Burgess 2023 | Burgess, Laura; Smith, Sasha; Babber, Adarsh; Shalhoub, Joseph; Fiorentino, Francesca; de la Rosa, Consuelo Nohpal; Klimowska-Nassar, Natalia; Epstein, David M.; Troncoso, Daniel Pérez; Braithwaite, Bruce; Chetter, Ian; Coulston, James; Gohel, Manjit; Hinchliffe, Robert; Stansby, Gerard; Davies, Alun H. | Neuromuscular electrical stimulation as an adjunct to standard care in improving walking distances in intermittent claudication patients: the NESIC RCT | Efficacy & Mechanism Evaluation (EME) | 2023 | 10 | 2 | i-71 | Not relevant |
| Bushnell 2018 | Bushnell, C.D.; Duncan, P. W.; Lycan, S. L.; Condon, C.N.; Pastva, A.M.; Lutz, B.J.; Halladay, J.R.; et al. | A Person-Centered Approach to Poststroke Care: The COMprehensive Post-Acute Stroke Services Model | Journal of the American Geriatrics Society | 2018 | 66 | 5 | 1025-1030 | Not relevant |
| Busk 2020 | Busk, H.; Kwakkel, G.; Skou, S.; Wienecke, T. | Neuromuscular electrical stimulation in addition to exercise therapy in acute ischemic stroke-a randomized Clinical trial | International Journal of Stroke | 2020 | 15 | 1 | 221 | Not relevant |
| BusraAlkan 2018 | Busra Alkan, B.; Ozalevli, S.; Gullu, N. | Assesment of symptom limited stair climbing test efficacy in patients with chronic heart failure | European Journal of Heart Failure | 2018 | 20 | 1 | 445 | Not relevant |
| BustamanteValles 2016 | Bustamante Valles, K.; Montes, S.; Madrigal Mde, J.; Burciaga, A.; Martí­nez, M. E.; Johnson, M. J. | Technology-assisted stroke rehabilitation in Mexico: a pilot randomized trial comparing traditional therapy to circuit training in a Robot/technology-assisted therapy gym | Journal of NeuroEngineering and Rehabilitation | 2016 | 13 | 1 | 83 | Not relevant |
| Buszman 2018 | Buszman, Pawel; Poland, American Heart of | Utilisation of TeleMedicine in Optimal Cardiac Rehabilitation Program in Patients After Myocardial Revascularization |  | 2018 |  |  |  | Wrong comparator - co-interventions not balanced between arms |
| Butler 2020 | Butler, V. | Service evaluation of lifestyle integrated functional exercise program for falls prevention in early supported discharge stroke patients | International Journal of Stroke | 2020 | 15 | 1 | 349 | Not relevant |
| Butler 2021 | Butler, V.; Dinsdale, J.; Butler, L.; French, K.; De Cruz, R.; Reynolds, N.; Riley, R.; Fountain, G.; McQue, K.; Chivhunga, M.; Thompson, J.; Gofton-Howard, M.; Scott, F.; Coates, S.; Foster, C. | Service evaluation of lifestyle integrated functional exercise program for falls prevention in early supported discharge stroke patients | Physiotherapy (United Kingdom) | 2021 | 113 | 1 | e74 | Not relevant |
| Butts 2018 | Butts, B.; Butler, J.; Dunbar, S. B.; Corwin, E.; Gary, R. A. | Effects of Exercise on ASC Methylation and IL-1 Cytokines in Heart Failure | Medicine & Science in Sports & Exercise | 2018 | 50 | 9 | 1757-1766 | Wrong comparator - active control |
| Butts 2018 | Butts, B.; Dunbar, S. B.; Butler, J.; Gary, R. A. | Effects of an exercise intervention on asc methylation and IL-1 cytokines in persons with heart failure | Journal of Cardiac Failure | 2016 | 22 |  | S13 | Wrong comparator - active control |
| Butts 2018 | Butts, Brittany | Effects of Exercise on Epigenetic Pathways in Persons with Heart Failure | Effects of Exercise on Epigenetic Pathways in Persons with Heart Failure | 2016 |  |  | 1-1 | Wrong comparator - active control |
| by9h 2018 | (WHO) International Clinical Trials Registry Platform | Influence of Aquatic Physiotherapy in the weight-bearing symmetry in the sit-to-stand transfer in hemiparetics after Stroke | https://trialsearch.who.int/Trial2.aspx?TrialID=RBR-6by9h2 | 2018 |  |  |  | Wrong intervention - does not meet exercise definition |
| Byl 2015 | Byl, N.; Zhang, W.; Coo, S.; Tomizuka, M. | Clinical impact of gait training enhanced with visual kinematic biofeedback: Patients with Parkinson's disease and patients stable post stroke | Neuropsychologia | 2015 | 79 | Pt B | 332-43 | Not relevant |
| Cai 2019 | Cai, H.; Zheng, Y.; Liu, Z.; Zhang, X.; Li, R.; Shao, W.; Wang, L.; Zou, L.; Cao, P. | Effect of pre-discharge cardiopulmonary fitness on outcomes in patients with ST-elevation myocardial infarction after percutaneous coronary intervention | BMC Cardiovascular Disorders | 2019 | 19 | 1 | 210 | Wrong study design - not RCT |
| Cai 2022 | Cai, Y.; Kang, L.; Li, H.; Luo, Y.; Wen, J.; Gong, Z.; Chu, Q.; Qiu, Y.; Luo, C.; Chen, K.; Zhao, X.; Li, R. | Effects of Home-Based Baduanjin Exercise on Left Ventricular Remodeling in Patients With Acute Anterior ST-Segment Elevation Myocardial Infarction: study Protocol for a Randomized Controlled Trial | Frontiers in Cardiovascular Medicine | 2022 | 9 |  | 778583 | Wrong comparator - exercise v exercise |
| Calabro 2019 | Calabro, R. S.; Accorinti, M.; Porcari, B.; Carioti, L.; Ciatto, L.; Billeri, L.; Andronaco, V. A.; Galletti, F.; Filoni, S.; Naro, A. | Does hand robotic rehabilitation improve motor function by rebalancing interhemispheric connectivity after chronic stroke? Encouraging data from a randomised-Clinical-trial | Clinical Neurophysiology | 2019 | 130 | 5 | 767-780 | Not relevant |
| Cao 2018 | Cao, R.; Hong, Y.; Zheng, H.; Li, Q.; Mi, Q.; Yang, J. | Trial progress of aerobic exercise-based cardiac rehabilitation in chinese patients with atherosclerotic heart disease | Atherosclerosis supplements | 2018 | 32 |  | 155-156 | Wrong comparator - exercise v exercise |
| Cao 2021 | Cao, R. Y.; Zheng, H.; Hong, Y.; Zheng, Y.; Ding, Y.; Zhao, L.; Li, H.; Li, Q.; Yuan, W.; Liu, S.; Wang, L.; Yang, J. | Cardiac Rehabilitation with Targeted Intensity Improves Cardiopulmonary Functions Accompanying with Reduced Copeptin Level in Patients with Coronary Artery Disease | Journal of Cardiovascular Translational Research | 2021 | 14 | 2 | 317-326 | Wrong comparator - exercise v exercise |
| Cao 2018 | Cao, R. Y.; Zheng, H.; Mi, Q.; Li, Q.; Yuan, W.; Ding, Y.; Yang, J. | Aerobic exercise-based cardiac rehabilitation in Chinese patients with coronary heart disease: study protocol for a pilot randomized controlled trial | Trials [Electronic Resource] | 2018 | 19 | 1 | 363 | Wrong comparator - exercise v exercise |
| Capisizu 2016 | Capisizu, A.; Aurelian, S. M.; Mirsu-Paun, A.; Badiu, C.; Dascalescu, R.; Nica, A. S.; Zamfirescu, A. | A key for post stroke rehabilitation in the elderly | Acta Medica Mediterranea | 2016 | 32 | 4 | 1003-1007 | Wrong intervention < 6 weeks |
| Capizzi 2018 | Capizzi, A.; Rudolph, L. S.; Hayes, H. A.; Teramoto, M.; Edgley, S. R. | Assessing stroke patients' steps using the Garmin Vivofit3: A validity study | PM and R | 2018 | 10 | 9 | S36 | Not relevant |
| Caputo 2023 | Caputo, C.; Kowaleski, J.; Thaut, M.; Loria, T. | PAIRED NEUROLOGIC MUSIC THERAPY AND TRANSCRANIAL DIRECT CURRENT STIMULATION FOR UPPER EXTREMITIES FOLLOWING STROKE | International Journal of Stroke | 2023 | 18 | 3 | 233 | Not relevant |
| Carlson 2016 | Carlson, D. J.; McFarlane, J. R.; Dieberg, G.; Smart, N. A. | Isometric handgrip exercise to reduce hypertension for stroke prevention and recovery | Archives of Physical Medicine and Rehabilitation | 2016 | 96 | 12 | e25 | Not relevant |
| Carolina 2019 | Carolina, University of South | Arm Training in Standing After Stroke |  | 2019 |  |  |  | Not relevant |
| Carr 2024 | Carr, E.; Whiston, A.; O'Reilly, S.; O. Donoghue M; Cardy, N.; Carter, D.; Glynn, L.; Walsh, J. C.; et al. | Sequential multiple assignment randomised trial to develop an adaptive mobile health intervention to increase physical activity in people poststroke in the community setting in Ireland: TAPAS trial protocol | BMJ Open | 2024 | 14 | 1 |  | Wrong comparator - co-interventions not balanced between arms |
| Carrasco-Poyatos 2022 | Carrasco-Poyatos, M.; Granero-Gallegos, A.; Lopez-Garcia, G. D.; Lopez-Osca, R. | HRV-Guided Training for Elders after Stroke: A Protocol for a Cluster-Randomized Controlled Trial | International Journal of Environmental Research & Public Health [Electronic Resource] | 2022 | 19 | 17 | 31 | Wrong comparator - exercise v exercise |
| Carrico 2019 | Carrico, C.; Annichiarico, N.; Powell, E. S.; Westgate, P. M.; Sawaki, L. | Chronicity of Stroke Does Not Affect Outcomes of Somatosensory Stimulation Paired With Task-Oriented Motor Training: A Secondary Analysis of a Randomized Controlled Trial | Archives of Rehabilitation Research and Clinical Translation | 2019 | 1 |  | 1-2 | Not relevant |
| Caruso 2015 | Caruso, F. R.; Arena, R.; Phillips, S. A.; Bonjorno, J. C., Jr.; Mendes, R. G.; Arakelian, V. M.; Bassi, D.; Nogi, C.; Borghi-Silva, A. | Resistance exercise training improves heart rate variability and muscle performance: a randomized controlled trial in coronary artery disease patients | European Journal of Physical & Rehabilitation Medicine. | 2015 | 51 | 3 | 281-9 | Wrong comparator - exercise v exercise |
| Caruso 2017 | Caruso, F. R.; Bonjorno, J. C., Jr.; Arena, R.; Phillips, S. A.; Cabiddu, R.; Mendes, R. G.; Arakelian, V. M.; Bassi, D.; Borghi-Silva, A. | Hemodynamic, Autonomic, Ventilatory, and Metabolic Alterations After Resistance Training in Patients With Coronary Artery Disease: A Randomized Controlled Trial | American Journal of Physical Medicine & Rehabilitation | 2017 | 96 | 4 | 226-235 | Wrong comparator - exercise v exercise |
| Casillas 2016 | Casillas, J. M.; Besson, D.; Hannequin, A.; Gremeaux, V.; Morisset, C.; Tordi, N.; Laurent, Y.; Laroche, D. | Effects of an eccentric training personalized by a low rate of perceived exertion on the maximal capacities in chronic heart failure: a randomized controlled trial | European Journal of Physical & Rehabilitation Medicine. | 2016 | 52 | 2 | 159-68 | Wrong comparator - exercise v exercise |
| Castel-Lacanal 2016 | Castel-Lacanal, E. | Combination of motor imagery exercises and brain stimulation tms type pas in patients after hemiplegic stroke (MIPAS) | Clinicaltrials.gov | 2016 |  |  |  | Not relevant |
| Castro-Conde 2021 | Castro-Conde, A.; Abeytua, M.; Arrarte Esteban, V. I.; Caravaca Perez, P.; Dalmau Gonzalez-Gallarza, R.; Garza Benito, F.; Hidalgo Urbano, R. J.; Torres Marques, J.; Vidal-Perez, R.; Nunez-Gil, I. J. | Feasibility and results of an intensive cardiac rehabilitation program. Insights from the MxM (Mas por Menos) randomized trial | Revista Espanola de Cardiologia | 2021 | 74 | 6 | 518-525 | Not relevant |
| Catapani 2021 | Catapani, L. B.; Dos Santos, T. P.; Toffano, G. C.; Souza, H. C. D.; de Araujo, J. E. | Aerobic Exercise After Left-Sided Stroke Improves Gait Speed and Endurance: A Prospective Cohort Study | American Journal of Physical Medicine & Rehabilitation | 2021 | 100 | 6 | 576-583 | Wrong study design - not RCT |
| Cattaneo 2019 | Cattaneo, D.; Gervasoni, E.; Pupillo, E.; Bianchi, E.; Aprile, I.; Imbimbo, I.; Russo, R.; Cruciani, A.; Turolla, A.; Jonsdottir, J.; Agostini, M.; Beghi, E.; Group, Neurofall | Educational and Exercise Intervention to Prevent Falls and Improve Participation in Subjects With Neurological Conditions: the NEUROFALL Randomized Controlled Trial | Frontiers in Neurology | 2019 | 10 |  | 865 | Wrong population - under 80% vascular |
| Cavalcante 2017 | Cavalcante, B. R.; Ritti-Dias, R. M.; Soares, A. H.; Lima, A. H.; Correia, M. A.; De Matos, L. D.; Gobbi, F.; Leicht, A. S.; Wolosker, N.; Cucato, G. G. | A Single Bout of Arm-crank Exercise Promotes Positive Emotions and Post-Exercise Hypotension in Patients with Symptomatic Peripheral ArteryÂ Disease | European Journal of Vascular and Endovascular Surgery | 2017 | 53 | 2 | 223-228 | Wrong intervention < 6 weeks |
| Cavallo 2020 | Cavallo, M.; Donato, D.; Casile, C.; Castagna, G.; Capomolla, S.; Daniele, M. | Outpatient management program of ischemic heart disease: comparison between structured outpatient control and usual care | European Heart Journal, Supplement | 2020 | 22 | SUPPL G | G183-G184 | Wrong comparator - co-interventions not balanced between arms |
| Cayton 2017 | Cayton, T.; Harwood, A. E.; Smith, G. E.; Totty, J. P.; Carradice, D.; Chetter, I. C. | Extracorporeal shockwave therapy for the treatment of lower limb intermittent claudication: study protocol for a randomised controlled trial (the SHOCKWAVE 1 trial) | Trials | 2017 | 18 | 1 | 104 | Not relevant |
| Celano 2018 | Celano, C. M.; Albanese, A. M.; Millstein, R. A.; Mastromauro, C. A.; Chung, W. J.; Campbell, K. A.; Legler, S. R.; Park, E. R.; Healy, B. C.; Collins, L. M.; Januzzi, J. L.; Huffman, J. C. | Optimizing a Positive Psychology Intervention to Promote Health Behaviors After an Acute Coronary Syndrome: The Positive Emotions After Acute Coronary Events III (PEACE-III) Randomized Factorial Trial | Psychosomatic Medicine | 2018 | 80 | 6 | 526-534 | Not relevant |
| Celano 2018 | Celano, C. M.; Beale, E. E.; Freedman, M.; Januzzi, J. L.; Huffman, J. C. | A positive psychology-based intervention to promote health behaviors in heart failure: the reach for health proof-ofconcept trial | Psychosomatic Medicine | 2018 | 80 | 3 | A36 | Not relevant |
| Celano 2020 | Celano, C. M.; Freedman, M.; Harnedy, L.; Huffman, J. C. | A Positive Psychology-Based Health Behavior Intervention in Heart Failure: results from the Reach for Health Pilot Trial | Psychosomatic Medicine | 2020 | 82 | 6 | A109 | Not relevant |
| Celano 2018 | Celano, C. M.; Freedman, M. E.; Beale, E. E.; Gomez-Bernal, F.; Huffman, J. C. | A Positive Psychology Intervention to Promote Health Behaviors in Heart Failure: a Proof-of-Concept Trial | Journal of nervous and mental Disease | 2018 | 206 | 10 | 800-808 | Not relevant |
| Center 2024 | Center, Beth Israel Deaconess Medical; Brigham,; Hospital, Women's; System, V. A. Boston Healthcare; Michigan, University of | Mindful Steps 2.0: Promoting physical activity in Patients With COPD and HF |  | 2024 |  |  |  | Wrong comparator - co-interventions not balanced between arms |
| Center 2019 | Lui X. | Time Window for Ischemic Stroke First Mobilization Effectiveness |  | 2019 |  |  |  | Not relevant |
| Center 2024 | Center, University of Kansas Medical | To Develop a Walking Exercise Program for Non-ambulatory Stroke Survivors |  | 2024 |  |  |  | Wrong comparator - exercise v exercise |
| Center 2024 | Center, University of Texas Southwestern Medical | Efficacy of LoDoCo in Improving Exercise Capacity Among Patients With HFpEF and Inflammation |  | 2024 |  |  |  | Not relevant |
| Center 2018 | Center, University of Texas Southwestern Medical; Se, Biotronik; Kg, Co; Medicine, American College of Sports; Association, American Heart | High Intensity Exercise for Increasing Fitness in Patients With Hypertrophic Cardiomyopathy |  | 2018 |  |  |  | Wrong population - under 80% vascular |
| Center 2020 | Center, Vanderbilt University Medical | Exercise Pulmonary Transit Time |  | 2020 |  |  |  | Not relevant |
| CentreHospitalierUniversitaire 2017 | Centre Hospitalier Universitaire, Amiens | Evaluation of physical activity Practice (AP) After Cerebral Vascular Stroke (Stroke) |  | 2017 |  |  |  | Not relevant |
| Cerrahpasa 2021 | Cerrahpasa, Istanbul University | Core Stabilization Exercises in Stroke |  | 2021 |  |  |  | Wrong intervention - does not meet exercise definition |
| Cerrahpasa 2021 | Cerrahpasa, Istanbul University | The Effects of Core Stabilization Exercises With Swisball in Stroke Patients |  | 2021 |  |  |  | Not relevant |
| Cha 2017 | Cha, H. G.; Kim, M. K. | Effects of strengthening exercise integrated repetitive transcranial magnetic stimulation on motor function recovery in subacute stroke patients: a randomized controlled trial | Technology and Health Care | 2017 | 25 | 3 | 521-529 | Not relevant |
| Cha 2017 | Cha, H. G.; Shin, Y. J.; Kim, M. K. | Effects of the Bad Ragaz Ring Method on muscle activation of the lower limbs and balance ability in chronic stroke: A randomised controlled trial | Hong Kong Physiotherapy Journal | 2017 | 37 |  | 39-45 | Wrong intervention - does not meet exercise definition |
| Cha 2018 | Cha, Y. J.; Kim, J. D.; Choi, Y. R.; Kim, N. H.; Son, S. M. | Effects of gait training with auditory feedback on walking and balancing ability in adults after hemiplegic stroke: a preliminary, randomized, controlled study | International Journal of Rehabilitation Research | 2018 | 41 | 3 | 239-243 | Not relevant |
| Chae 2020 | Chae, S. H.; Kim, Y.; Lee, K. S.; Park, H. S. | Development and Clinical Evaluation of a Web-Based Upper Limb Home Rehabilitation System Using a Smartwatch and Machine Learning Model for Chronic Stroke Survivors: Prospective Comparative Study | JMIR MHealth and UHealth | 2020 | 8 | 7 | e17216 | Not relevant |
| Chair 2024 | Chair, S. Y.; Cheng, H. Y.; Lo, S. W. S.; Sit, J. W. H.; Wong, E. M. L.; Leung, K. C.; Wang, Q.; Choi, K. C.; Leung, T. S. Y. | Effectiveness of a home-based music-paced physical activity programme on exercise-related outcomes after cardiac rehabilitation: a randomized controlled trial | European Journal of Cardiovascular Nursing. | 2024 | 2 |  |  | Not relevant |
| Chakraborty 2020 | Chakraborty, S.; Dey, T.; Mukherjee, A.; Alberts, J. L.; Linder, S. M. | Functional modeling of pedaling kinematics for the Stroke patients | Journal of Biopharmaceutical Statistics | 2020 | 30 | 4 | 674-688 | Not relevant |
| Chan 2017 | Chan, A. W.; Leung, D. Y.; Sit, J. W.; Chair, S. Y. | Tai Chi exercise reduces stroke risk factors: A randomized controlled trial | Stroke. Conference: American Heart Association/American Stroke Association | 2017 | 48 | 1 |  | Not relevant |
| Chan 2017 | Chan, K.; Phadke, C. P.; Stremler, D.; Suter, L.; Pauley, T.; Ismail, F.; Boulias, C. | The effect of water-based exercises on balance in persons post-stroke: a randomized controlled trial | Topics in Stroke Rehabilitation | 2017 | 24 | 4 | 228-235 | Wrong intervention - does not meet exercise definition |
| Chan 2022 | Chan, N. P. T.; Lai, A. Y. K.; Choy, H. K.; Cheung, D. Y. T.; Wan, A. N. T.; Cheng, V. Y. H.; Chan, K. Y.; Lau, Y. K.; Yung, C. Y.; Cheung, G. O. C.; Lam, T. H. | Feasibility and Potential Effectiveness of a Smartphone Zero-Time Exercise Intervention for Promoting physical activity and Fitness in Patients With Coronary Heart Disease: A Pilot Randomized Controlled Trial | Frontiers in Public Health | 2022 | 10 |  | 865712 | Not relevant |
| Chang 2021 | Chang, K. W.; Lin, C. M.; Yen, C. W.; Yang, C. C.; Tanaka, T.; Guo, L. Y. | The Effect of Walking Backward on a Treadmill on Balance, Speed of Walking and Cardiopulmonary Fitness for Patients with Chronic Stroke: A Pilot Study | International Journal of Environmental Research & Public Health [Electronic Resource] | 2021 | 18 | 5 | 1 | Wrong intervention < 6 weeks |
| Chang-Yong 2017 | Chang-Yong, Kim; Jung-Sun, Lee; Hyeong-Dong, Kim | Comparison of the Effect of Lateral and Backward Walking Training on Walking Function in Patients with Poststroke Hemiplegia...A Pilot Randomized Controlled Trial | American Journal of Physical Medicine & Rehabilitation | 2017 | 96 | 2 | 61-67 | Not relevant |
| Chaparro 2018 | Chaparro, D.; Daviet, J. C.; Borel, B.; Kammoun, B.; Salle, J. Y.; Tchalla, A.; Mandigout, S. | Home-based physical activity incentive and education program in subacute phase of stroke recovery (Ticaa'dom): study protocol for a randomized controlled trial | Trials | 2018 | 19 | 1 | 68 | Not relevant |
| ChaparroObando 2018 | Chaparro Obando, D. A.; Borel, B.; Salle, J. Y.; Compagnat, M.; Daviet, J. C.; Stephane, M. | Effects of home-based physical activity incitation and education program in subacute phase of stroke recovery after 6 months of monitoring | Annals of Physical and Rehabilitation Medicine | 2018 | (no pagination) |  |  | Not relevant |
| CharalambousCharalambos 2018 | Charalambous Charalambos, C.; Helm Erin, E.; Lau Kristin, A.; Morton Susanne, M.; Reisman Darcy, S. | The feasibility of an acute high-intensity exercise bout to promote locomotor learning after stroke | Topics in Stroke Rehabilitation | 2018 | 25 | 2 | 83-89 | Not relevant |
| CharlesUniversity 2022 | Charles University, Czech Republic; Hospital, Thomayer University; Republic, Academy of Science Czech; Ministry of Health, Czech Republic; Vascular surgery, University hospital Královské Vinohrady Prague | Comprehensive Intensive Stroke Rehabilitation |  | 2022 |  |  |  | Wrong intervention < 6 weeks |
| Chattopadhyay 2019 | Chattopadhyay, K.; Chandrasekaran, A. M.; Praveen, P. A.; Manchanda, S. C.; Madan, K.; Ajay, V. S.; Singh, K.; Tillin, T.; Hughes, A. D.; Chaturvedi, N.; Ebrahim, S.; Pocock, S.; Reddy, K. S.; Tandon, N.; Prabhakaran, D.; Kinra, S. | Development of a Yoga-Based Cardiac Rehabilitation (Yoga-CaRe) Programme for Secondary Prevention of Myocardial Infarction | Evidence-based Complementary and Alternative Medicine | 2019 | (no pagination) |  |  | Not relevant |
| Chaveles 2021 | Chaveles, I.; Papazachou, O.; al Shamari, M.; Delis, D.; Ntalianis, A.; Panagopoulou, N.; Nanas, S.; Karatzanos, E. | Effects of exercise training on diastolic and systolic dysfunction in patients with chronic heart failure | World Journal of Cardiology | 2021 | 13 | 9 | 514-525 | Wrong comparator - exercise v exercise |
| Chehuen 2017 | Chehuen, M.; Cucato, G. G.; Carvalho, C. R. F.; Ritti-Dias, R. M.; Wolosker, N.; Leicht, A. S.; Forjaz, C. L. M. | Walking training at the heart rate of pain threshold improves cardiovascular function and autonomic regulation in intermittent claudication: a randomized controlled trial | Journal of Science and Medicine in Sport | 2017 | 20 | 10 | 886-892 | Wrong comparator - active control |
| Chehuen 2021 | Chehuen, M. D. R.; Cucato, G. G.; Carvalho, C. R. F.; Zerati, A. E.; Leicht, A.; Wolosker, N.; Ritti-Dias, R. M.; Forjaz, C. L. M. | Walking Training Improves Ambulatory Blood Pressure Variability in Claudication | Arquivos Brasileiros de Cardiologia | 2021 | 116 | 5 | 898-905 | Full text not in English |
| Chen 2021 | Chen, J.; Gu, S.; Song, Y.; Ji, X.; Zeng, W.; Wang, X.; Wang, Y.; Feng, Q. | The impact of cardiomotor rehabilitation on endothelial function in elderly patients with chronic heart failure | BMC Cardiovascular Disorders | 2021 | 21 | 1 | 524 | Wrong comparator - active control |
| Chen 2020 | Chen, J.; Sun, D.; Zhang, S.; Shi, Y.; Qiao, F.; Zhou, Y.; Liu, J.; Ren, C. | Effects of home-based telerehabilitation in patients with stroke: A randomized controlled trial | Neurology | 2020 | 95 | 17 | e2318-e2330 | Not relevant |
| Chen 2016 | Chen, J. T.; Lin, T. H.; Voon, W. C.; Lai, W. T.; Huang, M. H.; Sheu, S. H.; Chen, C. K. | Beneficial effects of home-based cardiac rehabilitation on metabolic profiles in coronary heart-disease patients | Kaohsiung Journal of Medical Sciences | 2016 | 32 | 5 | 267-75 | Wrong comparator - co-interventions not balanced between arms |
| Chen 2022 | Chen, L.; Chen, Y.; Fu, W. B.; Huang, D. F.; Lo, W. L. A. | The Effect of Virtual Reality on Motor Anticipation and Hand Function in Patients with Subacute Stroke: A Randomized Trial on Movement-Related Potential | Neural Plasticity | 2022 | 2022 |  | 7399995 | Not relevant |
| Chen 2019 | Chen, L.; Wang, F.; Lv, L.; Zhang, Y.; Shen, X. | The efficacy of a patient-centered self-management empowerment intervention program (PCSMEI) for first-time stroke survivors: a randomized controlled trial | Stroke | 2019 | 50 |  |  | Not relevant |
| Chen 2017 | Chen, M.; Xue, L.; Cha, C.; Liang, J.; Ma, P. | Impact of strengthening Clinical management and intervention on prognosis and compliance among patients with heart failure | BioMedical Research (India) | 2017 | 28 | 13 | 5751-5757 | Not relevant |
| Chen 2020 | Chen, M. G.; Liang, X.; Kong, L.; Wang, J.; Wang, F.; Hu, X.; He, J.; Zeng, R. X.; Mao, S.; Guo, L.; Zhang, M. Z.; Zhang, X. | Effect of Baduanjin Sequential Therapy on the Quality of Life and Cardiac Function in Patients with AMI after PCI: a Randomized Controlled Trial | Evidence-based Complementary and Alternative Medicine | 2020 | 2020 |  |  | Wrong comparator - exercise v exercise |
| Chen 2015 | Chen, M. H.; Huang, L. L.; Lee, C. F.; Hsieh, C. L.; Lin, Y. C.; Liu, H.; Chen, M. I.; Lu, W. S. | A controlled pilot trial of two commercial video games for rehabilitation of arm function after stroke | Clinical Rehabilitation | 2015 | 29 | 7 | 674-82 | Wrong study design - not RCT |
| Chen 2021 | Chen, S.; Lv, C.; Wu, J.; Zhou, C.; Shui, X.; Wang, Y. | Effectiveness of a home-based exercise program among patients with lower limb spasticity post-stroke: A randomized controlled trial | Asian Nursing Research | 2021 | 15 | 1 | 1-7 | Wrong intervention - does not meet exercise definition |
| Chen 2022 | Chen, S. C.; Kang, J. H.; Peng, C. W.; Hsu, C. C.; Lin, Y. N.; Lai, C. H. | Adjustable Parameters and the Effectiveness of Adjunct Robot-Assisted Gait Training in Individuals with Chronic Stroke | International Journal of Environmental Research & Public Health [Electronic Resource] | 2022 | 19 | 13 | 4 | Not relevant |
| Chen 2018 | Chen, T. | Effects of martial arts on recovery of motor function and nerve excitability of stroke patients | NeuroQuantology | 2018 | 16 | 6 | 894-898 | Not relevant |
| Chen 2017 | Chen, T. Y.; Cheng, Y. C.; Huang, S. J.; Lin, C. H.; Hsia, K. Y.; Wang, C. L.; Chen, R. J. | Does task-oriented virtual reality training on chronic stroke patients decrease the resources utilization of physical therapy in Taiwan? | International Journal of Stroke | 2017 | 12 | 3 | 50-51 | Wrong intervention - does not meet exercise definition |
| Chen 2019 | Chen, W.; Ni, J.; Qiao, Z.; Wu, Y.; Lu, L.; Zheng, J.; Chen, R.; Lu, X. | Comparison of the Clinical outcomes of two physiological ischemic training methods in patients with coronary heart disease | Open Medicine (Poland) | 2019 | 14 | 1 | 224-233 | Wrong intervention - does not meet exercise definition |
| Chen 2020 | Chen, X.; Gan, Z.; Tian, W.; Lv, Y. | Effects of rehabilitation training of core muscle stability on stroke patients with hemiplegia | Pakistan Journal of Medical Sciences | 2020 | 36 | 3 | 461-466 | Not relevant |
| Chen 2018 | Chen, X.; Jiang, W.; Lin, X.; Lundborg, C. S.; Wen, Z.; Lu, W.; Marrone, G. | Effect of an exercise-based cardiac rehabilitation program "Baduanjin Eight-Silken-Movements with self-efficacy building" for heart failure (BESMILE-HF study): study protocol for a randomized controlled trial | Trials | 2018 | 19 | 1 | 150 | Wrong intervention - does not meet exercise definition |
| Chen 2021 | Chen, X.; Jiang, W.; Olson, T. P.; Lundborg, C. S.; Wen, Z.; Lu, W.; Marrone, G. | Feasibility and Preliminary Effects of the BESMILE-HF Program on Chronic Heart Failure Patients: A Pilot Randomized Controlled Trial | Frontiers in Cardiovascular Medicine | 2021 | 8 |  | 715207 | Wrong intervention - does not meet exercise definition |
| Chen 2019 | Chen, X.; Lu, W.; Wen, Z.; Marrone, G.; Jiang, W. | Feasibility and acceptability of an exercise-based cardiac rehabilitation program ?Baduanjin Eight- Silken-Movements with self-efficacy building for heart failure? (BESMILE-HF) | Advances in Integrative Medicine | 2019 | 6 |  | S88 | Wrong intervention - does not meet exercise definition |
| Chen 2019 | Chen, X.; Zhu, H.; Wen, Z.; Marrone, G.; Jiang, W.; Lu, W. | Are Traditional Forms of Exercise Feasible and Acceptable for Chronic Heart Failure Patients in China? A Mixed-Method Pilot Study | Journal of Cardiac Failure | 2019 | 25 | 8 | S101 | Wrong intervention - does not meet exercise definition |
| Chen 2023 | Chen, Y. C.; Chou, W.; Hong, R. B.; Lee, J. H.; Chang, J. H. | Home-based rehabilitation versus hospital-based rehabilitation for stroke patients in post-acute care stage: Comparison on the quality of life | Journal of the Formosan Medical Association | 2023 | 122 | 9 | 862-871 | Not relevant |
| Chen 2022 | Chen, Y. W.; Chiang, W. C.; Chang, C. L.; Lo, S. M.; Wu, C. Y. | Comparative effects of EMG-driven robot-assisted therapy versus task-oriented training on motor and daily function in patients with stroke: a randomized cross-over trial | Journal of NeuroEngineering and Rehabilitation | 2022 | 19 | 1 | 6 | Wrong intervention < 6 weeks |
| Cheng 2018 | Cheng, J. H.; Wang, Y. J.; Chou, S. S.; Yeh, M. L. | Chan-Chuang Qigong Improves Exercise Capacity, Depression, and Quality of Life in Patients With Heart Failure. [Chinese] | Hu li za zhi The Journal of Nursing | 2018 | 65 | 5 | 34-44 | Full text not in English |
| Cheng 2022 | Cheng, J. Y.; Yang, Y. R.; Yeh, N. C.; Cho, H.; Wang, V.; Li, J. C.; Wang, R. Y. | Effects of inclined treadmill training on inadequate ankle control during walking in individuals after stroke: A pilot randomized controlled trial | NeuroRehabilitation | 2022 | 51 | 1 | 171-180 | Wrong intervention < 6 weeks |
| Cherian 2019 | Cherian, B.; Jackson, C. | Evaluation of a group based exercise programme for community dwelling stroke survivors...The Chartered Society of Physiotherapy UK Conference 2018, Birmingham, UK, 19-20 October 2018 | Physiotherapy | 2019 | 105 |  | e117-e117 | Not relevant |
| Chi 2016 | Chi, Ctr Ior | Comparision of effectiveness of different types of gait training with high intensity in patients with hemipledge: multi-directional pertubation-basd and maximum speed-tolerated gait training | https://trialsearch.who.int/Trial2.aspx?TrialID=ChiCTR-IOR-16009536 | 2016 |  |  |  | Wrong intervention < 6 weeks |
| Chi 2016 | Chi, Ctr Inr | The effect of Baduanjin exercise on cognitive function in patients with post-stroke cognitive impairment: a randomized controlled trial | https://trialsearch.who.int/Trial2.aspx?TrialID=ChiCTR-INR-16009364 | 2016 |  |  |  | Wrong intervention - does not meet exercise definition |
| Chi 2016 | Chi, Ctr Inr | SMARTphone-based Home Cardiac Rehabilitation and Secondary Prevention in Chinese Coronary Heart Disease Patients (SMART-CR/SP): a randomized controlled trial | https://trialsearch.who.int/Trial2.aspx?TrialID=ChiCTR-INR-16009598 | 2016 |  |  |  | Wrong comparator - co-interventions not balanced between arms |
| Chi 2017 | Chi, Ctr Ior | Acupuncture on the motor area of the scalp combined with exercise therapy simultaneously for motor dysfunction in patients with cerebral infarction | https://trialsearch.who.int/Trial2.aspx?TrialID=ChiCTR-IOR-17012232 | 2017 |  |  |  | Not relevant |
| Chi 2017 | Chi, Ctr Ior | The effect of Baduanjin exercise on cardiac rehabilitation in patients with stable coronary artery disease: a randomized controlled trial | https://trialsearch.who.int/Trial2.aspx?TrialID=ChiCTR-IOR-17014149 | 2017 |  |  |  | Wrong intervention < 6 weeks |
| Chi 2017 | Chi, Ctr Ior | The effect of comprehensive rehabilitation on patients with coronary heart disease and stroke | https://trialsearch.who.int/Trial2.aspx?TrialID=ChiCTR-IOR-17011721 | 2017 |  |  |  | Wrong comparator - exercise v exercise |
| Chi 2017 | Chi, Ctr Iic | Effects of a Group- plus Home-based Tai Chi Program on Improving physical Function and Psychosocial Well-being in Patients with Coronary Heart Disease: a Pilot Randomized Controlled Trial | https://trialsearch.who.int/Trial2.aspx?TrialID=ChiCTR-IIC-17013986 | 2017 |  |  |  | Not relevant |
| Chi 2017 | Chi, Ctr Ior | Effects of Baduanjin Exercise Intervention on Limb Contracture among Cerebral Infarction Patients with Hemiplegia | https://trialsearch.who.int/Trial2.aspx?TrialID=ChiCTR-IOR-17012447 | 2017 |  |  |  | Not relevant |
| Chi 2017 | Chi, Ctr Ipr | The effects of I stage cardiac rehabilitation on cardiopulmonary function in patients undergoing open heart surgery: a randomized controlled study | https://trialsearch.who.int/Trial2.aspx?TrialID=ChiCTR-IPR-17011445 | 2017 |  |  |  | Not relevant |
| Chi 2017 | Chi, Ctr Ior | Music-paced physical activity intervention for post-cardiac rehabilitation patients with coronary heart disease | https://trialsearch.who.int/Trial2.aspx?TrialID=ChiCTR-IOR-17011015 | 2017 |  |  |  | Not relevant |
| Chi 2017 | Chi, Ctr Ipr | A randomized controlled trial to assess feasibility of aerobic exercise-based cardiac rehabilitation on patients with coronary heart disease | https://trialsearch.who.int/Trial2.aspx?TrialID=ChiCTR-IPR-17010556 | 2017 |  |  |  | Wrong comparator - exercise v exercise |
| Chi 2017 | Chi, Ctr Rnr | An Exploratory Clinical Study on Effect of Home-based Cardiac Exercise Rehabilitation with Remote Electrocardiogram Monitoring in Patients with Chronic Heart Failure | https://trialsearch.who.int/Trial2.aspx?TrialID=ChiCTR-RNR-17012446 | 2017 |  |  |  | Not relevant |
| Chia 2015 | Chia, N.; Ambrosini, E.; Baccinelli, W.; Nardone, A.; Monticone, M.; Ferrigno, G.; Pedrocchi, A.; Ferrante, S. | A multi-channel biomimetic neuroprosthesis to support treadmill gait training in stroke patients | Annual International Conference of the IEEE Engineering in Medicine and Biology Society. | 2015 |  |  | 7159-7162 | Not relevant |
| Chicago 2019 | Chicago, University of Illinois at | Alternative Therapies for Improving physical Function in Individuals With Stroke |  | 2019 |  |  |  | Not relevant |
| ChiCtr 2018 | ChiCtr | The diagnosis and intervenen strategy study for heart failure with preserved ejection fraction based on cardio-pulmonary exercise test | https://trialsearch.who.int/Trial2.aspx?TrialID=ChiCTR1800019183 | 2018 |  |  |  | Wrong comparator - co-interventions not balanced between arms |
| ChiCtr 2018 | ChiCtr | Effect of different intensity rehabilitation training on hemiplegic patients after stroke | https://trialsearch.who.int/Trial2.aspx?TrialID=ChiCTR1800016841 | 2018 |  |  |  | Wrong comparator - active control |
| ChiCtr 2018 | ChiCtr | Effect of enhanced external counterpulsation (EECP) on cardiac rehabilitation treatment in patients with stable angina pectoris | https://trialsearch.who.int/Trial2.aspx?TrialID=ChiCTR1800020102 | 2018 |  |  |  | Not relevant |
| ChiCtr 2018 | ChiCtr | Effectiveness of different type of Baduanjin excercise on I and II phase cardiac rehabilitation for acute myocardial infarction: a randomized controlled trail (BECHAMI) | https://trialsearch.who.int/Trial2.aspx?TrialID=ChiCTR1800016209 | 2018 |  |  |  | Wrong comparator - exercise v exercise |
| ChiCtr 2018 | ChiCtr | The effectiveness of eHealth cardiac rehabilitation on health outcomes of Chinese patients with coronary artery disease | https://trialsearch.who.int/Trial2.aspx?TrialID=ChiCTR1800020411 | 2018 |  |  |  | Not relevant |
| ChiCtr 2018 | ChiCtr | Effects of Wearable-Device-Monitored Home-Based Cardiac Rehabilitation on cardiopulmonary function and outcomes of patients after percutaneous coronary intervention: a single-centered prospective study | https://trialsearch.who.int/Trial2.aspx?TrialID=ChiCTR1800015042 | 2018 |  |  |  | Wrong study design - not RCT |
| ChiCtr 2018 | ChiCtr | Exploring Clinical efficacy evaluation of psychological and physical rehabilitation on post-stroke patients: a Zelen's design study | https://trialsearch.who.int/Trial2.aspx?TrialID=ChiCTR1800017752 | 2018 |  |  |  | Not relevant |
| ChiCtr 2018 | ChiCtr | The Feasibility and Effects of Smartphone-based Application on Cardiac Rehabilitation for Percutaneous Coronary Intervention Patients in Macau | https://trialsearch.who.int/Trial2.aspx?TrialID=ChiCTR1800014521 | 2018 |  |  |  | Wrong comparator - exercise v exercise |
| ChiCtr 2018 | ChiCtr | The multiple modal tasks on gait control and motor cognition after stroke | https://trialsearch.who.int/Trial2.aspx?TrialID=ChiCTR1800017487 | 2018 |  |  |  | Not relevant |
| ChiCtr 2018 | ChiCtr | The recovery mechanism and effect of AlterG anti-gravity treadmill training to lower limb function in patients with stroke | https://trialsearch.who.int/Trial2.aspx?TrialID=ChiCTR1800020253 | 2018 |  |  |  | Wrong comparator - exercise v exercise |
| ChiCtr 2019 | ChiCtr | Application of TCM features comprehensive rehabilitation program in chronic heart failure treatment | http://www.who.int/trialsearch/Trial2.aspx?TrialID=ChiCTR1900026779 | 2019 |  |  |  | Not relevant |
| ChiCtr 2019 | ChiCtr | Clinical study for heart failure management | http://www.who.int/trialsearch/Trial2.aspx?TrialID=ChiCTR1900022559 | 2019 |  |  |  | Not relevant |
| ChiCtr 2019 | ChiCtr | Clinical validation of SE-NaturalGait in Clinical application | http://www.who.int/trialsearch/Trial2.aspx?TrialID=ChiCTR1900026792 | 2019 |  |  |  | Not relevant |
| ChiCtr 2019 | ChiCtr | Effect of Aquatic Strength Training on Postural Stability and Walking Function in Stroke Patients: a Randomised Trial | https://trialsearch.who.int/Trial2.aspx?TrialID=ChiCTR1900021097 | 2019 |  |  |  | Wrong comparator - exercise v exercise |
| ChiCtr 2019 | ChiCtr | Effect of bilateral knee training on walking ability of stroke patients with hemiplegia | http://www.who.int/trialsearch/Trial2.aspx?TrialID=ChiCTR1900026433 | 2019 |  |  |  | Not relevant |
| ChiCtr 2019 | ChiCtr | Effect of gait assessment and training system based on visual feedback mode on walking function of stroke patients | http://www.who.int/trialsearch/Trial2.aspx?TrialID=ChiCTR1900024253 | 2019 |  |  |  | Not relevant |
| ChiCtr 2019 | ChiCtr | Effect of lower-body positive pressure treadmills on rebuilding and dynamic biomechanical mechanism of lower limbs motor function after stroke | http://www.who.int/trialsearch/Trial2.aspx?TrialID=ChiCTR1900023800 | 2019 |  |  |  | Not relevant |
| ChiCtr 2019 | ChiCtr | Effect of multimodal walking training on walking function of stroke patients | http://www.who.int/trialsearch/Trial2.aspx?TrialID=ChiCTR1900026187 | 2019 |  |  |  | Wrong intervention - does not meet exercise definition |
| ChiCtr 2019 | ChiCtr | Effect of Sensory Infusion Therapy Based on Exercise Program on Recovery Stroke Patients | http://www.who.int/trialsearch/Trial2.aspx?TrialID=ChiCTR1900024495 | 2019 |  |  |  | Not relevant |
| ChiCtr 2019 | ChiCtr | The effect of the optimal-lymph-flow exercise on fluid overload symptoms among patients with heart failure | http://www.who.int/trialsearch/Trial2.aspx?TrialID=ChiCTR1900024499 | 2019 |  |  |  | Wrong intervention - does not meet exercise definition |
| ChiCtr 2019 | ChiCtr | Effect of voluntary breathing exercise on heart rate variability and heart rate systolic blood pressure product in patients with stable coronary heart disease | http://www.who.int/trialsearch/Trial2.aspx?TrialID=ChiCTR1900024043 | 2019 |  |  |  | Not relevant |
| ChiCtr 2019 | ChiCtr | An effective analysis of exercise therapy based on muscle chain theory in improving walking ability of stroke patients | http://www.who.int/trialsearch/Trial2.aspx?TrialID=ChiCTR1900025791 | 2019 |  |  |  | Wrong intervention - does not meet exercise definition |
| ChiCtr 2019 | ChiCtr | Effects of dual task walking training on motor and cognitive function in stroke patients: a randomized controlled trial | https://trialsearch.who.int/Trial2.aspx?TrialID=ChiCTR1900026598 | 2019 |  |  |  | Not relevant |
| ChiCtr 2019 | ChiCtr | The mechanism and effect of Pro-kin system training on Static and dynamic balance | https://trialsearch.who.int/Trial2.aspx?TrialID=ChiCTR1900021291 | 2019 |  |  |  | Wrong intervention - does not meet exercise definition |
| ChiCtr 2019 | ChiCtr | Rehabilitation and mechanism study for post-stroke dyskinesia with high-intensity interval training (HIIT) | http://www.who.int/trialsearch/Trial2.aspx?TrialID=ChiCTR1900022892 | 2019 |  |  |  | Wrong comparator - exercise v exercise |
| ChiCtr 2019 | ChiCtr | Therapeutic Effect of AiWalker on Balance and Walking Ability of Patients with Stroke | http://www.who.int/trialsearch/Trial2.aspx?TrialID=ChiCTR1900026535 | 2019 |  |  |  | Not relevant |
| ChiCtr 2019 | ChiCtr | The effect of kashi resistance lumbar and dorsal muscle trainer and freehand therapy on poststroke orthostatic balance: a randomized controlled trial |  | 2019 |  |  |  | Wrong intervention - does not meet exercise definition |
| ChiCtr 2019 | ChiCtr | Effect of mind-body intervention Liuzijue for patients with chronic heart failure: a randomized controlled trial |  | 2019 |  |  |  | Wrong comparator - active control |
| ChiCtr 2020 | ChiCtr | 24-posture Tai Chi improves pulmonary functions of patients undergoing coronary artery bypass grafting: a randomized single-blind controlled Clinical trial | https://trialsearch.who.int/Trial2.aspx?TrialID=ChiCTR2000030026 | 2020 |  |  |  | Not relevant |
| ChiCtr 2020 | ChiCtr | Baduanjin exercise and health maintenance was applied to the Clinical study of chronic heart failure patients with coronary heart disease | http://www.who.int/trialsearch/Trial2.aspx?TrialID=ChiCTR2000037719 | 2020 |  |  |  | Wrong comparator - co-interventions not balanced between arms |
| ChiCtr 2020 | ChiCtr | Clinical study on the effect of tai chi ball exercise combined with eight-section brocade tonal method on postoperative rehabilitation PCI patients with acute myocardial infarction | http://www.who.int/trialsearch/Trial2.aspx?TrialID=ChiCTR2000034078 | 2020 |  |  |  | Wrong intervention - does not meet exercise definition |
| ChiCtr 2020 | ChiCtr | Effect and mechanism of cognitive-motor dual task training on vascular cognitive impairment after ischemic stroke | http://www.who.int/trialsearch/Trial2.aspx?TrialID=ChiCTR2000034862 | 2020 |  |  |  | Not relevant |
| ChiCtr 2020 | ChiCtr | The effect of caregiver-mediated exercises in-hospital rehabilitation on limb function of patients with hemiplegia after stroke | http://www.who.int/trialsearch/Trial2.aspx?TrialID=ChiCTR2000032705 | 2020 |  |  |  | Not relevant |
| ChiCtr 2020 | ChiCtr | Effect of Taiji Cloud Hand Visual Feedback Training on Balance Function of Stroke Patients with Subacute Hemiplegia | http://www.who.int/trialsearch/Trial2.aspx?TrialID=ChiCTR2000040083 | 2020 |  |  |  | Not relevant |
| ChiCtr 2020 | ChiCtr | Effects of Tai Chi Yunshou exercises on upper-limb proprioception and hand-eye coordination among post-stroke patients with hemiplegia | http://www.who.int/trialsearch/Trial2.aspx?TrialID=ChiCTR2000038553 | 2020 |  |  |  | Wrong intervention - does not meet exercise definition |
| ChiCtr 2020 | ChiCtr | Evaluation of Eight-Style Taijiquan on cognitive function in patients with cognitive impairment of cerebral small vessel disease | http://www.who.int/trialsearch/Trial2.aspx?TrialID=ChiCTR2000033176 | 2020 |  |  |  | Not relevant |
| ChiCtr 2020 | ChiCtr | The full cycle of cardiac rehabilitation in aged people with Coronary heart disease | http://www.who.int/trialsearch/Trial2.aspx?TrialID=ChiCTR2000040509 | 2020 |  |  |  | Wrong comparator - exercise v exercise |
| ChiCtr 2020 | ChiCtr | Rehabilitative Strategy of Remote Ischemic Conditioning with Exercise (RICE) in Patients with Acute Ischemic Stroke: a Randomized Controlled Study | https://trialsearch.who.int/Trial2.aspx?TrialID=ChiCTR2000041042 | 2020 |  |  |  | Not relevant |
| ChiCtr 2020 | ChiCtr | The study effect of 6-minute walking method to guide exercise prescription of patients with coronary heart disease in community | http://www.who.int/trialsearch/Trial2.aspx?TrialID=ChiCTR2000037836 | 2020 |  |  |  | Wrong comparator - exercise v exercise |
| ChiCtr 2020 | ChiCtr | Study on Exercise Rehabilitation Intervention in Chronic Stroke Patients with Hemiplegia | http://www.who.int/trialsearch/Trial2.aspx?TrialID=ChiCTR2000041335 | 2020 |  |  |  | Wrong intervention - does not meet exercise definition |
| ChiCtr 2020 | ChiCtr | Study on non-exercise test regression equation for predicting anaerobic threshold oxygen uptake and peak oxygen uptake in patients with coronary heart disease | http://www.who.int/trialsearch/Trial2.aspx?TrialID=ChiCTR2000037835 | 2020 |  |  |  | Wrong comparator - exercise v exercise |
| ChiCtr 2020 | ChiCtr | Study on the Clinical effect and mechanism of Taijiquan training on patients with silent brain infarction | http://www.who.int/trialsearch/Trial2.aspx?TrialID=ChiCTR2000040748 | 2020 |  |  |  | Wrong population - under 80% vascular |
| ChiCtr 2020 | ChiCtr | Study on the effect and mechanism of Taijiquan in improving cardiopulmonary function in convalescent stroke patients | http://www.who.int/trialsearch/Trial2.aspx?TrialID=ChiCTR2000034719 | 2020 |  |  |  | Wrong comparator - exercise v exercise |
| ChiCtr 2020 | ChiCtr | Study on the Effect and Mechanism of Thi Chi for Motor Dysfunction in Different Stages of Stroke: a Multicentre Randomized Controlled Trial | https://trialsearch.who.int/Trial2.aspx?TrialID=ChiCTR2000033417 | 2020 |  |  |  | Wrong comparator - exercise v exercise |
| ChiCtr 2020 | ChiCtr | Taiji postural training on rehabilitation of dyskinesia in convalescence of acute ischemic stroke: a Multi-center Randomized Controlled Trial | https://trialsearch.who.int/Trial2.aspx?TrialID=ChiCTR2000032999 | 2020 |  |  |  | Not relevant |
| ChiCtr 2020 | ChiCtr | To explore the mechanism of knee hyperextension in stroke patients at central and peripheral levels and the effectiveness of retrogression intervention | http://www.who.int/trialsearch/Trial2.aspx?TrialID=ChiCTR2000032295 | 2020 |  |  |  | Not relevant |
| ChiCtr 2021 | ChiCtr | Application of the movement characteristics of 'taking the waist as the axis' of Tai Chi on trunk posture control disorder in the convalescent stage of stroke | http://www.who.int/trialsearch/Trial2.aspx?TrialID=ChiCTR2100043760 | 2021 |  |  |  | Not relevant |
| ChiCtr 2021 | ChiCtr | Clinical multi-center study of Wuqinxi intervention on respiratory function of elderly stroke patients | https://trialsearch.who.int/Trial2.aspx?TrialID=ChiCTR2100052771 | 2021 |  |  |  | Not relevant |
| ChiCtr 2021 | ChiCtr | Clinical study on accurate assessment of volume load in patients with heart failure by MC-780MA body composition analyzer | https://trialsearch.who.int/Trial2.aspx?TrialID=ChiCTR2100047564 | 2021 |  |  |  | Not relevant |
| ChiCtr 2021 | ChiCtr | A Clinical study on ankle proprioception and balance function in stroke patients | https://trialsearch.who.int/Trial2.aspx?TrialID=ChiCTR2100054720 | 2021 |  |  |  | Wrong intervention - does not meet exercise definition |
| ChiCtr 2021 | ChiCtr | Clinical study on the effect of abdominal breathing combined with draw-in training on walking function in patients with early- to moderate-stage stroke | https://trialsearch.who.int/Trial2.aspx?TrialID=ChiCTR2100054897 | 2021 |  |  |  | Not relevant |
| ChiCtr 2021 | ChiCtr | A Clinical study on the effect of PNF technology and lower limb exoskeleton rehabilitation robot on lower limb motor function and walking ability after stroke | http://www.who.int/trialsearch/Trial2.aspx?TrialID=ChiCTR2100043541 | 2021 |  |  |  | Wrong intervention - does not meet exercise definition |
| ChiCtr 2021 | ChiCtr | Effect of different early bedside rehabilitation information intervention on elderly patients with different TYPES of ST-segment myocardial infarction complicated with diabetes | https://trialsearch.who.int/Trial2.aspx?TrialID=ChiCTR2100044827 | 2021 |  |  |  | Not relevant |
| ChiCtr 2021 | ChiCtr | Effect of healthy extremity strength training on fatigue and limb function recovery after stroke | https://trialsearch.who.int/Trial2.aspx?TrialID=ChiCTR2100049658 | 2021 |  |  |  | Wrong intervention - does not meet exercise definition |
| ChiCtr 2021 | ChiCtr | Effect of lower limb rehabilitation robot combined with 3D gait training system on reconstruction of lower limb motor function in patients with sequelae of stroke | https://trialsearch.who.int/Trial2.aspx?TrialID=ChiCTR2100053916 | 2021 |  |  |  | Not relevant |
| ChiCtr 2021 | ChiCtr | Effect of walking exercise combined with active breathing and circulation technique on activity tolerance in patients with chronic heart failure | https://trialsearch.who.int/Trial2.aspx?TrialID=ChiCTR2100053570 | 2021 |  |  |  | Not relevant |
| ChiCtr 2021 | ChiCtr | Effects of 60% AOP combined with low intensity resistance exercise on lower extremity function and neuroelectrophysiology in stroke patients | http://www.who.int/trialsearch/Trial2.aspx?TrialID=ChiCTR2100044004 | 2021 |  |  |  | Not relevant |
| ChiCtr 2021 | ChiCtr | Effects of AR combined with VR gait adaptability training on balance and walking ability of stroke patients with hemiplegia | http://www.who.int/trialsearch/Trial2.aspx?TrialID=ChiCTR2100042736 | 2021 |  |  |  | Wrong intervention - does not meet exercise definition |
| ChiCtr 2021 | ChiCtr | Effects of different brisk walking intensities on adherence and cardiorespiratory endurance of cardiac rehabilitation programmes among CHD patients after PCI | https://trialsearch.who.int/Trial2.aspx?TrialID=ChiCTR2100047568 | 2021 |  |  |  | Wrong comparator - exercise v exercise |
| ChiCtr 2021 | ChiCtr | Effects of different intensity resistance training on walking ability and cardiopulmonary function in stroke patients | https://trialsearch.who.int/Trial2.aspx?TrialID=ChiCTR2100050299 | 2021 |  |  |  | Wrong comparator - exercise v exercise |
| ChiCtr 2021 | ChiCtr | Efficacy evaluation of improving cardiopulmonary exercise tolerance of stable coronary heart disease by activating blood circulation and removing blood stasis | https://trialsearch.who.int/Trial2.aspx?TrialID=ChiCTR2100042591 | 2021 |  |  |  | Not relevant |
| ChiCtr 2021 | ChiCtr | Evaluation of Therapeutic function of Kickstart Lower limb Rehabilitation exercise device | http://www.who.int/trialsearch/Trial2.aspx?TrialID=ChiCTR2100046521 | 2021 |  |  |  | Not relevant |
| ChiCtr 2021 | ChiCtr | Image and correlation study of three-dimensional gait kinematics and diffusion tensor imaging in patients with cerebral small vessel disease by the rehabilitation training mode of combined intervention of visual feedback balance system and digital treadmill | https://trialsearch.who.int/Trial2.aspx?TrialID=ChiCTR2100050084 | 2021 |  |  |  | Not relevant |
| ChiCtr 2021 | ChiCtr | A prospective, multicenter, randomized controlled, non inferiority trial to evaluate the effectiveness and safety of the lower extremity exoskeleton | https://trialsearch.who.int/Trial2.aspx?TrialID=ChiCTR2100044475 | 2021 |  |  |  | Not relevant |
| ChiCtr 2021 | ChiCtr | Study on the effect of Taichi combined with rTMS on poststroke cognitive impairment and its neural mechanism based on multimodal magnetic resonance imaging | https://trialsearch.who.int/Trial2.aspx?TrialID=ChiCTR2100050239 | 2021 |  |  |  | Not relevant |
| ChiCtr 2021 | ChiCtr | Study on the effect of vigorous walking on patients with coronary heart disease after elective intervention | http://www.who.int/trialsearch/Trial2.aspx?TrialID=ChiCTR2100042918 | 2021 |  |  |  | Wrong comparator - active control |
| ChiCtr 2021 | ChiCtr | Traditional exercise techniques and stroke | https://trialsearch.who.int/Trial2.aspx?TrialID=ChiCTR2100048031 | 2021 |  |  |  | Wrong intervention - does not meet exercise definition |
| ChiCtr 2021 | ChiCtr | Effects of low-intensity resistance training with or without blood flow restriction on serum BDNF, VEGF in patients with post-stroke depression |  | 2021 |  |  |  | Wrong comparator - exercise v exercise |
| ChiCtr 2021 | ChiCtr | Changes of intestinal flora and metabolites of short-chain fatty acids in stroke patients and the effect of rehabilitation intervention |  | 2021 |  |  |  | Wrong study design - not RCT |
| ChiCtr 2021 | ChiCtr | Study on the effect of hypoxic cardiopulmonary exercise test on the evaluation of cardiac function in stroke patients |  | 2021 |  |  |  | Not relevant |
| ChiCtr 2022 | ChiCtr | Application of strengthening the | https://trialsearch.who.int/Trial2.aspx?TrialID=ChiCTR2200062329 | 2022 |  |  |  | Wrong intervention - does not meet exercise definition |
| ChiCtr 2022 | ChiCtr | biofeedback physical regulation of post-ischemic stroke with spastic hypertonia based on acupoints: a Clinical trial | https://trialsearch.who.int/Trial2.aspx?TrialID=ChiCTR2200067186 | 2022 |  |  |  | Not relevant |
| ChiCtr 2022 | ChiCtr | Clinical study on the effect of modified Tai Chi skill on balance and lower limb motor dysfunction in convalescent patients with cerebral infarction | https://trialsearch.who.int/Trial2.aspx?TrialID=ChiCTR2200063440 | 2022 |  |  |  | Wrong intervention - does not meet exercise definition |
| ChiCtr 2022 | ChiCtr | Design of Study protocol for A Randomized Clinical Trial | https://trialsearch.who.int/Trial2.aspx?TrialID=ChiCTR2200061757 | 2022 |  |  |  | Not relevant |
| ChiCtr 2022 | ChiCtr | Effect of gravity shifting training with NeuroCom Smart Balance Master balance training system on the walking ability of stroke patients | https://trialsearch.who.int/Trial2.aspx?TrialID=ChiCTR2200055694 | 2022 |  |  |  | Not relevant |
| ChiCtr 2022 | ChiCtr | Effect of intensive out-of-bed mobilization on the motion outcomes of patients with acute stroke | https://trialsearch.who.int/Trial2.aspx?TrialID=ChiCTR2200057404 | 2022 |  |  |  | Wrong intervention < 6 weeks |
| ChiCtr 2022 | ChiCtr | Effect of intramuscular patch combined with weight-loss walking training on walking function in stroke patients | https://trialsearch.who.int/Trial2.aspx?TrialID=ChiCTR2200064714 | 2022 |  |  |  | Wrong comparator - co-interventions not balanced between arms |
| ChiCtr 2022 | ChiCtr | Effect of moderate intensity aerobic exercise on cognition in stroke patients | https://trialsearch.who.int/Trial2.aspx?TrialID=ChiCTR2200062833 | 2022 |  |  |  | Wrong comparator - exercise v exercise |
| ChiCtr 2022 | ChiCtr | Effect of symmetrical walking training with antigravity treadmill on walking function in subacute stroke patients | https://trialsearch.who.int/Trial2.aspx?TrialID=ChiCTR2200064433 | 2022 |  |  |  | Wrong intervention - does not meet exercise definition |
| ChiCtr 2022 | ChiCtr | Effect of TCM cardiac rehabilitation program-liuzijue breathing method on cardiac function in patients with atrial fibrillation and chronic heart failure | https://trialsearch.who.int/Trial2.aspx?TrialID=ChiCTR2200066173 | 2022 |  |  |  | Not relevant |
| ChiCtr 2022 | ChiCtr | Effect of traditional exercise therapy on stroke patients | https://trialsearch.who.int/Trial2.aspx?TrialID=ChiCTR2200055619 | 2022 |  |  |  | Wrong intervention - does not meet exercise definition |
| ChiCtr 2022 | ChiCtr | Exercise rehabilitation study of patients with angina pectoris after PCI under the guidance of radionuclide myocardial perfusion: a randomized controlled trial | https://trialsearch.who.int/Trial2.aspx?TrialID=ChiCTR2200065131 | 2022 |  |  |  | Wrong comparator - exercise v exercise |
| ChiCtr 2022 | ChiCtr | Feasibility and effectiveness of digital therapy in full cycle rehabilitation of patients with heart failure | https://trialsearch.who.int/Trial2.aspx?TrialID=ChiCTR2200056020 | 2022 |  |  |  | Wrong comparator - exercise v exercise |
| ChiCtr 2022 | ChiCtr | Impact of resistance exercise rehabilitation and whey protein supplementation in elderly patients with heart failure with preserved ejection fraction and sarcopenia: a study protocol for a prospective randomized controlled trial | https://trialsearch.who.int/Trial2.aspx?TrialID=ChiCTR2200061069 | 2022 |  |  |  | Wrong comparator - exercise v exercise |
| ChiCtr 2022 | ChiCtr | Influence on Occupational Performance of Home-based Stroke Patients by Coaching-based Tele-occupational Guidance | https://trialsearch.who.int/Trial2.aspx?TrialID=ChiCTR2200061107 | 2022 |  |  |  | Not relevant |
| ChiCtr 2022 | ChiCtr | The interaction mechanism between the mechanical changes of spastic muscle and motor dysfunction at extremity of stroke survivors | https://trialsearch.who.int/Trial2.aspx?TrialID=ChiCTR2200055293 | 2022 |  |  |  | Not relevant |
| ChiCtr 2022 | ChiCtr | Research on the construction and application of exercise engagement and adherence intervention program for patients with heart failure based on PHE model | https://trialsearch.who.int/Trial2.aspx?TrialID=ChiCTR2200056688 | 2022 |  |  |  | Not relevant |
| ChiCtr 2022 | ChiCtr | Study on neurobiomechanical mechanism of Taijiquan in improving upper limb motor strategy in stroke patients | https://trialsearch.who.int/Trial2.aspx?TrialID=ChiCTR2200061376 | 2022 |  |  |  | Wrong intervention < 6 weeks |
| ChiCtr 2022 | ChiCtr | Study on the difference of sEMG delivery in key muscles of stroke patients after the intervention of virtual reality and upper limb robotic training | https://trialsearch.who.int/Trial2.aspx?TrialID=ChiCTR2200063546 | 2022 |  |  |  | Not relevant |
| ChiCtr 2022 | ChiCtr | A study on the driving mechanism and intervention of rehabilitation in young and middle-aged patients with hemorrhagic stroke | https://trialsearch.who.int/Trial2.aspx?TrialID=ChiCTR2200066498 | 2022 |  |  |  | Wrong population - under 80% vascular |
| ChiCtr 2022 | ChiCtr | The study on the nerve mechanism of lower limb rehabilitation robot-assisted walking training in improving walking dysfunction after stroke | https://trialsearch.who.int/Trial2.aspx?TrialID=ChiCTR2200060668 | 2022 |  |  |  | Not relevant |
| ChiCtr 2022 | ChiCtr | The Synergistic Effect and Mechanism of Aerobic Exercise Combined with Cognitive Training in Improving Cognitive Impairment of Subjects with Cerebrovascular Disease | https://trialsearch.who.int/Trial2.aspx?TrialID=ChiCTR2200066899 | 2022 |  |  |  | Wrong population - under 80% vascular |
| ChiCtr 2022 | ChiCtr | Transcranial direct current stimulation combined with body weight support-Tai Chi footwork for motor function of stroke survivors: a study protocol of randomized controlled trial | https://trialsearch.who.int/Trial2.aspx?TrialID=ChiCTR2200059329 | 2022 |  |  |  | Wrong intervention - does not meet exercise definition |
| ChiCtr 2022 | ChiCtr | Effect of high-intensity interval training on cardiopulmonary function in bedridden patients with stroke: a randomized controlled study |  | 2022 |  |  |  | Wrong comparator - exercise v exercise |
| ChiCtr 2022 | ChiCtr | Efficacy of Flexi-bar Exercise Shaking by Healthy Side on Trunk Muscles after Stroke |  | 2022 |  |  |  | Not relevant |
| ChiCtr 2022 | ChiCtr | Effects of aerobic training on prognosis of function (cognition, emotion, sleep, exercise) in stroke patients |  | 2022 |  |  |  | Wrong study design - not RCT |
| ChiCtr 2023 | ChiCtr | Accurate assessment and analysis of the effect of modified badanjin on balance function in stroke hemiplegia patients | https://trialsearch.who.int/Trial2.aspx?TrialID=ChiCTR2300069394 | 2023 |  |  |  | Wrong intervention - does not meet exercise definition |
| ChiCtr 2023 | ChiCtr | Application and mechanism of foot-ankle brain-computer interface training based on motor imagery after stroke | https://trialsearch.who.int/Trial2.aspx?TrialID=ChiCTR2300074381 | 2023 |  |  |  | Not relevant |
| ChiCtr 2023 | ChiCtr | Application of rehabilitation guidance on remote platform in patients with stroke | https://trialsearch.who.int/Trial2.aspx?TrialID=ChiCTR2300069426 | 2023 |  |  |  | Not relevant |
| ChiCtr 2023 | ChiCtr | A Clinical randomized controlled study of "music swallowing" training based on near-infrared brain function imaging technology and embodied virtual mirror visual feedback theory on swallowing function and emotional state after brainstem stroke | https://trialsearch.who.int/Trial2.aspx?TrialID=ChiCTR2300074848 | 2023 |  |  |  | Not relevant |
| ChiCtr 2023 | ChiCtr | Clinical study of cerebellar iTBS combined with lower limb rehabilitation robot training in the treatment of walking dysfunction after stroke | https://trialsearch.who.int/Trial2.aspx?TrialID=ChiCTR2300074994 | 2023 |  |  |  | Not relevant |
| ChiCtr 2023 | ChiCtr | Clinical study of walking and disturbance training for hip weight loss after stroke | https://trialsearch.who.int/Trial2.aspx?TrialID=ChiCTR2300071572 | 2023 |  |  |  | Wrong intervention - does not meet exercise definition |
| ChiCtr 2023 | ChiCtr | Clinical study on the effect of Tai Chi gait motor imagery training on lower limb motor function in stroke patients | https://trialsearch.who.int/Trial2.aspx?TrialID=ChiCTR2300073028 | 2023 |  |  |  | Not relevant |
| ChiCtr 2023 | ChiCtr | A Clinical study on the relief of lower extremity ankle plantarflexion spasticity by neurotomy of S2 nerve root +/- partial triceps branch | https://trialsearch.who.int/Trial2.aspx?TrialID=ChiCTR2300068145 | 2023 |  |  |  | Not relevant |
| ChiCtr 2023 | ChiCtr | Clinical trial of improvement of motor function by limb linkage training in stroke patients | https://trialsearch.who.int/Trial2.aspx?TrialID=ChiCTR2300069075 | 2023 |  |  |  | Not relevant |
| ChiCtr 2023 | ChiCtr | Construction and application of exercise rehabilitation system for patients with coronary heart disease after percutaneous coronary intervention (PCI) | https://trialsearch.who.int/Trial2.aspx?TrialID=ChiCTR2300071666 | 2023 |  |  |  | Wrong comparator - exercise v exercise |
| ChiCtr 2023 | ChiCtr | Effect analysis of the "hospital-community-family" ternary linkage cardiac rehabilitation in patients with acute myocardial infarction after percutaneous coronary intervention | https://trialsearch.who.int/Trial2.aspx?TrialID=ChiCTR2300074176 | 2023 |  |  |  | Not relevant |
| ChiCtr 2023 | ChiCtr | Effect and mechanism of foaming therapy on motor function in convalescent patients with ischemic stroke: a single-center, double-blind, randomized, parallel controlled study | https://trialsearch.who.int/Trial2.aspx?TrialID=ChiCTR2300069467 | 2023 |  |  |  | Not relevant |
| ChiCtr 2023 | ChiCtr | Effect of backward walking combined with scalp acupuncture on cerebral cortex function in stroke patients | https://trialsearch.who.int/Trial2.aspx?TrialID=ChiCTR2300075413 | 2023 |  |  |  | Wrong intervention - does not meet exercise definition |
| ChiCtr 2023 | ChiCtr | Effect of dynamic balance training on balance ability and walking function in hospital patients with hemiplegia after stroke | https://trialsearch.who.int/Trial2.aspx?TrialID=ChiCTR2300070423 | 2023 |  |  |  | Not relevant |
| ChiCtr 2023 | ChiCtr | Effects of C-Mill gait adaptation training combined with transcranial magnetic stimulation on walking function and its neural mechanism in stroke patients based on near infrared and electroencephalogram technology | https://trialsearch.who.int/Trial2.aspx?TrialID=ChiCTR2300070367 | 2023 |  |  |  | Not relevant |
| ChiCtr 2023 | ChiCtr | Effects of computerized cognitive training combined with aerobic exercise on patients with PSCI | https://trialsearch.who.int/Trial2.aspx?TrialID=ChiCTR2300076646 | 2023 |  |  |  | Wrong comparator - exercise v exercise |
| ChiCtr 2023 | ChiCtr | Effects of Mobile Robot-assisted Walking Training on Walking Function and Lower Extremity Surface Electromyography in Patients with Hemiplegia after Stroke | https://trialsearch.who.int/Trial2.aspx?TrialID=ChiCTR2300073389 | 2023 |  |  |  | Not relevant |
| ChiCtr 2023 | ChiCtr | Influence of training with intelligent walking aid under suspension protection on patients with early stroke | https://trialsearch.who.int/Trial2.aspx?TrialID=ChiCTR2300070247 | 2023 |  |  |  | Not relevant |
| ChiCtr 2023 | ChiCtr | Observation on the therapeutic effect of manual therapy based on Posture Secret on walking function of stroke patients with hemiplegia | https://trialsearch.who.int/Trial2.aspx?TrialID=ChiCTR2300067792 | 2023 |  |  |  | Not relevant |
| ChiCtr 2023 | ChiCtr | Rehabilitation training participated by caregivers in ischemic stroke: protocol for a randomized controlled trial to test the effect of home-based rehabilitation intervention on limb motor function | https://trialsearch.who.int/Trial2.aspx?TrialID=ChiCTR2300078798 | 2023 |  |  |  | Not relevant |
| ChiCtr 2023 | ChiCtr | Study of cognitive dysfunction of the brain in patients with myocardial infarction and the role of exercise therapy | https://trialsearch.who.int/Trial2.aspx?TrialID=ChiCTR2300067679 | 2023 |  |  |  | Wrong intervention - does not meet exercise definition |
| ChiCtr 2023 | ChiCtr | To evaluate the efficacy and safety of digital therapy for tele-rehabilitation in patients with acute coronary syndrome after percutaneous coronary intervention: a single-center, prospective, open-label, randomized controlled Clinical study | https://trialsearch.who.int/Trial2.aspx?TrialID=ChiCTR2300071318 | 2023 |  |  |  | Not relevant |
| ChiCtr 2023 | ChiCtr | The effects of blood flow restriction combined with walking training on lower limb motor function and balance in stroke patients: a Clinical trial |  | 2023 |  |  |  | Not relevant |
| ChiCtr 2023 | ChiCtr | Exploration and evaluation of comprehensive rehabilitation model for stroke-related sarcopenia |  | 2023 |  |  |  | Wrong comparator - active control |
| ChiCtr 2024 | ChiCtr | Application of Remote Cardiac Rehabilitation in Patients with Coronary Heart Disease: a Randomized Controlled Study | https://trialsearch.who.int/Trial2.aspx?TrialID=ChiCTR2400079467 | 2024 |  |  |  | Wrong comparator - exercise v exercise |
| ChiCtr 2024 | ChiCtr | Clinical Study of a Novel Rehabilitation Technique for Post-Stroke Hemiplegia Based on Motion Visual Capture and Electromyography Analysis in Traditional Chinese Medicine Practice |  | 2024 |  |  |  | Wrong intervention - does not meet exercise definition |
| ChiCtr 2024 | ChiCtr | The effect of enhanced symmetrical weight-bearing sitting training on the effectiveness and safety of walking in stroke patients with hemiplegia |  | 2024 |  |  |  | Not relevant |
| ChiCtr 2024 | ChiCtr | Construction and application of early progressive activity program in patients with acute decompensated heart failure based on the Temporal Self-regulation theory |  | 2024 |  |  |  | Wrong study design - not RCT |
| Chihara 2016 | Chihara, H.; Takagi, Y.; Nishino, K.; Yoshida, K.; Arakawa, Y.; Kikuchi, T.; Takenobu, Y.; Miyamoto, S. | Factors Predicting the Effects of Hybrid Assistive Limb Robot Suit during the Acute Phase of Central Nervous System Injury | Neurologia Medico-Chirurgica | 2016 | 56 | 1 | 33-7 | Not relevant |
| Chimura 2023 | Chimura, M.; Koba, S.; Sakata, Y.; Ise, T.; Miura, H.; Murai, R.; Suzuki, H.; Maekawa, E.; et al | Evaluation of the efficacy and safety of an integrated telerehabilitation platform for home-based cardiac REHABilitation in patients with heart failure (E-REHAB): protocol for a randomised controlled trial | BMJ Open | 2023 | 13 | 8 | e073846 | Wrong comparator - exercise v exercise |
| Chin 2021 | Chin, L. F.; Hayward, K. S.; Chai, A. L. M.; Brauer, S. G. | A Self-Empowered Upper Limb Repetitive Engagement Program to Improve Upper Limb Recovery Early Post-Stroke: phase II Pilot Randomized Controlled Trial | NeuroRehabilitation and Neural Repair | 2021 | 35 | 9 | 836-848 | Not relevant |
| Chitra 2015 | Chitra, J.; Sharan, R. | A COMPARATIVE STUDY ON THE EFFECTIVENESS OF CORE STABILITY EXERCISE AND PELVIC PROPRIOCEPTIVE NEUROMUSCULAR FACILITATION ON BALANCE, MOTOR RECOVERY AND FUNCTION IN HEMIPARETIC PATIENTS: A RANDOMIZED CLINICAL TRIAL | Romanian Journal of Physical Therapy / Revista Romana de Kinetoterapie | 2015 | 21 | 36 | 12-18 | Wrong intervention - does not meet exercise definition |
| Chlebus 2019 | Chlebus, E.; Warenczak, A.; Miedzyblocki, M.; Lisinski, P. | The usefulness of isometric protocol for foot flexors and extensors in assessing the effects of 16-week rehabilitation regiment in poststroke patients | BioMedical Engineering Online | 2019 | 18 | 1 | 57 | Not relevant |
| Cho 2020 | Cho, H.; Kim, K. | Effects of Action Observation Training with Auditory Stimulation on Static and Dynamic Balance in Chronic Stroke Patients | Journal of Stroke and Cerebrovascular Diseases | 2020 | 29 | 5 | 104775 | Not relevant |
| Cho 2019 | Cho, J.; Lee, E.; Lee, S. | Effectiveness of mid-thoracic spine mobilization versus therapeutic exercise in patients with subacute stroke: a randomized Clinical trial | Technology and Health Care | 2019 | 27 | 2 | 149-158 | Wrong intervention - does not meet exercise definition |
| Choi 2020 | Choi, M. S.; Kim, Y. M.; Kyoung,; Lee, J. S. | Effects of treadmill gait training according to different inclination on pulmonary function in patients with chronic stroke | Indian Journal of Public Health Research and Development | 2020 | 11 | 7 | 1539-1544 | Wrong comparator - exercise v exercise |
| Choi 2022 | Choi, S. B.; Choi, S. Y.; Lee, E. S.; Kim, J. H. | Effects of cardiac rehabilitation in older female coronary artery patients | European Geriatric Medicine | 2022 | 13 | 1 | S304 | Wrong study design - not RCT |
| Choi 2022 | Choi, W. | Effects of Robot-Assisted Gait Training with Body Weight Support on Gait and Balance in Stroke Patients | International Journal of Environmental Research and Public Health | 2022 | 19 | 10 |  | Not relevant |
| Choi 2017 | Choi, W.; Han, D.; Kim, J.; Lee, S. | Whole-Body Vibration Combined with Treadmill Training Improves Walking Performance in Post-Stroke Patients: A Randomized Controlled Trial | Medical Science Monitor | 2017 | 23 |  | 4918-4925 | Not relevant |
| Choi 2020 | Choi, Y-H.; Kim, K.; Lee, S-Y.; Cha, Y-J. | Lower limb muscle activities and gain in balancing ability following two types of stair gait intervention in adult post-chronic stroke patients: A preliminary, randomized-controlled study | Turkish Journal of Physical Medicine & Rehabilitation (2587-1250) | 2020 | 66 | 1 | 17-23 | Not relevant |
| Choi 2019 | Choi, Y. H.; Kim, J. D.; Lee, J. H.; Cha, Y. J. | Walking and balance ability gain from two types of gait intervention in adult patients with chronic hemiplegic stroke: A pilot study | Assistive Technology | 2019 | 31 | 2 | 112-115 | Not relevant |
| Choi 2020 | Choi, Y. H.; Kim, N. H.; Son, S. M.; Cha, Y. J. | Effects of Trunk Stabilization Exercise While Wearing a Pelvic Compression Belt on Walking and Balancing Abilities in Patients With Stroke: An Assessor Blinded, Preliminary, Randomized, Controlled Study | American Journal of Physical Medicine & Rehabilitation | 2020 | 99 | 11 | 1048-1055 | Wrong intervention - does not meet exercise definition |
| Chokshi 2018 | Chokshi, N. P.; Adusumalli, S.; Small, D. S.; Morris, A.; Feingold, J.; Ha, Y. P.; Lynch, M. D.; Rareshide, C. A. L.; Hilbert, V.; Patel, M. S. | Loss-Framed Financial Incentives and Personalized Goal-Setting to Increase physical activity Among Ischemic Heart Disease Patients Using Wearable Devices: The ACTIVE REWARD Randomized Trial | Journal of the American Heart Association | 2018 | 7 | 12 | 13 | Not relevant |
| Chokshi 2017 | Chokshi, N. P.; Adusumalli, S.; Small, D. S.; Morris, A.; Feingold, J.; Ha, Y. P.; Rareshide, C. A.; Hilbert, V.; Patel, M. S. | Loss-framed financial incentives and personalized goal setting increase physical activity in ischemic heart disease patients using wearable devices: the ACTIVE REWARD randomized Clinical trial | Circulation | 2017 | 136 |  |  | Not relevant |
| Cholet 2021 | Cholet, Centre Hospitalier de | Maintenance of physical Condition and physical activity at One Year After Cardiac Rehabilitation Program |  | 2021 |  |  |  | Not relevant |
| Chong 2022 | Chong, M. S.; Sit, J. W. H.; Choi, K. C.; Suhaimi, A.; Chair, S. Y. | Feasibility and preliminary effects of technology-assisted interventions in hybrid cardiac rehabilitation (TecHCR): a pilot randomised controlled trial | European Journal of Preventive Cardiology | 2022 | 29 | 1 | i308 | Wrong comparator - active control |
| Chong 2023 | Chong, M. S.; Sit, J. W. H.; Choi, K. C.; Suhaimi, A.; Chair, S. Y. | A Theory-Based, Technology-Assisted Intervention in a Hybrid Cardiac Rehabilitation Program for Patients with Coronary Heart Disease: A Feasibility Study | Asian Nursing Research | 2023 | 17 | 3 | 180-190 | Wrong comparator - active control |
| Choudhary 2018 | Choudhary, N. | A novel approach to training eye hand coordination for improving arm function in chronic stroke survivors-a randomized controlled study | International Journal of Stroke | 2018 | 13 | 2 | 12 | Not relevant |
| Choudhury 2020 | Choudhury, S.; Singh, R.; Shobhana, A.; Sen, D.; Anand, S. S.; Shubham, S.; Gangopadhyay, S.; Baker, M. R.; Kumar, H.; Baker, S. N. | A Novel Wearable Device for Motor Recovery of Hand Function in Chronic Stroke Survivors | NeuroRehabilitation and Neural Repair | 2020 | 34 | 7 | 600-608 | Not relevant |
| Christa 2019 | Christa, E.; Srivastava, P.; Chandran, D. S.; Jaryal, A. K.; Yadav, R. K.; Roy, A.; Deepak, K. K. | Effect of Yoga-Based Cardiac Rehabilitation on Heart Rate Variability: Randomized Controlled Trial in Patients Post-MI | International Journal of Yoga Therapy | 2019 | 29 | 1 | 43-50 | Wrong comparator - co-interventions not balanced between arms |
| Christa 2023 | Christa, E.; Srivastava, P.; Chandran, D. S.; Jaryal, A. K.; Yadav, R. K.; Roy, A.; Deepak, K. K. | Effect of Yoga Based Cardiac Rehabilitation on Blood Pressure Variability and Baroreflex Sensitivity: RCT in Patients Post MI | Applied psychophysiology and biofeedback | 2023 | 48 | 1 | 1-15 | Wrong comparator - co-interventions not balanced between arms |
| Chu 2015 | Chu, J.; Bao, Y.; Zhu, M. | Effects of Acupuncture Intervention Combined with Rehabilitation on Standing-balance-walking Ability in Stroke Patients. [Chinese] | Zhen ci yan jiu = Acupuncture Research / [Zhongguo yi xue ke xue yuan Yi xue qing bao yan jiu suo bian ji] | 2015 | 40 | 6 | 474-478 | Full text not in English |
| Chua 2016 | Chua, J.; Culpan, J.; Menon, E. | Efficacy of an Electromechanical Gait Trainer Poststroke in Singapore: a Randomized Controlled Trial | Archives of Physical Medicine and Rehabilitation | 2016 | 97 | 5 | 683-690 | Not relevant |
| Chung 2020 | Chung, B. P. H.; Chiang, W. K. H.; Lau, H.; Lau, T. F. O.; Lai, C. W. K.; Sit, C. S. Y.; Chan, K. Y.; Yeung, C. Y.; Lo, T. M.; Hui, E.; Lee, J. S. W. | Pilot study on comparisons between the effectiveness of mobile video-guided and paper-based home exercise programs on improving exercise adherence, self-efficacy for exercise and functional outcomes of patients with stroke with 3-month follow-up: a single-blind randomized controlled trial | Hong kong Physiotherapy Journal : official publication of the hong kong Physiotherapy association limited = wu li chih liao | 2020 | 40 | 1 | 63-73 | Not relevant |
| Chung 2016 | Chung, S. M.; Lee, K. B.; Kim, Y. D. | Effects of shoulder reaching exercise on the balance of patients with hemiplegia after stroke | Journal of Physical Therapy Science | 2016 | 28 | 7 | 2151-2153 | Not relevant |
| Chung-shan 2019 | Chung-shan, H.; Keh-chung, L.; Wan-ying, C.; Wen-chih, H.; Ya-Ju, C.; Chia-ling, C.; Kaiping G., Yao; Ya-yun, L. | Unilateral vs Bilateral Hybrid Approaches for Upper Limb Rehabilitation in Chronic Stroke: A Randomized Controlled Trial Controlled Trial | Archives of Physical Medicine & Rehabilitation | 2019 | 100 | 12 | 2225-2232 | Not relevant |
| CİN 2022 | CİN, Aynur; University, Karadeniz Technical | The Effect of Green Walking on Myocardial Infarction Patients |  | 2022 |  |  |  | Wrong study design - not RCT |
| Cinar 2016 | Cinar, E.; Karapolat, H.; Capaci, K.; Engin, C.; Yagdi, T.; Ozbaran, M.; Nalbantgil, S.; Zoghi, M. | The effect of cardiac rehabilitation on functional capacity, psychological symptoms and quality of life in patients with a left ventricular assist device | Journal of Heart and Lung Transplantation | 2016 | 1 |  | S395 | Wrong comparator - active control |
| Cincinnati 2017 | Cincinnati, University of | The Effect of Yoga on Cardiac Sympathetic Innervation Evaluated by I-123 mIBG |  | 2017 |  |  |  | Not relevant |
| Clague-Baker 2016 | Clague-Baker, N.; Robinson, T.; Drewry, S.; Hagenberg, A.; Gillies, C.; Singh, S. | A comparison of laboratory and field based exercise tests of cardiovascular fitness in sub-acute, mild to moderate stroke patients | International Journal of Stroke | 2016 | 11 | 4 | 21 | Not relevant |
| Clague-Baker 2022 | Clague-Baker, N.; Robinson, T.; Gillies, C. L.; Drewry, S.; Hagenberg, A.; Singh, S. | Adapted cardiac rehabilitation for people with sub-acute, mild-to-moderate stroke: a mixed methods feasibility study | Physiotherapy | 2022 | 115 |  | 93-101 | Not relevant |
| Clanchy 2016 | Clanchy, K. M.; Tweedy, S. M.; Trost, S. G. | Evaluation of a physical activity Intervention for Adults with Brain Impairment: a Controlled Clinical Trial | NeuroRehabilitation and Neural Repair | 2016 | 30 | 9 | 854-865 | Not relevant |
| Clark 2021 | Clark, D. J.; Rose, D. K.; Butera, K. A.; Hoisington, B.; DeMark, L.; Chatterjee, S. A.; Hawkins, K. A.; Otzel, D. M.; Skinner, J. W.; Christou, E. A.; Wu, S. S.; Fox, E. J. | Rehabilitation with accurate adaptability walking tasks or steady state walking: A randomized Clinical trial in adults post-stroke | Clinical Rehabilitation | 2021 | 35 | 8 | 1196-1206 | Not relevant |
| Clays 2021 | Clays, E.; Puddu, P. E.; Luštrek, M.; Pioggia, G.; Derboven, J.; Vrana, M.; De Sutter, J.; Le Donne, R.; et al. | Proof-of-concept trial results of the HeartMan mobile personal health system for self-management in congestive heart failure | Scientific reports | 2021 | 11 | 1 | 5663 | Not relevant |
| Clinic 2021 | Clinic, Mayo; Research, National Institute of Nursing | Improving Cardiac Rehabilitation Outcomes Through Mobile Case Management (iCARE) |  | 2021 |  |  |  | Wrong comparator - co-interventions not balanced between arms |
| Coca-Martinez 2023 | Coca-Martinez, M.; Girsowicz, E.; Doonan, R. J.; Obrand, D. I.; Bayne, J. P.; Steinmetz, O. K.; Mackenzie, K. S.; Carli, F.; Martinez-Palli, G.; Gill, H. L. | Multimodal Prehabilitation for Peripheral Arterial Disease Patients with Intermittent Claudication-A Pilot Randomized Controlled Trial | Annals of Vascular Surgery. | 2023 |  |  |  | Wrong comparator - co-interventions not balanced between arms |
| Coca-Martinez 2021 | Coca-Martinez, M.; Vitagliano, M.; Girsowicz, E. E.; Obrand, D. I.; Steinmetz, O. K.; Bayne, J. P.; Mackenzie, K. S.; Carli, F. F.; Gill, H. L. | Multimodal Prehabilitation for Peripheral Arterial Disease: results of an In-Trial Pilot Randomized Controlled Trial | Journal of Vascular Surgery | 2021 | 74 | 5 | e426-e427 | Wrong comparator - co-interventions not balanced between arms |
| Collett 2021 | Collett, J.; Fleming, M. K.; Meester, D.; Al-Yahya, E.; Wade, D. T.; Dennis, A.; Salvan, P.; Meaney, A.; Cockburn, J.; Dawes, J.; Johansen-Berg, H.; Dawes, H. | Dual-task walking and automaticity after Stroke: Insights from a secondary analysis and imaging sub-study of a randomised controlled trial | Clinical Rehabilitation | 2021 | 35 | 11 | 1599-1610 | Not relevant |
| Collins 2023 | Collins, B.; Gordon, B.; Wundersitz, D.; Hunter, J.; Hanson, L. C.; O'Doherty, A. F.; Hayes, A.; Kingsley, M. | Comparison of telehealth and supervised phase III cardiac rehabilitation in regional Australia: protocol for a non-inferiority trial | BMJ Open | 2023 | 13 | 6 | e070872 | Wrong comparator - exercise v exercise |
| Collins 2022 | Collins, T.; Geana, M.; Overton, K.; Benton, M.; Lu, L.; Khan, F.; Rohleder, M.; Ahluwalia, J.; Resnicow, K.; Zhu, Y. | Use of a Smartphone App Versus Motivational Interviewing to Increase Walking Distance and Weight Loss in Overweight/Obese Adults With Peripheral Artery Disease: Pilot Randomized Trial | JMIR formative Research | 2022 | 6 | 2 | e30295 | Not relevant |
| Collins 2017 | Collins, T. C.; Lu, K.; He, J. | Promoting walking among african americans with peripheral artery disease | Journal of general Internal Medicine | 2017 | 32 | 2 | S291 | Not relevant |
| Collins 2019 | Collins, T. C.; Lu, L.; Ahluwalia, J. S.; Nollen, N. L.; Sirard, J.; Marcotte, R.; Post, S.; Zackula, R. | Efficacy of Community-Based Exercise Therapy Among African American Patients With Peripheral Artery Disease: A Randomized Clinical Trial | JAMA Network Open | 2019 | 2 | 2 | e187959 | Not relevant |
| Coombs 2023 | Coombs, G. B.; Al-Khazraji, B. K.; Suskin, N.; Shoemaker, J. K. | Impact of ischemic heart disease and cardiac rehabilitation on cerebrovascular compliance | Journal of Applied Physiology | 2023 | 135 | 4 | 753-762 | Wrong study design - not RCT |
| Corporation 2017 | Corporation, Hamad Medical | PROspective Study to OPTimize thE HEALTH of Patients With TIAs (Transient Ischemic Attacks) and Stroke Admitted to the Hamad General Hospital |  | 2017 |  |  |  | Not relevant |
| Corre 2016 | Corre, J.; Bonnet, G.; Poustis, P.; Douard, H. | High-intensity interval training versus moderate continuous training in coronary artery disease. A randomized controlled trial | Archives of Cardiovascular Diseases Supplements | 2016 | 8 | 3 | 265 | Wrong comparator - exercise v exercise |
| Corre 2016 | Corre, J.; Bonnet, G.; Poustis, P.; Silva, C.; Minvielle, C.; Aimable, L.; Douard, H. | High-intensity interval training versus moderate continuous training in coronary artery disease. A randomized controlled trial | Archives of Cardiovascular Diseases Supplements | 2016 | 1) |  | 90-91 | Wrong comparator - exercise v exercise |
| Correia 2020 | Correia, Marilia A.; Oliveira, Paulo L.; Farah, Breno Q.; Vianna, Lauro C.; Wolosker, Nelson; Puech-Leao, Pedro; Green, Daniel J.; Cucato, Gabriel G.; Ritti-Dias, Raphael M.; A Correia, Marilia | Effects of Isometric Handgrip Training in Patients With Peripheral Artery Disease: A Randomized Controlled Trial | Journal of the American Heart Association | 2020 | 9 | 4 | 1-12 | Not relevant |
| Coruña 2016 | Coruña, Universidade da | Effects of an Aquatic Therapy Program Versus a Land Program in Patients Who Suffered a Stroke |  | 2016 |  |  |  | Wrong intervention - does not meet exercise definition |
| Coruña 2017 | Coruña, University Hospital A. | Secondary PRevention in Cardiovascular Disease by a Nursing Guided Program (SPRING) |  | 2017 |  |  |  | Not relevant |
| Costa 2021 | Costa, E. C.; Cucato, G. G.; Ritti-Dias, R. M. | Effect of Low-Intensity vs High-Intensity Walking Exercise on Walk Distance in Patients with Peripheral Artery Disease | JAMA Journal of the American Medical Association | 2021 | 326 | 8 | 767-768 | Not relevant |
| Costa 2021 | Costa, R.; Frias, A.; Campinas, A.; Fernandes, P.; Magalhaes, S.; Santos, M.; Torres, S. | Impact of cardiac rehabilitation on inflammation in patients with ischaemic cardiomyopathy | European Heart Journal | 2021 | 42 | 1 | 2685 | Wrong study design - not RCT |
| Cox 2017 | Cox, K. L.; Cyarto, E. V.; Ellis, K. A.; Ames, D.; Desmond, P.; Phal, P.; Sharman, M.; Salvado, O.; Szoeke, C.; Lautenschlager, N. T. | The short-and long-term effects of a 24-month home-based physical activity program on physical activity levels and body composition of older adults at risk of Alzheimer's disease: the Aibl active trial | Alzheimer's & Dementia | 2017 | 13 | 7 | P895 | Not relevant |
| Cramer 2018 | Cramer, S.; Lucy, D.; Le, V.; Jill, S.; Renee, A.; Alison, M.; Robert, Z.; Nina, C.; et al | Telerehabilitation in the home versus therapy in-clinic for patients with stroke | European Stroke Journal | 2018 | 3 | 1 | 590-591 | Not relevant |
| Cramer 2020 | Cramer, S. C.; Dodakian, L.; Le, V.; McKenzie, A.; See, J.; Augsburger, R.; Zhou, R. J.; Raefsky, S. M.; Nguyen, T.; Vanderschelden, B.; Wong, G.; Bandak, D.; Nazarzai, L.; Dhand, A.; Scacchi, W.; Heckhausen, J. | A Feasibility Study of Expanded Home-Based Telerehabilitation After Stroke | Frontiers in Neurology | 2020 | 11 |  |  | Not relevant |
| Cramer 2019 | Cramer, S. C.; Dodakian, L.; Le, V.; See, J.; Augsburger, R.; McKenzie, A.; Zhou, R. J.; Chiu, N. L.; et al. | Efficacy of Home-Based Telerehabilitation vs In-Clinic Therapy for Adults after Stroke: a Randomized Clinical Trial | JAMA neurology | 2019 | 76 | 9 | 1079-1087 | Not relevant |
| Crema 2021 | Crema, A.; Furfaro, I.; Raschella, F.; Rossini, M.; Zajc, J.; Wiesener, C.; Baccinelli, W.; Proserpio, D.; et al | Reactive Exercises with Interactive Objects: Interim Analysis of a Randomized Trial on Task-Driven NMES Grasp Rehabilitation for Subacute and Early Chronic Stroke Patients | Sensors | 2021 | 21 | 20 | 11 | Not relevant |
| Criekinge 2020 | Criekinge, T. V.; Hallemans, A.; Herssens, N.; Lafosse, C.; Claes, D.; Hertogh, W. D.; Truijen, S.; Saeys, W. | SWEAT2 Study: effectiveness of Trunk Training on Gait and Trunk Kinematics After Stroke: a Randomized Controlled Trial | Physical Therapy | 2020 | 100 | 9 | 1568-1581 | Not relevant |
| Cronin 2023 | Cronin, E.; Monaghan, K. | Online neuropilates classes in chronic stroke patients: protocol for a randomised controlled feasibility study | Contemporary Clinical Trials Communications | 2023 | 32 |  | 101068 | Wrong comparator - exercise v exercise |
| Crozier 2023 | Crozier, A.; Cocks, M.; Hesketh, K.; Miller, G.; McGregor, G.; Thomas, L.; Jones, H. | MOBILE HEALTH BIOMETRICS TO PRESCRIBE IMMEDIATE REMOTE PHYSICAL ACTIVITY FOR ENHANCING UPTAKE TO CARDIAC REHABILITATION (MOTIVATE-CR+): PROTOCOL FOR A RANDOMISED CONTROLLED FEASIBILITY TRIAL | Heart (British Cardiac Society) | 2023 | 109 | 5 | A14-A15 | Not relevant |
| Crozier 2018 | Crozier, J.; Roig, M.; Eng, J.; MacKay-Lyons, M.; Ploughman, M.; Fung, J.; Bailey, D.; Sweet, S.; Giacomoantonio, N.; Thiel, A.; Trivino, M.; Tang, A. | High-intensity interval training after stroke as an opportunity to promote functional recovery, cardiovascular health and neuroplasticity: a Clinical perspective | International Journal of Stroke | 2018 | 13 | 2 | 65 | Not relevant |
| Crozier 2018 | Crozier, J.; Roig, M.; Eng, J. J.; MacKay-Lyons, M.; Fung, J.; Ploughman, M.; Bailey, D. M.; Sweet, S.N.; et al. | High-Intensity Interval Training After Stroke: An Opportunity to Promote Functional Recovery, Cardiovascular Health, and Neuroplasticity | NeuroRehabilitation & Neural Repair | 2018 | 32 | 6-7 | 543-556 | Not relevant |
| Ctri 2016 | CTRI | Effectiveness of Physiotherapy training to improve control of sitting on floor | https://trialsearch.who.int/Trial2.aspx?TrialID=CTRI/2016/10/007337 | 2016 |  |  |  | Not relevant |
| Ctri 2016 | CTRI | Exercise using all four limbs in half-side paralysis | https://trialsearch.who.int/Trial2.aspx?TrialID=CTRI/2016/09/007258 | 2016 |  |  |  | Wrong intervention - does not meet exercise definition |
| Ctri 2016 | CTRI | Role of unaffected arm in paralytic patients | https://trialsearch.who.int/Trial2.aspx?TrialID=CTRI/2016/09/007248 | 2016 |  |  |  | Not relevant |
| Ctri 2017 | CTRI | Benefits of Arm-Hand Practice with Splinting of Wrist on Hand Function in Stroke | https://trialsearch.who.int/Trial2.aspx?TrialID=CTRI/2017/04/008426 | 2017 |  |  |  | Not relevant |
| Ctri 2017 | CTRI | Benefits of Training Pelvis on Leg Movement and Walking Recovery in People with Stroke | https://trialsearch.who.int/Trial2.aspx?TrialID=CTRI/2017/04/008309 | 2017 |  |  |  | Wrong intervention - does not meet exercise definition |
| Ctri 2017 | CTRI | Benefits of Treating Trunk on Strength and Community Participation in Late Stage Stroke | https://trialsearch.who.int/Trial2.aspx?TrialID=CTRI/2017/04/008355 | 2017 |  |  |  | Wrong intervention - does not meet exercise definition |
| Ctri 2017 | CTRI | A Clinical trial to identify effect of arm exercises along with behavioural support to increase arm use after stroke | https://trialsearch.who.int/Trial2.aspx?TrialID=CTRI/2017/10/010108 | 2017 |  |  |  | Not relevant |
| Ctri 2017 | CTRI | A Clinical trial to study the effects of two trunk exercises regimes on trunk control and balance, physio ball and therapeutic mat in patients with stroke | https://trialsearch.who.int/Trial2.aspx?TrialID=CTRI/2017/02/007760 | 2017 |  |  |  | Not relevant |
| Ctri 2017 | CTRI | The effect of Task Oriented Circuit Training on Hand function in people with one-sided weakness living in the society | http://www.who.int/trialsearch/Trial2.aspx?TrialID=CTRI/2017/05/008482 | 2017 |  |  |  | Not relevant |
| Ctri 2017 | CTRI | A study to check the effects of cardiac rehabilitation program by the nurses involving patient teaching and counseling for the patients undergoing Angioplasty. It can help to reduce Blood Pressure, Body weight, Cholesterol levels, also help in quitting smoking and improving exercise among patients | https://trialsearch.who.int/Trial2.aspx?TrialID=CTRI/2017/03/008022 | 2017 |  |  |  | Not relevant |
| Ctri 2018 | CTRI | Adherence to home-based exercises among stroke survivors | https://trialsearch.who.int/Trial2.aspx?TrialID=CTRI/2018/08/015212 | 2018 |  |  |  | Not relevant |
| Ctri 2018 | CTRI | Arm function training with visual and auditory feedback | https://trialsearch.who.int/Trial2.aspx?TrialID=CTRI/2018/12/016690 | 2018 |  |  |  | Not relevant |
| Ctri 2018 | CTRI | Checking the effect of specific strengthening of core muscles in addition to regular trunk physiotherapy program on the ability to detect the trunk position sense and ability to move around in surrounding | https://trialsearch.who.int/Trial2.aspx?TrialID=CTRI/2018/08/015323 | 2018 |  |  |  | Wrong comparator - active control |
| Ctri 2018 | CTRI | A computer game based exercise protocol for patients with arm and hand problems after stroke | https://trialsearch.who.int/Trial2.aspx?TrialID=CTRI/2018/06/014577 | 2018 |  |  |  | Not relevant |
| Ctri 2018 | CTRI | Effect of continuous monitoring of exercise rehabilitation program through whatsapp/skype video calls on exercise capacity, functional status and health related quality of life in patients with coronary artery bypass graft | https://trialsearch.who.int/Trial2.aspx?TrialID=CTRI/2018/05/013575 | 2018 |  |  |  | Not relevant |
| Ctri 2018 | CTRI | Effect of Exercises in improving the overall health and heart functions in Patients following a heart attack | https://trialsearch.who.int/Trial2.aspx?TrialID=CTRI/2018/05/013649 | 2018 |  |  |  | Wrong comparator - exercise v exercise |
| Ctri 2019 | CTRI | íƒÂ¢??Effectiveness of Graded Motor Imagery (GMI) on upper limb motor function in patients with subacute stroke íƒÂ¢?? | https://trialsearch.who.int/Trial2.aspx?TrialID=CTRI/2019/01/016966 | 2019 |  |  |  | Not relevant |
| Ctri 2019 | CTRI | Effect of cardiac rehabilitation in patients undergone myocardial infarction and percutaneous coronary intervention | https://trialsearch.who.int/Trial2.aspx?TrialID=CTRI/2019/06/019948 | 2019 |  |  |  | Wrong comparator - co-interventions not balanced between arms |
| Ctri 2019 | CTRI | Physical activity in stroke | https://trialsearch.who.int/Trial2.aspx?TrialID=CTRI/2019/05/019478 | 2019 |  |  |  | Wrong comparator - co-interventions not balanced between arms |
| Ctri 2019 | CTRI | Role of Targeted and assisted physiotherapy in post-stroke rehabilitation | https://trialsearch.who.int/Trial2.aspx?TrialID=CTRI/2019/04/018667 | 2019 |  |  |  | Not relevant |
| Ctri 2020 | CTRI | ASSESSMENT OF THE EFFECT OF CARDIAC REHABILITATION PROGRAM ON LIFE STYLE, MEDICATION ADHERENCE, QUALITY OF LIFE LIFE, FUNCTIONAL CAPACITY, BLOOD PRESSURE, BLOOD SUGAR AND CHOLESTEROL LEVEL AMONG PATIENTS UNDERGOING ANGIOPLASTY | https://trialsearch.who.int/Trial2.aspx?TrialID=CTRI/2020/12/029875 | 2020 |  |  |  | Wrong comparator - co-interventions not balanced between arms |
| Ctri 2020 | CTRI | A Clinical trail to analyze the effect of Physiotherapy treatment ,PNF resistance training of the unaffected arm on recovery of the affected arm in stroke patients | https://trialsearch.who.int/Trial2.aspx?TrialID=CTRI/2020/03/024032 | 2020 |  |  |  | Wrong intervention - does not meet exercise definition |
| Ctri 2020 | CTRI | Effectiveness of center and home based Cardiac Rehabilitation on Psychological distress, Sense of Coherence and Quality of Life among patients undergoing Coronary Artery Bypass Graft (CABG) surgery | https://trialsearch.who.int/Trial2.aspx?TrialID=CTRI/2020/12/029596 | 2020 |  |  |  | Wrong comparator - exercise v exercise |
| Ctri 2020 | CTRI | Efficacy of trunk neurodevelopmental therapy on trunk control and upper limb function in stroke patients: a randomized controlled trial | https://trialsearch.who.int/Trial2.aspx?TrialID=CTRI/2020/10/028393 | 2020 |  |  |  | Not relevant |
| Ctri 2020 | CTRI | A Randomised Clinical trial for Rehabilitation intervention in Acute Stroke Patients | https://trialsearch.who.int/Trial2.aspx?TrialID=CTRI/2020/04/024911 | 2020 |  |  |  | Wrong intervention - does not meet exercise definition |
| Ctri 2020 | CTRI | Study to determine if exercises involving daily tasks improve arm and hand function in those who had stroke recently | https://trialsearch.who.int/Trial2.aspx?TrialID=CTRI/2020/12/030144 | 2020 |  |  |  | Wrong intervention - does not meet exercise definition |
| Ctri 2020 | CTRI | To make lower limb activities better by using mirror in patient having weakness of half body | https://trialsearch.who.int/Trial2.aspx?TrialID=CTRI/2020/03/023773 | 2020 |  |  |  | Not relevant |
| Ctri 2021 | CTRI | Cardiac Rehabilitation intervention for women with Heart Diseases | https://trialsearch.who.int/Trial2.aspx?TrialID=CTRI/2021/07/035197 | 2021 |  |  |  | Wrong comparator - co-interventions not balanced between arms |
| Ctri 2021 | CTRI | A Clinical trial to assess the Effectiveness of lifestyle modification follow up program in patients with post myocardial infraction | https://trialsearch.who.int/Trial2.aspx?TrialID=CTRI/2021/05/033432 | 2021 |  |  |  | Not relevant |
| Ctri 2021 | CTRI | EFFECT OF COMBINED RHYTHMIC ARM MOVEMENT TRAINING ON BALANCE AND WALKING IN STROKE PATIENTS | https://trialsearch.who.int/Trial2.aspx?TrialID=CTRI/2021/09/036712 | 2021 |  |  |  | Wrong intervention < 6 weeks |
| Ctri 2021 | CTRI | Effect of Myofascial release on spastic muscles and functional outcome in chronic paralytic subject | https://trialsearch.who.int/Trial2.aspx?TrialID=CTRI/2021/03/031902 | 2021 |  |  |  | Not relevant |
| Ctri 2021 | CTRI | Effect of WippRacer Training to improve balance in stroke patiant | https://trialsearch.who.int/Trial2.aspx?TrialID=CTRI/2021/11/038227 | 2021 |  |  |  | Not relevant |
| Ctri 2021 | CTRI | Effects of Circuit Resistance Training, Conventional Resistance Training and Functional Electrical Stimulator (FES) on Patients with Spinal Cord Injury (SCI) | https://trialsearch.who.int/Trial2.aspx?TrialID=CTRI/2021/11/038271 | 2021 |  |  |  | Not relevant |
| Ctri 2021 | CTRI | Effects of home based cognitive (related to mental process involved in knowing learning and understanding) and balance training on fall incidences in recovering rural stroke patients | https://trialsearch.who.int/Trial2.aspx?TrialID=CTRI/2021/09/036384 | 2021 |  |  |  | Not relevant |
| Ctri 2021 | CTRI | Plinth Trunk Exercises and Physio Ball Exercises in Stroke | https://trialsearch.who.int/Trial2.aspx?TrialID=CTRI/2021/08/035913 | 2021 |  |  |  | Wrong intervention - does not meet exercise definition |
| Ctri 2021 | CTRI | Study of physical therapy rehabilitation of stroke patients given through teleMedicine comparing group therapy with individual therapy and in-person therapy | https://trialsearch.who.int/Trial2.aspx?TrialID=CTRI/2021/11/038077 | 2021 |  |  |  | Wrong intervention - does not meet exercise definition |
| Ctri 2021 | CTRI | A study to test whether mobile-based applications (apps) used along with home exercises taught to stroke patients after their discharge from hospital is effective or not | https://trialsearch.who.int/Trial2.aspx?TrialID=CTRI/2021/06/034305 | 2021 |  |  |  | Wrong intervention < 6 weeks |
| Ctri 2022 | CTRI | AA study to add on effect of alpha music rhythm on cognition and upper extremity motor recovery in people with chronic stroke or paralysis | https://trialsearch.who.int/Trial2.aspx?TrialID=CTRI/2022/04/041612 | 2022 |  |  |  | Not relevant |
| Ctri 2022 | CTRI | comparing exercise done at home with Multimodality exercise under supervision at cardiac rehabilitation center and home in Ischemic Heart disease | https://trialsearch.who.int/Trial2.aspx?TrialID=CTRI/2022/12/048318 | 2022 |  |  |  | Wrong comparator - exercise v exercise |
| Ctri 2022 | CTRI | Effect of cycle ergometer in phase - I cardiac rehabilitation | https://trialsearch.who.int/Trial2.aspx?TrialID=CTRI/2022/07/044295 | 2022 |  |  |  | Wrong intervention < 6 weeks |
| Ctri 2022 | CTRI | Effectiveness Of Various Walking Patterns On A Treadmill and The PNF Technique in Chronic Stroke | https://trialsearch.who.int/Trial2.aspx?TrialID=CTRI/2022/12/048324 | 2022 |  |  |  | Wrong intervention - does not meet exercise definition |
| Ctri 2022 | CTRI | Efficacy Of Two Types Of Rehabilitative Regimen in Patients Of Stroke | https://trialsearch.who.int/Trial2.aspx?TrialID=CTRI/2022/12/048076 | 2022 |  |  |  | Not relevant |
| Ctri 2022 | CTRI | formulation and testing of a mobilization algorithm | https://trialsearch.who.int/Trial2.aspx?TrialID=CTRI/2022/11/047582 | 2022 |  |  |  | Wrong comparator - active control |
| Ctri 2022 | CTRI | Stair Climbing Exercise in contrast to Rebound Exercise for Balance among Stroke Survivors | https://trialsearch.who.int/Trial2.aspx?TrialID=CTRI/2022/11/047239 | 2022 |  |  |  | Wrong intervention - does not meet exercise definition |
| Ctri 2022 | CTRI | a study to see effect of tibial nerve mobilization on muscle hypertonia (spasticity) and mobility in people with chronic stroke or paralysis | https://trialsearch.who.int/Trial2.aspx?TrialID=CTRI/2022/04/041665 | 2022 |  |  |  | Not relevant |
| Ctri 2022 | CTRI | Trunk and Hand Training Exercises in Stroke Patients | https://trialsearch.who.int/Trial2.aspx?TrialID=CTRI/2022/07/044286 | 2022 |  |  |  | Wrong intervention - does not meet exercise definition |
| Ctri 2023 | CTRI | An Ayurvedic management of Pakshagata-hemiplegia with sarvanga dhanyamla seka, sarvanga abhyanga nadi sweda,talam and eranda taila prayoga | https://trialsearch.who.int/Trial2.aspx?TrialID=CTRI/2023/08/056652 | 2023 |  |  |  | Not relevant |
| Ctri 2023 | CTRI | Changes in ability to perform ground level activity and balance after 12-week Yoga training in stroke patients | https://trialsearch.who.int/Trial2.aspx?TrialID=CTRI/2023/02/049507 | 2023 |  |  |  | Wrong intervention - does not meet exercise definition |
| Ctri 2023 | CTRI | A CLINICAL TRIAL TO STUDY THE EFFECTS OF EMG BIOFEEDBACK THERAPY WITH AND WITHOUT BOBATH THERAPY IN STROKE PATIENTS | https://trialsearch.who.int/Trial2.aspx?TrialID=CTRI/2023/04/051999 | 2023 |  |  |  | Wrong intervention - does not meet exercise definition |
| Ctri 2023 | CTRI | A comparative study on the effects of proprioceptive Neuromuscular Facilitation and Conventional physiotherapy treatment on upper limb and gait function in stroke subjects | https://trialsearch.who.int/Trial2.aspx?TrialID=CTRI/2023/05/052930 | 2023 |  |  |  | Wrong intervention - does not meet exercise definition |
| Ctri 2023 | CTRI | A COMPARISON OF ORDINARY SUPERVISION AND ONLINE SUPERVISION FOR AIM SPECIFIC EXERCISES IN IMPROVING UPPER LIMB FUNCTION IN STROKE PATIENTS | https://trialsearch.who.int/Trial2.aspx?TrialID=CTRI/2023/02/050093 | 2023 |  |  |  | Not relevant |
| Ctri 2023 | CTRI | effect of exercise training on routine activities and quality of life in heart diseases patients | https://trialsearch.who.int/Trial2.aspx?TrialID=CTRI/2023/04/051326 | 2023 |  |  |  | Wrong comparator - exercise v exercise |
| Ctri 2023 | CTRI | Effect of Home-based Cardiac Rehabilitation on Health Outcomes among patients with Heart Failure | https://trialsearch.who.int/Trial2.aspx?TrialID=CTRI/2023/11/060276 | 2023 |  |  |  | Not relevant |
| Ctri 2023 | CTRI | Effect of PNF Neck Patterns and Vestibular Rehabilitation for balance in stroke : RCT | https://trialsearch.who.int/Trial2.aspx?TrialID=CTRI/2023/11/059438 | 2023 |  |  |  | Not relevant |
| Ctri 2023 | CTRI | Effect of Yoga based Cardiac Rehabilitation (Yoga-CaRe) in Heart Failure | https://trialsearch.who.int/Trial2.aspx?TrialID=CTRI/2023/03/050656 | 2023 |  |  |  | Wrong comparator - co-interventions not balanced between arms |
| Ctri 2023 | CTRI | Impact of cardiac rehabilitation on strength and autonomic functions in stroke paitents | https://trialsearch.who.int/Trial2.aspx?TrialID=CTRI/2023/06/054265 | 2023 |  |  |  | Wrong comparator - exercise v exercise |
| Ctri 2023 | CTRI | Improving walking among post-stroke subjects using real-time visual feedback | https://trialsearch.who.int/Trial2.aspx?TrialID=CTRI/2023/10/058299 | 2023 |  |  |  | Wrong intervention - does not meet exercise definition |
| Ctri 2023 | CTRI | Manual Perturbation compare with PNF for Trunk Stability and Lower Extremity Function Stroke | https://trialsearch.who.int/Trial2.aspx?TrialID=CTRI/2023/07/054754 | 2023 |  |  |  | Wrong intervention - does not meet exercise definition |
| Ctri 2023 | CTRI | Novel Exercise for Stroke Patients | https://trialsearch.who.int/Trial2.aspx?TrialID=CTRI/2023/10/058803 | 2023 |  |  |  | Wrong intervention - does not meet exercise definition |
| Ctri 2023 | CTRI | Optimization and Efficacy of Exercise Protocols in Patients Suffering from Stroke : a Randomized Controlled Trial | https://trialsearch.who.int/Trial2.aspx?TrialID=CTRI/2023/06/053372 | 2023 |  |  |  | Not relevant |
| Ctri 2023 | CTRI | A RANDOMISED CONTROL TRIAL TO STUDY EFFECT OF BREATHER DEVICE ON RESPIRATORY MUSCLE STRENGTH AND QUALITY OF LIFE IN CABG PATIENTS | https://trialsearch.who.int/Trial2.aspx?TrialID=CTRI/2023/05/053052 | 2023 |  |  |  | Not relevant |
| Ctri 2023 | CTRI | A Randomized Controlled Trial was conducted to see how Pilates exercise compared to physio ball exercise affected the patients trunk control and balance | https://trialsearch.who.int/Trial2.aspx?TrialID=CTRI/2023/02/049723 | 2023 |  |  |  | Wrong intervention < 6 weeks |
| Ctri 2023 | CTRI | A study to compare the effect of two therapies on the improvement of hand functions in stroke patients | https://trialsearch.who.int/Trial2.aspx?TrialID=CTRI/2023/06/054543 | 2023 |  |  |  | Not relevant |
| Ctri 2023 | CTRI | Studying the Combined effect of Swiss ball training and Backward walking on balance and walking in stroke patients | https://trialsearch.who.int/Trial2.aspx?TrialID=CTRI/2023/06/053948 | 2023 |  |  |  | Wrong study design - not RCT |
| Ctri 2023 | CTRI | Task Oriented circuit training for the patient of stroke | https://trialsearch.who.int/Trial2.aspx?TrialID=CTRI/2023/09/057907 | 2023 |  |  |  | Wrong intervention - does not meet exercise definition |
| Ctri 2023 | CTRI | Use of group muscle activation based motor treatment on upper limb motor function in stroke patients | https://trialsearch.who.int/Trial2.aspx?TrialID=CTRI/2023/10/058619 | 2023 |  |  |  | Not relevant |
| Cucato 2022 | Cucato, G.; Snowden, C.; McCone, E.; Nesbitt, C.; Nandhra, S.; Fong, M.; Kane, E.; Rowland, M.; Bhattarai, N.; Court, P.; Bell, O.; Saxton, J. M.; Prentis, J. | Evaluating the feasibility and acceptability of an exercise and behaviour change intervention in socioeconomically deprived patients with peripheral arterial disease: the textpad study protocol | PLoS ONE | 2022 | 17 | 6 | e0269999 | Wrong comparator - co-interventions not balanced between arms |
| Cuesta-Vargas 2023 | Cuesta-Vargas, A. I.; Fuentes-Abolafio, I. J.; Garcí­a-Conejo, C.; Dí­az-Balboa, E.; Trinidad-Fernández, M.; Gutiérrez-Sánchez, D.; Escriche-Escuder, A.; Cobos-Palacios, L.; et al. | Effectiveness of a cardiac rehabilitation program on biomechanical, imaging, and physiological biomarkers in elderly patients with heart failure with preserved ejection fraction (HFpEF): fUNNEL?+?study protocol | BMC Cardiovascular disorders | 2023 | 23 | 1 | 550 | Wrong comparator - co-interventions not balanced between arms |
| Cui 2020 | Cui, Z.; Li, N.; Gao, C.; Fan, Y.; Zhuang, X.; Liu, J.; Zhang, J.; Tan, Q. | Precision implementation of early ambulation in elderly patients undergoing off-pump coronary artery bypass graft surgery: a randomized-controlled Clinical trial | BMC Geriatrics | 2020 | 20 | 1 | 404 | Wrong intervention < 6 weeks |
| Cumming 2016 | Cumming, T.; Linden, T.; Bernhardt, J. | The impact of early mobilisation on cognition: moCA outcomes from avert | Cerebrovascular Diseases (Basel, Switzerland) | 2016 | 42 |  | 5 | Wrong intervention - does not meet exercise definition |
| Cunningham 2016 | Cunningham, P.; Turton, A. J.; Van Wijck, F.; Van Vliet, P. | Task-specific reach-to-grasp training after stroke: development and description of a home-based intervention | Clinical Rehabilitation | 2016 | 30 | 8 | 731-40 | Not relevant |
| Currie 2015 | Currie, K. D.; Bailey, K. J.; Jung, M. E.; McKelvie, R. S.; MacDonald, M. J. | Effects of resistance training combined with moderate-intensity endurance or low-volume high-intensity interval exercise on cardiovascular risk factors in patients with coronary artery disease | Journal of Science & Medicine in Sport | 2015 | 18 | 6 | 637-42 | Wrong comparator - exercise v exercise |
| cv 2019 | cv, R. B. R. | Analysis of the results of an intensive therapy for the legs in the gait and in the balance of patients who suffered stroke | https://trialsearch.who.int/Trial2.aspx?TrialID=RBR-467cv6 | 2019 |  |  |  | Not relevant |
| CyrilleHerkert 2019 | Cyrille Herkert, C.; Kraal, J. J.; Van Loon, E. M. A.; Van Hooff, M.; Brouwers, R. W. M.; Kemps, H. M. C. | Validation of two wrist-worn devices for the assessment of energy expenditure in patients with chronic heart failure and coronary artery disease | European Journal of Preventive Cardiology | 2019 | 26 | 1 | S7 | Not relevant |
| D'Andrea 2022 | D'Andrea, A.; Carbone, A.; Ilardi, F.; Pacileo, M.; Savarese, C.; Sperlongano, S.; Di Maio, M.; Giallauria, F.; Russo, V.; Bossone, E.; Picano, E. | Effects of High Intensity Interval Training Rehabilitation Protocol after an Acute Coronary Syndrome on Myocardial Work and Atrial Strain | Medicina | 2022 | 58 | 3 | 21 | Wrong comparator - exercise v exercise |
| d7yy 2016 | (WHO) International Clinical Trials Registry Platform | Effect of Strength Training on the activation Myoelectric Respiratory, Structure Diaphragmatic, Pulmonary Function and Quality of Life in individuals with Heart Failure | https://trialsearch.who.int/Trial2.aspx?TrialID=RBR-3d7yy4 | 2016 |  |  |  | Not relevant |
| daCostaTorres 2016 | da Costa Torres, D.; dos Santos, P. M. R.; Reis, H. J. L.; Paisani, D. M.; Chiavegato, L. D. | Effectiveness of an early mobilization program on functional capacity after coronary artery bypass surgery: a randomized controlled trial protocol | SAGE Open Medicine | 2016 | 4 | no pagination |  | Wrong intervention < 6 weeks |
| daSilvaFilho 2017 | da Silva Filho, E. M.; Andrade de Albuquerque, J. | Influence of constraint induced movement therapy on functional performance in stroke patients: a randomized Clinical trial | Fisioterapia e Pesquisa | 2017 | 24 | 2 | 184-190 | Not relevant |
| daSilva 2022 | da Silva, N. D.; Andrade-Lima, A.; Chehuen, M. R.; Leicht, A. S.; Brum, P. C.; Oliveira, E. M.; Wolosker, N.; Pelozin, B. R. A.; Fernandes, T.; Forjaz, C. L. M. | Walking Training Increases microRNA-126 Expression and Muscle Capillarization in Patients with Peripheral Artery Disease | Genes | 2022 | 14 | 1 |  | Wrong comparator - active control |
| DaSilvaRibeiro 2015 | Da Silva Ribeiro, N. M.; Dominguez Ferraz, D.; Pedreira, E.; Pinheiro, I.; Da Silva Pinto, A. C.; Gomes Neto, M.; Dos Santos, L. R. A.; Guimaraes Pozzato, M. G.; Silva Pinho, R.; Rodrigues Masruha, M. | Virtual rehabilitation via Nintendo Wii and conventional physical therapy effectively treat post-stroke hemiparetic patients | Topics in Stroke Rehabilitation | 2015 | 22 | 4 | 299-305 | Wrong intervention - does not meet exercise definition |
| DaSilvaRodrigues 2019 | Da Silva Rodrigues, J. C.; Luvizutto, G. J.; Da Costa, R. D. M.; Prudente, R. A.; Da Silva, T. R.; De Souza, J. T.; Da Silveira, Cfdsmp; Rossi, D. A. A.; et al. | Influence of an exercise program on cardiac remodeling and functional capacity in patients with stroke (CRONuS trial): study protocol for a randomized controlled trial | Trials | 2019 | 20 | 1 | 298 | Wrong comparator - active control |
| daSilva 2019 | da Silva, R. S.; da Silva, S. T.; de Souza, J. M.; de Figueiredo, M. C. C.; Mendes, Thaí­s A. S.; de Sena Nunes, M. C.; et al. | Effects of inclined treadmill training on functional and cardiovascular parameters of stroke patients: study protocol for a randomized controlled trial | Trials | 2019 | 20 | 1 | N.PAG-N.PAG | Wrong comparator - exercise v exercise |
| Dai 2022 | Dai, Y.; Huang, F.; Zhu, Y. | Clinical efficacy of motor imagery therapy based on fNIRs technology in rehabilitation of upper limb function after acute cerebral infarction | Pakistan Journal of Medical Sciences | 2022 | 38 | 7 | 1980-1985 | Not relevant |
| Dalal 2018 | Dalal, H.; Jolly, K.; Davis, R.; Doherty, P.; Austin, J.; Van Lingen, R.; Warren, F.; Green, C.; Wingham, J.; Britten, N.; Greaves, C.; Abraham, C.; Singh, S.; Paul, K.; Lang, C.; Smith, K.; Eyre, V.; Hayward, C. | Rehabilitation enablement in chronic heart failure (REACH-HF) a multicentre randomised controlled trial of facilitated self-care rehabilitation intervention in heart failure with reduced ejection fraction | Heart (British Cardiac Society) | 2018 | 104 | 6 | A43-A44 | Wrong comparator - co-interventions not balanced between arms |
| Dalal 2019 | Dalal, H. M.; Taylor, R. S.; Jolly, K.; Davis, R. C.; Doherty, P.; Miles, J.; van Lingen, R.; Warren, F. C.; Green, C.; Wingham, J.; Greaves, C.; Sadler, S.; Hillsdon, M.; Abraham, C.; Britten, N.; Frost, J.; Singh, S.; Hayward, C.; Eyre, V.; Paul, K.; Lang, C. C.; Smith, K. | The effects and costs of home-based rehabilitation for heart failure with reduced ejection fraction: the REACH-HF multicentre randomized controlled trial | European Journal of Preventive Cardiology | 2019 | 26 | 3 | 262-272 | Wrong comparator - co-interventions not balanced between arms |
| DalliPeydró 2022 | Dalli Peydró, E.; Sanz Sevilla, N.; Tuzón Segarra, M. T.; Miró Palau, V.; Sánchez Torrijos, J.; Cosí­n Sales, J. | A randomized controlled Clinical trial of cardiac telerehabilitation with a prolonged mobile care monitoring strategy after an acute coronary syndrome | Clinical Cardiology | 2022 | 45 | 1 | 31-41 | Wrong comparator - exercise v exercise |
| Daly 2016 | Daly, J.; McCabe, J. P.; Monquiewicz, M.; Holcomb, J.; Pundik, S. | Greater recovery of upper limb function in moderate/severely impaired chronic stroke in response to customized whole limb vs prescribed focused training | Stroke | 2016 | 47 | no pagination |  | Wrong study design - not RCT |
| Daly 2019 | Daly, Janis J.; McCabe, Jessica P.; Holcomb, John; Monkiewicz, Michelle; Gansen, Jennifer; Pundik, Svetlana | Long-Dose Intensive Therapy Is Necessary for Strong, Clinically Significant, Upper Limb Functional Gains and Retained Gains in Severe/Moderate Chronic Stroke | NeuroRehabilitation & Neural Repair | 2019 | 33 | 7 | 523-537 | Wrong study design - not RCT |
| Dang 2017 | Dang, W.; Yi, A.; Jhamnani, S.; Wang, S. Y. | Cost-Effectiveness of Multidisciplinary Management Program and Exercise Training Program in Heart Failure | American Journal of Cardiology | 2017 | 120 | 8 | 1338-1343 | Not relevant |
| DanielIvanPerez-Vasquez 2022 | Daniel Ivan Perez-Vasquez, D. I.; Galeana Garcia, M. B.; Soriano Orozco, P. C.; Arenas Fonseca, J. G.; Lara Vargas, J.; Rascon Sabido, R.; Lopez Tiro, D.; Sanchez Reyna, L. D.; Avilez Rosales, J.; Guzman Olea, J.; Gomez Pluma, M. A.; Escutia Cuevas, H. H. | Cardiac rehabilitation programs as a strategy to improve and maintain the quality of life of patients with heart failure: results at 1 year of follow-up | European Journal of Heart Failure | 2022 | Conference |  | Heart Failure 2022 and the World Congress on Acute Heart Failure. Madrid Spain. 24(Supplement 2) (pp 120-121) | Not relevant |
| Danks 2016 | Danks, K. A.; Pohlig, R.; Reisman, D. S. | Combining Fast-Walking Training and a Step activity Monitoring Program to Improve Daily Walking activity After Stroke: a Preliminary Study | Archives of Physical Medicine and Rehabilitation | 2016 | 97 | 9 Suppl | S185-93 | Not relevant |
| Dantas 2023 | Dantas, Mtap; Fernani, Dcgl; Silva, T. D. D.; Assis, I. S. A.; Carvalho, A. C.; Silva, S. B.; Abreu, L. C.; Barbieri, F. A.; Monteiro, C. B. M. | Gait Training with Functional Electrical Stimulation Improves Mobility in People Post-Stroke | International Journal of Environmental Research and Public Health | 2023 | 20 | 9 |  | Not relevant |
[truncated: 1,046,790 more chars]
